# Direct catalytic enantioselective amination of ketones for the formation of tri- and tetrasubstituted stereocenters†
†Electronic supplementary information (ESI) available: Experimental details, and characterization data. See DOI: 10.1039/c8sc00147b


**DOI:** 10.1039/c8sc00147b

**Published:** 2018-02-14

**Authors:** B. M. Trost, J. S. Tracy, T. Saget

**Affiliations:** a Department of Chemistry , Stanford University , Stanford , CA 94305-5080 , USA . Email: bmtrost@stanford.edu

## Abstract

Herein, we report a Zn–ProPhenol catalyzed direct asymmetric amination reaction of unactivated aryl and vinyl ketones using di-*tert*-butyl azodicarboxylate as a cheap and practical electrophilic nitrogen source. Importantly, this methodology works with both α-branched and unbranched ketones for the construction of tri- and tetrasubstituted N-containing stereocenters. The reaction can be run at gram-scale with low catalyst loadings and features a recoverable and reusable ligand. Finally, the enantioenriched hydrazine products can be readily converted into versatile building blocks such as α-amino carbonyl compounds and β-amino alcohols.

## Abstract 2

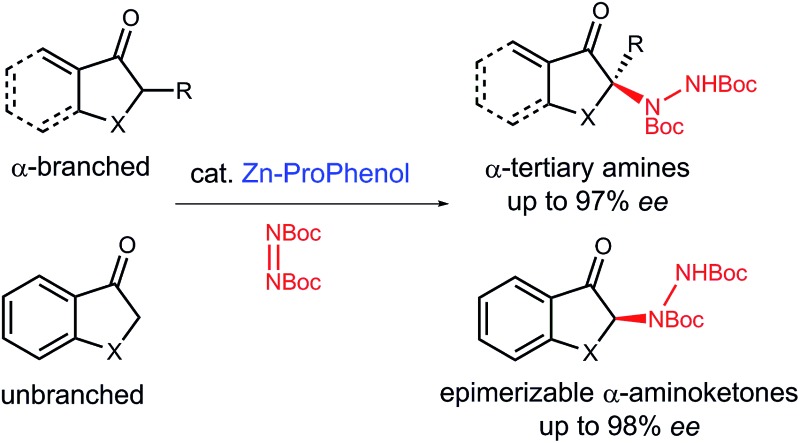
A Zn–ProPhenol catalyzed direct amination of α-branched and unbranched ketones produces α-amino carbonyl and β-amino alcohol products.

## Introduction

**Scheme 1:**
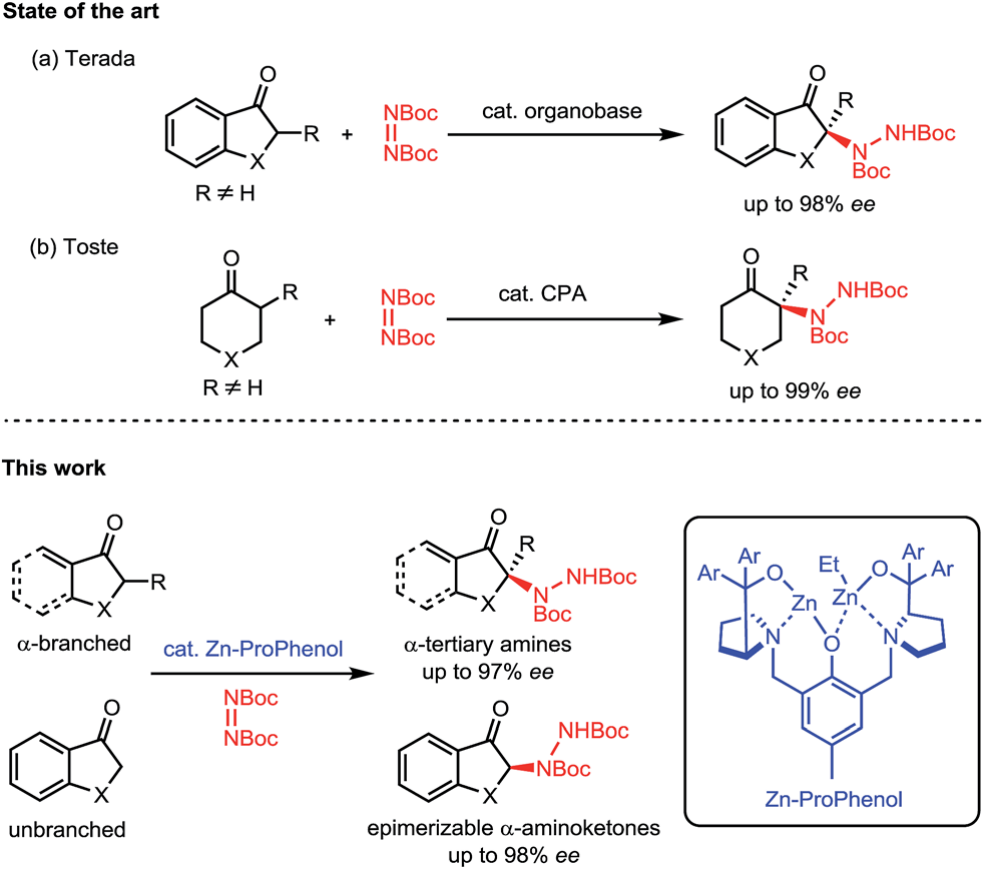
Direct catalytic enantioselective amination of α-branched ketones.

Nitrogen-containing molecules are ubiquitous among natural products and pharmaceuticals. In this regard, the stereoselective formation and functionalization of amines has been an area of continuous interest in synthetic organic chemistry.^1^ Despite considerable advances in this field, the enantioselective synthesis of α-tertiary amines, a common motif in naturally occurring and synthetic bioactive molecules,^2^ still represents a synthetic challenge.^3^ In this context, we report a direct catalytic enantioselective amination of cyclic ketones for the construction of tri- and tetrasubstituted N-containing stereogenic centers. This transformation allows efficient and stereocontrolled access to a broad array of α-amino carbonyl and β-amino alcohol motifs starting from widely available ketone precursors (Scheme 1).

Nitrogen atoms are usually incorporated within organic scaffolds by the addition of a nitrogen-based nucleophile to a carbon-based electrophile, often with the intermediacy of a C=N bond which can undergo further reactivity to afford amines. In comparison, the electrophilic amination of carbon-based nucleophiles has received much less attention.^4^–^6^ This latter strategy is particularly relevant for the enantioselective synthesis of α-tertiary amines, since the catalytic asymmetric addition of nucleophiles to ketimines is an extremely challenging process.^5^ Indeed, the direct catalytic enantioselective α-amination of carbonyl compounds^6^ has proven to be a useful strategy to access molecules decorated with tetrasubstituted N-containing stereogenic centers. However, these reactions rely on the use of aldehydes^7^ or activated carbonyl compounds such as 1,3-dicarbonyls,^8^ α-cyanocarbonyls,^9^ α-keto esters,^10^ and 2-oxindoles.^11^ There are only a few reports of direct enantioselective amination of unactivated ketones^12^,^13^ and only two examples describe the use of α-branched ketones for the synthesis of α-tertiary amines. Terada's initial report utilizing a chiral organosuperbase with cyclic aromatic ketones^14^ (Scheme 1a) was followed by Toste's chiral phosphoric acid methodology with cyclic aliphatic ketones^15^ (Scheme 1b). Both methods require the formation of a tetrasubstituted carbon alpha to the carbonyl to avoid epimerization of the final product. To date, there is no general method allowing for the direct catalytic asymmetric amination of both α-branched and unbranched ketones.

Our group's aza-hemicrown family of ProPhenol ligands form dinuclear main group metal catalysts when treated with alkyl metal reagents such as Et_2_Zn.^16^ The ability of the Zn–ProPhenol catalytic system to promote a dual-activation of both nucleophiles and electrophiles has allowed for the development of a wide range of enantioselective addition reactions, but the use of heteroatom electrophiles has so far not been achieved.^17^ In this context, we wished to evaluate the potential of our Zn–ProPhenol catalysts for amination reactions of unactivated ketones using azodicarboxylates.

## Results and discussion

We initiated our studies with 2-methyl-1-indanone **1a** and di-*tert*-butyl azodicarboxylate as a cheap and practical electrophilic nitrogen source (Table 1). Pleasingly, our standard Zn–ProPhenol catalyst promoted the desired amination in toluene at 40 °C to afford **2a** with promising yield and selectivity (entry 1). Following this initial hit, a screen of Lewis-basic additives was undertaken based upon the previous observation that such additives can significantly impact the catalyst turnover and resulting enantioselectivity of the Zn–ProPhenol catalyst.^17^ The use of triphenylphosphine oxide (TPPO) or *tert*-butyl-*N*-methylcarbamate (**3**) as additives resulted in modest increases in ee (entries 2 and 3). However, bis-phosphine oxide (*R*)-BINAPO provided a significant boost in both yield (97%) and ee (89%) (entry 4). A small but measurable mismatched effect was observed when (*S*)-BINAPO was used, since the same enantiomer of **2a** was obtained albeit with lower selectivities (entry 5). Keeping with bidentate phosphine oxides, additives **4** and **5** were screened, but both resulted in a significant reduction in ee (entries 6 and 7). Finally, while the bis-phosphine oxide of 1,4-bis(diphenylphosphino)butane (dppbO_2_) proved no better than TPPO, the use of the bis-phosphine oxide of 1,2-bis(diphenylphosphino)ethane (dppeO_2_) restored the selectivity and reactivity of the additive (*R*)-BINAPO with the added benefit of being achiral (entries 8 and 9). Switching ligands from (*R*,*R*)-**L1** to the non-C_2_ symmetric (*S*,*S*)-**L2** resulted in an improved enantioselectivity at the expense of yield (entry 10). However, a slight increase of catalyst loading to 15 mol% gave **2a** in high yield and selectivity (entry 11).

**Table 1:** Optimization studies for the amination of 2-methyl-1-indanone^*a*^

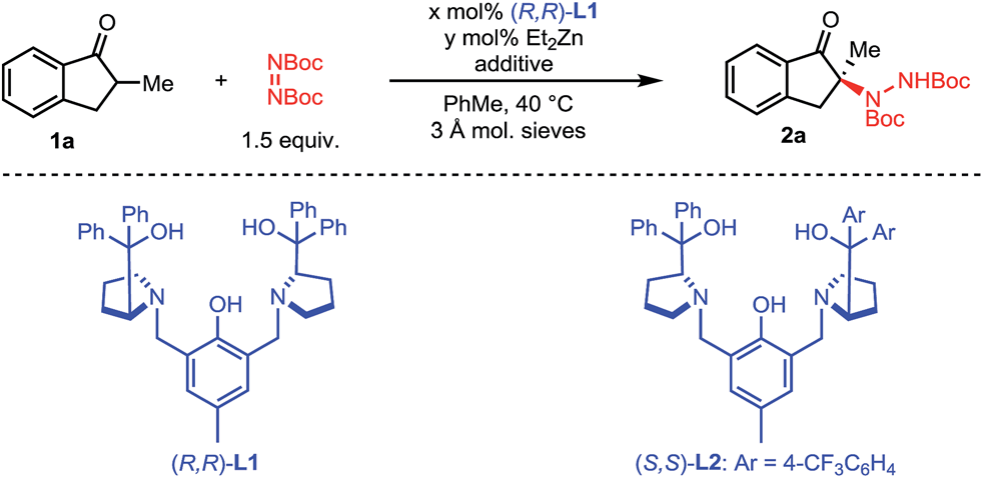					
Entry	*x*	*y*	Additive [mol%]	Yield^*b*^ [%]	ee^*c*^ [%]
1	11	20	None	77	70
2	11	20	TPPO [10]	79	79
3	11	20	**3** [10]	72	78
4	11	20	(*R*)-BINAPO [10]	97	89
5	11	20	(*S*)-BINAPO [10]	73	75
6	11	20	**4** [10]	69	11
7	11	20	**5** [10]	72	51
8	11	20	dppbO_2_ [10]	79	83
9	11	20	dppeO_2_ [10]	93	89
10^*d*^	11	20	dppeO_2_ [10]	66	–91
11^*d*^	16	30	dppeO_2_ [15]	98	–97
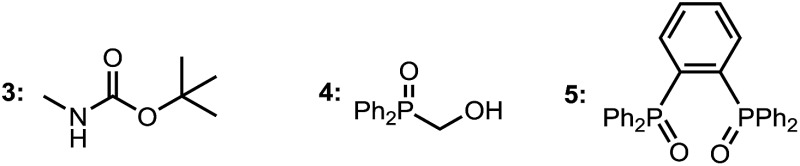					

^*a*^0.20 mmol **1a**, 0.30 mmol di-*tert*-butyl azodicarboxylate, *x* mol% **L1**, *y* mol% ZnEt_2_, 10 mg 3 Å mol. sieves, in 1.0 mL of toluene at 40 °C.

^*b*^Yield of the isolated product.

^*c*^Determined by HPLC on a chiral stationary phase.

^*d*^(*S*,*S*)-**L2** utilized.

**Scheme 2:**
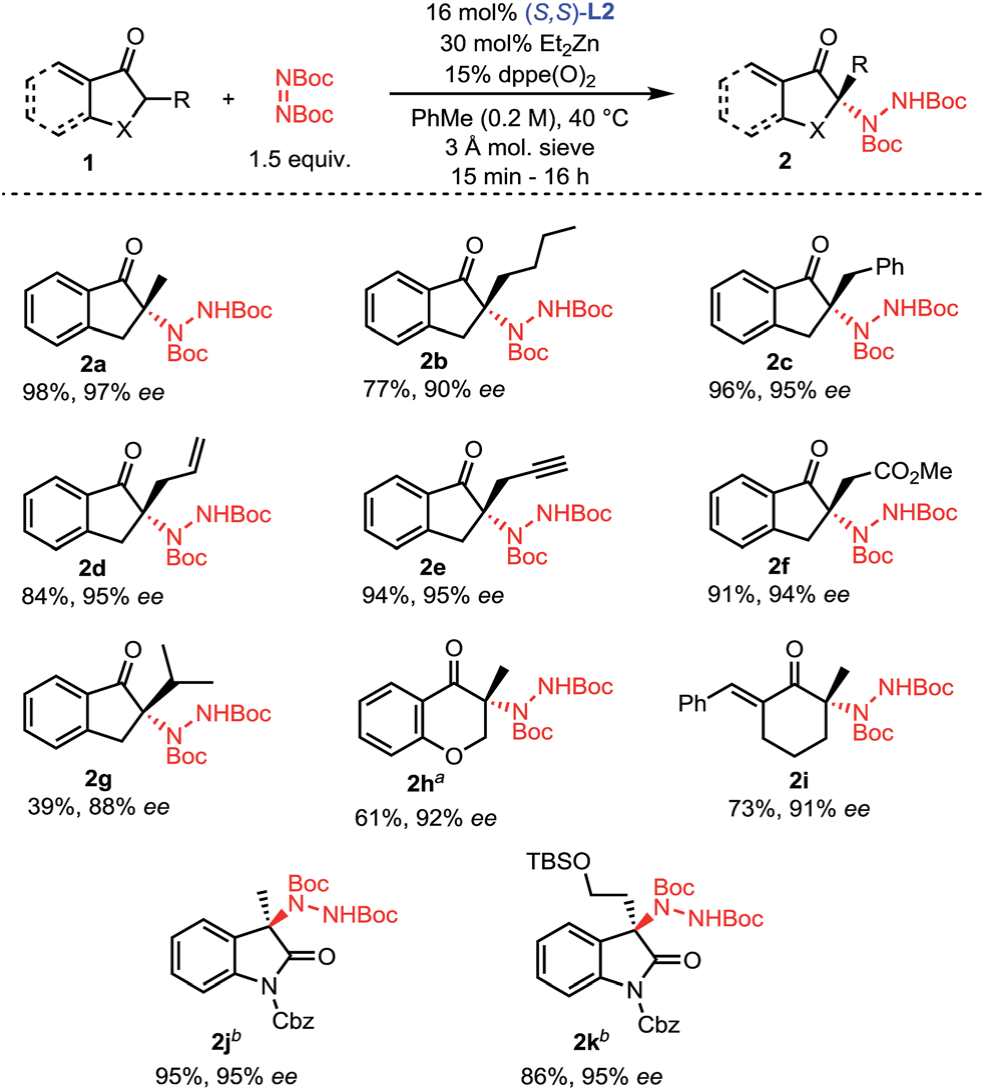
Scope of the amination of α-branched ketones. ^*a*^Reaction performed at 60 °C. ^*b*^Reaction performed with 5.5 mol% **L2**, 10 mol% ZnEt_2_, at 23 °C without any phosphine oxide additive.

With optimized conditions in hand (Table 1, entry 11), the scope of the process was explored (Scheme 2). Indanones having a linear alkyl chain at the 2-position all gave excellent results (**2a–2f**). Of note, functional groups such as methyl esters, alkenes and terminal alkynes are well tolerated (**2d–2f**). Introduction of a bulkier isopropyl group at the 2-position of indanone still afforded product **2g** with good enantioselectivity, albeit with a lower yield. Substrates were not limited to 5-membered rings, as 6-membered aryl and even vinyl ketones showed excellent reactivity and selectivity (**2h–2i**). To further highlight the broad scope of our catalytic amination process, two 3-alkyl-2-oxindole substrates were aminated using a reduced catalyst loading at room temperature (**2j–2k**).

We next turned our attention to the use of unbranched aromatic ketones as nucleophiles in our amination reaction. Importantly, these substrates are not compatible with either Toste's or Terada's protocols due to product epimerization from the strongly acidic or strongly basic catalysts required. We felt the mild reaction conditions provided by our bifunctional Zn–ProPhenol catalyst should be perfectly suited for such challenging substrates.

When 1-tetralone **1l** was reacted with 2.0 equivalents of di-*tert*-butyl azodicarboxylate in THF (0.4 M) at room temperature using 11 mol% of (*R*,*R*)-**L1** and 20 mol% of diethylzinc, the desired amination product **2l** was isolated in a promising 66% yield and 88% ee (Table 2, entry 1). Lowering the equivalents of electrophile to 1.1 resulted in an increase in yield without impacting the ee (entry 2). The molecular sieves were found to be important to catalyst turnover but not selectivity (entry 3). Decreasing the reaction concentration to 0.25 M improved the selectivity to 94% ee without sacrificing catalyst activity, while performing the reaction at 4 °C significantly impacted catalyst turnover (entries 4 and 5).

**Table 2:** Optimization studies for the amination of 1-tetralone^*a*^

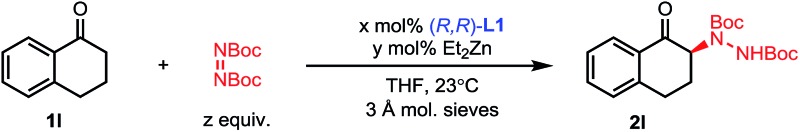						
Entry	*x*	*y*	*z*	Conc.	Yield^*b*^ [%]	ee^*c*^ [%]
1	11	20	2.0	0.4 M	66	88
2	11	20	1.1	0.4 M	86	86
3^*d*^	11	20	1.1	0.4 M	49	90
4	11	20	1.1	0.25 M	86	94
5^*e*^	11	20	1.1	0.25 M	28	87

^*a*^0.20 mmol **1l**, *z* equiv di-*tert*-butyl azodicarboxylate, *x* mol% **L1**, *y* mol% ZnEt_2_, 10 mg 3 Å mol. sieves, in 0.8 mL of THF at rt.

^*b*^Yield of the isolated product.

^*c*^Determined by HPLC on a chiral stationary phase.

^*d*^No 3 Å mol. sieves.

^*e*^Reaction performed at 4 °C.

**Scheme 3:**
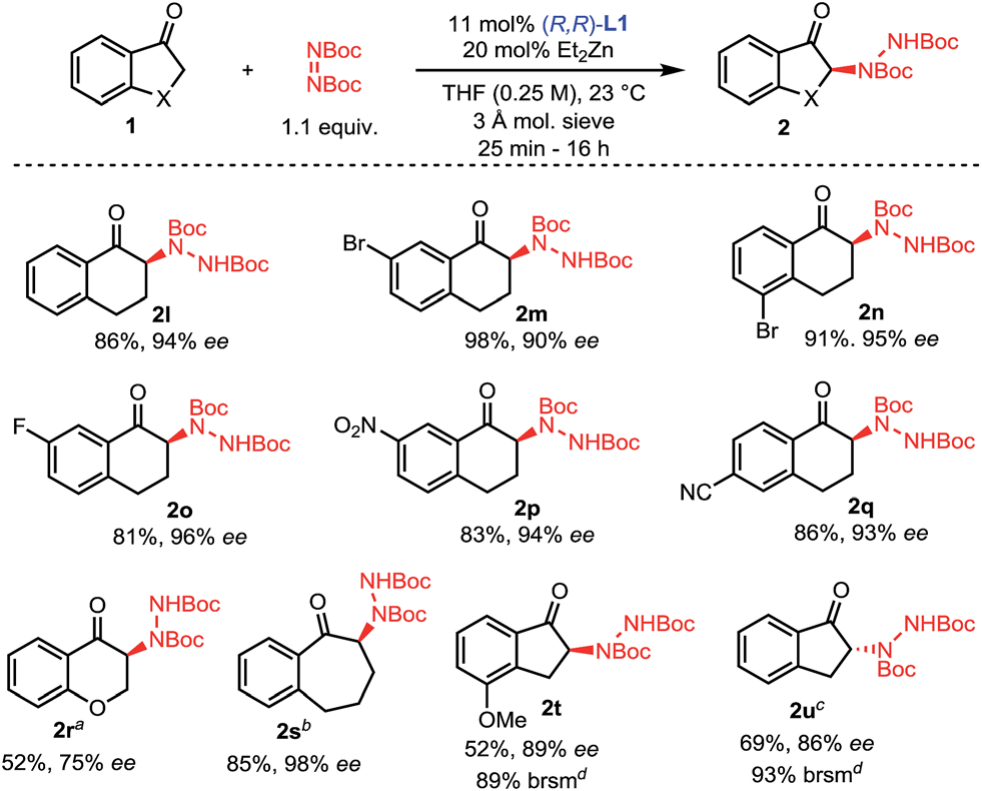
Scope of the amination of unbranched ketones. ^*a*^Reaction performed at 4 °C. ^*b*^Reaction performed with 16 mol% **L1** and 30 mol% ZnEt_2_ at 0.4 M. ^*c*^Reaction performed with 5.5 mol% (*S*,*S*)-**L2** and 10 mol% ZnEt_2_. ^*d*^Based on recovered starting material (brsm).

The scope of this amination process was then explored (Scheme 3). A range of 1-tetralones substituted at the 5-, 6- and 7-positions were successfully aminated in high yields and selectivities (**2l–2q**). Of note, tetralones **1o**, **1p** and **1q** substituted with strongly electron-withdrawing groups showed no evidence of epimerization under the reaction conditions, showcasing the mild activation provided by the Zn–ProPhenol bifunctional catalyst. Importantly, such electron deficient tetralones were poor substrates for previously reported amination methods. The use of 1-chromanone **1r** was challenging and lower temperatures as well as partial conversion were required to prevent significant epimerization of **2r**. On the other hand, 1-benzosuberone **1s** gave excellent results using our protocol. Finally, the 1-indanone class of substrates was studied. This class of substrates proved to be a significant challenge for previous amination reactions because they afford highly epimerizable products which were isolated with poor enantioselectivities.13c,e Using our mild Zn–ProPhenol catalyst and stopping the reaction at partial conversion led to the isolation of products **2t** and **2u** with great enantioselectivities and good yields. In the case of substrate **1u**, the use of **L2** was beneficial.

**Scheme 4:**
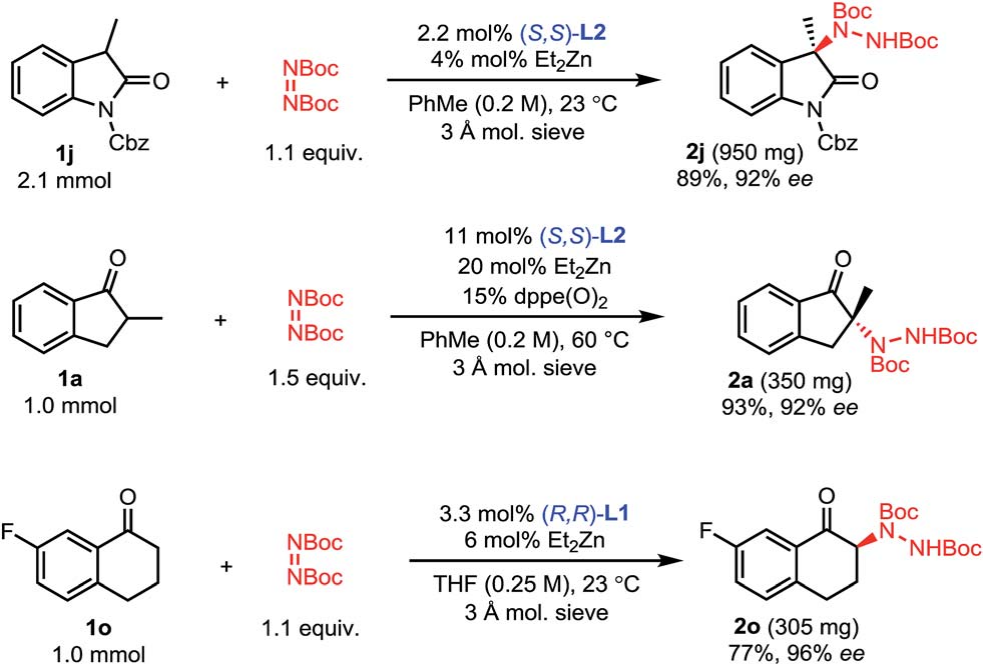
Large scale reactions.

To showcase the scalability and practicality of the process, we performed some amination reactions on a 1 to 2 mmol scale with reduced catalyst loadings (Scheme 4). Amination of oxindole **1j** was scaled to 2.1 mmol using 2 mol% catalyst without any significant change in outcome. Similarly, the amination of 2-methyl-1-indanone **1a** could be run on a 1 mmol scale using 10 mol% of catalyst without a significant impact on the efficiency of the reaction. Finally, the amination of 7-fluorotetralone **1o** was performed on a 1 mmol scale with 3 mol% catalyst and **2o** was obtained with comparably excellent yield and enantioselectivity.

**Scheme 5:**
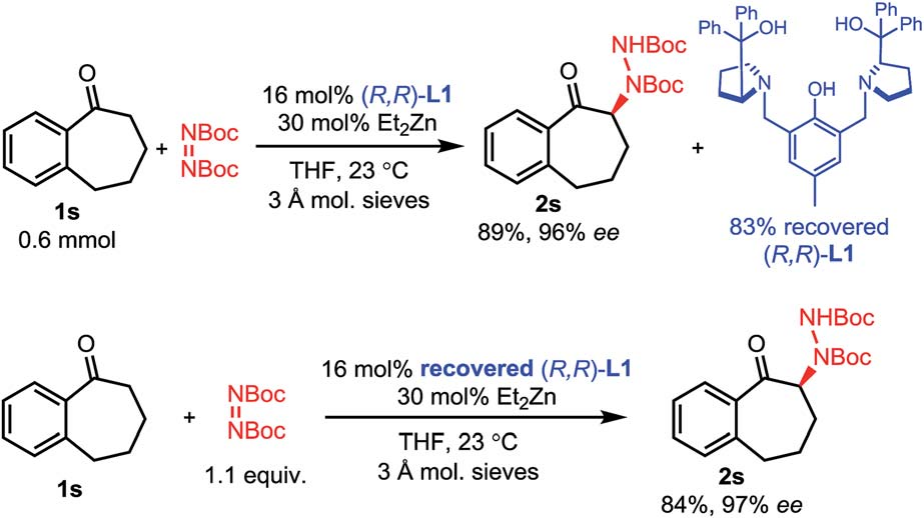
Recovery of ligand.

While some of these amination processes require relatively high catalyst loadings, these loadings can be mitigated by the easy recovery of the ProPhenol ligand through column chromatography and a subsequent acid–base extraction. To showcase this feature, the amination of 1-benzosuberone **1s** was performed on a 0.6 mmol scale using our conditions from Scheme 3 (Scheme 5). After workup and purification, 83% of ligand **L1** was recovered. This recovered ligand was then reused in a subsequent reaction with no loss of reactivity or selectivity.

**Scheme 6:**
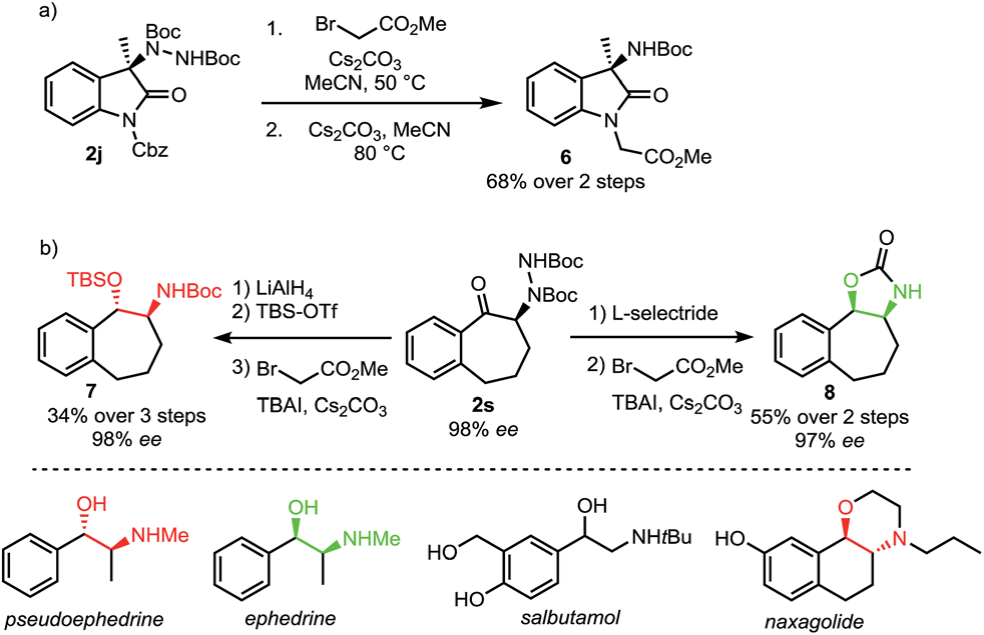
Cleavage of N–N bond to form amino products.

A requirement for any useful amination technique utilizing dialkyl azodicarboxylate electrophiles is to show that the resulting hydrazines can be readily transformed into synthetically desirable amino products. In this respect, the use of di-*tert*-butyl azodicarboxylate represents a significant improvement over related methods requiring use of the less readily deprotected dimethyl azodicarboxylate (DMAD) or diethyl azodicarboxylate (DEAD).13a,c,e To highlight the utility of our products, oxindole **2j** was converted to Boc-amine **6** in a two-step sequence involving an alkylation with methyl bromoacetate followed by an E1cB elimination promoted by cesium carbonate (Scheme 6a).^18^ In addition, both *syn* and *anti* amino alcohol derivatives could be accessed from ketone **2s** with no erosion of ee (Scheme 6b). Bulky l-selectride delivers hydride from the more sterically accessible face to form the *syn*-product while the use of lithium aluminium hydride results in an internal delivery of the hydride to form the *anti*-product.^19^ We then developed a one-pot procedure for the subsequent alkylation–elimination cleavage of the N–N bond to access compounds *syn*-**7** and *anti*-**8** in 3 and 2 steps respectively. Overall, this approach allows a stereodivergent synthesis of cyclic compounds incorporating a phenylethanolamine motif. Such a motif can be found in many bioactive natural products, including the *Ephedra* alkaloids.^20^ It is also a common motif in marketed and clinical drugs as exemplified by salbutamol, a bronchodilator used to treat asthma, and naxagolide, Merck's antiparkinson compound.^21^,^22^

**Scheme 7:**
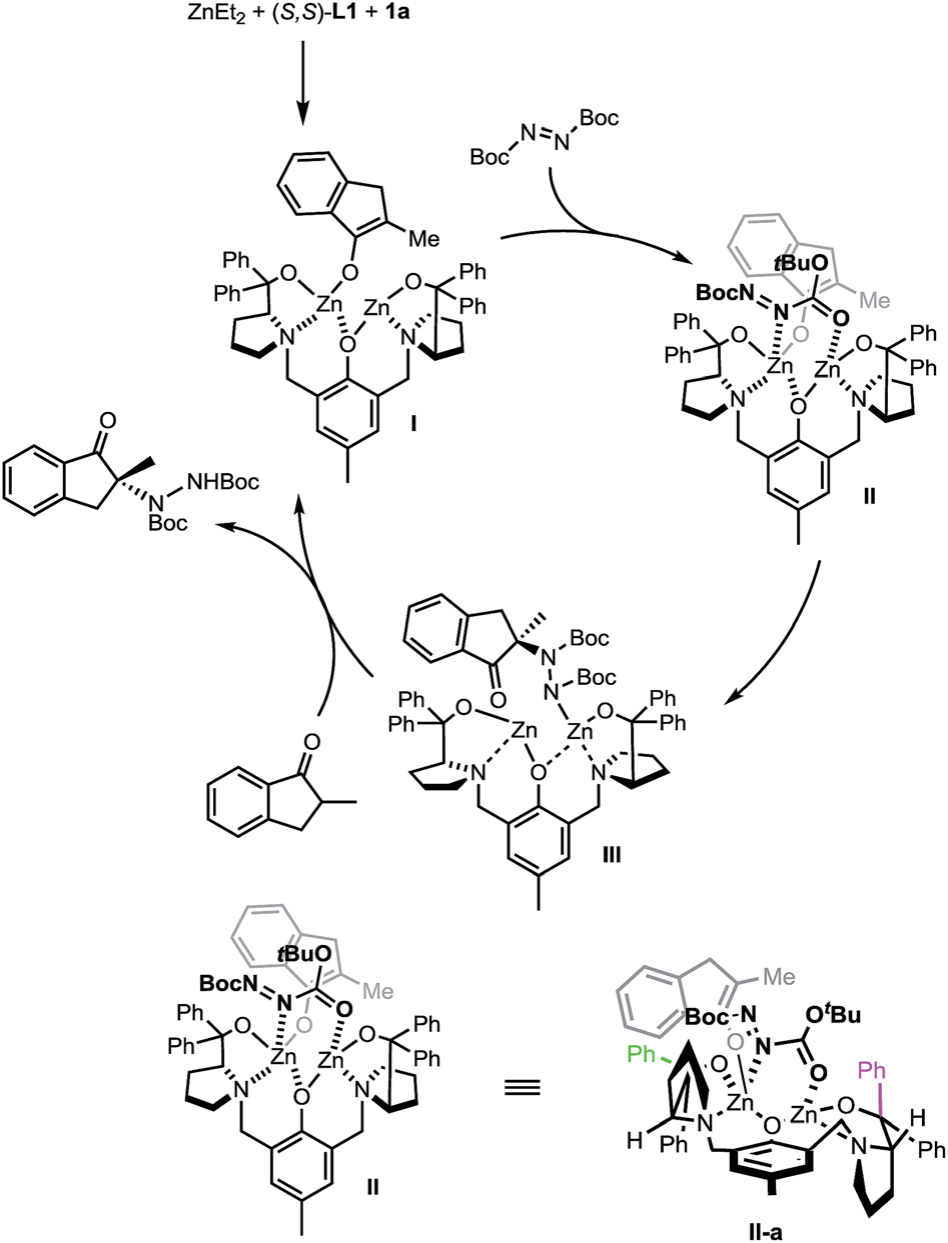
Proposed catalytic cycle.

A proposed catalytic cycle is depicted in Scheme 7 using (*S*,*S*)-**L1** instead of the non-C_2_ symmetric (*S*,*S*)-**L2** for purposes of simplicity. The initially formed dinuclear Zn–ProPhenol catalyst enters the catalytic cycle through a deprotonation of the ketone to form complex **I**. Bi-dentate coordination of the electrophile produces complex **II** which sets the electrophile up for attack from the re-face of the enolate. The resulting zinc–hydrazine product (**III**) serves as a source of base to deprotonate another equivalent of ketone while simultaneously releasing the product. Importantly, the stereochemical outcome of the reaction is in perfect agreement with our recent work on related Mannich reactions.^23^ The source of selectivity in complex **II** is made clearer when viewed down the axis of the phenol (**IIa**, bottom of Scheme 7). From this orientation, steric interactions are minimized when the bulk of the reactants is directed, as shown in **IIa**, towards the western pseudo-equatorial phenyl group (green) and would be maximized if the bulk of the reactants is instead positioned towards the eastern pseudo-axial phenyl group (purple).

## Conclusion

In conclusion, we have developed a mild catalytic method for the direct amination of aromatic and vinyl ketones as well as of oxindoles. The procedure is unique in its ability for a single catalyst to form tri- and tetrasubstituted N-containing stereocenters with high enantioselectivities, even those whose products contain readily epimerizable indanone or electron-withdrawing tetralone scaffolds that do not fare well under previously reported conditions.^13^ This method negates the need for cryogenic temperatures and multi-day reaction times characteristic of previous reports.^14^,^15^ The reactions can be scaled up with reduced catalyst loadings without impacting the efficiency of the process and the ligand can be recovered and reused, making the effective catalyst loadings very low. Finally, the resulting hydrazine products are readily converted into valuable amines utilizing a simplified one-pot alkylation–elimination procedure.

## Conflicts of interest

There are no conflicts of interest to declare.

## Supplementary Material

Supplementary information
