# Supplementary material for: Direct catalytic enantioselective amination of ketones for the formation of tri- and tetrasubstituted stereocenters
Source: Chem Sci. 2018 Feb 14;9(11):2975–80. doi: 10.1039/c8sc00147b (PMC5897889; doi:10.1039/c8sc00147b)
Supplement: Supplementary file 1 [file SC-009-C8SC00147B-s001.pdf]

Electronic Supplementary Material (ESI) for Chemical Science.
This journal is © The Royal Society of Chemistry 2018

Supporting Information

Direct Catalytic Enantioselective Amination of Ketones for the Formation of Tri- and Tetrasubstituted Stereocenters

B. M. Trost,* J. S. Tracy and T. Saget

Department of Chemistry, Stanford University
Stanford, CA 94305-5080 (USA)
E-mail: bmtrost@stanford.edu

Table of Contents

I. General Information……………………………………………………..…S2
II. Experimental Procedures and Compound Characterization………S3
III. SI References…………………………………………………………S34
IV. Copies of NMR Spectra…………………………………………......S35

I. General Information

All reactions were carried out in oven- or flame-dried glassware under a nitrogen atmosphere. THF was distilled from a sodium-benzophenone still under argon, toluene and acetonitrile was stored under an atmosphere of air over 4 Å molecular sieves. All commercial reagents were used without further purification unless states otherwise. Flash chromatography was performed with 0.035-0.070 µm Silica Gel (Acros). ^{1}H and ^{13}C NMR spectroscopy was performed at ambient temperature with $CDCl_3$ as the solvent on a Mercury NMR at 400 (^{1}H) or 100 (^{13}C) MHz, an Inova NMR at 500 (^{1}H) or 125 (^{13}C) MHz, and an Inova NMR at 600 (^{1}H) MHz unless otherwise indicated. Chemical shifts are reported in ppm relative to tetramethylsilane or residual protonated solvent. All ^{13}C NMR spectra are proton decoupled. When high temperature NMR is utilized with epimerizable products, the *ee* values reported are those obtained before the material is subjected to the high temperature NMR. Infrared spectroscopy data was recorded on sodium chloride plates as thin films made from the indicated solvent on a Thermo Scientific Nicolet IR100 FT-IR spectrometer. Analytical thin layer chromatography was performed using silica gel 60-F_{254} plates (Millipore) and visualized with a UV lamp (254 nm), or using a solution of $KMnO_4$-K_2CO_3 in water followed by heating. High-resolution mass spectra were obtained in the Vincent Coates Foundation Mass Spectrometry Laboratory, Stanford University Mass Spectrometry (http://mass-spec.stanford.edu).

II. Experimental Procedures and Compound Characterization

ProPhenol Ligands:

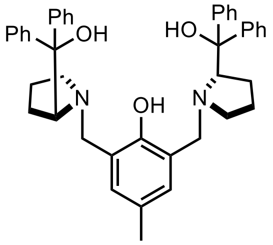

(*R,R*)-**L1**

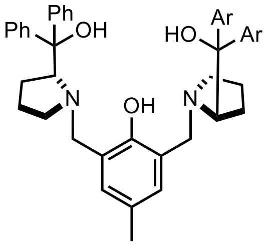

(*S,S*)-**L2**: Ar = 4-$CF_3C_6H_4$

di-tert-butyl 1-(2-methyl-1-oxo-2,3-dihydro-1H-inden-2-yl)hydrazine-1,2-dicarboxylate **1a**

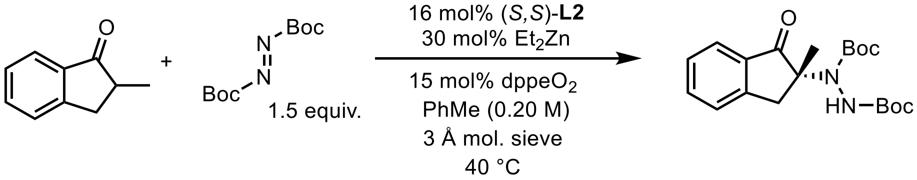

(*S,S*)-**L2** (24.8 mg, 0.0320 mmol), 2-methyl-1-indanone (29.2 mg, 0.200 mmol), and activated 3 Å molecular sieves (5 mg) were measured into a microwave vial equipped with stir bar. The flask was placed under a nitrogen atmosphere and PhMe (0.6 mL) was added. After stirring for 15 minutes, diethylzinc (1.0 M in hexanes, 60µL, 0.060 mmol) was added and the reaction was allowed to stir for an additional 15 minutes. At this time, a premixed (15 min) PhMe (0.4 mL) solution of di-*tert*-butyl azodicarboxylate (69.1 mg, 0.300 mmol) and 3 Å molecular sieves (5 mg) was added. The reaction was allowed to stir at 40 °C for 16 h at which point the reaction mixture was filtered through a small plug of fluorosil. The residue was concentrated under reduced pressure using rotary evaporation. The resulting residue was purified via flash chromatography (SiO2; DCM to petroleum ether / EtOAc 6:1 to petroleum ether / EtOAc 5:1 to petroleum ether / EtOAc 4:1) to afford 73.8 mg (98 %) of a clear oil.

IR($CDCl_3$) 3316, 2979, 2933, 1730, 1611, 1478, 1368, 1302, 1251, 1160, 1119, 1057, 733 cm^{-1}. ^{1}H NMR (500 MHz, 75 °C, Benzene-d_6) δ 7.77 (d, *J* = 7.6 Hz, 1H), 7.17 (t, *J* = 7.6 Hz, 1H), 7.06 – 6.95 (m, 2H), 6.26 (s, 1H), 3.89 (d, *J* = 17.2 Hz, 1H), 2.97 (d, *J* = 17.1 Hz, 1H), 1.41 (s, 9H), 1.20 (2 S, 12H total). ^{13}C NMR (126 MHz, 75 °C, Benzene) δ 202.95, 156.39, 154.23, 150.90, 135.24, 134.45, 127.42, 126.53, 124.85, 81.92, 80.70, 68.30, 42.56, 28.40, 28.16, 23.09. HRMS (EI) calculated for $C_{20}H_{28}N_2O_5$ (Na^+) 399.1896, found: 399.1896. R_f (petroleum ether / EtOAc 4:1) = 0.35. $[\alpha]_D^{23}$ = -10.9 (c = 0.97, $CHCl_3$). HPLC: (*Chiralpak® IA*, heptane-isopropanol 95:5, 0.8 mL/min, 254 nm): t_R(minor) = 7.5 min, t_R(major) = 21.5 min, 97% *ee*.

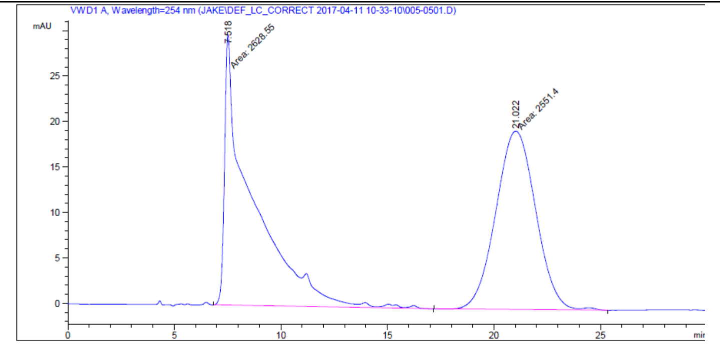

Peak #	RetTime [min]	Type	Width [min]	Area mAU *s	Height [mAU]	Area %
1	7.518	MM	1.4717	2628.55298	29.76828	50.7447
2	21.022	MM	2.1680	2551.40283	19.61428	49.2553

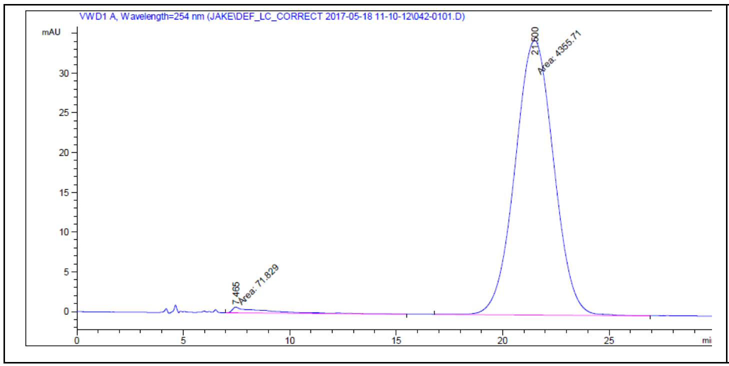

Peak #	RetTime [min]	Type	Width [min]	Area mAU *s	Height [mAU]	Area %
1	7.465	MM	1.7017	71.82899	7.03502e-1	1.6223
2	21.500	MM	2.1015	4355.70703	34.54463	98.3777

To determine the absolute stereochemistry of the tetrasubstituted ketone products, the optical rotation of the title compound was compared to that reported by Terada. $[\alpha]_D^{25} = 12.6$ (c = 1.01, $CHCl_3$). As the signs of the optical rotations are opposite, we assigned the stereochemistry of our products to be opposite of that reported by Terada.[1] All other tetrasubstituted ketone products are assigned by analogy to the title compound.

Large-scale procedure:
(*S*,*S*)-**L2** (85.2 mg, 0.110 mmol), 2-methyl-1-indanone (146 mg, 1.00 mmol), and activated 3 Å molecular sieves (25 mg) were measured into a microwave vial equipped with stir bar. The flask was placed under a nitrogen atmosphere and PhMe (3.0 mL) was added. After stirring for 15 minutes, diethylzinc (1.0 M in hexanes, 200 µL, 0.20 mmol) was added and the reaction was allowed to stir for an additional 15 minutes. At this time, a premixed (15 min) PhMe (2 mL) solution of di-*tert*-butyl azodicarboxylate (345 mg, 1.50 mmol) and 3 Å molecular sieves (25 mg) was added. The reaction was allowed to stir at 60 °C for 16 h at which point the reaction mixture was filtered through a small plug of fluorosil. The residue was concentrated under reduced pressure using rotary evaporation. The resulting residue was purified via flash chromatography (SiO2; petroleum ether / EtOAc 6:1) to afford 0.35 g (93 %) of a clear oil.

HPLC: (*Chiralpak® IA*, heptane-isopropanol 95:5, 0.8 mL/min, 254 nm): t_R(minor) = 7.1 min, t_R(major) = 19.3 min, 92% *ee*.

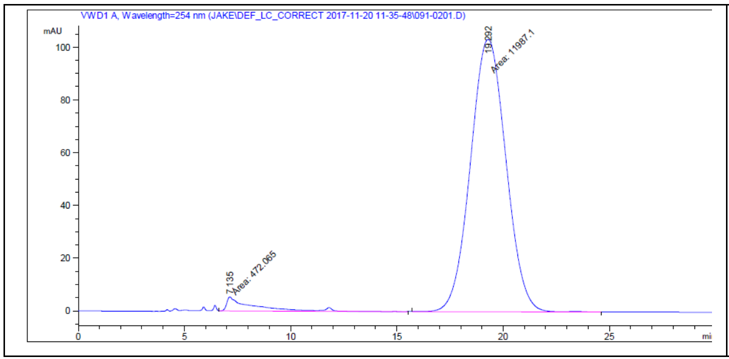

Peak #	RetTime [min]	Type	Width [min]	Area mAU *s	Height [mAU]	Area %
1	7.135	MM	1.4782	472.06458	5.32269	3.7889
2	19.292	MM	1.9345	1.19871e4	103.27386	96.2111

di-tert-butyl 1-(2-butyl-1-oxo-2,3-dihydro-1H-inden-2-yl)hydrazine-1,2-dicarboxylate **2b**

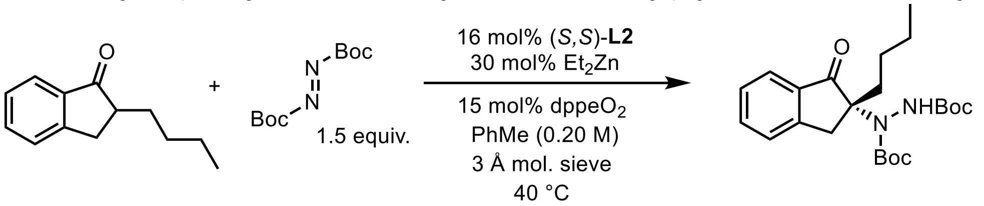

(*S*,*S*)-**L2** (24.8 mg, 0.0320 mmol), 2-butyl-1-indanone (37.7 mg, 0.200 mmol), and activated 3 Å molecular sieves (5 mg) were measured into a microwave vial equipped with stir bar. The flask was placed under a nitrogen atmosphere and PhMe (0.6 mL) was added. After stirring for 15 minutes, diethylzinc (1.0 M in hexanes, 60μL, 0.060 mmol) was added and the reaction was allowed to stir for an additional 15 minutes. At this time, a premixed (15 min) PhMe (0.4 mL) solution of di-*tert*-butyl azodicarboxylate (69.1 mg, 0.300 mmol) and 3 Å molecular sieves (5 mg) was added. The reaction was allowed to stir at 40 °C for 16 h at which point the reaction mixture was filtered through a small plug of fluorosil. The residue was concentrated under reduced pressure using rotary evaporation. The resulting residue was purified via flash chromatography (SiO2; DCM to petroleum ether / EtOAc 6:1 to petroleum ether / EtOAc 5:1 to petroleum ether / EtOAc 4:1) to afford 64.1 mg (77 %) of a clear oil.

IR($CDCl_3$) 3348, 2929, 1726, 1613, 1466, 1368, 1251, 1159 cm^{-1}. ^{1}H NMR (500 MHz, 75 °C, Benzene-d_6) δ 7.77 (d, J = 7.6 Hz, 1H), 7.21 – 7.12 (obscured t, 1H), 7.05 (d, J = 7.7 Hz, 1H), 6.99 (t, J = 7.5 Hz, 1H), 6.32 – 6.16 (m, 1H), 3.92 (d, J = 17.4 Hz, 1H), 3.11 (d, J = 17.2 Hz, 1H), 1.96 – 1.84 (m, 1H), 1.74 – 1.60 (m, 1H), 1.43 (s, 9H), 1.38 – 1.28 (m, 2H), 1.22 (s, 9H), 1.12 – 1.05 (m, 2H), 0.68 (t, J = 6.8 Hz, 3H). ^{13}C NMR (126 MHz, 75 °C, Benzene) δ 202.41, 156.56, 154.56, 151.30, 136.85, 134.37, 127.36, 126.15, 124.35, 82.00, 80.81, 70.93, 41.35, 36.79, 28.43, 28.16, 26.13, 23.47, 13.60. HRMS (EI) calculated for $C_{23}H_{34}N_2O_5$ (Na^+) 441.2365, found: 441.2347. R_f (petroleum ether / EtOAc 4:1) = 0.41. $[\alpha]_D^{23}$ = 15.3 (c = 0.83, $CHCl_3$). HPLC: (*Chiralpak® IA*, heptane-isopropanol 95:5, 0.8 mL/min, 254 nm): t_R(minor) = 7.7 min, t_R(major) = 16.2 min, 90% *ee*.

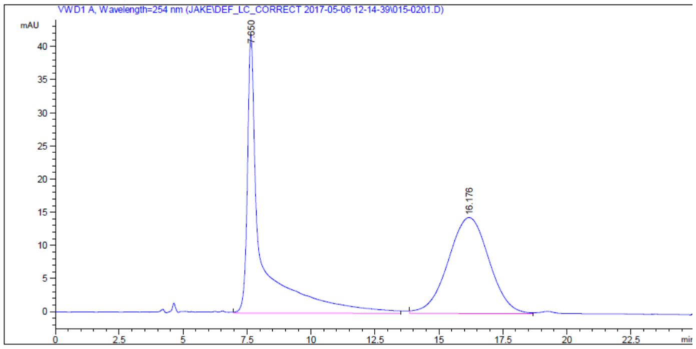

Peak #	RetTime [min]	Type	Width [min]	Area mAU *s	Height [mAU]	Area %
1	7.650	BB	0.5044	1586.54810	42.03174	49.5588
2	16.176	BV	1.6824	1614.79919	14.48181	50.4412

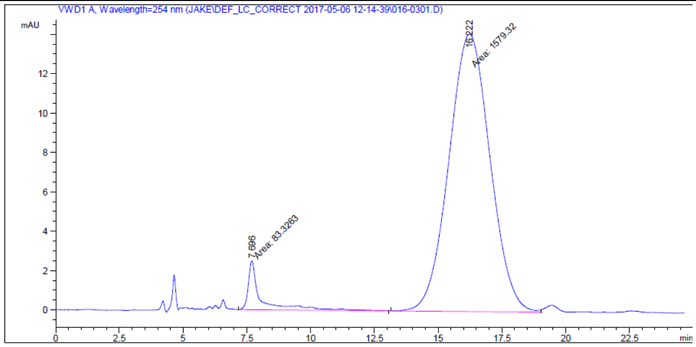

Peak #	RetTime [min]	Type	Width [min]	Area mAU *s	Height [mAU]	Area %
1	7.696	MM	0.5608	83.32632	2.47642	5.0117
2	16.222	MM	1.8685	1579.31616	14.08702	94.9883

di-tert-butyl (R)-1-(2-benzyl-1-oxo-2,3-dihydro-1H-inden-2-yl)hydrazine-1,2-dicarboxylate **2c**

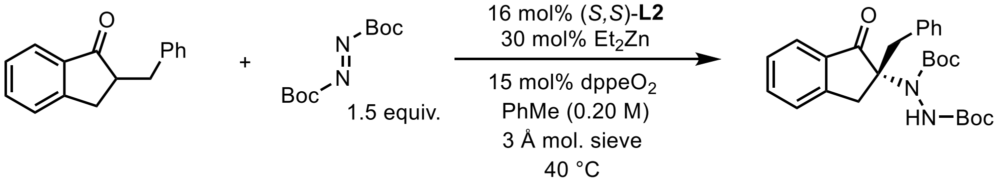

(*S*,*S*)-**L2** (24.8 mg, 0.0320 mmol), 2-benzyl-1-indanone (44.5 mg, 0.200 mmol), and activated 3 Å molecular sieves (5 mg) were measured into a microwave vial equipped with a stir bar. The flask was placed under a nitrogen atmosphere and PhMe (0.6 mL) was added. After stirring for 15 minutes, diethylzinc (1.0 M in hexanes, 60μL, 0.060 mmol) was added and the reaction was allowed to stir for an additional 15 minutes. At this time, a premixed (15 min) PhMe (0.4 mL) solution of di-*tert*-butyl azodicarboxylate (69.1 mg, 0.300 mmol) and 3 Å molecular sieves (5 mg) was added. The reaction was allowed to stir at 40 °C for 15 h at which point the reaction mixture was filtered through a small plug of fluorosil. The residue was concentrated under reduced pressure using rotary evaporation. The resulting residue was purified via flash chromatography (SiO2; petroleum ether / EtOAc 7:1) to afford 86.7 mg (96 %) of a clear oil.

IR(C_6D_6) 3346, 3303, 2979, 2932, 1723, 1611, 1477, 1456, 1392, 1368, 1328, 1250, 1159, 1048, 755, 729, 702 cm^{-1}. ^{1}H NMR (500 MHz, 75 °C, Benzene-d_6) δ 7.58 (d, *J* = 7.6 Hz, 1H), 7.00 (d, *J* = 7.4 Hz, 2H), 6.92 (t, *J* = 7.6 Hz, 1H), 6.84 – 6.70 (m, 5H), 6.29 (s, 1H), 3.91 (s, 1H), 3.41 (d, *J* = 17.4 Hz, 1H), 3.23 (d, *J* = 12.7 Hz, 1H), 3.08 (d, *J* = 12.7 Hz, 1H), 1.42 (series of s, 9H), 1.22 (s, 9H). ^{13}C NMR (126 MHz, 75 °C, Benzene) δ 203.15, 156.50, 154.34, 151.29, 137.39, 134.94, 134.04, 130.95, 126.87, 125.63, 123.87, 82.13, 80.89, 71.34, 42.80, 39.78, 28.45, 28.14. Two aromatic carbon peaks are obscured in the spectrum. HRMS (EI) calculated for $C_{26}H_{32}N_2O_5$ (Na^+) 475.2203, found: 475.2203. $[\alpha]_D^{23}$ = 50.8 (c = 1.76, $CHCl_3$). R_f (petroleum ether / EtOAc 6:1) = 0.27. HPLC: (*Chiralpak® IA*, heptane-isopropanol-triethylamine 95:5:0.5, 0.8 mL/min, 254 nm): t_R(minor) = 7.9 min, t_R(major) = 17.3 min, 95% *ee*.

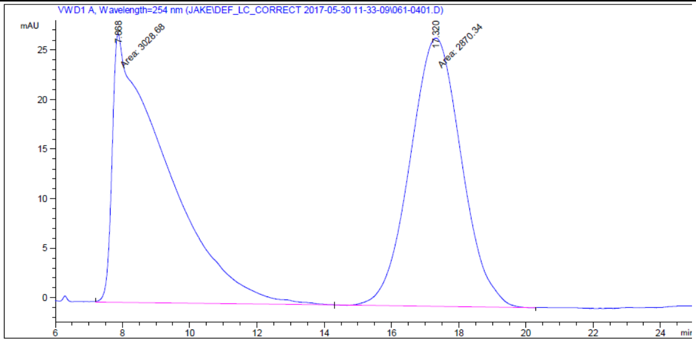

Peak #	RetTime [min]	Type	Width [min]	Area mAU *s	Height [mAU]	Area %
1	7.868	MM	1.8637	3028.67554	27.08494	51.3421
2	17.320	MM	1.7678	2870.33789	27.06071	48.6579

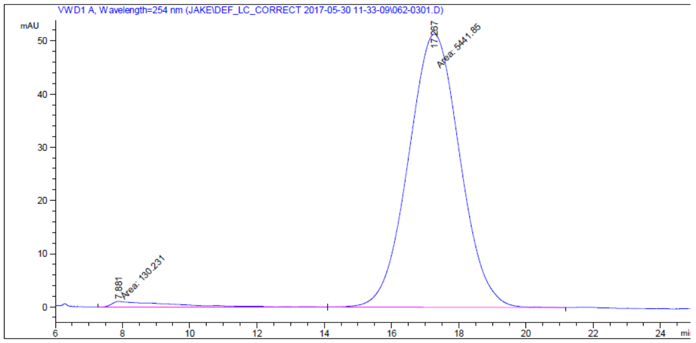

Peak #	RetTime [min]	Type	Width [min]	Area mAU *s	Height [mAU]	Area %
1	7.881	MM	2.0651	130.23111	1.05106	2.3372
2	17.267	MM	1.7675	5441.85010	51.31544	97.6628

di-tert-butyl 1-(2-allyl-1-oxo-2,3-dihydro-1H-inden-2-yl)hydrazine-1,2-dicarboxylate **2d**

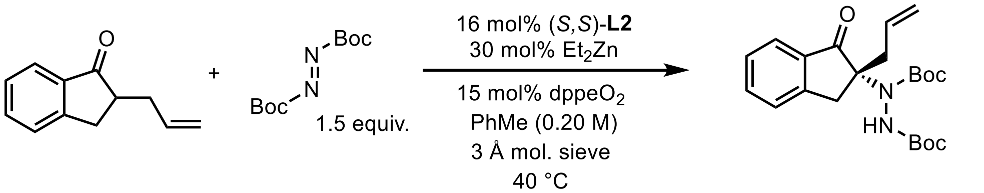

(*S*,*S*)-**L2** (24.8 mg, 0.0320 mmol), 2-allyl-1-indanone (34.4 mg, 0.200 mmol), and activated 3 Å molecular sieves (5 mg) were measured into a microwave vial equipped with stir bar. The flask was placed under a nitrogen atmosphere and PhMe (0.6 mL) was added. After stirring for 15 minutes, diethylzinc (1.0 M in hexanes, 60μL, 0.060 mmol) was added and the reaction was allowed to stir for an additional 15 minutes. At this time, a premixed (15 min) PhMe (0.4 mL) solution of di-*tert*-butyl azodicarboxylate (69.1 mg, 0.300 mmol) and 3 Å molecular sieves (5 mg) was added. The reaction was allowed to stir at 40 °C for 16 h at which point the reaction mixture was filtered through a small plug of fluorosil. The residue was concentrated under reduced pressure using rotary evaporation. The resulting residue was purified via flash chromatography (SiO2; DCM to petroleum ether / EtOAc 6:1 to petroleum ether / EtOAc 5:1 to petroleum ether / EtOAc 4:1) to afford 67.8 mg (84 %) of a clear oil.

IR($CDCl_3$) 3379, 2982, 2937, 1723, 1611, 1477, 1368, 1249, 1158, 754 cm^{-1}. ^{1}H NMR (500 MHz, 75 °C, Benzene-d_6) δ 7.75 (d, *J* = 7.6 Hz, 1H), 7.15 (obscured t, *J* = 7.0 Hz, 1H), 7.02 (d, *J* = 7.6 Hz, 1H), 6.98 (t, *J* = 7.5 Hz, 1H), 6.27 (s, 1H), 5.62 (dq, *J* = 17.0, 7.8 Hz, 1H), 4.90 (d, *J* = 16.9 Hz, 1H), 4.76 (d, *J* = 10.2 Hz, 1H), 3.89 (d, *J* = 17.4 Hz, 1H), 3.17 (d, *J* = 17.3 Hz, 1H), 2.58 (dd, *J* = 14.0, 7.3 Hz, 1H), 2.44 (dd, *J* = 14.1, 6.8 Hz, 1H), 1.42 (s, 9H), 1.21 (s, 9H). ^{13}C NMR (126 MHz, 75 °C, Benzene) δ 202.03, 156.38, 154.46, 151.14, 136.57, 134.47, 132.71, 127.38, 126.17, 124.43, 118.59, 82.11, 80.86, 70.61, 41.29, 40.48, 28.43, 28.14. HRMS (EI) calculated for $C_{22}H_{30}N_2O_5$ (Na^+) 425.2052, found: 425.2050. R_f (petroleum ether / EtOAc 4:1) = 0.38. $[\alpha]_D^{25}$ = 17.5 (c = 1.16, $CHCl_3$). HPLC: (*Chiralpak® IA*, heptane-isopropanol 95:5, 0.8 mL/min, 254 nm): t_R(minor) = 7.6 min, t_R(major) = 22.2 min, 95% *ee*.

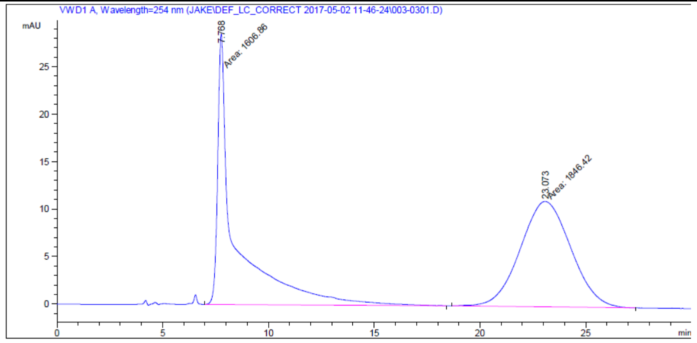

Peak #	RetTime [min]	Type	Width [min]	Area mAU *s	Height [mAU]	Area %
1	7.768	MM	0.9403	1606.86462	28.48058	46.5315
2	23.073	MM	2.7731	1846.41614	11.09702	53.4685

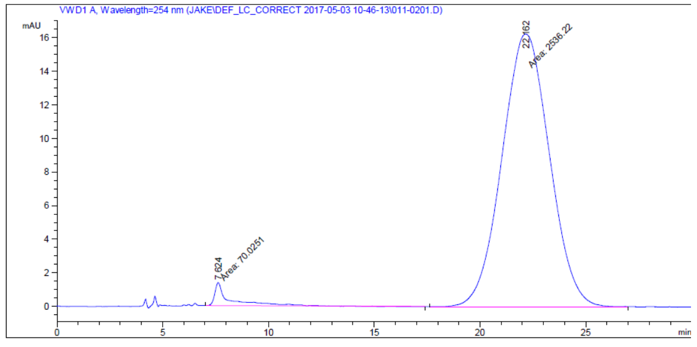

Peak #	RetTime [min]	Type	Width [min]	Area mAU *s	Height [mAU]	Area %
1	7.624	MM	0.8509	70.02509	1.37163	2.6868
2	22.162	MM	2.6016	2536.22046	16.24764	97.3132

di-tert-butyl 1-(2-propargyl-1-oxo-2,3-dihydro-1H-inden-2-yl)hydrazine-1,2-dicarboxylate **2e**

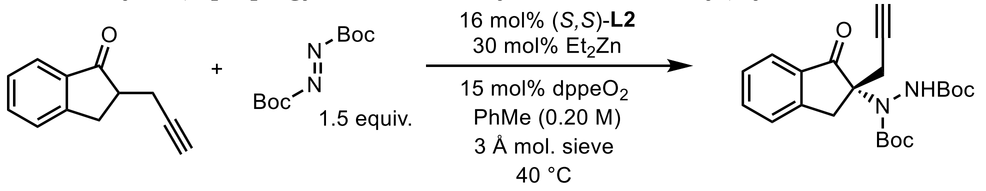

(*S*,*S*)-**L2** (24.8 mg, 0.0320 mmol), 2-propargyl-1-indanone (34.0 mg, 0.200 mmol), and activated 3 Å molecular sieves (5 mg) were measured into a microwave vial equipped with stir bar. The flask was placed under a nitrogen atmosphere and PhMe (0.6 mL) was added. After stirring for 15 minutes, diethylzinc (1.0 M in hexanes, 60μL, 0.060 mmol) was added and the reaction was allowed to stir for an additional 15 minutes. At this time, a premixed (15 min) PhMe (0.4 mL) solution of di-*tert*-butyl azodicarboxylate (69.1 mg, 0.300 mmol) and 3 Å molecular sieves (5 mg) was added. The reaction was allowed to stir at 40 °C for 16 h at which point the reaction mixture was filtered through a small plug of fluorosil. The residue was concentrated under reduced pressure using rotary evaporation. The resulting residue was purified via flash chromatography (SiO2; DCM to petroleum ether / EtOAc 6:1 to petroleum ether / EtOAc 4:1) to afford 75.0 mg (94 %) of a clear oil.

IR(C_6D_6) 3313, 2980, 2931, 1728, 1611, 1479, 1368, 1250, 1158, 757 cm^{-1}. ^{1}H NMR (500 MHz, 75 °C, Benzene-d_6) δ 7.76 (d, *J* = 7.7 Hz, 1H), 7.16 (obscured t, 1H), 7.03 (d, *J* = 7.7 Hz, 1H), 6.98 (t, *J* = 7.5 Hz, 1H), 6.19 (s, 1H), 3.89 (d, *J* = 17.6 Hz, 1H), 3.39 (d, *J* = 17.4 Hz, 1H), 2.70 – 2.48 (m, 2H), 1.53 – 1.46 (m, 1H), 1.41 (s, 9H), 1.20 (s, 9H). ^{13}C NMR (126 MHz, 75 °C, Benzene) δ 200.89, 156.25, 154.11, 151.53, 136.14, 134.54, 127.36, 126.10, 124.64, 82.30, 80.94, 79.16, 71.38, 69.67, 40.32, 28.40, 28.10, 26.76. HRMS (EI) calculated for $C_{22}H_{28}N_2O_5$ (Na^+) 423.1896, found: 423.1909. R_f (petroleum ether / EtOAc 4:1) = 0.22. $[\alpha]_D^{24}$ = 8.0 (c = 1.44, $CHCl_3$). HPLC: (*Chiralpak® IA*, heptane-isopropanol 95:5, 0.8 mL/min, 254 nm): t_R(minor) = 9.5 min, t_R(major) = 35.3 min, 95% *ee*.

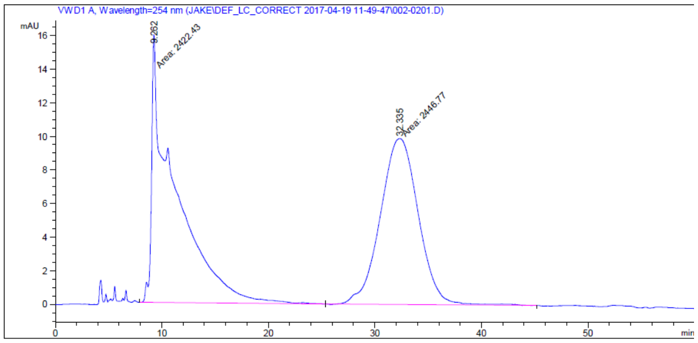

Peak #	RetTime [min]	Type	Width [min]	Area mAU *s	Height [mAU]	Area %
1	9.262	MM	2.5299	2422.43115	15.95845	49.7501
2	32.335	MM	4.1295	2446.76685	9.87508	50.2499

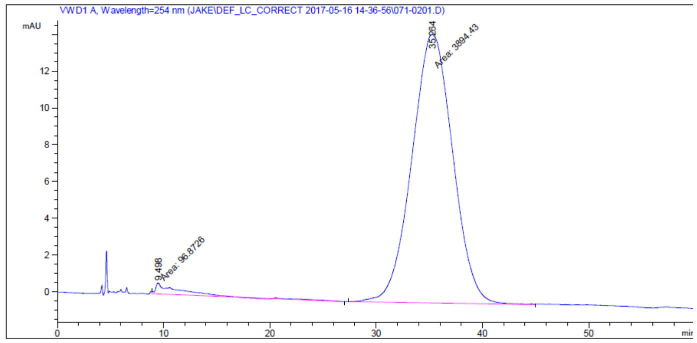

Peak #	RetTime [min]	Type	Width [min]	Area mAU *s	Height [mAU]	Area %
1	9.498	MM	2.6937	96.87257	5.99369e-1	2.4271
2	35.264	MM	4.4464	3894.43335	14.59783	97.5729

di-tert-butyl 1-(2-(2-methoxy-2-oxoethyl)-1-oxo-2,3-dihydro-1H-inden-2-yl)hydrazine-1,2-dicarboxylate **2f**

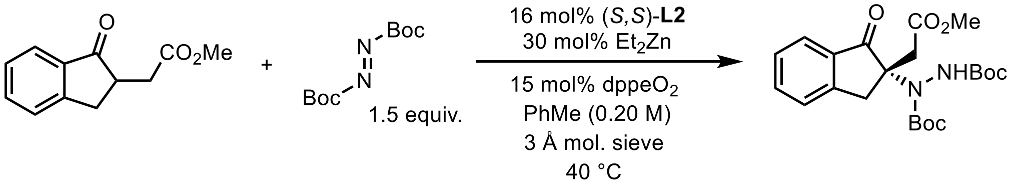

(*S*,*S*)-**L2** (24.8 mg, 0.0320 mmol), methyl 2-(1-oxo-2,3-dihydro-1H-inden-2-yl)acetate (40.8 mg, 0.200 mmol), and activated 3 Å molecular sieves (5 mg) were measured into a microwave vial equipped with stir bar. The flask was placed under a nitrogen atmosphere and PhMe (0.6 mL) was added. After stirring for 15 minutes, diethylzinc (1.0 M in hexanes, 60μL, 0.060 mmol) was added and the reaction was allowed to stir for an additional 15 minutes. At this time, a premixed (15 min) PhMe (0.4 mL) solution of di-*tert*-butyl azodicarboxylate (69.1 mg, 0.300 mmol) and 3 Å molecular sieves (5 mg) was added. The reaction was allowed to stir at 40 °C for 16 h at which point the reaction mixture was filtered through a small plug of fluorosil. The residue was concentrated under reduced pressure using rotary evaporation. The resulting residue was purified via flash chromatography (SiO2; DCM to petroleum ether / EtOAc 5:1 to petroleum ether / EtOAc 4:1 to petroleum ether / EtOAc 3:1) to afford 79.0 mg (91 %) of a clear oil.

IR(C_6D_6) 3310, 2979, 1730, 1611, 1467, 1437, 1368, 12248, 1171, 1063, 1013, 853, 776, 739 cm^{-1}. ^1H NMR (400 MHz, 75 °C, Benzene-d_6) spectrum complicated due to rotomers δ 7.76 (d, J = 7.6 Hz, 1H), 7.12 (d, J = 7.7 Hz, 1H), 7.08 – 6.98 (m, 1H), 6.95 (t, J = 7.4 Hz, 1H), 6.41 (2S, 1H total), 4.28 – 3.44 (broad m, 2H total), 3.25 (s, 3H), 2.57 (s, 2H), 1.32 (series of s, 18H total). ^{13}C NMR (101 MHz, 75 °C, Benzene-d_6) spectrum complicated due to rotomers δ 200.42, 169.80, 155.83, 154.19, 150.99, 135.39, 134.61, 127.40, 126.44, 124.81, 82.08, 80.90, 69.97, 51.24, 40.36, 39.79, 28.40, 28.11. R_f (petroleum ether / EtOAc 3:1) = 0.37. HRMS (EI) calculated for $C_{22}H_{30}N_2O_7$ (Na^+) 457.1951, found: 457.1939. $[\alpha]_D^{23}$ = 6.7 (c = 1.27, $CHCl_3$). HPLC**:** (*Chiralpak® IA*, heptane-isopropanol 90:10, 0.8 mL/min, 254 nm): t_R(minor) = 8.4 min, t_R(major) = 23.6 min, 94% *ee*.

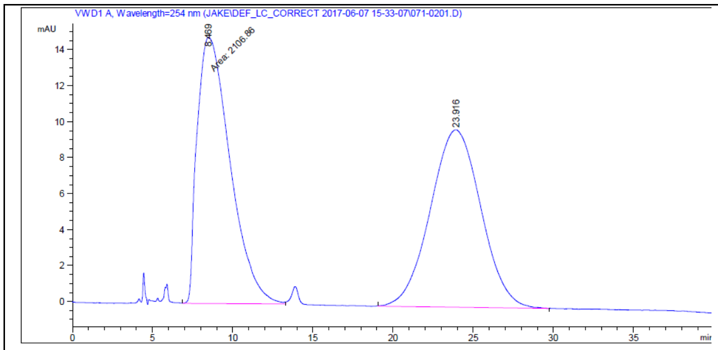

Peak #	RetTime [min]	Type	Width [min]	Area mAU *s	Height [mAU]	Area %
1	8.469	MM	2.3752	2106.86182	14.78362	49.2773
2	23.916	BB	2.7443	2168.66406	9.86859	50.7227

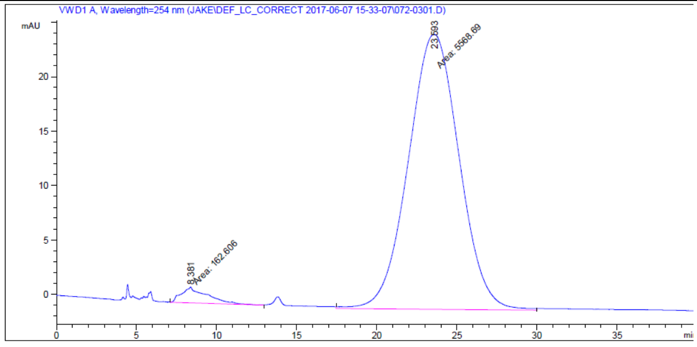

Peak #	RetTime [min]	Type	Width [min]	Area mAU *s	Height [mAU]	Area %
1	8.381	MM	1.8635	162.60594	1.45432	2.8372
2	23.593	MM	3.6750	5568.69141	25.25515	97.1628

di-tert-butyl (S)-1-(2-isopropyl-1-oxo-2,3-dihydro-1H-inden-2-yl)hydrazine-1,2-dicarboxylate **2g**

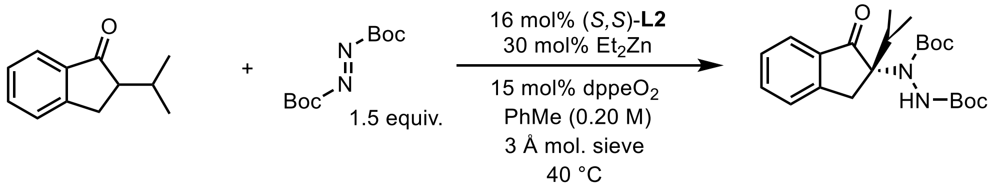

(*S*,*S*)-**L2** (24.8 mg, 0.0320 mmol), 2-isopropyl-1-indanone (34.8 mg, 0.200 mmol), and activated 3 Å molecular sieves (5 mg) were measured into a microwave vial equipped with a stir bar. The flask was placed under a nitrogen atmosphere and PhMe (0.6 mL) was added. After stirring for 15 minutes, diethylzinc (1.0 M in hexanes, 60μL, 0.060 mmol) was added and the reaction was allowed to stir for an additional 15 minutes. At this time, a premixed (15 min) PhMe (0.4 mL) solution of di-*tert*-butyl azodicarboxylate (69.1 mg, 0.300 mmol) and 3 Å molecular sieves (5 mg) was added. The reaction was allowed to stir at 40 °C for 17 h at which point the reaction mixture was filtered through a small plug of fluorosil. The residue was concentrated under reduced pressure using rotary evaporation. The resulting residue was purified via flash chromatography (SiO2; petroleum ether / EtOAc 7:1) to afford 31.2 mg (39 %) of a clear oil.

IR(C_6D_6) 3323, 2978, 2935, 1724, 1611, 1469, 1393, 1368, 1249, 1157, 1053, 1020, 997, 867, 771, 735 cm^{-1}. ^{1}H NMR (500 MHz, 75 °C, Benzene-d_6) δ 7.82 – 7.65 (m, 1H), 7.29 – 7.18 (m, 1H), 7.10 – 6.90 (m, 2H), 6.31 (s, 1H), 3.93 (d, J = 17.7 Hz, 1H), 3.22 – 3.05 (m, 1H), 2.43 – 2.20 (m, 1H), 1.49 – 1.39 (m, 9H), 1.27 – 1.12 (m, 9H), 0.98 – 0.89 (m, 3H), 0.69 (dd, J = 12.4, 6.9 Hz, 3H). ^{13}C NMR (126 MHz, 75 °C, Benzene) δ 201.62, 156.65, 155.22, 151.85, 137.89, 134.25, 127.22, 125.64, 123.92, 82.17, 80.97, 73.70, 40.03, 33.90, 28.46, 28.15, 18.08, 17.75. HRMS (EI) calculated for $C_{22}H_{32}N_2O_5$ (Na^+) 427.2203, found: 427.2203. $[\alpha]_D^{24}$ = 25.5 (c = 1.53, $CHCl_3$). R_f (petroleum ether / EtOAc 7:1) = 0.26. HPLC: (*Chiralpak® IB*, heptane-isopropanol 99:1, 0.8 mL/min, 254 nm): t_R(minor) = 12.2 min, t_R(major) = 9.7 min, 88% *ee*.

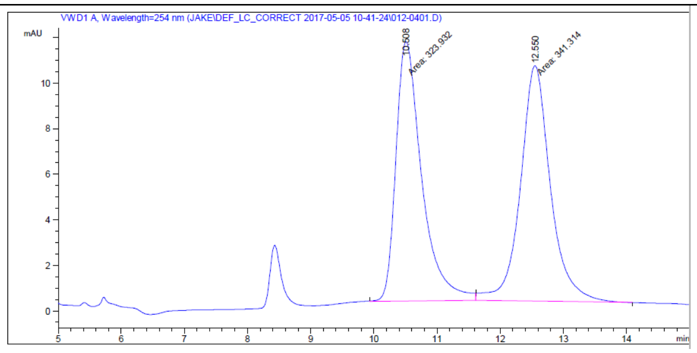

Peak #	RetTime [min]	Type	Width [min]	Area mAU *s	Height [mAU]	Area %
1	10.508	MM	0.4740	323.93179	11.39102	48.6935
2	12.550	MM	0.5504	341.31430	10.33487	51.3065

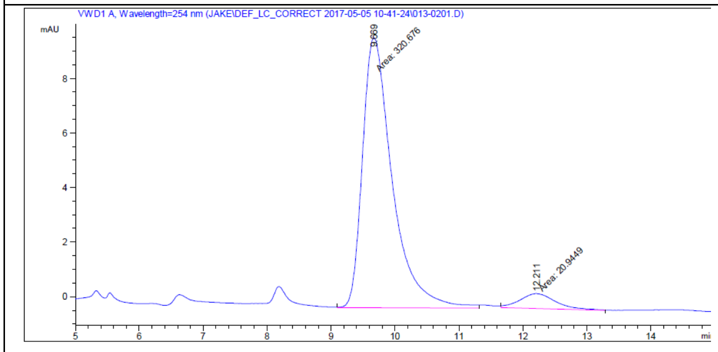

Peak #	RetTime [min]	Type	Width [min]	Area mAU *s	Height [mAU]	Area %
1	9.669	MM	0.5400	320.67563	9.89743	93.8690
2	12.211	MM	0.6415	20.94489	5.44143e-1	6.1310

di-tert-butyl 1-(3-methyl-4-oxochroman-3-yl)hydrazine-1,2-dicarboxylate **2h**

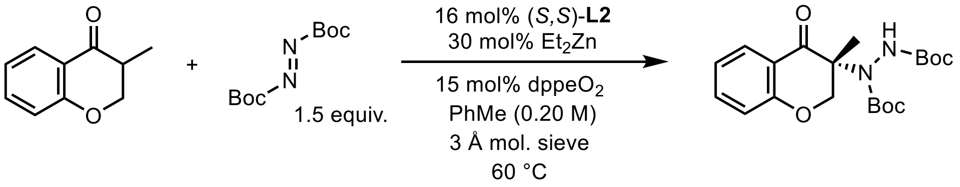

(*S*,*S*)-**L2** (24.8 mg, 0.0320 mmol), 2-methyl-1-chromanone (32.4 mg, 0.200 mmol), and activated 3 Å molecular sieves (5 mg) were measured into a microwave vial equipped with stir bar. The flask was placed under a nitrogen atmosphere and PhMe (0.6 mL) was added. After stirring for 15 minutes, diethylzinc (1.0 M in hexanes, 60μL, 0.060 mmol) was added and the reaction was allowed to stir for an additional 15 minutes. At this time, a premixed (15 min) PhMe (0.4 mL) solution of di-*tert*-butyl azodicarboxylate (69.1 mg, 0.300 mmol) and 3 Å molecular sieves (5 mg) was added. The reaction was allowed to stir at 60 °C for 16 h at which point the reaction mixture was filtered through a small plug of fluorosil. The residue was concentrated under reduced pressure using rotary evaporation. The resulting residue was purified via flash chromatography (SiO2; DCM to petroleum ether / EtOAc 7:1 to petroleum ether / EtOAc 6:1) to afford 47.8 mg (61 %) of a clear oil.

IR(C_6D_6) 3326, 2982, 2933, 1703, 1608, 1480, 1368, 1281, 1250, 1152, 1016, 761 cm^{-1}. ^1H NMR (400 MHz, 75 °C, Benzene-d_6) δ 8.10 (d, *J* = 7.8 Hz, 1H), 7.10 – 6.98 (m, 1H), 6.81 (d, *J* = 8.3 Hz, 1H), 6.76 – 6.65 (m, 1H), 6.13 (s, 1H), 5.23 (d, *J* = 11.1 Hz, 1H), 4.47 (s, 1H), 1.37 (s, 3H), 1.34 (s, 9H), 1.31 (s, 9H). ^{13}C NMR (101 MHz, 75 °C, Benzene-d_6) δ 191.10, 161.19, 156.37, 154.14, 135.40, 128.59, 121.89, 121.16, 118.14, 82.16, 80.89, 73.53, 65.45, 28.27, 28.22, 19.14. HRMS (EI) calculated for $C_{20}H_{28}N_2O_6$ (Na^+) 415.1840, found: 415.1837. R_f (petroleum ether / EtOAc 6:1) = 0.24. $[\alpha]_D^{24}$ = 5.0 (c = 1.56, $CHCl_3$). HPLC: (*Chiralpak® IA*, heptane-isopropanol 90:10, 0.8 mL/min, 254 nm): t_R(minor) = 5.5 min, t_R(major) = 9.0 min, 92% *ee*.

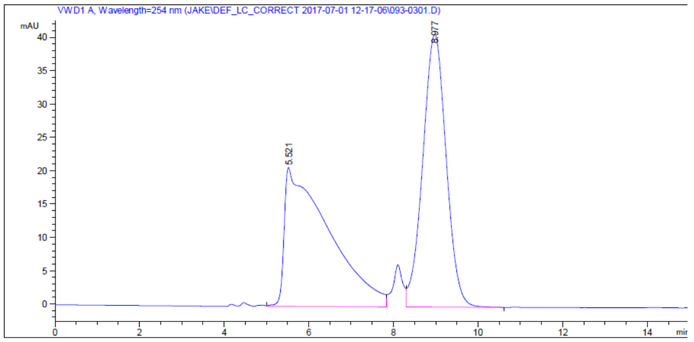

Peak #	RetTime [min]	Type	Width [min]	Area mAU *s	Height [mAU]	Area %
1	5.521	VV	0.9002	1528.91968	20.82472	49.3435
2	8.977	VB	0.5932	1569.60181	40.90887	50.6565

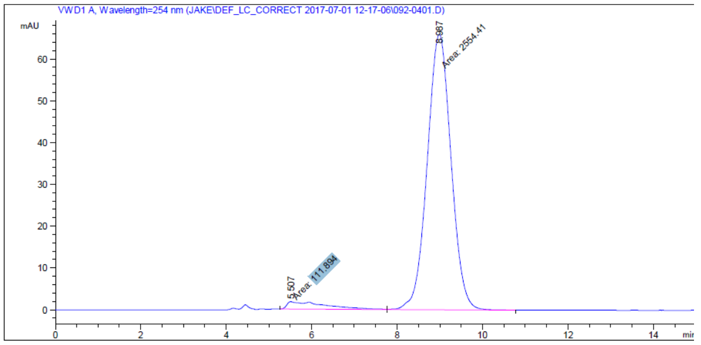

Peak #	RetTime [min]	Type	Width [min]	Area mAU *s	Height [mAU]	Area %
1	5.507	MM	1.0398	111.89423	1.79346	4.1966
2	8.987	MM	0.6459	2554.41211	65.91761	95.8034

di-tert-butyl (E)-1-(3-benzylidene-1-methyl-2-oxocyclohexyl)hydrazine-1,2-dicarboxylate **2i**

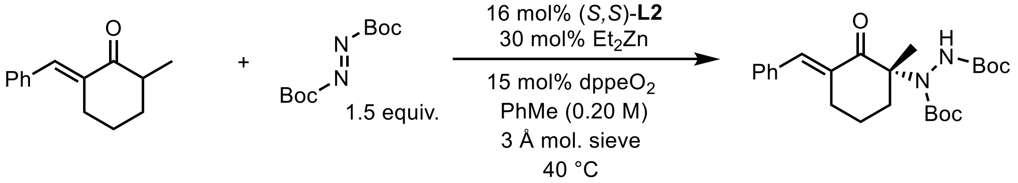

(*S*,*S*)-**L2** (24.8 mg, 0.0320 mmol), (E)-2-benzylidene-6-methylcyclohexan-1-one (40.0 mg, 0.200 mmol), and activated 3 Å molecular sieves (5 mg) were measured into a microwave vial equipped with stir bar. The flask was placed under a nitrogen atmosphere and PhMe (0.6 mL) was added. After stirring for 15 minutes, diethylzinc (1.0 M in hexanes, 60µL, 0.060 mmol) was added and the reaction was allowed to stir for an additional 15 minutes. At this time, a premixed (15 min) PhMe (0.4 mL) solution of di-*tert*-butyl azodicarboxylate (69.1 mg, 0.300 mmol) and 3 Å molecular sieves (5 mg) was added. The reaction was allowed to stir at 40 °C for 16 h at which point the reaction mixture was filtered through a small plug of fluorosil. The residue was concentrated under reduced pressure using rotary evaporation. The resulting residue was purified via flash chromatography (SiO2; DCM to petroleum ether / EtOAc 7:1 to petroleum ether / EtOAc 6:1) to afford 62.9 mg (73 %) of a clear oil.

IR(C_6D_6) 3339, 2980, 2938, 1698, 1598, 1450, 1367, 1246, 1157, 1121, 1079, 1121, 1079, 1010 cm^{-1}. ^{1}H NMR (500 MHz, 75 °C, Benzene-d_6) δ 7.77 (s, 1H), 7.20 – 7.13 (obscured m, 2H), 7.09 (t, J = 8.1, 3H), 7.07 – 6.98 (m, 1H), 6.18 (s, 1H), 2.85 (d, J = 14.5 Hz, 2H), 2.63 (m, 1H), 2.05 (s, 1H), 1.75 – 1.59 (m, 1H), 1.42 (2 S, 18H total), 1.39 – 1.37 (m, 1H), 1.33 – 1.26 (broad s, 3H). ^{13}C NMR (126 MHz, 75 °C, Benzene) δ 199.22, 156.17, 154.74, 137.10, 136.91, 136.23, 130.40, 128.46, 128.46 (identified via HSQC), 81.49, 80.39, 68.39, 36.94, 28.61, 28.48, 28.44, 23.38, 21.70. HRMS (EI) calculated for $C_{24}H_{34}N_2O_5$ (Na^+) 453.2360, found: 453.2357. $[\alpha]_D^{23}$ = 161.4 (c = 1.57, $CHCl_3$). HPLC: (*Chiralpak® IA*, heptane-isopropanol 90:10, 0.8 mL/min, 254 nm): t_R(minor) = 6.5 min, t_R(major) = 9.6 min, 91% *ee*.

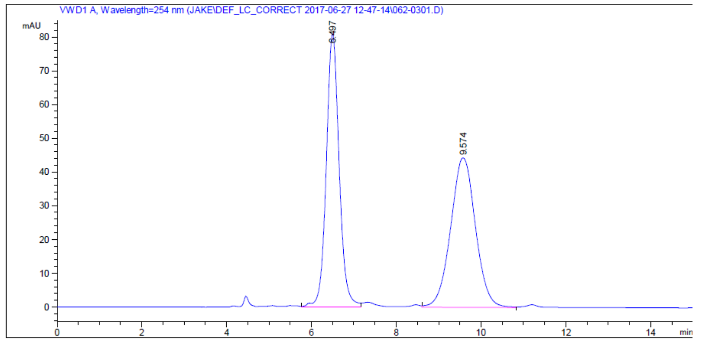

Peak #	RetTime [min]	Type	Width [min]	Area mAU *s	Height [mAU]	Area %
1	6.497	VV	0.3255	1713.36206	80.95639	48.7678
2	9.574	VV	0.6196	1799.94238	44.30431	51.2322

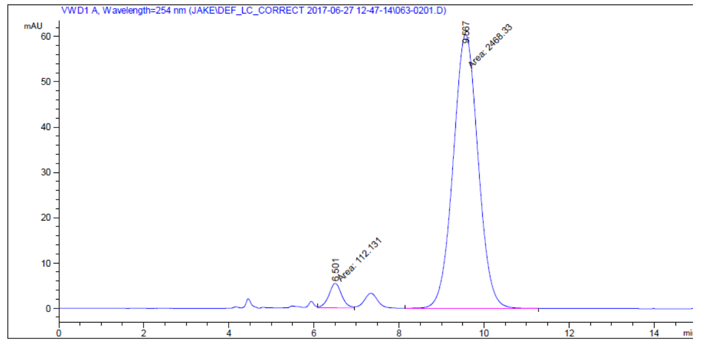

Peak #	RetTime [min]	Type	Width [min]	Area mAU *s	Height [mAU]	Area %
1	6.501	MM	0.3465	112.13118	5.39282	4.3454
2	9.567	MM	0.6797	2468.32568	60.52916	95.6546

di-tert-butyl (R)-1-(1-((benzyloxy)carbonyl)-3-methyl-2-oxoindolin-3-yl)hydrazine-1,2-dicarboxylate **2j**

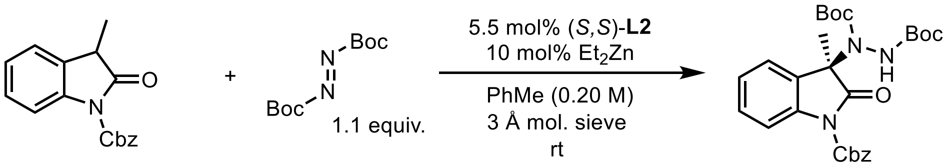

(*S*,*S*)-**L2** (8.6 mg, 0.0110 mmol), N-Cbz-3-methyl-2-oxinodle (56.3 mg, 0.200 mmol), and activated 3 Å molecular sieves (5 mg) were measured into a microwave vial equipped with a stir bar. The flask was placed under a nitrogen atmosphere and PhMe (0.6 mL) was added. After stirring for 15 minutes, diethylzinc (1.0 M in hexanes, 20μL, 0.020 mmol) was added and the reaction was allowed to stir for an additional 15 minutes. At this time, a premixed (15 min) PhMe (0.4 mL) solution of di-*tert*-butyl azodicarboxylate (50.7 mg, 0.220 mmol) and 3 Å molecular sieves (5 mg) was added. The reaction was allowed to stir at rt for 15 min at which point the reaction mixture was filtered through a small plug of fluorosil. The residue was concentrated under reduced pressure using rotary evaporation. The resulting residue was purified via flash chromatography (SiO2; petroleum ether / EtOAc 6:1) to afford 97.0 mg (95 %) of a clear oil.

IR(C_6D_6) 3323, 2981, 2933, 1783, 1730, 1609, 1481, 1456, 1370, 1345, 1290, 1229, 1154, 1107, 1074, 1051, 1017, 772, 755, 698 cm^{-1}. ^{1}H NMR (400 MHz, 75 °C, Benzene-d_6) δ 8.20 (s, 1H), 8.15 (d, *J* = 8.3 Hz, 1H), 7.40 (dd, *J* = 7.8, 4.1 Hz, 2H), 7.16 – 7.03 (m, 4H), 7.02 – 6.94 (m, 1H), 6.58 (s, 1H), 5.23 (s, 2H), 1.41 (s, 9H), 1.36 (s, 3H), 1.08 (s, 9H). ^{13}C NMR (101 MHz, 75 °C, c_6d6) δ 175.59, 156.66, 153.24, 151.54, 138.83, 136.02, 133.17, 128.80, 128.73, 128.40, 125.18, 124.27, 115.21, 82.57, 80.97, 68.50, 66.31, 28.35, 27.88, 24.28. HRMS (EI) calculated for $C_{27}H_{33}N_3O_7$ (H^+) 512.2391, found: 512.2393. $[\alpha]_D^{24}$ = -14.0 (c = 1.41, $CHCl_3$). R_f (petroleum ether / EtOAc 6:1) = 0.22. HPLC: (*Chiralpak® IA*, heptane-isopropanol 90:10, 0.8 mL/min, 254 nm): t_R(minor) = 7.9 min, t_R(major) = 35.2 min, 95% *ee*.

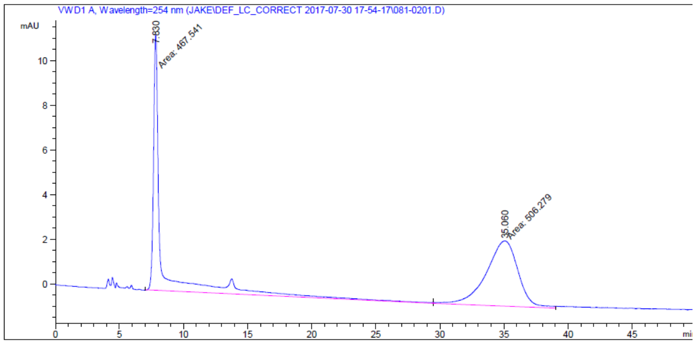

Peak #	RetTime [min]	Type	Width [min]	Area mAU *s	Height [mAU]	Area %
1	7.830	MM	0.6799	467.54071	11.46052	48.0110
2	35.060	MM	2.8831	506.27872	2.92670	51.9890

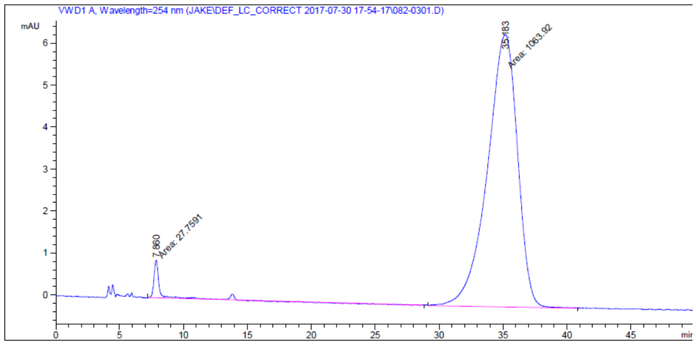

Peak #	RetTime [min]	Type	Width [min]	Area mAU *s	Height [mAU]	Area %
1	7.860	MM	0.5169	27.75909	8.95072e-1	2.5428
2	35.183	MM	2.7331	1063.91675	6.48775	97.4572

Large-scale procedure:

S,S)-**L2** (35.8 mg, 0.0462 mmol), N-Cbz-3-methyl-2-oxinodle (591 mg, 2.11 mmol), and activated 3 Å molecular sieves (55 mg) were measured into a round-bottom flask equipped with a stir bar. The flask was placed under a nitrogen atmosphere and PhMe (6.3 mL) was added. After stirring for 15 minutes, diethylzinc (1.0 M in hexanes, 0.84 mL, 0.84 mmol) was added and the reaction was allowed to stir for an additional 15 minutes. At this time, a premixed (15 min) PhMe (4.2 mL) solution of di-*tert*-butyl azodicarboxylate (531.9 mg, 2.31 mmol) and 3 Å molecular sieves (55 mg) was added. The reaction was allowed to stir at rt for 4 h at which point the reaction mixture was filtered through a small plug of fluorosil. The residue was concentrated under reduced pressure using rotary evaporation. The resulting residue was purified via flash chromatography (SiO2; petroleum ether / EtOAc 6:1) to afford 950 mg (89 %) of a white foam.

HPLC: (*Chiralpak® IA*, heptane-isopropanol 90:10, 0.8 mL/min, 254 nm): t_R(minor) = 7.8 min, t_R(major) = 35.3 min, 92% *ee*.

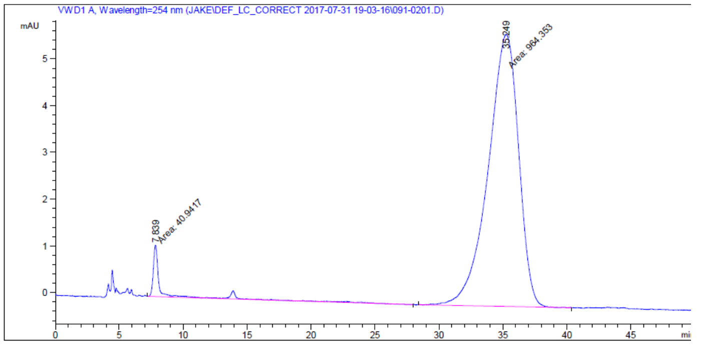

Peak #	RetTime [min]	Type	Width [min]	Area mAU *s	Height [mAU]	Area %
1	7.839	MM	0.6223	40.94165	1.09650	4.0726
2	35.249	MM	2.7621	964.35272	5.81897	95.9274

di-tert-butyl (R)-1-(1-((benzyloxy)carbonyl)-3-(2-((tert-butyldimethylsilyl)oxy)ethyl)-2-oxoindolin-3-yl)hydrazine-1,2-dicarboxylate **2k**

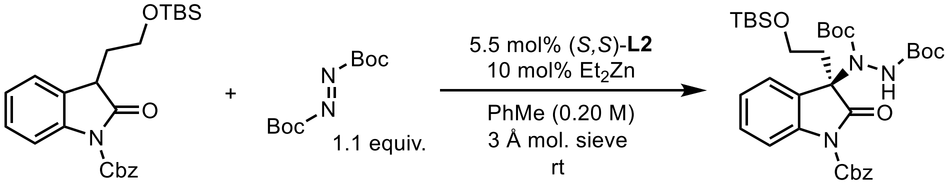

(*S,S*)-**L2** (8.6 mg, 0.0110 mmol), 3-alkyl-2-oxinodle (85.1 mg, 0.200 mmol), and activated 3 Å molecular sieves (5 mg) were measured into a microwave vial equipped with a stir bar. The flask was placed under a nitrogen atmosphere and PhMe (0.6 mL) was added. After stirring for 15 minutes, diethylzinc (1.0 M in hexanes, 20μL, 0.020 mmol) was added and the reaction was allowed to stir for an additional 15 minutes. At this time, a premixed (15 min) PhMe (0.4 mL) solution of di-*tert*-butyl azodicarboxylate (50.7 mg, 0.220 mmol) and 3 Å molecular sieves (5 mg) was added. The reaction was allowed to stir at rt for 1 h at which point the reaction mixture was filtered through a small plug of fluorosil. The residue was concentrated under reduced pressure using rotary evaporation. The resulting residue was purified via flash chromatography (SiO2; petroleum ether / EtOAc 7:1) to afford 113.3 mg (86 %) of a clear oil.

IR(C_6D_6) 3328, 2957 2931, 2885, 2857, 1786, 1727, 1481, 1468, 1369, 1348, 1291, 1251, 1223, 1164, 1112, 1075, 1045, 1022, 837, 772, 756, 698 cm^{-1}. ^{1}H NMR (500 MHz, 75 °C, Benzene-d_6) δ 8.15 (dt, *J* = 8.1, 0.8 Hz, 1H), 8.06 (s, 1H), 7.41 (dm, *J* = 7.5 Hz, 2H), 7.15 – 7.11 (m, 2H), 7.12 – 7.02 (m, 2H), 6.98 (td, *J* = 7.5, 1.1 Hz, 1H), 6.32 (s, 1H), 5.29 (d, *J* = 12.6 Hz, 1H), 5.20 (d, *J* = 12.6 Hz, 1H), 3.45 – 3.34 (m, 2H), 2.49 (dt, *J* = 13.4, 7.5 Hz, 1H), 2.19 (dt, *J* = 13.4, 5.3 Hz, 1H), 1.42 (s, 9H), 1.09 (s, 9H), 0.83 (s,

9H), -0.09 (s, 3H), -0.11 (s, 3H). ^{13}C NMR (126 MHz, 75 °C, Benzene) δ 174.55, 156.69, 153.42, 151.54, 140.29, 136.15, 130.92, 129.05, 128.69, 128.36, 128.31, 124.95, 124.74, 115.16, 82.70, 81.11, 68.36, 68.13, 58.89, 40.76, 28.39, 27.92, 26.14, 18.48, -5.42, -5.43. HRMS (EI) calculated for $C_{34}H_{49}N_3O_8Si$ (Na^+) 678.3181, found: 678.3179. $[\alpha]_D^{24}$ = -11.3 (c = 1.61, $CHCl_3$). R_f (petroleum ether / EtOAc 7:1) = 0.22. HPLC: (*Chiralpak® IA*, heptane-isopropanol 97:3, 0.8 mL/min, 254 nm): t_R(minor) = 49.4 min, t_R(major) = 66.0 min, 95% *ee*.

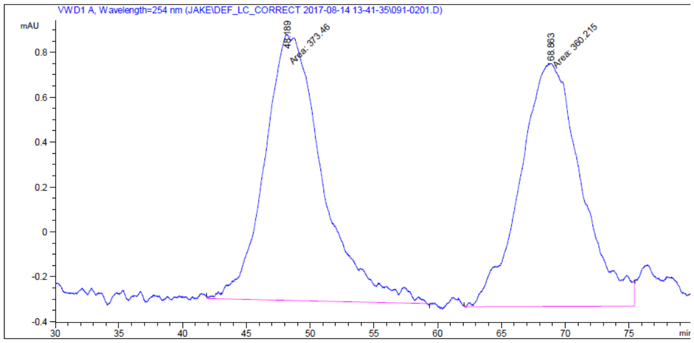

Peak #	RetTime [min]	Type	Width [min]	Area mAU *s	Height [mAU]	Area %
1	48.189	MM	5.2494	373.46017	1.18573	50.9026
2	68.863	MM	5.5414	360.21545	1.08340	49.0974

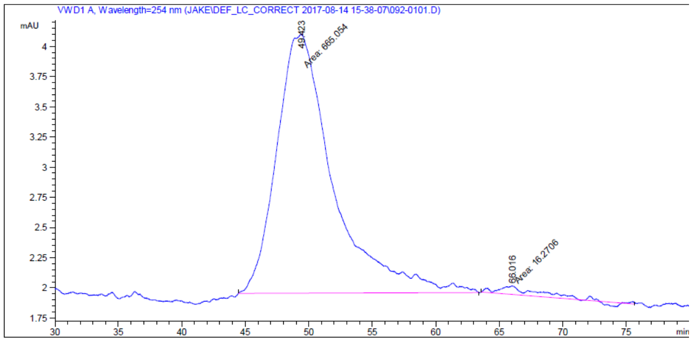

Peak #	RetTime [min]	Type	Width [min]	Area mAU *s	Height [mAU]	Area %
1	49.423	MM	5.1623	665.05389	2.14714	97.6119
2	66.016	MM	3.8427	16.27064	7.05702e-2	2.3881

<u>di-tert-butyl (R)-1-(1-(tert-butoxycarbonyl)-3-methyl-2-oxoindolin-3-yl)hydrazine-1,2-dicarboxylate</u> **SI**

For determination of absolute stereochemistry of oxindole products:

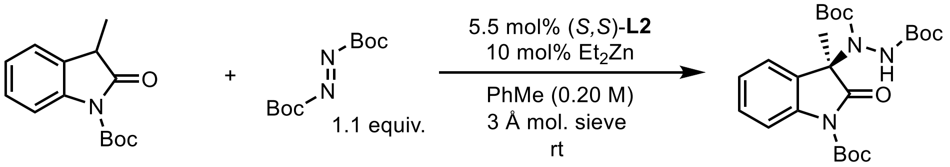

(*S*,*S*)-**L2** (8.3 mg, 0.011 mmol), N-Boc-3-methyl-2-oxinodle (22 mg, 0.089 mmol), and activated 3 Å molecular sieves (5 mg) were measured into a microwave vial equipped with a stir bar. The flask was placed under a nitrogen atmosphere and PhMe (0.3 mL) was added. After stirring for 15 minutes, diethylzinc (1.0 M in hexanes, 20μL, 0.020 mmol) was added and the reaction was allowed to stir for an additional 15 minutes. At this time, a premixed (15 min) PhMe (0.2 mL) solution of di-*tert*-butyl azodicarboxylate (69 mg, 0.30 mmol) and 3 Å molecular sieves (5 mg) was added. The reaction was allowed to stir at rt for 15 min at which point the reaction mixture was filtered through a small plug of fluorosil. The residue was concentrated under reduced pressure using rotary evaporation. The resulting residue was purified via flash chromatography (SiO2; petroleum ether / EtOAc 8:1) to afford 32.6 mg (77 %) of a white solid.

^1H NMR (400 MHz, Chloroform-*d*) δ 7.89 (d, *J* = 7.4 Hz, 1H), 7.79 (d, *J* = 8.1 Hz, 1H), 7.33 – 7.27 (m, 1H), 7.19 (t, *J* = 7.5 Hz, 1H), 6.59 (s, 1H), 1.64 (s, 9H), 1.53 (s, 9H), 1.52 (s, 3H), 1.13 (s, 9H). ^{13}C NMR

(101 MHz, $CDCl_3$) δ 176.38, 156.37, 152.98, 149.38, 138.07, 128.59, 125.13, 123.82, 114.79, 84.48, 83.13, 81.68, 65.92, 28.35, 28.24, 27.79, 24.53. $[\alpha]_D^{23}$ = -11.0 (c = 1.75, $CHCl_3$). HPLC: (*Chiralpak® IA*, heptane-isopropanol 90:10, 0.8 mL/min, 254 nm): t_R(minor) = 5.3 min, t_R(major) = 11.4 min, 70% *ee*.

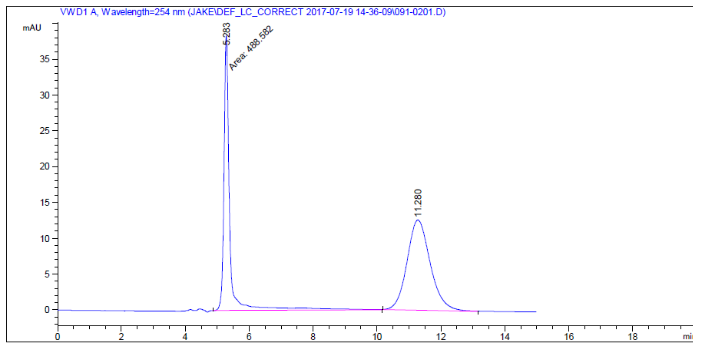

Peak #	RetTime [min]	Type	Width [min]	Area mAU *s	Height [mAU]	Area %
1	5.283	MM	0.2122	488.58218	38.37923	43.5008
2	11.280	BB	0.7681	634.57471	12.60023	56.4992

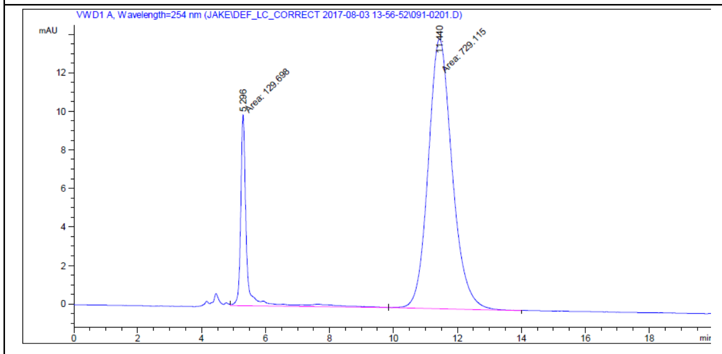

Peak #	RetTime [min]	Type	Width [min]	Area mAU *s	Height [mAU]	Area %
1	5.296	MM	0.2180	129.69756	9.91524	15.1020
2	11.440	MM	0.8680	729.11548	13.99969	84.8980

For the same compound, Shibasaki reports $[\alpha]_D^{22.7}$ = -21.0 (*c* = 1.34, $CHCl_3$) for a compound with 99% *ee*. As the same sign was obtained, the absolute chirality was assigned as that reported by Shibasaki. The N-Cbz substrates were assigned by analogy.[2]

<u>methyl (R)-2-(3-((tert-butoxycarbonyl)amino)-3-methyl-2-oxoindolin-1-yl)acetate</u> **6**

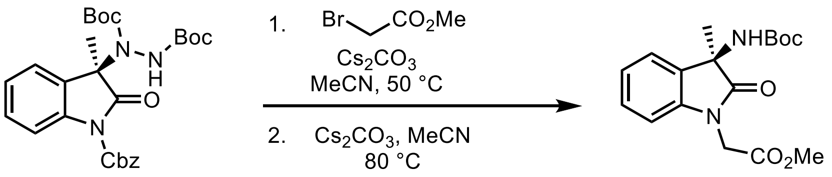

A vial containing a magnetic stir bar was charged with the hydrazine oxindole (76.7 mg, 0.150 mmol), placed under a nitrogen atmosphere, and brought into the glovebox. In the glovebox, freshly ground Cs_2CO_3 (147 mg, 0.451 mmol) was added to the vial. The vial was sealed and removed from the glovebox and dry acetonitrile (1.0 mL) was added followed by methyl bromoacetate (35.5 μL, 0.375 mmol). The reaction was allowed to stir in a 50 °C oil bath for 15 h at which point the reaction mixture was filtered through a small plug of celite using ethyl acetate as the eluting solvent. The filtrate was concentrated under reduced pressure using rotary evaporation and the resulting residue was purified via flash chromatography (SiO2; petroleum ether / EtOAc 4:1 to 3:1). R_f (petroleum ether / EtOAc 3:1) = 0.29.

The purified material from above was utilized directly in the next step without full characterization due to the presence of significant rotomers. A vial containing a magnetic stir bar was charged with the purified alkylation product, placed under a nitrogen atmosphere, and brought into the glovebox. In the glovebox, freshly ground Cs_2CO_3 (147 mg, 0.451 mmol) was added to the vial. The vial was removed from the glovebox and dry acetonitrile (1.0 mL) was added. The reaction was allowed to stir in an 80 °C oil bath for 20 h. At this point, the reaction was quenched with a saturated aqueous solution of ammonium chloride (15 ml) and diluted with 2 mL H_2O and ethyl acetate (25 mL). The organic layer was separated and the aqueous layer was extracted with ethyl acetate (2 x 25 mL). The combined organic layers were washed with brine (20 mL), dried (MgSO4), filtered, and concentrated under reduced pressure using rotary evaporation. The resulting residue was purified via flash chromatography (SiO2; petroleum ether / EtOAc 2:1 to 1.5:1) to afford 34 mg (68% over 2 steps) of a white solid (mp 169-170 °C).

IR($CDCl_3$) 3355, 3332, 2982, 2918, 1728, 1616, 1493, 1469, 1367, 1217, 1168, 1065, 754, 665 cm^{-1}. ^1H NMR (500 MHz, 55 °C, Chloroform-*d*) δ 7.30 (dd, *J* = 7.4, 1.3 Hz, 1H), 7.25 (td, *J* = 7.7, 1.4 Hz, 1H), 7.07 (td, *J* = 7.5, 1.0 Hz, 1H), 6.72 (d, *J* = 7.8 Hz, 1H), 5.06 (s, 1H), 4.83 (d, *J* = 17.4 Hz, 1H), 4.14 (d, *J* = 17.4 Hz, 1H), 3.76 (s, 3H), 1.54 (s, 3H), 1.26 (s, 9H). ^{13}C NMR (126 MHz, 55 °C, $CDCl_3$) δ 177.35, 168.32, 153.92, 141.80, 132.43, 128.77, 123.24, 122.46, 108.37, 80.61, 58.71, 52.50, 41.75, 28.30, 25.09. HRMS (EI) calculated for $C_{17}H_{22}N_2O_5$ (Na^+) 357.1421, found: 357.1421. $[\alpha]_D^{23}$ = 23.8 (c = 0.68, $CHCl_3$).

di-tert-butyl 1-((5S,6S)-5-hydroxy-6,7,8,9-tetrahydro-5H-benzo[7]annulen-6-yl)hydrazine-1,2-dicarboxylate **SII**

The procedure used was based upon the work of Gmeiner and Bollinger, as recently used by Ma.[3,4]

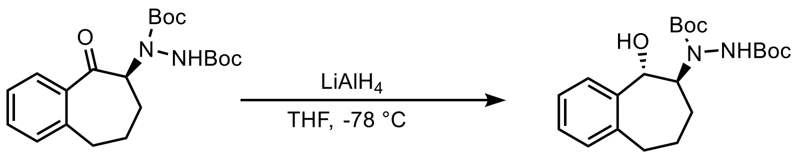

A round-bottom flask equipped with a magnetic stir bar was charged with the ketone (145.2 mg, 0.372 mmol) and placed under an atmosphere of nitrogen. THF (10 mL) was added and the reaction was cooled to -78 °C. $LiAlH_4$ (1.0M in Et_2O, 0.37 mL, 0.37 mmol) was added dropwise and the reaction was allowed to stir at -78 °C for 1.5 h. At this time, the reaction mixture was quenched via addition to 15 mL of 10% aqueous ammonium chloride solution. The reaction mixture was diluted with DCM (25 mL) and the organic layer was separated. The aqueous layer was extracted with DCM (2 x 25 mL). The organic layers were combined, washed with brine (15 mL), dried (Na_2SO_4), filtered, and concentrated under reduced pressure via rotary evaporation. The resulting residue was purified via careful flash chromatography (SiO2; petroleum ether / EtOAc 7:1) to afford 138 mg (94%) of a clear oil.

IR(CH_2Cl_2) 3394, 3302, 2979, 2932, 2857, 1712, 1482, 1456, 1393, 1368, 1326, 1290, 1254, 1157, 1110, 1061, 942, 857, 751 cm^{-1}. ^1H NMR (400 MHz, 75 °C, Benzene-d_6) δ 8.21 (s, 1H), 7.22 (t, *J* = 7.7 Hz, 1H), 7.07 (td, *J* = 7.4, 1.4 Hz, 1H), 6.92 (dd, *J* = 7.3, 1.4 Hz, 1H), 5.95 (s, 1H), 4.80 (d, *J* = 9.3 Hz, 1H), 4.10 (s, 1H), 2.49 – 2.35 (m, 2H), 1.79 – 1.53 (m, 3H), 1.43 (s, 9H), 1.40 (s, 9H), 1.26 – 1.10 (m, 1H). ^{13}C NMR (101 MHz, 75 °C, c_6d_6) δ 158.61 (broad), 155.43, 142.13, 140.10, 128.59, 127.07, 126.96, 126.05, 82.14, 81.44, 72.14, 63.07 (broad), 35.27, 33.48, 28.37, 28.30, 26.94. HRMS (EI) calculated for $C_{21}H_{32}N_2O_5$ (Na^+) 415.2203, found: 415.2202. $[\alpha]_D^{22}$ = 89.4 (c = 1.47, $CHCl_3$). R_f (petroleum ether /

EtOAc 7:1) = 0.23. HPLC: (*Chiralpak® IC*, heptane-isopropanol 95:5, 0.8 mL/min, 220 nm): t_R(minor) = 10.8 min, t_R(major) = 17.3 min, 98% *ee*.

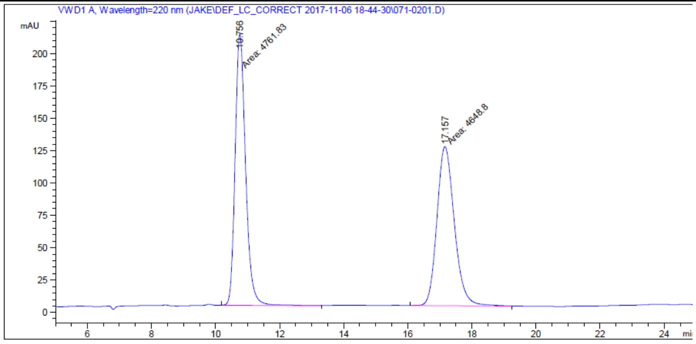

Peak #	RetTime [min]	Type	Width [min]	Area mAU *s	Height [mAU]	Area %
1	10.756	MM	0.3788	4761.82568	209.49161	50.6005
2	17.157	MM	0.6304	4648.79639	122.91516	49.3995

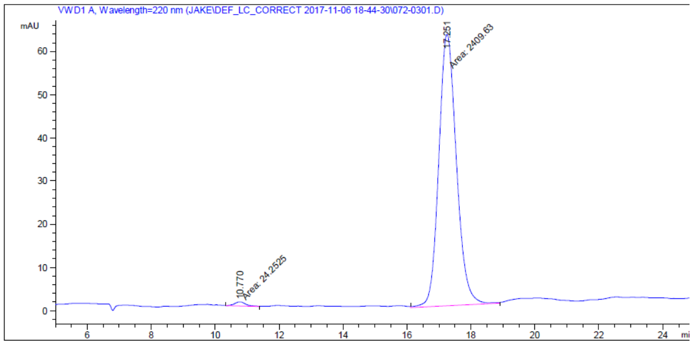

Peak #	RetTime [min]	Type	Width [min]	Area mAU *s	Height [mAU]	Area %
1	10.770	MM	0.4134	24.25246	9.77825e-1	0.9965
2	17.251	MM	0.6392	2409.63281	62.82560	99.0035

<u>di-tert-butyl 1-((5S,6S)-5-((tert-butyldimethylsilyl)oxy)-6,7,8,9-tetrahydro-5H-benzo[7]annulen-6-yl)hydrazine-1,2-dicarboxylate</u> **SIII**

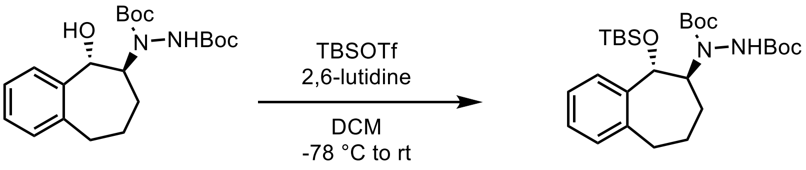

A round-bottom flask equipped with a magnetic stir bar was charged with the alcohol (127 mg, 0.324 mmol) and placed under an atmosphere of nitrogen. DCM (3.3 mL) was added followed by 2,6-lutidine (0.15 mL, 1.3 mmol). The reaction flask was cooled to -78 °C and *tert*-butyldimethylsilyl trifluoromethanesulfonate (TBSOTf) (84 µL, 0.36 mmol) was added dropwise. After stirring for 1 h at -78 °C, the dry ice acetone bath was removed and the reaction was stirred at rt for 1 h. Incomplete reactivity was observed, so the reaction mixture was cooled once again to -78 °C and additional TBSOTf (84 µL, 0.36 mmol) was added dropwise. After stirring for 20 min at -78 °C, the cold bath was removed and the reaction was allowed to stir at rt for 1.5 h. Once again, incomplete conversion was observed, and the reaction mixture was cooled to -78 °C. Additional TBSOTf (20 µL, 0.086 mmol) was added dropwise. The reaction was allowed to stir at -78 °C for 20 minutes at which point the cold bath was once again removed and the reaction was allowed to stir 1 h at rt. At this point, saturated aqueous sodium bicarbonate (20 mL) was added to the reaction mixture, which was subsequently diluted with DCM (25 mL). The organic layer was separated and the aqueous layer was extracted with DCM (2 x 25 mL). The organic layers were combined, washed with brine (15 mL), dried (Na_2SO_4), filtered, and concentrated

under reduced pressure via rotary evaporation. The resulting residue was purified via careful flash chromatography (SiO2; petroleum ether / EtOAc 20:1) to afford 116 mg (71%) of a clear oil. The product contained very significant rotomers, even at 70 °C, and was used immediately in the next step without full characterization. The unassigned/unprocessed ^{1}H NMR spectra at rt and at 70 °C in C_6D_6 are included below for reference.

<u>tert-butyl ((5S,6S)-5-((tert-butyldimethylsilyl)oxy)-6,7,8,9-tetrahydro-5H-benzo[7]annulen-6-yl)carbamate</u> **7**

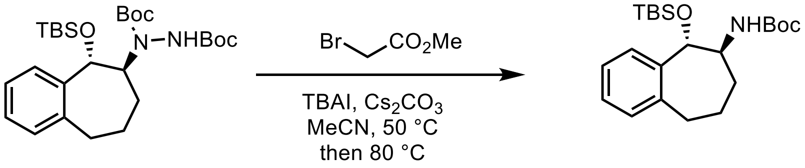

A vial containing a magnetic stir bar was charge with the protected alcohol (55.8 mg, 0.110 mmol). NBu_4I (40.6 mg, 0.110 mmol) was added and the vial was placed under a nitrogen atmosphere and brought into the glovebox. In the glovebox, freshly ground Cs_2CO_3 (127 mg, 0.390 mmol) was added. The vial was brought out of the glovebox and dry acetonitrile (0.7 mL) was added followed by methyl bromoacetate (11.4 µL, 0.120 mmol). The reaction mixture was allowed to stir in a 50 °C oil bath for 18 h at which point the oil bath temperature was raised to 80 °C and the reaction mixture was allowed to stir for an additional 48 h. At this point, the reaction was quenched with a saturated aqueous solution of ammonium chloride (15 ml) and diluted with 2 mL H_2O and ethyl acetate (25 mL). The organic layer was separated and the aqueous layer was extracted with ethyl acetate (2 x 25 mL). The combined organic layers were washed with brine (20 mL), dried (MgSO4), filtered, and concentrated under reduced pressure using rotary evaporation. The resulting residue was purified via flash chromatography (SiO2; petroleum ether / EtOAc 30:1) to afford 21.7 mg (51%) of a clear oil.

IR(CH_2Cl_2) 3441, 3337, 2954, 2930, 2857, 1719, 1494, 1390, 1365, 1251, 1170, 1061, 993, 941, 881, 859, 837, 777, 750, 666 cm^{-1}. ^{1}H NMR (500 MHz, Benzene-d_6) δ 7.05 (dd, J = 7.2, 1.6 Hz, 1H), 6.99 – 6.90 (m, 2H), 6.88 (dd, J = 7.3, 1.5 Hz, 1H), 4.89 (d, J = 6.2 Hz, 1H), 4.49 – 4.37 (m, 1H), 4.26 (d, J = 9.1 Hz, 1H), 2.48 – 2.38 (m, 1H), 2.33 (ddt, J = 13.9, 6.9, 1.5 Hz, 1H), 1.81 (d, J = 14.2 Hz, 1H), 1.59 – 1.45 (m, 1H), 1.40 (s, 9H), 1.25 – 1.13 (m, 1H), 0.89 (s, 9H), 0.04 (s, 3H), -0.22 (s, 3H). ^{13}C NMR (126 MHz, Benzene) δ 155.21, 143.70, 139.28, 130.97, 130.60, 128.51, 126.03, 78.86, 78.43, 51.88, 35.29, 30.21, 28.48, 26.00, 23.45, 18.27, -4.74, -5.07. HRMS (EI) calculated for $C_{22}H_{37}NO_3Si$ (Na^+) 414.2435, found: 414.2435. $[\alpha]_D^{22}$ = 12.6 (c = 0.54, $CHCl_3$). R_f (petroleum ether / EtOAc 25:1) = 0.34.

<u>tert-butyl ((3aS,10bR)-2-oxo-4,5,6,10b-tetrahydro-2H-benzo[3,4]cyclohepta[1,2-d]oxazol-3(3aH)-yl)carbamate</u> **SIV**

The procedure used was based upon the work of Gmeiner and Bollinger:[3]

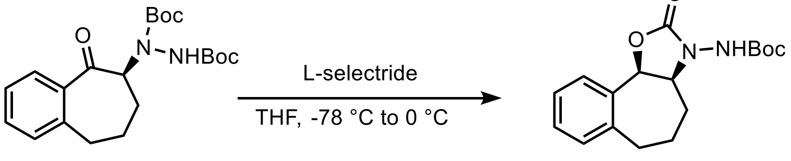

A flask equipped with a magnetic stir bar was charged with the ketone (66.2 mg, 0.170 mmol) and placed under nitrogen. THF (10 mL) was added and the flask was cooled to -78 °C. L-selectride (1.0 M in THF, 0.19 mL, 0.19 mmol) was added dropwise and the reaction was allowed to slowly warm up to rt overnight. In the morning, the reaction mixture was quenched with a saturated aqueous solution of ammonium chloride and diluted with 20 mL $CHCl_3$. The organic layer was separated and the aqueous layer was extracted with $CHCl_3$ (2 x 20 mL). The organic layers were combined, washed with brine (15 mL), dried ($MgSO_4$), filtered, and concentrated under reduced pressure via rotary evaporation. The resulting residue was purified via flash chromatography (SiO2; petroleum ether / EtOAc 3.5:1) to afford 50.6 mg (94%) of a clear oil that was 90-95% pure.

IR($CDCl_3$) 3251, 2947, 1780, 1729, 1457, 1370, 1257, 1160, 1029, 912, 734 cm^{-1}. ^{1}H NMR (400 MHz, Chloroform-*d*) δ 7.45 (dd, *J* = 5.3, 3.7 Hz, 1H), 7.33 – 7.22 (m, 2H), 7.13 – 7.07 (m, 1H), 6.58 (s, 1H), 5.82 (d, *J* = 9.6 Hz, 1H), 4.21 (s, 1H), 2.76 (dd, *J* = 14.6, 7.5, 2.1 Hz, 1H), 2.50 (ddd, *J* = 14.0, 11.3, 7.4 Hz, 1H), 1.88 – 1.61 (m, 3H), 1.47 (s, 9H), 1.21 – 1.10 (m, 1H) some "grease" is visible in the nmr spectrum. ^{13}C NMR (101 MHz, cdcl$_3$) δ 156.57, 154.48, 135.79, 134.40, 128.76, 128.64, 127.10, 124.24, 82.62, 76.09, 57.30, 30.31, 28.27, 25.24, 21.31. HRMS (EI) calculated for $C_{17}H_{22}N_2O_4$ (Na^+) 341.1472, found: 341.1472. R_f (petroleum ether / EtOAc 4:1) = 0.15. HPLC: (*Chiralpak® IB*, heptane-isopropanol 90:10, 0.8 mL/min, 220 nm): t_R(minor) = 9.5 min, t_R(major) = 12.2 min, 97% *ee*.

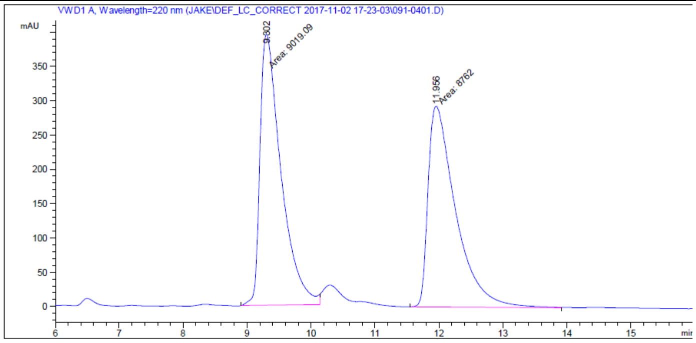

Peak #	RetTime [min]	Type	Width [min]	Area mAU *s	Height [mAU]	Area %
1	9.302	MM	0.3807	9019.09375	394.82556	50.7229
2	11.956	MM	0.4989	8762.00000	292.71005	49.2771

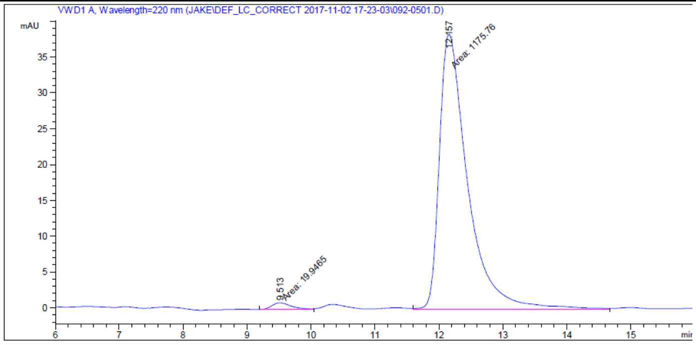

Peak #	RetTime [min]	Type	Width [min]	Area mAU *s	Height [mAU]	Area %
1	9.513	MM	0.3565	19.94647	9.32618e-1	1.6682
2	12.157	MM	0.5111	1175.76233	38.33860	98.3318

(3aS,10bR)-3,3a,4,5,6,10b-hexahydro-2H-benzo[3,4]cyclohepta[1,2-d]oxazol-2-one **8**

One pot reduction/elimination:

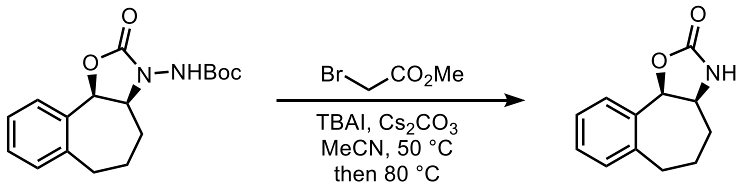

A vial containing a magnetic stir bar was charge with the cyclic carbamate (34 mg, 0.11 mmol). NBu_4I (39.6 mg, 0.107 mmol) was added and the vial was placed under a nitrogen atmosphere and brought into the glovebox. In the glovebox, freshly ground Cs_2CO_3 (122 mg, 0.375 mmol) was added. The vial was brought out of the glovebox and dry acetonitrile (0.7 mL) was added followed by methyl bromoacetate (11.1 µL, 0.117 mmol). The reaction mixture was allowed to stir in a 50 °C oil bath for 18 h at which point the oil bath temperature was raised to 80 °C and the reaction mixture was allowed to stir for an additional 24 h. At this point, the reaction was quenched with a saturated aqueous solution of ammonium chloride (15 ml) and diluted with 2 mL H_2O and ethyl acetate (25 mL). The organic layer was separated and the aqueous layer was extracted with ethyl acetate (2 x 25 mL). The combined organic layers were washed with brine (20 mL), dried (MgSO4), filtered, and concentrated under reduced pressure using rotary evaporation. The resulting residue was purified via flash chromatography (SiO2; petroleum ether / EtOAc 1:1) to afford 12.8 mg (59%) of a clear oil.

Two pot alkylation/elimination:

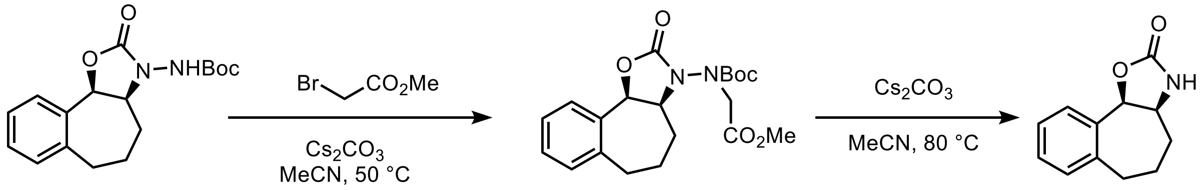

A vial equipped with a magnetic stir bar was charged with the cyclic carbamate (59.7 mg, 0.188 mmol), placed under a nitrogen atmosphere, and brought into the glovebox. In the glovebox, freshly ground Cs_2CO_3 (184 mg, 0.564 mmol) was added to the vial and the vial was removed from the glovebox. Dry acetonitrile (1.2 mL) and dry $CDCl_3$ (for solubility, 0.5 mL) was added followed by methyl bromoacetate (44.5 µL, 0.470 mmol). The reaction mixture was allowed to stir in a 50 °C oil bath for 17 h. At this point, the reaction was quenched with a saturated aqueous solution of ammonium chloride (15 ml) and diluted with 2 mL H_2O and ethyl acetate (25 mL). The organic layer was separated and the aqueous layer was extracted with ethyl acetate (2 x 25 mL). The combined organic layers were washed with brine (20 mL), dried (MgSO4), filtered, and concentrated under reduced pressure using rotary evaporation. The resulting residue was purified via a short silica plug (SiO2; petroleum ether / EtOAc 3:1). The purified material was utilized immediately in the following step without characterization due to the presence of significant rotomers.

A vial equipped with a magnetic stir bar was charged with the purified alkylation product, placed under an atmosphere of nitrogen, and brought into the glovebox. In the glovebox, freshly ground Cs_2CO_3 (184 mg, 0.564 mmol) was added to the vial. The vial was removed from the glovebox and dry acetonitrile (1.0 mL) was added. The reaction was allowed to stir in an 80 °C oil bath for 20 h. At this point, the reaction was quenched with a saturated aqueous solution of ammonium chloride (15 ml) and diluted with 2 mL H_2O and ethyl acetate (25 mL). The organic layer was separated and the aqueous layer was extracted with ethyl acetate (2 x 25 mL). The combined organic layers were washed with brine (15 mL),

dried (MgSO4), filtered, and concentrated under reduced pressure using rotary evaporation. The resulting residue was purified via careful flash chromatography (SiO2; petroleum ether / EtOAc 1:1) to afford 23 mg (64% over 2 steps) of a white solid (mp 167-169 °C).

IR($CDCl_3$) 3283, 3244, 3220, 2925, 2844, 1744, 1705, 1447, 1273, 1241, 1108, 1027, 954, 899, 757, 726, 662 cm^{-1}. ^{1}H NMR (400 MHz, Chloroform-*d*) δ 7.53 – 7.46 (m, 1H), 7.32 – 7.26 (m, 2H), 7.17 – 7.07 (m, 1H), 5.93 (s, 1H), 5.85 (d, *J* = 9.5 Hz, 1H), 4.22 – 4.09 (m, 1H), 2.78 (ddd, *J* = 14.4, 7.4, 2.6 Hz, 1H), 2.52 (ddd, *J* = 14.4, 11.2, 7.7 Hz, 1H), 1.84 – 1.58 (m, 3H), 1.38 – 1.17 (m, 1H). ^{13}C NMR (101 MHz, $cdcl_3$) δ 158.67, 135.78, 134.80, 128.77, 128.67, 127.08, 124.86, 79.05, 53.62, 30.24, 27.99, 21.66. HRMS (EI) calculated for $C_{12}H_{13}NO_2$ (Na^+) 226.0839, found: 226.0839. $[\alpha]_D^{23}$ = -65.8 (c = 0.69, $CHCl_3$). R_f (petroleum ether / EtOAc 1:1) = 0.22.

di-*tert*-butyl 1-(1-oxo-1,2,3,4-tetrahydronaphthalen-2-yl)hydrazine-1,2-dicarboxylate **2l**

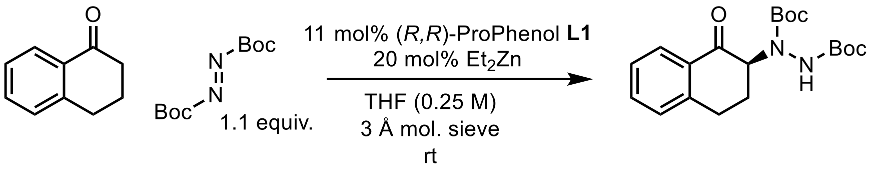

(*R*,*R*)-ProPhenol **L1** (13.7 mg, 0.0214 mmol), α-tetralone (30.0 mg, 0.205 mmol), and activated 3 Å molecular sieves (5 mg) were measured into a microwave vial equipped with stir bar. The flask was placed under a nitrogen atmosphere and THF (0.5 mL) was added. After stirring for 15 minutes, diethylzinc (1.0 M in hexanes, 40μL, 0.040 mmol) was added and the reaction was allowed to stir for an additional 15 minutes. At this time, a premixed (15 min) THF (0.3 mL) solution of di-*tert*-butyl azodicarboxylate (50.0 mg, 0.217 mmol) and 3 Å molecular sieves (5 mg) was added. The reaction was allowed to stir at rt for 14 hours at which point the reaction mixture was filtered through a small plug of fluorosil. The residue was concentrated under reduced pressure using rotary evaporation. The resulting residue was purified via flash chromatography (SiO2; DCM to petroleum ether / EtOAc 5:1 to petroleum ether / EtOAc 4:1) to afford 67.1 mg (86%) of a pale-yellow oil.

IR(C_6D_6) 3270, 2940, 2890, 1670, 1580, 1440, 1370, 1350, 1300, 1220, 1140, 1040, 950, 920, 880, 840, 750 cm^{-1}. ^{1}H NMR (500 MHz, 75 °C, Benzene-d_6) δ 8.13 – 7.99 (m, 1H), 7.10 – 7.00 (m, 1H), 6.94 (t, *J* = 7.6 Hz, 1H), 6.73 (d, *J* = 7.8 Hz, 1H), 6.49 (s, 1H), 5.08 (s, 1H), 2.67 – 2.53 (m, 1H), 2.49 – 2.29 (m, 2H), 2.26 – 1.93 (m, 1H), 1.46 (s, 9H), 1.39 (d, *J* = 4.0 Hz, 9H). ^{13}C NMR (126 MHz, 75 °C, Benzene-d_6) δ 194.39, 155.78, 144.10, 133.42, 133.03, 128.77, 126.73, 81.36, 80.32, 64.65, 29.08, 28.41, 28.38, 28.21. HRMS (EI) calculated for $C_{20}H_{28}N_2O_5$ (Na^+) 399.1890, found: 399.1892. R_f (petroleum ether / EtOAc 4:1) = 0.27. $[\alpha]_D^{23}$ = 7.7 (c = 0.37, $CHCl_3$). HPLC: (*Chiralpak® IA*, heptane-isopropanol 95:5, 0.8 mL/min, 254 nm): t_R(minor) = 20.9 min, t_R(major) = 15.9 min, 94% *ee*.

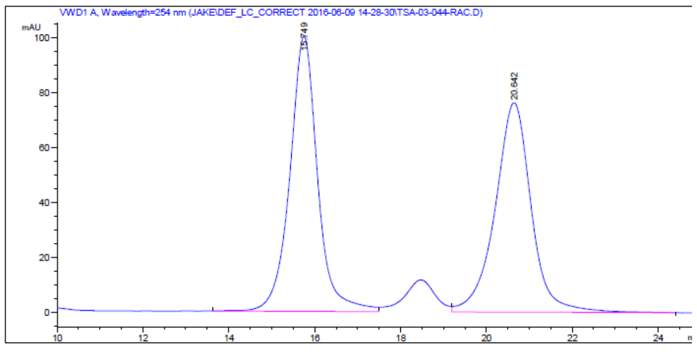

Peak #	RetTime [min]	Type	Width [min]	Area mAU *s	Height [mAU]	Area %
1	15.749	BV	0.6721	4517.42285	100.10468	50.3664
2	20.642	VB	0.8738	4451.69971	76.19547	49.6336

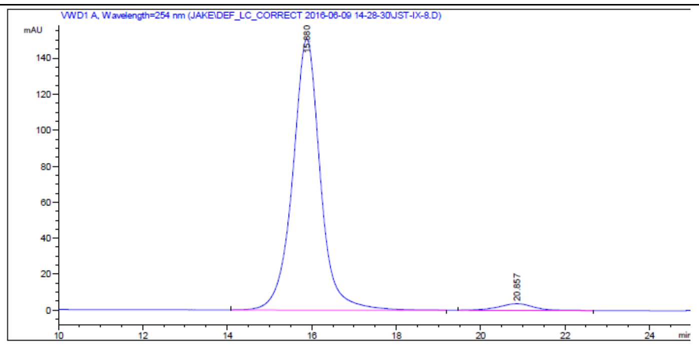

Peak #	RetTime [min]	Type	Width [min]	Area mAU *s	Height [mAU]	Area %
1	15.880	BB	0.6798	6853.11328	150.48248	96.9154
2	20.857	BB	0.8728	218.11800	3.73891	3.0846

The title compound was also run on an HPLC under the following conditions: HPLC: (*Chiralpak® IB*, heptane-isopropanol 95:5, 0.8 mL/min, 254 nm): t_R(minor) = 6.6 min, t_R(major) = 7.9 min, 93% *ee*. Under these conditions, the major enantiomer was the second enantiomer to elute, in line with the results obtained by Ma and co-workers for the same compound under the same HPLC conditions. As such, the absolute stereochemistry was assigned to be the same as that reported by Ma. All other trisubstituted products were assigned by analogy.[4]

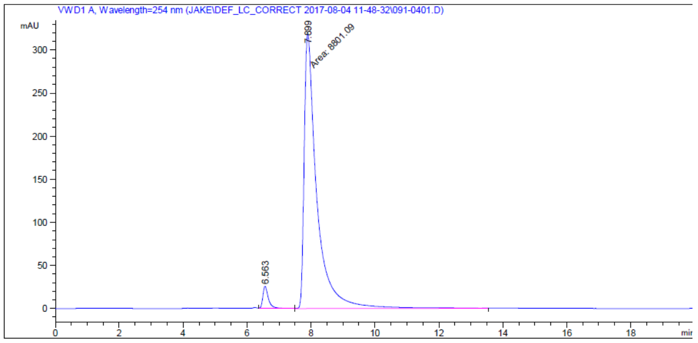

Peak #	RetTime [min]	Type	Width [min]	Area mAU *s	Height [mAU]	Area %
1	6.563	VB	0.1814	310.39529	25.75699	3.4066
2	7.899	MM	0.4602	8801.09473	318.72879	96.5934

<u>di-tert-butyl 1-(7-bromo-1-oxo-1,2,3,4-tetrahydronaphthalen-2-yl)hydrazine-1,2-dicarboxylate</u> **2m**

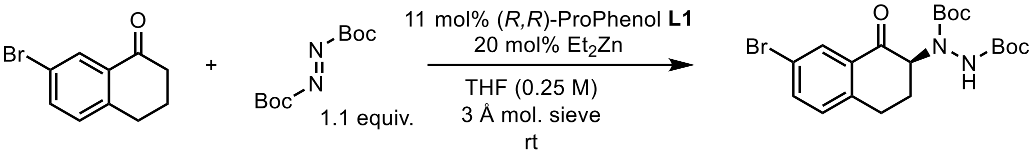

(*R*,*R*)-ProPhenol **L1** (13.7 mg, 0.0214 mmol), 7-bromo-1-tetralone (45.0 mg, 0.200 mmol), and activated 3 Å molecular sieves (5 mg) were measured into a microwave vial equipped with stir bar. The flask was placed under a nitrogen atmosphere and THF (0.5 mL) was added. After stirring for 15 minutes, diethylzinc (1.0 M in hexanes, 40μL, 0.040 mmol) was added and the reaction was allowed to stir for an additional 15 minutes. At this time, a premixed (15 min) THF (0.3 mL) solution of di-*tert*-butyl azodicarboxylate (50.7 mg, 0.220 mmol) and 3 Å molecular sieves (5 mg) was added. The reaction was allowed to stir at rt for 3.5 h at which point the reaction mixture was filtered through a small plug of fluorosil. The residue was concentrated under reduced pressure using rotary evaporation. The resulting residue was purified rapidly via flash chromatography (SiO2; DCM to petroleum ether / EtOAc 6:1 to petroleum ether / EtOAc 5:1 to petroleum ether / EtOAc 4:1) to afford 89.7 mg (98%) of a white foam (m.p. 56-57 °C).

IR($CDCl_3$) 3346, 2977, 2931, 1747, 1695, 1477, 1393, 1368, 1313, 1241, 1216, 1156, 733 cm^{-1}. ^{1}H NMR (500 MHz, 75 °C, Benzene-d_6) δ 8.18 (d, J = 2.2 Hz, 1H), 6.40 (d, J = 9.2 Hz, 2H), 4.97 (s, 1H), 2.52 – 2.22 (series of m, 3H total), 2.10 (m, 1H), 1.46 (s, 9H), 1.40 (s, 9H). ^{13}C NMR (126 MHz, 75 °C, Benzene) δ 193.12, 155.83 (broad), 155.63, 142.63, 136.23, 134.45, 130.86, 130.53, 121.02, 81.58, 80.50, 64.72 (broad), 28.48, 28.40, 28.36, 27.87. HRMS (EI) calculated for $C_{20}H_{27}BrN_2O_5$ (Na^+) 477.0996, found: 477.0995. R_f (petroleum ether / EtOAc 5:1) = 0.35. $[\alpha]_D^{23}$ = 17.4 (c = 0.80, $CHCl_3$). HPLC: (*Chiralpak® IA*, heptane-isopropanol 95:5, 0.8 mL/min, 254 nm): t_R(minor) = 13.6 min, t_R(major) = 19.0 min, 90% *ee*.

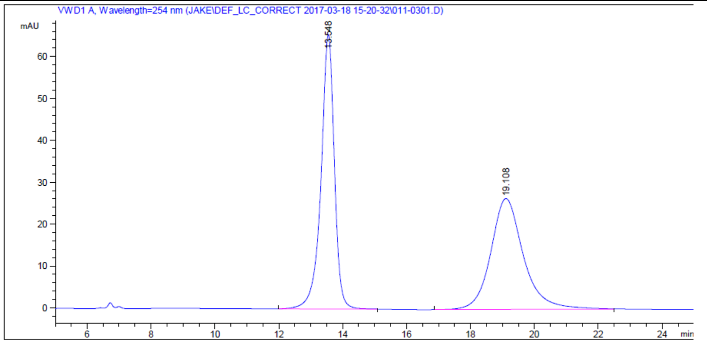

Peak #	RetTime [min]	Type	Width [min]	Area mAU *s	Height [mAU]	Area %
1	13.548	BB	0.4365	1914.89771	65.62157	50.4364
2	19.108	BB	1.0623	1881.75879	26.47285	49.5636

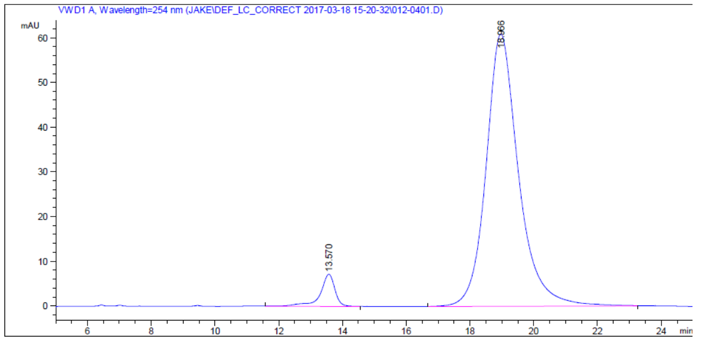

Peak #	RetTime [min]	Type	Width [min]	Area mAU *s	Height [mAU]	Area %
1	13.570	VB	0.4758	234.35204	7.20031	5.1433
2	18.966	BB	1.0550	4322.14502	60.91126	94.8567

<u>di-tert-butyl 1-(5-bromo-1-oxo-1,2,3,4-tetrahydronaphthalen-2-yl)hydrazine-1,2-dicarboxylate</u> **2n**

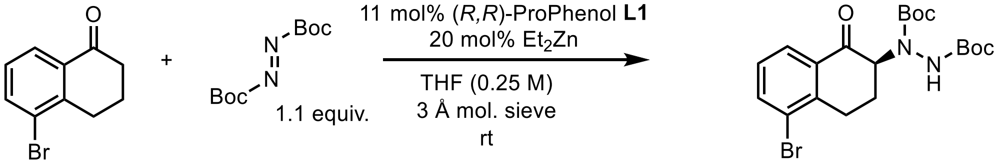

(*R*,*R*)-ProPhenol **L1** (13.7 mg, 0.0214 mmol), 5-bromo-1-tetralone (45.0 mg, 0.200 mmol), and activated 3 Å molecular sieves (5 mg) were measured into a microwave vial equipped with stir bar. The flask was placed under a nitrogen atmosphere and THF (0.5 mL) was added. After stirring for 15 minutes, diethylzinc (1.0 M in hexanes, 40µL, 0.040 mmol) was added and the reaction was allowed to stir for an additional 15 minutes. At this time, a premixed (15 min) THF (0.3 mL) solution of di-*tert*-butyl azodicarboxylate (50.7 mg, 0.220 mmol) and 3 Å molecular sieves (5 mg) was added. The reaction was allowed to stir at rt for 3 h at which point the reaction mixture was filtered through a small plug of fluorosil. The residue was concentrated under reduced pressure using rotary evaporation. The resulting residue was purified rapidly via flash chromatography (SiO2; DCM to petroleum ether / EtOAc 7:1 to petroleum ether / EtOAc 6:1) to afford 83.2 mg (91 %) of a clear viscous oil.

IR($CDCl_3$) 3370, 2978, 2932, 1747, 1697, 1588, 1479, 1393, 1367, 1318, 1254, 1155, 1050, 757 cm^{-1}. ^{1}H NMR (500 MHz, 75 °C, Benzene-d_6) δ 7.89 (dd, J = 7.7, 1.3 Hz, 1H), 7.34 (dd, J = 7.8, 1.3 Hz, 1H), 6.65

(t, J = 7.8 Hz, 1H), 6.43 (s, 1H), 4.98 (s, 1H), 2.85 (d, J = 18.2 Hz, 1H), 2.48 – 2.28 (two obscured m, 2H total), 2.04 (qd, J = 12.9, 4.6 Hz, 1H), 1.45 (s, 9H), 1.41 (s, 9H). ^{13}C NMR (126 MHz, 75 °C, Benzene) δ 193.56, 155.81, 155.65, 143.14, 137.42, 134.80, 127.22, 124.88, 81.50, 80.47, 63.93 (broad), 29.68, 28.42, 28.37, 26.75, 1 aromatic carbon peak obscured. HRMS (EI) calculated for $C_{20}H_{27}BrN_2O_5$ (Na^+) 477.0996, found: 477.0997. $[\alpha]_D^{23}$ = -5.9 (c = 1.29, $CHCl_3$). HPLC: (*Chiralpak® IA*, heptane-isopropanol 95:5, 0.8 mL/min, 254 nm): t_R(minor) = 21.7 min, t_R(major) = 16.7 min, 95% *ee*.

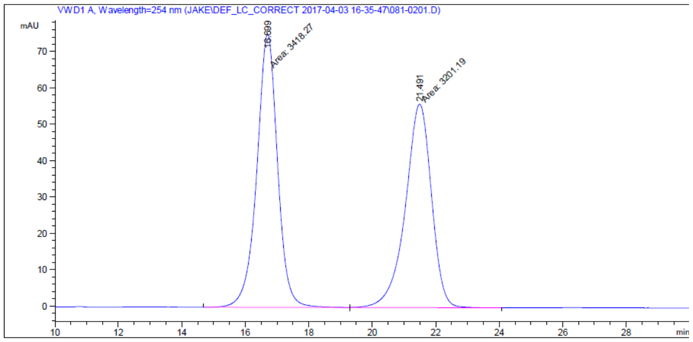

Peak #	RetTime [min]	Type	Width [min]	Area mAU *s	Height [mAU]	Area %
1	16.699	MM	0.7581	3418.27319	75.15451	51.6397
2	21.491	MM	0.9534	3201.18921	55.95829	48.3603

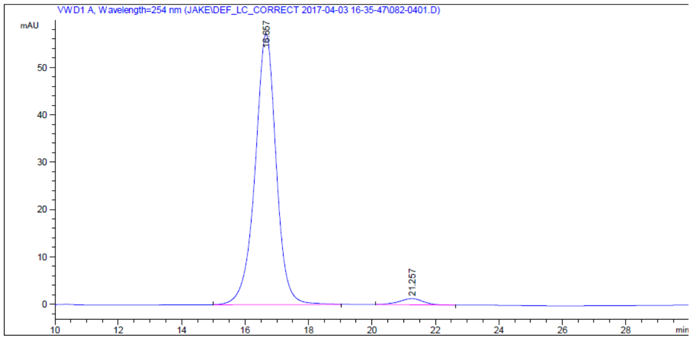

Peak #	RetTime [min]	Type	Width [min]	Area mAU *s	Height [mAU]	Area %
1	16.657	BB	0.6967	2644.43384	57.18843	97.5052
2	21.257	BB	0.7970	67.66010	1.28647	2.4948

di-tert-butyl 1-(7-fluoro-1-oxo-1,2,3,4-tetrahydronaphthalen-2-yl)hydrazine-1,2-dicarboxylate **2o**

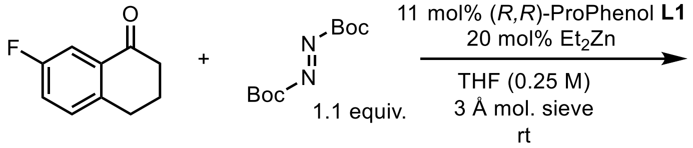

(R,R)-ProPhenol **L1** (13.7 mg, 0.0214 mmol), 7-fluoro-1-tetralone (32.8 mg, 0.200 mmol), and activated 3 Å molecular sieves (5 mg) were measured into a microwave vial equipped with stir bar. The flask was placed under a nitrogen atmosphere and THF (0.5 mL) was added. After stirring for 15 minutes, diethylzinc (1.0 M in hexanes, 40μL, 0.040 mmol) was added and the reaction was allowed to stir for an additional 15 minutes. At this time, a premixed (15 min) THF (0.3 mL) solution of di-*tert*-butyl azodicarboxylate (50.7 mg, 0.220 mmol) and 3 Å molecular sieves (5 mg) was added. The reaction was allowed to stir at rt for 1.3 h at which point the reaction mixture was filtered through a small plug of fluorosil. The residue was concentrated under reduced pressure using rotary evaporation. The resulting residue was purified rapidly via flash chromatography (SiO2; DCM to petroleum ether / EtOAc 6:1 to petroleum ether / EtOAc 4:1) to afford 63.8 mg (81 %) of a clear oil.

IR($CDCl_3$) 3314, 2979, 2932, 1747, 1694, 1614, 1493, 1457, 1426, 1394, 1368, 1307, 1262, 1241, 1158, 1052, 891, 850, 812 cm^{-1}. ^{1}H NMR (500 MHz, 75 °C, Benzene-d_6) δ 7.71 (dd, J = 9.0, 2.9 Hz, 1H), 6.73 (td, J = 8.3, 2.8 Hz, 1H), 6.54 (dd, J = 8.5, 5.1 Hz, 1H), 6.43 (s, 1H), 4.99 (s, 1H), 2.55 – 2.42 (m, 1H), 2.42 – 2.29 (m, 2H), 2.20 – 2.05 (m, 1H), 1.46 (s, 9H), 1.40 (s, 9H). ^{13}C NMR (126 MHz, 75 °C,

Benzene-d_6) δ 193.42, 162.11 (d, $J_{C\text{-}F}$ = 246.2 Hz), 155.84, 155.65, 139.74, 134.55, 130.60 (d, $J_{C\text{-}F}$ = 6.1 Hz), 120.73 (d, $J_{C\text{-}F}$ = 22.3 Hz), 113.74 (d, $J_{C\text{-}F}$ = 22.0 Hz), 81.54 , 80.48 , 64.89 (broad), 28.40, 28.36, 28.32, 28.15. HRMS (EI) calculated for $C_{20}H_{27}FN_2O_5$ (Na^+) 417.1796, found: 417.1793. R_f (petroleum ether / EtOAc 6:1) = 0.20. $[\alpha]_D^{22}$ = -2.4 (c = 0.33, $CHCl_3$). HPLC: (*Chiralpak® IA*, heptane-isopropanol 98:2, 0.8 mL/min, 254 nm): t_R(minor) = 24.5 min, t_R(major) = 29.8 min**,** 96% *ee*.

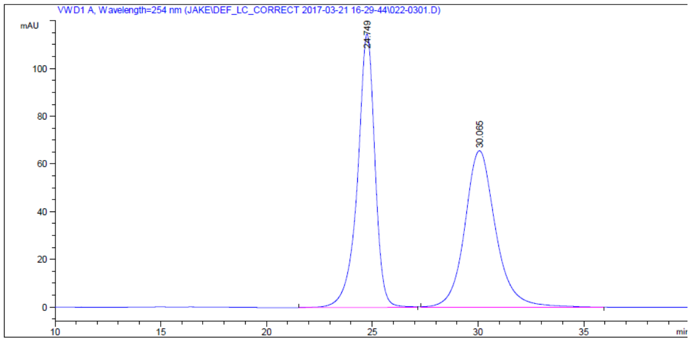

Peak #	RetTime [min]	Type	Width [min]	Area mAU *s	Height [mAU]	Area %
1	24.749	BB	0.8553	6598.76953	114.59664	49.6357
2	30.065	BB	1.5306	6695.63379	65.64745	50.3643

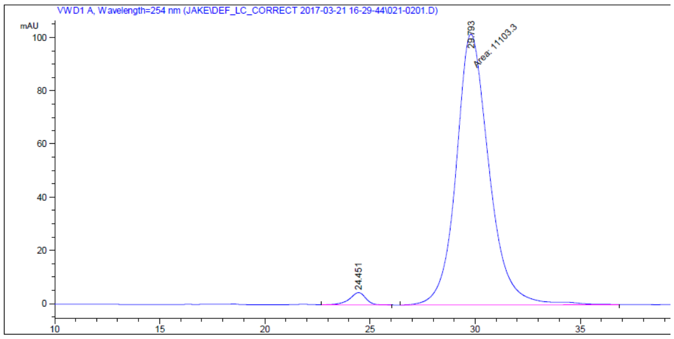

Peak #	RetTime [min]	Type	Width [min]	Area mAU *s	Height [mAU]	Area %
1	24.451	VB	0.7997	247.74710	4.60321	2.1826
2	29.793	MM	1.8187	1.11033e4	101.75088	97.8174

Large-scale procedure:
(*R*,*R*)-ProPhenol **L1** (21.1 mg, 0.0330 mmol), 7-fluoro-1-tetralone (164 mg, 1.00 mmol), and activated 3 Å molecular sieves (25 mg) were measured into a microwave vial equipped with stir bar. The flask was placed under a nitrogen atmosphere and THF (2.5 mL) was added. After stirring for 15 minutes, diethylzinc (1.0 M in hexanes, 60μL, 0.060 mmol) was added and the reaction was allowed to stir for an additional 15 minutes. At this time, a premixed (15 min) THF (1.5 mL) solution of di-*tert*-butyl azodicarboxylate (253 mg, 1.10 mmol) and 3 Å molecular sieves (25 mg) was added. The reaction was allowed to stir at rt for 5 h at which point the reaction mixture was filtered through a small plug of fluorosil. The residue was concentrated under reduced pressure using rotary evaporation. The resulting residue was purified rapidly via flash chromatography (SiO2; petroleum ether / EtOAc 6:1) to afford 305 mg (77 %) of a clear oil.

HPLC: (*Chiralpak® IA*, heptane-isopropanol 98:2, 0.8 mL/min, 254 nm): t_R(minor) = 21.1 min, t_R(major) = 25.1 min**,** 96% *ee*. Note: The difference in retention time between the large-scale and the standard-scale retention times is due to normal column drift, as the large-scale reaction was performed and run on the HPLC 1 year after the standard-scale reaction. The same racemic sample was utilized for both HPLC runs.

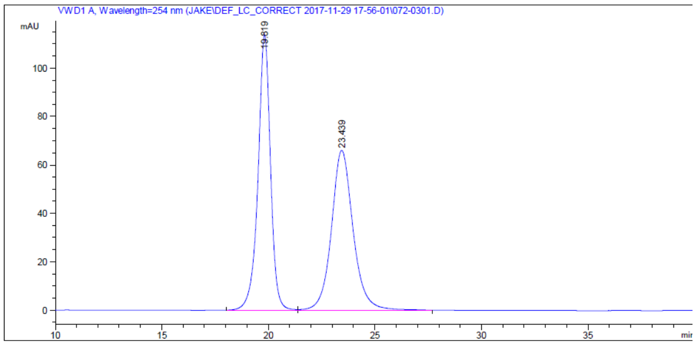

Peak #	RetTime [min]	Type	Width [min]	Area mAU *s	Height [mAU]	Area %
1	19.819	BV	0.6101	4641.94922	113.72623	49.8380
2	23.439	VB	1.0592	4672.11768	65.97396	50.1620

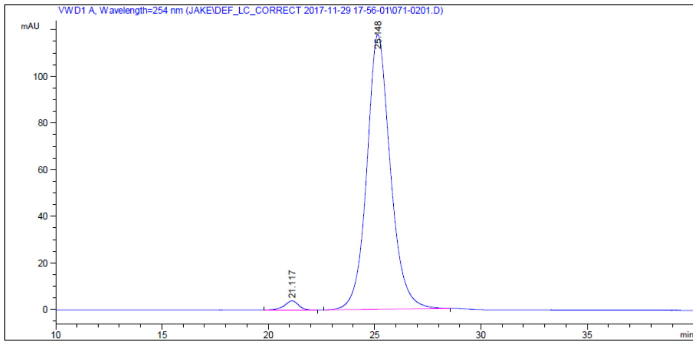

Peak #	RetTime [min]	Type	Width [min]	Area mAU *s	Height [mAU]	Area %
1	21.117	BB	0.6786	176.26579	3.90097	1.9043
2	25.148	BB	1.1555	9079.96289	117.79635	98.0957

di-tert-butyl 1-(7-nitro-1-oxo-1,2,3,4-tetrahydronaphthalen-2-yl)hydrazine-1,2-dicarboxylate **2p**

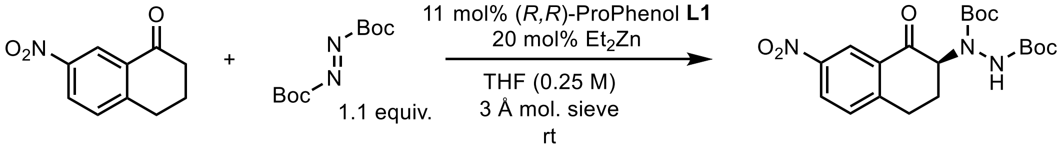

(*R*,*R*)-ProPhenol **L1** (13.7 mg, 0.0214 mmol), 7-nitro-1-tetralone (38.2 mg, 0.200 mmol), and activated 3 Å molecular sieves (5 mg) were measured into a microwave vial equipped with stir bar. The flask was placed under a nitrogen atmosphere and THF (0.5 mL) was added. After stirring for 15 minutes, diethylzinc (1.0 M in hexanes, 40µL, 0.040 mmol) was added and the reaction was allowed to stir for an additional 15 minutes. At this time, a premixed (15 min) THF (0.3 mL) solution of di-*tert*-butyl azodicarboxylate (50.7 mg, 0.220 mmol) and 3 Å molecular sieves (5 mg) was added. The reaction was allowed to stir at rt for 25 min at which point the reaction mixture was filtered through a small plug of fluorosil. The residue was concentrated under reduced pressure using rotary evaporation. The resulting residue was purified rapidly via preparatory TLC (SiO2; petroleum ether / EtOAc 1:1) to afford 65.7 mg (83 %) of a clear viscous oil.

IR($CDCl_3$) 3348, 2977, 1700, 1612, 1529, 1479, 1394, 1347, 1311, 1242, 1155, 760 cm^{-1}. ^{1}H NMR (500 MHz, 75 °C, Benzene-d_6) δ 8.70 (d, J = 2.5 Hz, 1H), 7.74 (dd, J = 8.4, 2.5 Hz, 1H), 6.43 (d, J = 8.4 Hz, 1H), 6.39 (s, 1H), 4.95 (s, 1H), 2.45 – 2.21 (m, 3H), 2.07 (tt, J = 15.3, 8.4 Hz, 1H), 1.47 (s, 9H), 1.41 (s, 9H). ^{13}C NMR (126 MHz, 75 °C, Benzene) δ 192.47, 149.64, 147.81, 133.42, 129.78, 127.02, 123.03, 81.85, 80.71, 64.47 (broad), 28.86, 28.38, 28.34, 27.40. HRMS (EI) calculated for $C_{20}H_{27}N_3O_7$ (Na^+) 444.1741, found: 444.1741. R_f (petroleum ether / EtOAc 1:1) = 0.68. $[\alpha]_D^{24}$ = 23.9 (c = 0.86, $CHCl_3$). HPLC: (*Chiralpak® IA*, heptane-isopropanol 90:10, 0.8 mL/min, 254 nm): t_R(minor) = 13.7 min, t_R(major) = 21.6 min**,** 94% *ee*.

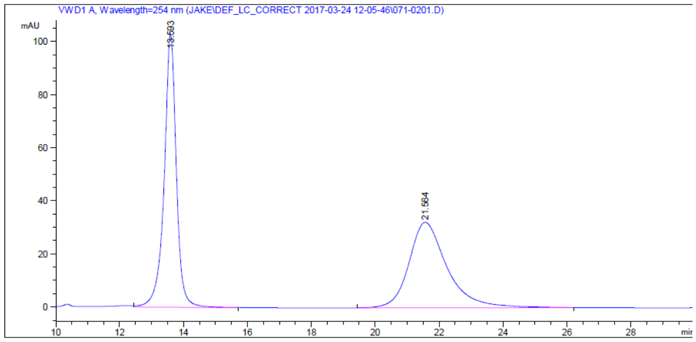

Peak #	RetTime [min]	Type	Width [min]	Area mAU *s	Height [mAU]	Area %
1	13.593	VB	0.3913	2730.11914	102.81159	50.5641
2	21.564	BB	1.2276	2669.20483	32.15595	49.4359

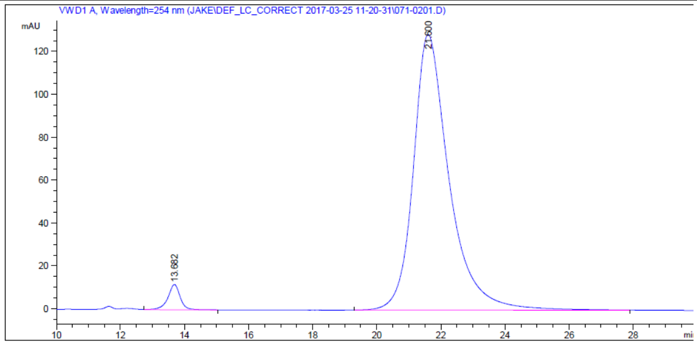

Peak #	RetTime [min]	Type	Width [min]	Area mAU *s	Height [mAU]	Area %
1	13.682	VB	0.3990	314.57767	11.77618	3.0507
2	21.600	BB	1.1435	9996.98926	128.49989	96.9493

di-tert-butyl 1-(6-cyano-1-oxo-1,2,3,4-tetrahydronaphthalen-2-yl)hydrazine-1,2-dicarboxylate **2q**

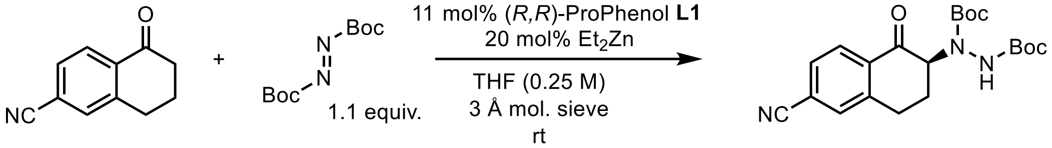

(*R*,*R*)-ProPhenol **L1** (13.7 mg, 0.0214 mmol), 6-cyano-1-tetralone (34.2 mg, 0.200 mmol), and activated 3 Å molecular sieves (5 mg) were measured into a microwave vial equipped with stir bar. The flask was placed under a nitrogen atmosphere and THF (0.5 mL) was added. After stirring for 15 minutes, diethylzinc (1.0 M in hexanes, 40μL, 0.040 mmol) was added and the reaction was allowed to stir for an additional 15 minutes. At this time, a premixed (15 min) THF (0.3 mL) solution of di-*tert*-butyl azodicarboxylate (50.7 mg, 0.220 mmol) and 3 Å molecular sieves (5 mg) was added. The reaction was allowed to stir at rt for 30 min at which point the reaction mixture was filtered through a small plug of fluorosil. The residue was concentrated under reduced pressure using rotary evaporation. The resulting residue was purified rapidly via preparatory TLC (SiO2; petroleum ether / EtOAc 1.5:1) to afford 69.1 mg (86 %) of a clear viscous oil.

IR($CDCl_3$) 3340, 3134, 2980, 2932, 1697, 1480, 1458, 1394, 1368, 1315, 1243, 1155, 757 cm^{-1}. ^{1}H NMR (500 MHz, 75 °C, Benzene-d_6) δ 7.73 (d, *J* = 8.1 Hz, 1H), 6.89 (d, *J* = 8.0 Hz, 1H), 6.70 (s, 1H), 6.37 (s, 1H), 4.92 (s, 1H), 2.41 – 2.25 (m, 2H), 2.25 – 2.15 (m, 1H), 2.05 (qd, *J* = 13.3, 12.9, 4.6 Hz, 1H), 1.45 (s, 9H), 1.40 (s, 9H). ^{13}C NMR (126 MHz, 75 °C, Benzene) δ 193.40, 155.84, 155.49, 144.34, 135.10, 132.63, 129.74, 128.29, 117.82, 117.37, 81.76, 80.69, 64.96 (broad), 28.45, 28.37, 28.33, 27.56. HRMS (EI) calculated for $C_{21}H_{27}N_3O_5$ (Na^+) 424.1843, found: 424.1842. R_f (petroleum ether / EtOAc 2:1) = 0.63. $[\alpha]_D^{23}$ = -6.6 (c = 1.12, $CHCl_3$). HPLC: (*Chiralpak® IA*, heptane-isopropanol 90:10, 0.8 mL/min, 254 nm): t_R(minor) = 22.6 min, t_R(major) = 17.2 min, 93% *ee*.

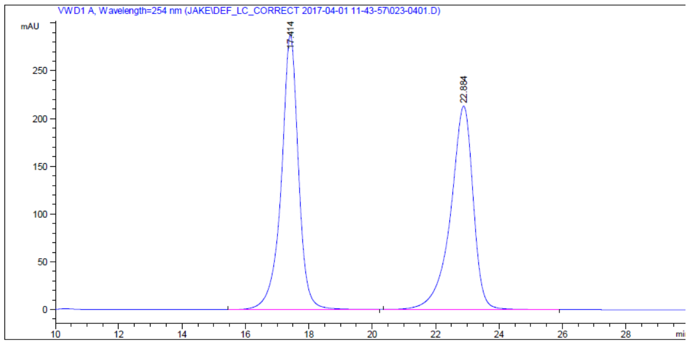

Peak #	RetTime [min]	Type	Width [min]	Area mAU *s	Height [mAU]	Area %
1	17.414	BB	0.5602	1.08809e4	288.00485	50.5995
2	22.884	BB	0.7443	1.06231e4	213.24203	49.4005

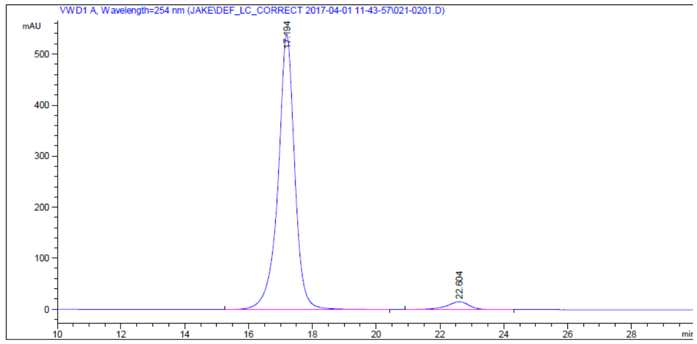

Peak #	RetTime [min]	Type	Width [min]	Area mAU *s	Height [mAU]	Area %
1	17.194	BB	0.5384	1.96258e4	538.91138	96.2549
2	22.604	BB	0.7346	763.60211	15.42896	3.7451

di-tert-butyl (S)-1-(4-oxochroman-3-yl)hydrazine-1,2-dicarboxylate **2r**

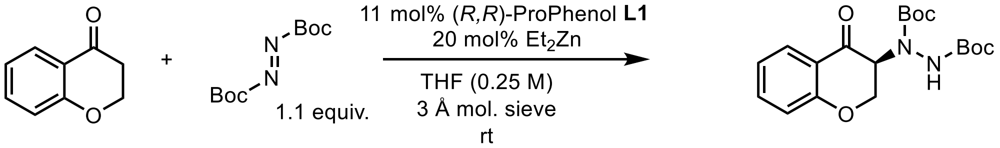

(*R*,*R*)-ProPhenol **L1** (13.7 mg, 0.0214 mmol), 4-chromanone (29.6 mg, 0.200 mmol), and activated 3 Å molecular sieves (5 mg) were measured into a microwave vial equipped with stir bar. The flask was placed under a nitrogen atmosphere and THF (0.5 mL) was added. After stirring for 15 minutes, diethylzinc (1.0 M in hexanes, 40μL, 0.040 mmol) was added and the reaction was allowed to stir for an additional 15 minutes at 4 °C. At this time, a premixed (15 min) THF (0.3 mL) solution of di-*tert*-butyl azodicarboxylate (50.7 mg, 0.220 mmol) and 3 Å molecular sieves (5 mg) at 4 °C was added. The reaction was allowed to stir at 4°C for 9 h at which point the reaction mixture was filtered through a small plug of fluorosil. The residue was concentrated under reduced pressure using rotary evaporation. The resulting residue was purified rapidly via flash chromatography (SiO2; petroleum ether / $CHCl_3$ 2:1 to DCM to to petroleum ether / EtOAc 4:1 to 3:1) to afford 39.4 mg (52 %) of a clear viscous oil.

IR($CHCl_3$) 3317, 2978, 2926, 1699, 1608, 1479, 1395, 1369, 1326, 1298, 1248, 1216, 1154, 1116, 1037, 1013, 867, 762 cm^{-1}. ^{1}H NMR (500 MHz, 75 °C, Benzene-d_6) δ 7.90 (d, *J* = 7.8 Hz, 1H), 7.03 – 6.92 (m, 1H), 6.72 (d, *J* = 8.4 Hz, 1H), 6.63 (t, *J* = 7.6 Hz, 1H), 6.45 (s, 1H), 5.32 (s, 1H), 4.76 (s, 1H), 4.42 (t, *J* = 12.2 Hz, 1H), 1.39 (d, *J* = 6.0 Hz, 9H), 1.33 (s, 9H). ^{13}C NMR (126 MHz, 75°C, Benzene) δ 189.22, 162.28, 155.94 (broad), 155.05, 135.92, 121.65, 121.47, 118.13, 82.09, 80.92, 69.07, 61.29 (broad), 28.20. One aromatic carbon peak is obscured. LRMS (EI) calculated for $C_{19}H_{26}N_2O_6$ (Na^+) 401.2, found: 401.2. $[\alpha]_D^{26}$ = -13.9 (c = 1.20, $CHCl_3$). HPLC: (*Chiralpak® IA*, heptane-isopropanol 95:10, 0.8 mL/min, 254 nm): t_R(minor) = 15.8 min, t_R(major) = 13.7 min, 75% *ee*.

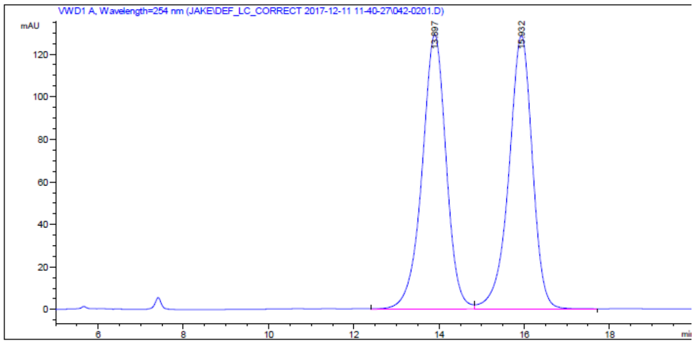

Peak #	RetTime [min]	Type	Width [min]	Area mAU *s	Height [mAU]	Area %
1	13.897	VV	0.5991	5077.63184	128.95918	50.7224
2	15.932	VB	0.5879	4933.00195	128.43765	49.2776

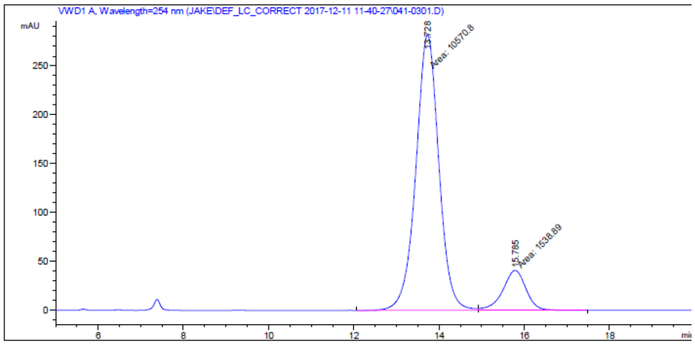

Peak #	RetTime [min]	Type	Width [min]	Area mAU *s	Height [mAU]	Area %
1	13.728	MM	0.6252	1.05708e4	281.78482	87.2920
2	15.785	MM	0.6292	1538.89111	40.76484	12.7080

di-*tert*-butyl 1-(1-oxo-1,2,3,4-tetrahydronaphthalen-2-yl)hydrazine-1,2-dicarboxylate **2s**

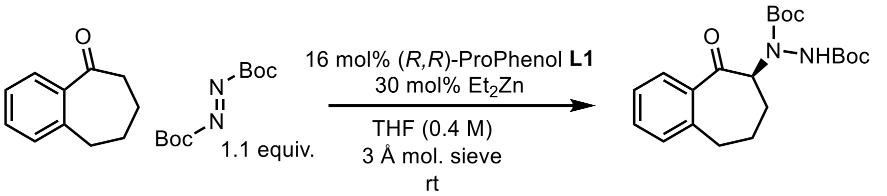

(*R*,*R*)-ProPhenol **L1** (20.6 mg, 0.0322 mmol), 1-benzosuberone (32.0mg, 0.200 mmol), and activated 3 Å molecular sieves (5 mg) were measured into a microwave vial equipped with stir bar. The flask was placed under a nitrogen atmosphere and THF (0.3 mL) was added. After stirring for 15 minutes, diethylzinc (1.0 M in hexanes, 60μL, 0.060 mmol) was added and the reaction was allowed to stir for an additional 15 minutes. At this time, a premixed (15 min) THF (0.2 mL) solution of di-*tert*-butyl azodicarboxylate (50.0 mg, 0.217 mmol) and 3 Å molecular sieves (5 mg) was added. The reaction was allowed to stir at rt for 14 hours at which point the reaction mixture was filtered through a small plug of fluorosil. The residue was concentrated under reduced pressure using rotary evaporation. The resulting residue was purified via flash chromatography (SiO2; DCM to petroleum ether / EtOAc 5:1 to petroleum ether / EtOAc 4:1) to afford 66.2 mg (85%) of a pale-yellow oil.

IR($CDCl_3$) 3270, 2940, 2890, 1670, 1580, 1440, 1370, 1350, 1300, 1220, 1140, 1040, 950, 920, 880, 840, 750 cm^{-1}. ^{1}H NMR (500 MHz, 75 °C, Benzene-d_6) δ 7.93 (dd, *J* = 7.7, 1.6 Hz, 1H), 7.04 (td, *J* = 7.4, 1.6 Hz, 1H), 6.97 (t, *J* = 7.3 Hz, 1H), 6.78 (d, *J* = 7.5 Hz, 1H), 6.67 (s, 1H), 5.22 (s, 1H), 2.57 (ddd, *J* = 15.5, 11.5, 3.9 Hz, 1H), 2.45 – 2.35 (m, 1H), 2.35 – 2.28 (m, 1H), 1.96 – 1.83 (m, 1H), 1.71 – 1.60 (m, 1H), 1.43 (s, 18H). ^{13}C NMR (126 MHz, 75 °C, Benzene-d_6) δ 201.83, 155.99, 155.01, 142.37, 137.49, 132.34, 130.17, 129.88, 126.83, 81.36, 80.28, 66.41, 33.96, 28.46, 28.36, 26.72, 24.38. HRMS (EI) calculated for $C_{21}H_{30}N_2O_5$ (Na^+) 413.2047, found: 413.2044. R_f (petroleum ether / EtOAc 4:1) = 0.40. $[\alpha]_D^{23}$ = 40.3 (c =

0.66, $CHCl_3$). HPLC: (*Chiralpak® IB*, heptane-isopropanol 98:2, 0.8 mL/min, 254 nm): t_R(minor) = 7.5 min, t_R(major) = 9.3 min, 98% *ee*.

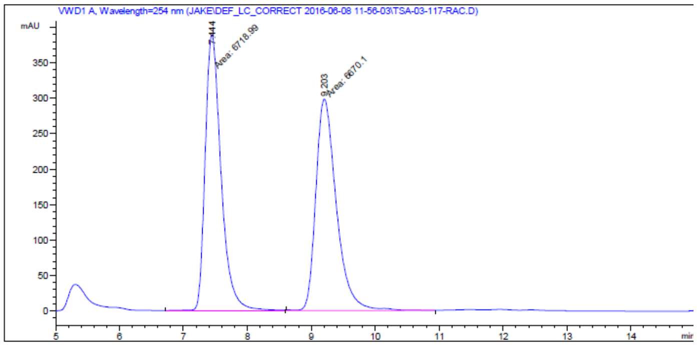

Peak #	RetTime [min]	Type	Width [min]	Area mAU *s	Height [mAU]	Area %
1	7.444	MM	0.2879	6718.99121	389.01019	50.1826
2	9.203	MM	0.3726	6670.10400	298.33868	49.8174

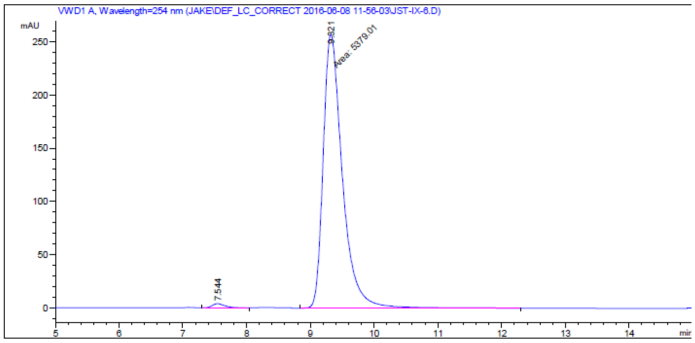

Peak #	RetTime [min]	Type	Width [min]	Area mAU *s	Height [mAU]	Area %
1	7.544	VV	0.2213	59.14909	4.01650	1.0877
2	9.321	MM	0.3485	5379.00684	257.26779	98.9123

Large-scale and recovery of ligand:
(*R*,*R*)-ProPhenol **L1** (61.8 mg, 0.0967 mmol), 1-benzosuberone (96.0 mg, 0.600 mmol), and activated 3 Å molecular sieves (15 mg) were measured into a microwave vial equipped with stir bar. The flask was placed under a nitrogen atmosphere and THF (0.9 mL) was added. After stirring for 15 minutes, diethylzinc (1.0 M in hexanes, 180μL, 0.180 mmol) was added and the reaction was allowed to stir for an additional 15 minutes. At this time, a premixed (15 min) THF (0.6 mL) solution of di-*tert*-butyl azodicarboxylate (150 mg, 0.651 mmol) and 3 Å molecular sieves (15 mg) was added. The reaction was allowed to stir at rt for 16 hours at which point silica gel was added to the reaction mixture. The reaction miture was concentrated under reduced pressure using rotary evaporation and the resulting silica gel was loaded directly onto a flash chromatography column (SiO2; petroleum ether / EtOAc 7.5:1 to 1.5:1 to 1:1) to afford 208 mg (89%) of a pale-yellow oil, and, separately, impure ligand. The impure ligand was dissolved in DCM (30 mL) and extracted with 10 mL of 0.5 M HSO_4Na (aqueous). The organic layer was disposed of and the aqueous layer was neutralized with 7.4 pH phosphate buffer. The neutral aqueous phase was extracted with DCM (3 x 30 mL). The combined organic layers were dried (Na_2SO_4), filtered, and concentrated to yield 51.1 mg (83%) of the (*R*,*R*)-ProPhenol **L1** ligand as a white foamy solid.

HPLC: (*Chiralpak® IB*, heptane-isopropanol 98:2, 0.8 mL/min, 254 nm): t_R(minor) = 7.2 min, t_R(major) = 8.8 min, 96% *ee*.

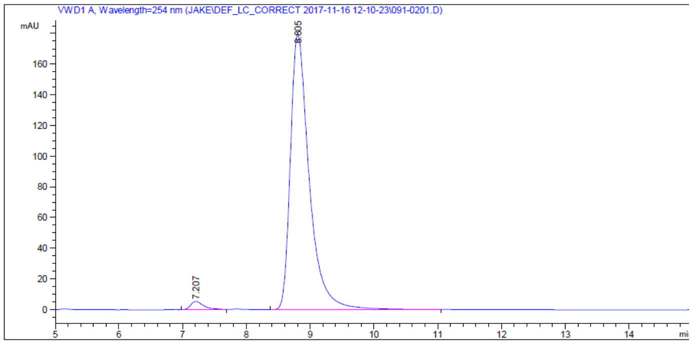

Peak #	RetTime [min]	Type	Width [min]	Area mAU *s	Height [mAU]	Area %
1	7.207	VV	0.2213	79.39741	5.39215	2.1061
2	8.805	VB	0.3098	3690.54614	179.43520	97.8939

The recovered ligand (20.6 mg, 0.0322 mmol), 1-benzosuberone (32.0mg, 0.200 mmol), and activated 3 Å molecular sieves (5 mg) were measured into a microwave vial equipped with stir bar. The flask was placed under a nitrogen atmosphere and THF (0.3 mL) was added. After stirring for 15 minutes, diethylzinc (1.0 M in hexanes, 60µL, 0.060 mmol) was added and the reaction was allowed to stir for an additional 15 minutes. At this time, a premixed (15 min) THF (0.2 mL) solution of di-*tert*-butyl azodicarboxylate (50.0 mg, 0.217 mmol) and 3 Å molecular sieves (5 mg) was added. The reaction was allowed to stir at rt for 13 hours at which point the reaction mixture was filtered through a small plug of fluorosil. The residue was concentrated under reduced pressure using rotary evaporation. The resulting residue was purified via flash chromatography (SiO2; petroleum ether / EtOAc 6:1) to afford 65.5 mg (84%) of a pale-yellow oil.

HPLC: (*Chiralpak® IB*, heptane-isopropanol 98:2, 0.8 mL/min, 254 nm): t_R(minor) = 7.2 min, t_R(major) = 8.8 min, 97% *ee*.

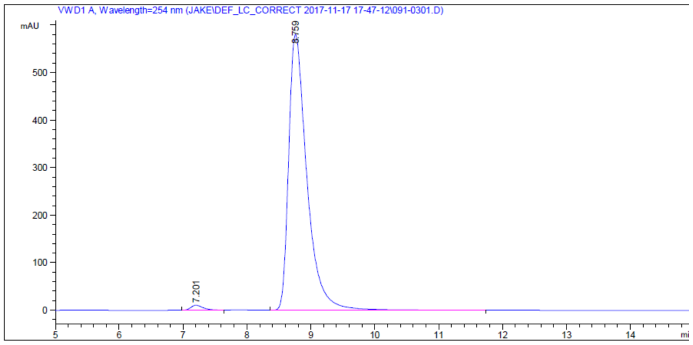

Peak #	RetTime [min]	Type	Width [min]	Area mAU *s	Height [mAU]	Area %
1	7.201	VV	0.2124	153.08322	10.77259	1.3103
2	8.759	VV	0.2989	1.15301e4	579.99469	98.6897

<u>di-tert-butyl 1-(4-methoxy-1-oxo-2,3-dihydro-1H-inden-2-yl)hydrazine-1,2-dicarboxylate</u> **2t**

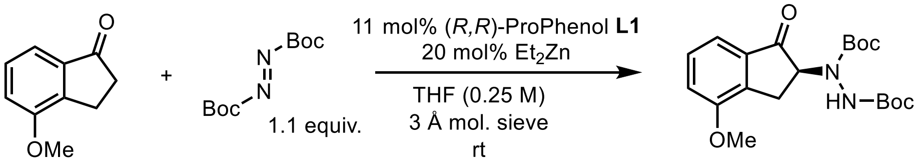

(*R*,*R*)-ProPhenol **L1** (13.7 mg, 0.0214 mmol), 4-methoxy-1-indanone (32.4 mg, 0.200 mmol), and activated 3 Å molecular sieves (5 mg) were measured into a microwave vial equipped with stir bar. The flask was placed under a nitrogen atmosphere and THF (0.5 mL) was added. After stirring for 15 minutes, diethylzinc (1.0 M in hexanes, 40µL, 0.040 mmol) was added and the reaction was allowed to stir for an additional 15 minutes. At this time, a premixed (15 min) THF (0.3 mL) solution of di-*tert*-butyl azodicarboxylate (50.7 mg, 0.220 mmol) and 3 Å molecular sieves (5 mg) was added. The reaction was allowed to stir at rt for 2 h at which point the reaction mixture was filtered through a small plug of fluorosil. The residue was concentrated under reduced pressure using rotary evaporation. The resulting

residue was purified rapidly via flash chromatography (SiO2; DCM to petroleum ether / EtOAc 5:1 to petroleum ether / EtOAc 3:1) to afford 40.9 mg (52%) of a white solid (m.p. 76-78 °C) as well as 12.0 mg of the starting indanone (37%).

IR($CDCl_3$) 3303, 2978, 2934, 2280, 1730, 1603, 1490, 1393, 1368, 1262, 1155, 1078, 1013, 936, 895, 845, 814, 768 cm^{-1}. ^1H NMR (500 MHz, 75 °C, Benzene-d_6) δ 7.36 (d, J = 7.6 Hz, 1H), 6.96 (t, J = 7.8 Hz, 1H), 6.55 (d, J = 8.0 Hz, 1H), 6.37 (s, 1H), 4.69 (s, 1H), 3.47 – 3.39 (m, 2H), 3.31 (s, 3H), 1.36 – 1.34 (m, 9H), 1.33 (s, 9H). ^{13}C NMR (126 MHz, 75 °C, Benzene) δ 200.92, 157.56, 155.90, 154.90, 141.21, 137.44, 128.97, 116.11, 115.78, 81.71, 80.78, 65.89, 55.19, 28.30, 28.27. HRMS (EI) calculated for $C_{20}H_{28}N_2O_6$ (Na^+) 415.1840, found: 415.1839. R_f (petroleum ether / EtOAc 4:1) = 0.27. $[\alpha]_D^{23}$ = 4.8 (c = 0.28, $CHCl_3$). HPLC: (*Chiralpak® IA*, heptane-isopropanol 95:5, 0.8 mL/min, 254 nm): t_R(minor) = 35.0 min, t_R(major) = 24.2 min, 89% *ee*.

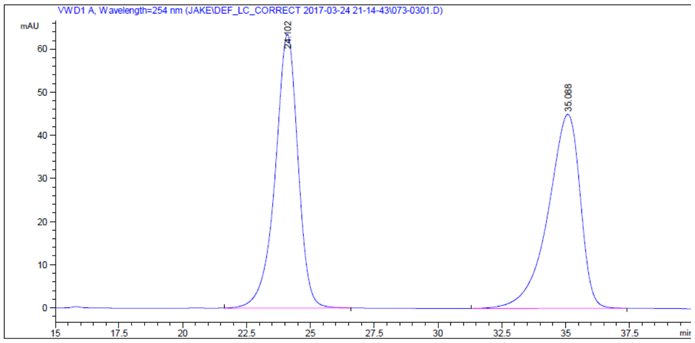

Peak #	RetTime [min]	Type	Width [min]	Area mAU *s	Height [mAU]	Area %
1	24.102	BB	0.9396	3954.82471	63.46221	50.0740
2	35.088	BB	1.3371	3943.13135	44.98711	49.9260

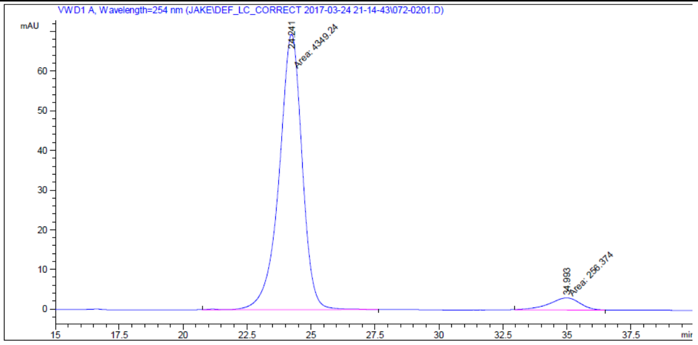

Peak #	RetTime [min]	Type	Width [min]	Area mAU *s	Height [mAU]	Area %
1	24.241	MM	1.0444	4349.24463	69.40398	94.4334
2	34.993	MM	1.4005	256.37424	3.05109	5.5666

<u>di-tert-butyl 1-(1-oxo-2,3-dihydro-1H-inden-2-yl)hydrazine-1,2-dicarboxylate</u> **2u**

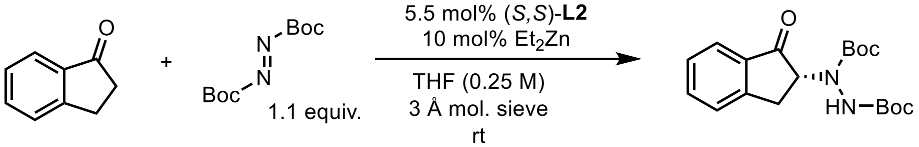

(*S*,*S*)-**L2** (8.5 mg, 0.011 mmol), 1-indanone (26.4 mg, 0.200 mmol), and activated 3 Å molecular sieves (5 mg) were measured into a microwave vial equipped with stir bar. The flask was placed under a nitrogen atmosphere and THF (0.5 mL) was added. After stirring for 15 minutes, diethylzinc (1.0 M in hexanes, 20µL, 0.020 mmol) was added and the reaction was allowed to stir for an additional 15 minutes. At this time, a premixed (15 min) THF (0.3 mL) solution of di-*tert*-butyl azodicarboxylate (50.7 mg, 0.220 mmol) and 3 Å molecular sieves (5 mg) was added. The reaction was allowed to stir at rt for 2.5 h at which point the reaction mixture was filtered through a small plug of fluorosil. The residue was concentrated under reduced pressure using rotary evaporation. The resulting residue was purified rapidly via flash chromatography (SiO2; DCM to petroleum ether / EtOAc 6:1 to petroleum ether / EtOAc 5:1 to

petroleum ether / EtOAc 4:1) to afford 49.7 mg (69 %) of a clear oil as well as 6.3 mg (24%) of the staring indanone.

IR($CDCl_3$) 3297, 2978, 2930, 1729, 1612, 1475, 1394, 1368, 1301, 1247, 1155, 760 cm^{-1}. ^{1}H NMR (500 MHz, 75 °C, Benzene-d_6) δ 7.63 (s, 1H), 7.10 (t, J = 7.5 Hz, 1H), 6.97 – 6.87 (m, 2H), 6.34 (s, 1H), 4.70 (s, 1H), 3.41 (dd, J = 17.1, 5.6 Hz, 1H), 3.19 (dd, J = 17.0, 8.4 Hz, 1H), 1.37 (s, 9H), 1.34 (s, 9H). ^{13}C NMR (126 MHz, 75 °C, Benzene) δ 200.75, 155.95, 154.92, 151.99, 135.96, 134.80, 127.53, 126.73, 124.25, 81.76, 80.80, 65.87 (broad), 31.61, 28.30, 28.27. HRMS (EI) calculated for $C_{19}H_{26}N_2O_5$ (Na^+) 385.1734, found: 385.1734. R_f (petroleum ether / EtOAc 4:1) = 0.26. $[\alpha]_D^{23}$ = 28.6 (c = 1.25, $CHCl_3$). HPLC: (*Chiralpak® IA*, heptane-isopropanol 90:10, 0.8 mL/min, 254 nm): t_R(minor) = 9.2 min, t_R(major) = 14.8 min, 86% *ee*.

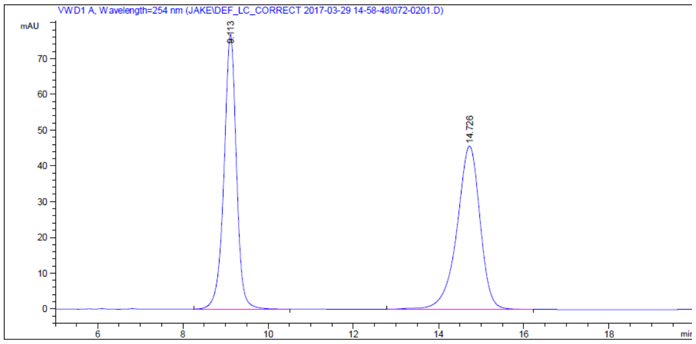

Peak #	RetTime [min]	Type	Width [min]	Area mAU *s	Height [mAU]	Area %
1	9.113	BB	0.3111	1567.45715	76.75031	47.6020
2	14.726	BB	0.5718	1725.37964	45.66551	52.3980

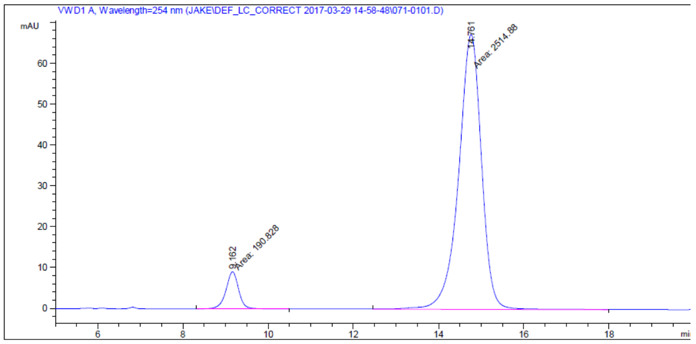

Peak #	RetTime [min]	Type	Width [min]	Area mAU *s	Height [mAU]	Area %
1	9.162	MM	0.3490	190.82840	9.11372	7.0528
2	14.761	MM	0.6226	2514.88477	67.32288	92.9472

III. References

1. T. Takeda and M. Terada, *J. Am. Chem. Soc.*, 2013, **135**, 15306-15309.
2. S. Mouri, Z. Chen, H. Mitsunuma, M. Furutachi, S. Matsunaga and M. Shibasaki, *J. Am. Chem. Soc.*, 2010, **132**, 1255-1257.
3. P. Gmeiner and B. Bollinger, *Liebigs Ann. Chem.*, 1992, *273-278*.
4. Y. Wei, H.-X. Zhang, J.-L. Zeng, J. Nie and J.-A. Ma, *Org. Lett.*, 2017, **19**, 2162-2165.

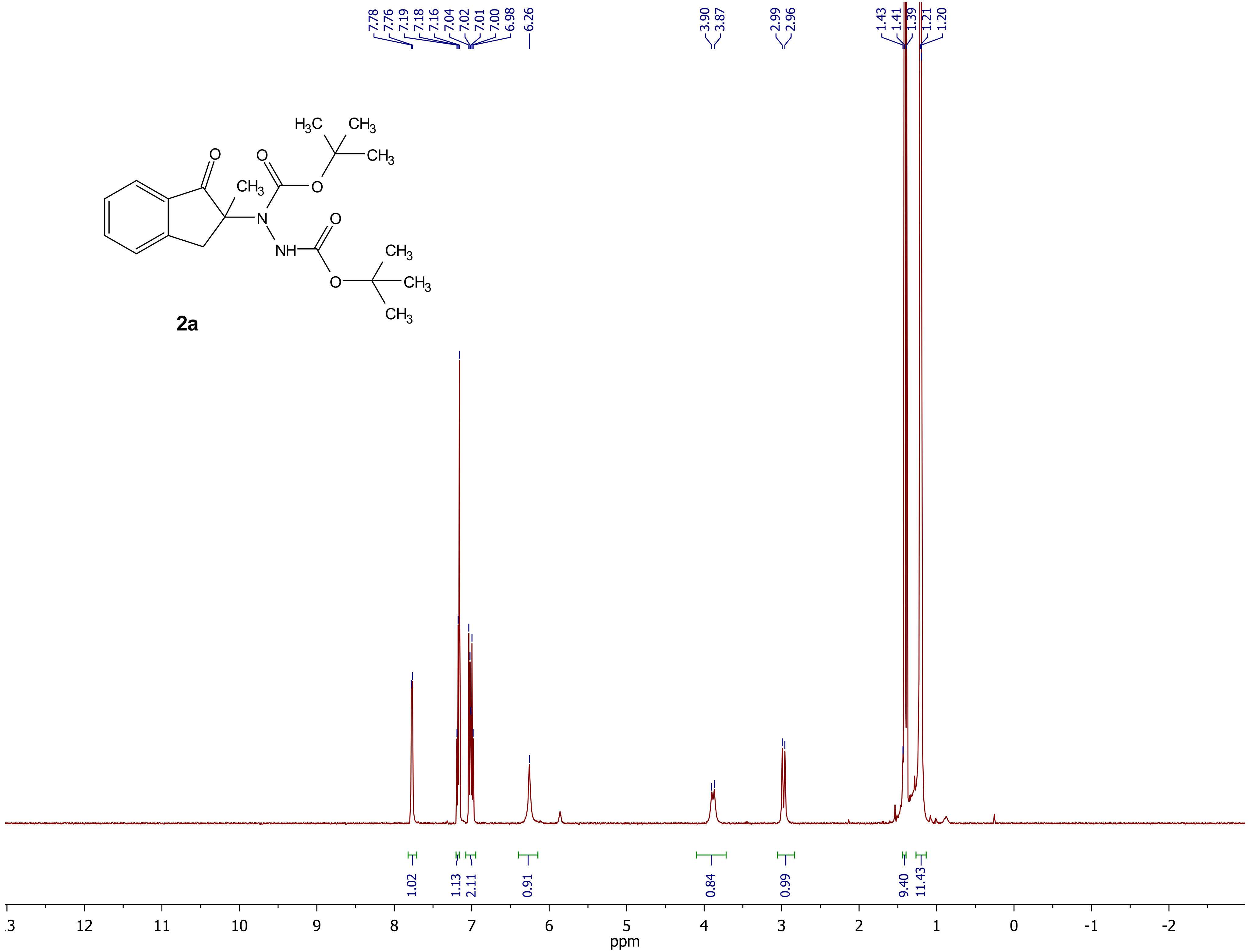
7.78
7.76
7.19
7.18
7.16
7.04
7.02
7.01
7.00
6.98
6.26
3.90
3.87
2.99
2.96
1.43
1.41
1.39
1.21
1.20
2a
1.02
1.13
2.11
0.91
0.84
0.99
9.40
11.43
ppm

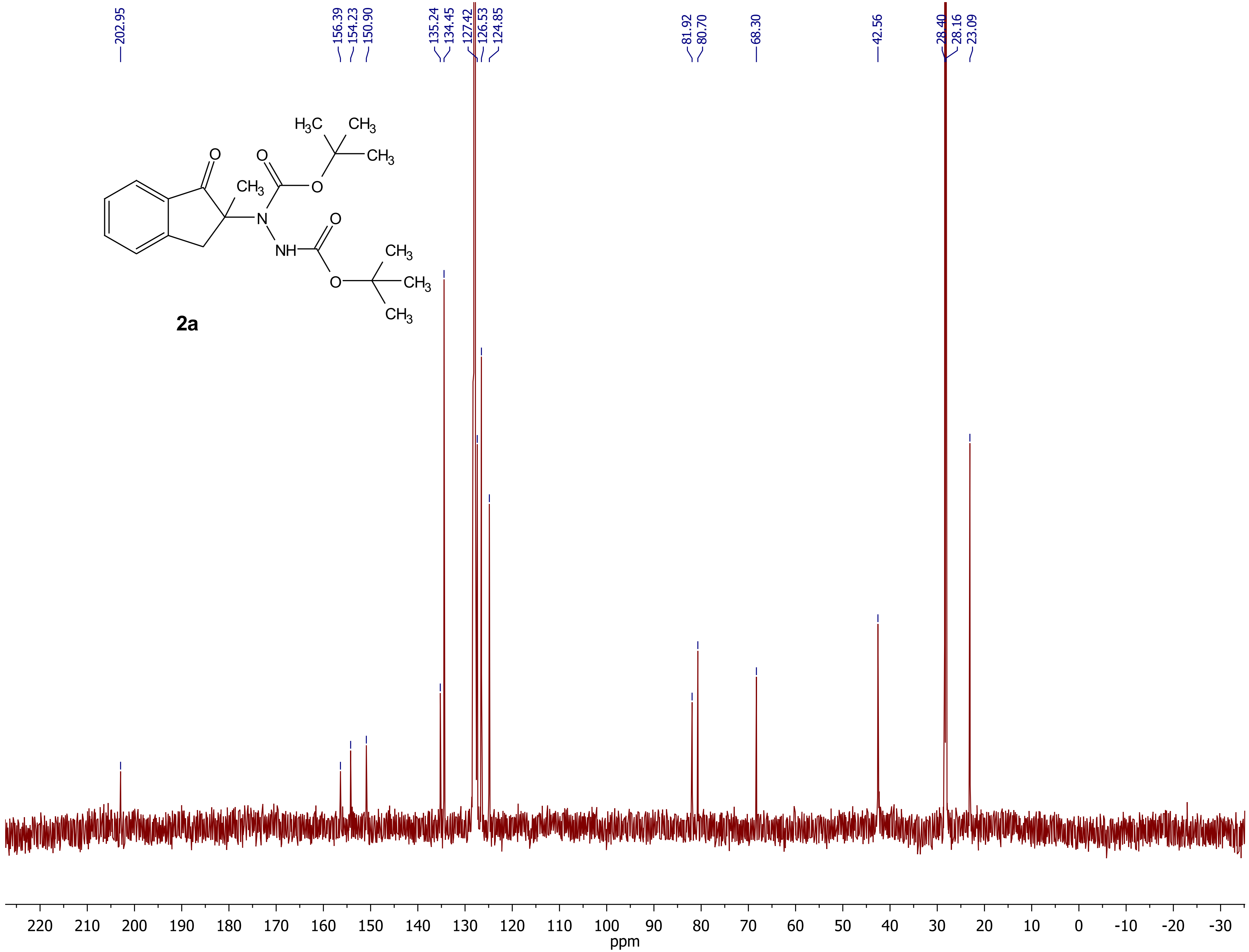
202.95
156.39
154.23
150.90
135.24
134.45
127.42
126.53
124.85
81.92
80.70
68.30
42.56
28.40
28.16
23.09
2a
220 210 200 190 180 170 160 150 140 130 120 110 100 90 80 70 60 50 40 30 20 10 0 -10 -20 -30
ppm

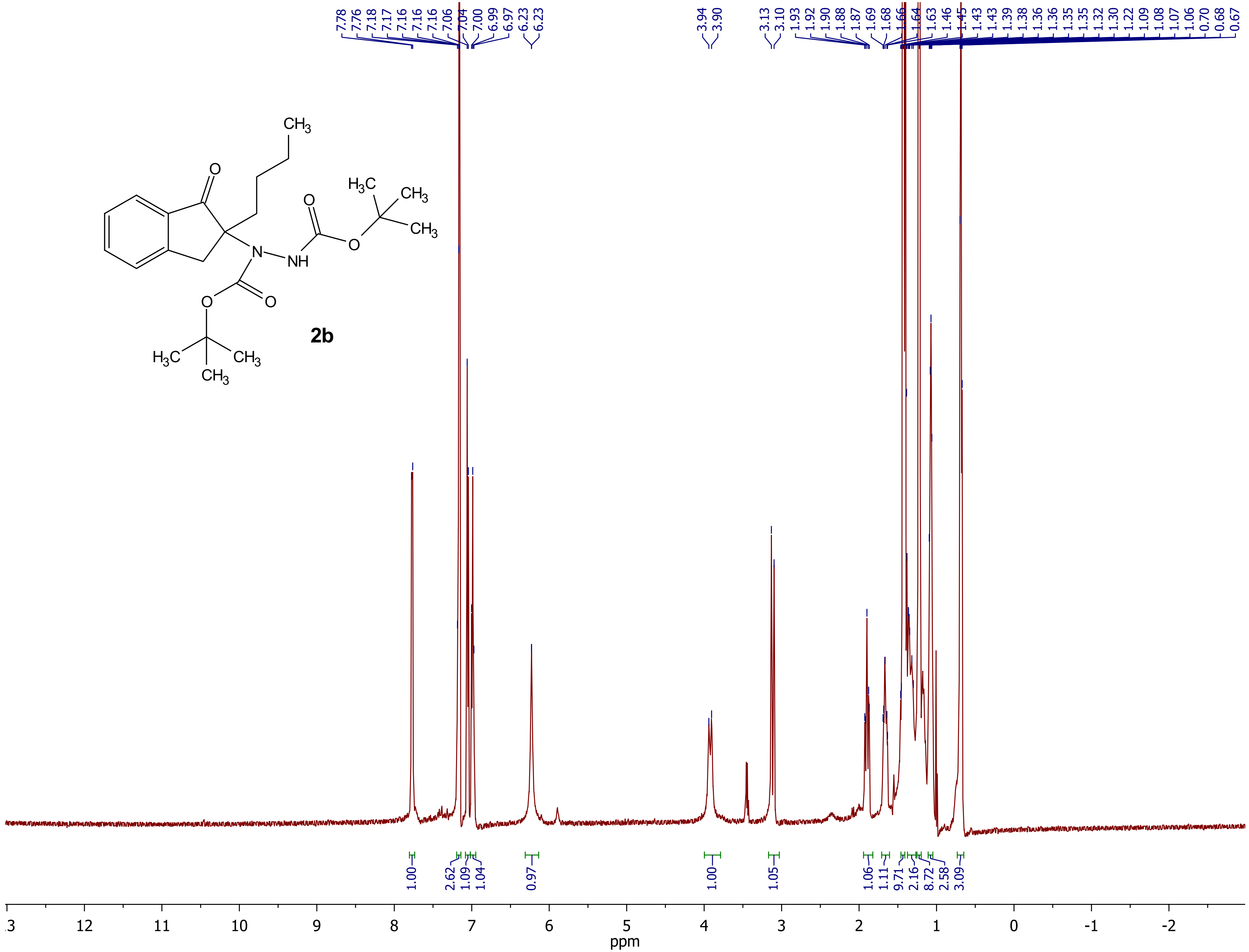
7.78
7.76
7.18
7.17
7.16
7.16
7.16
7.06
7.04
7.00
6.99
6.97
6.23
6.23
3.94
3.90
3.13
3.10
1.93
1.92
1.90
1.88
1.87
1.69
1.68
1.66
1.64
1.63
1.46
1.45
1.43
1.43
1.39
1.38
1.36
1.36
1.35
1.35
1.32
1.30
1.22
1.09
1.08
1.07
1.06
0.70
0.68
0.67
CH3
O
H3C
CH3
O
CH3
N
NH
O
O
O
H3C
CH3
CH3
2b
1.00
2.62
1.09
1.04
0.97
1.00
1.05
1.06
1.11
9.71
2.16
8.72
2.58
3.09
13
12
11
10
9
8
7
6
5
4
3
2
1
0
-1
-2
ppm

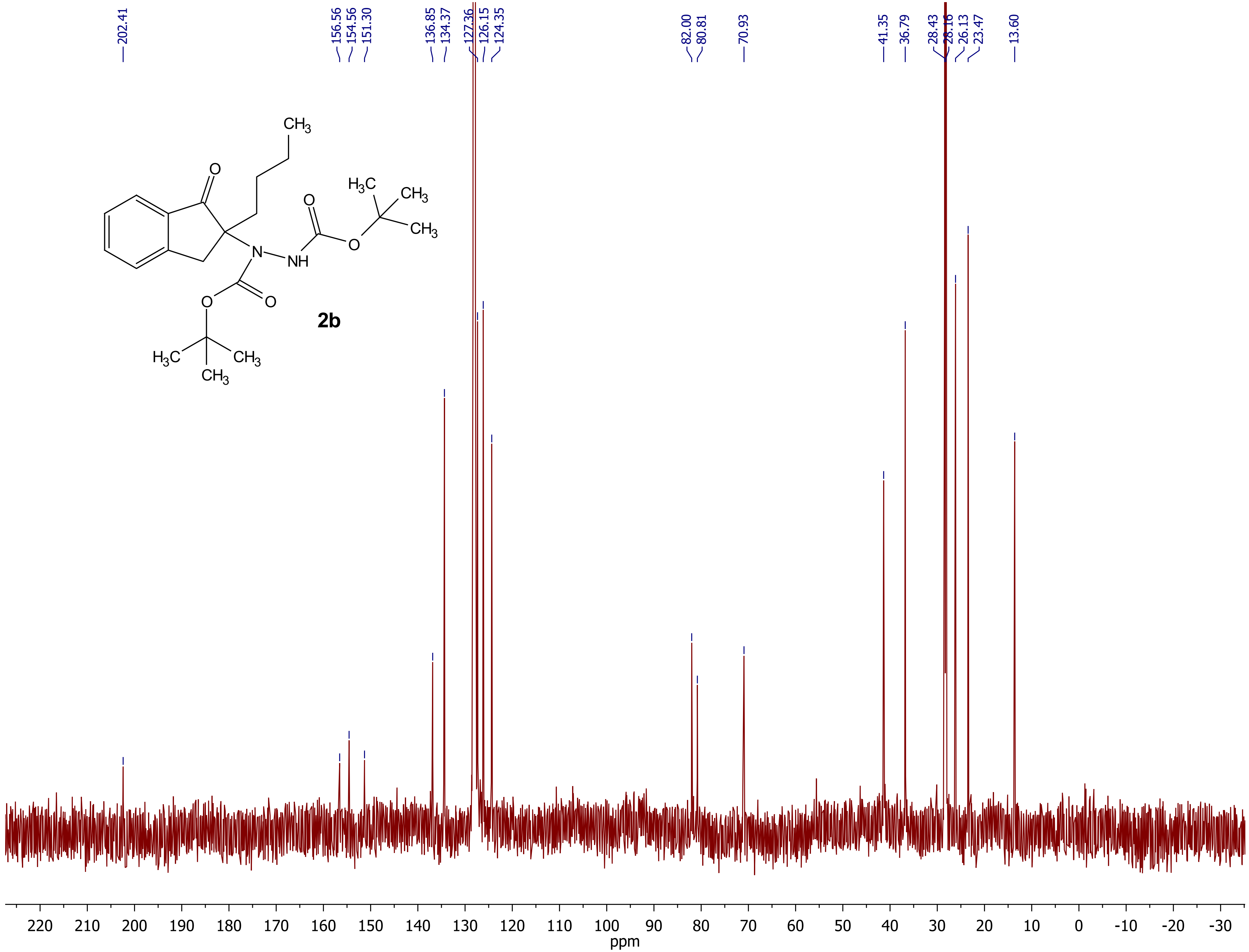
202.41
156.56
154.56
151.30
136.85
134.37
127.36
126.15
124.35
82.00
80.81
70.93
41.35
36.79
28.43
28.16
26.13
23.47
13.60
2b
220 210 200 190 180 170 160 150 140 130 120 110 100 90 80 70 60 50 40 30 20 10 0 -10 -20 -30
ppm

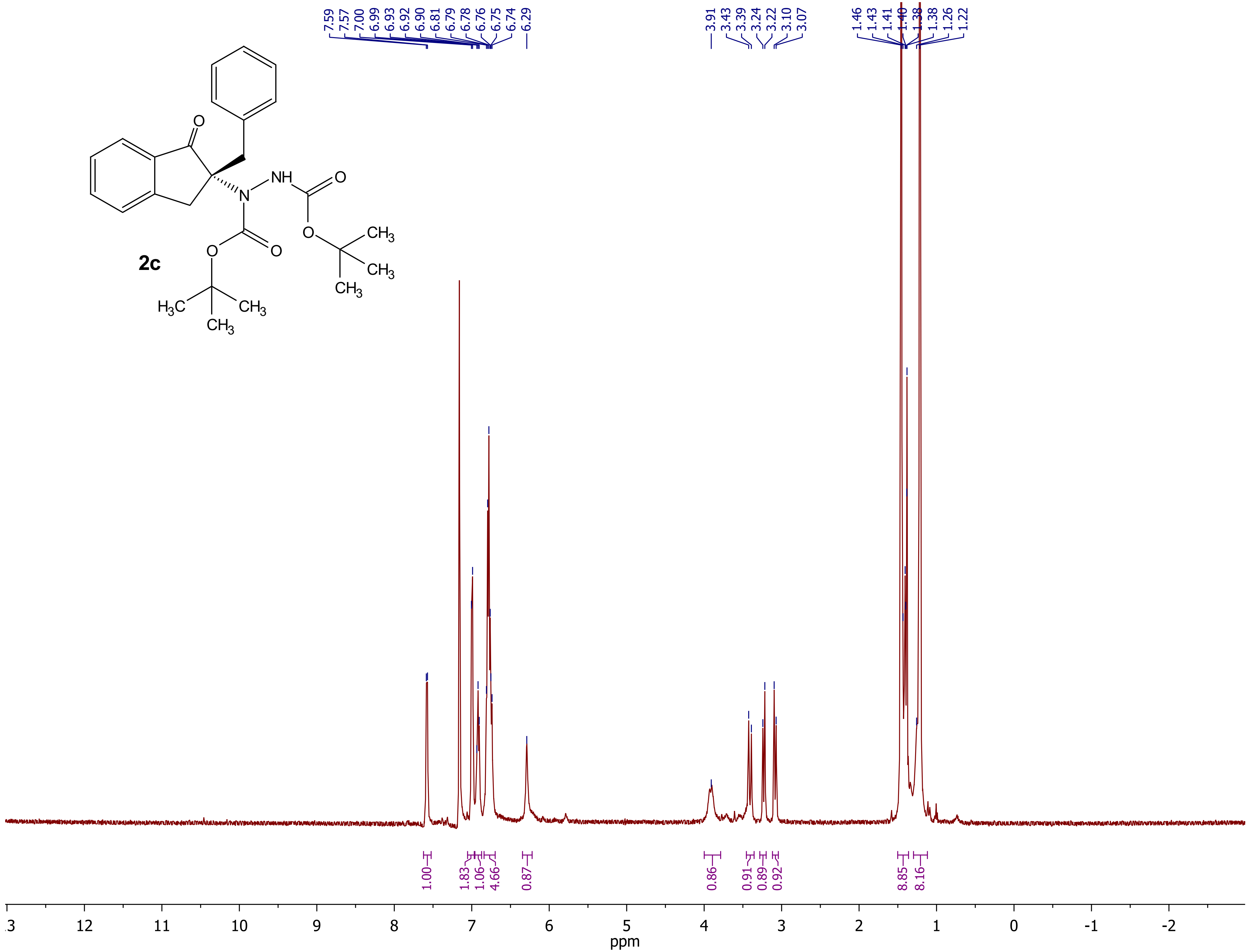
7.59
7.57
7.00
6.99
6.93
6.92
6.90
6.81
6.79
6.78
6.76
6.75
6.74
6.29
3.91
3.43
3.39
3.24
3.22
3.10
3.07
1.46
1.43
1.41
1.40
1.38
1.38
1.26
1.22
O
NH
O
N
O
CH3
O
O
2c
CH3
CH3
H3C
CH3
CH3
CH3
1.00
1.83
1.06
4.66
0.87
0.86
0.91
0.89
0.92
8.85
8.16
12
11
10
9
8
7
6
5
4
3
2
1
0
-1
-2
ppm

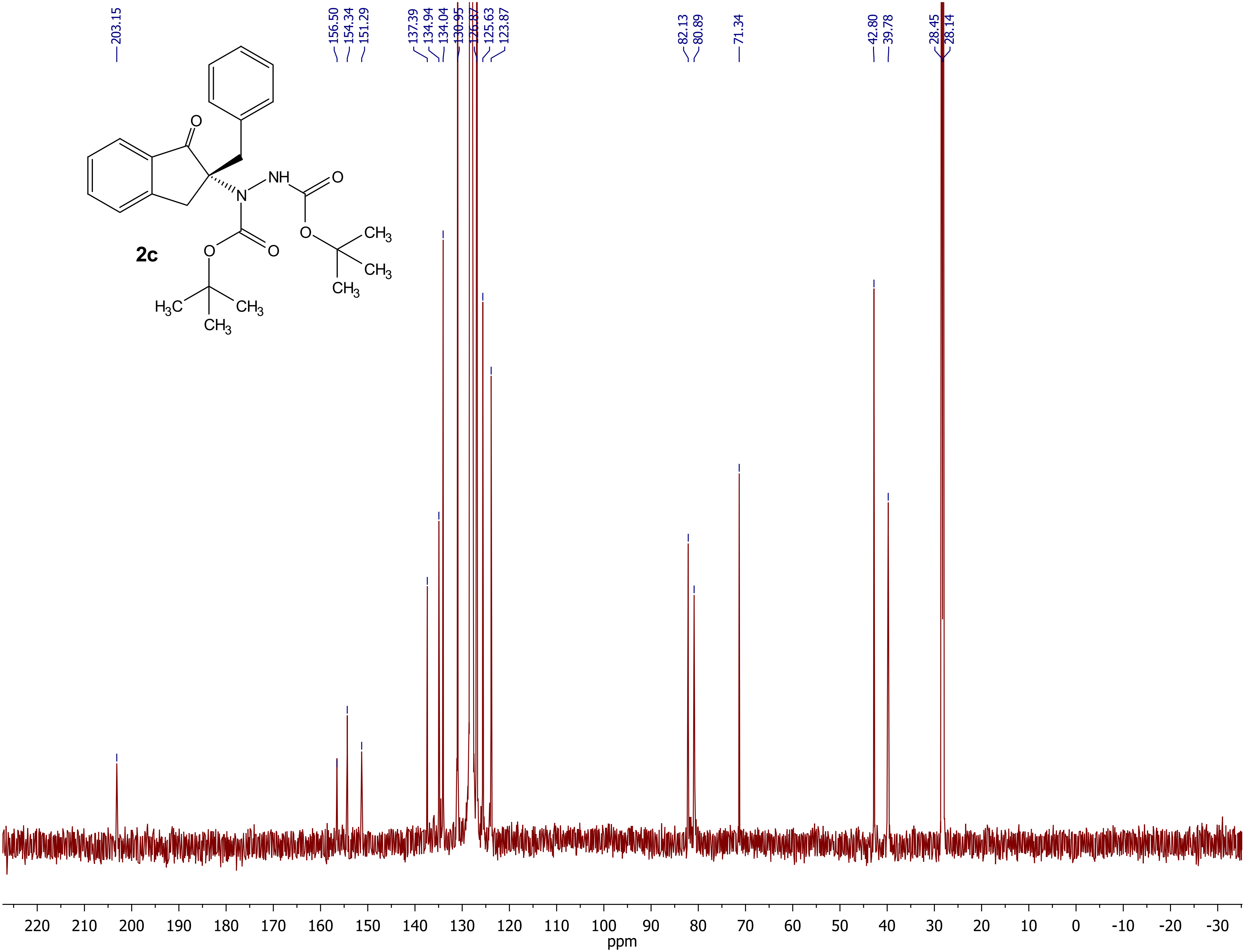

203.15
156.50
154.34
151.29
137.39
134.94
134.04
130.95
126.87
125.63
123.87
82.13
80.89
71.34
42.80
39.78
28.45
28.14
2c
O
NH
N
O
O
O
O
CH3
CH3
CH3
H3C
CH3
CH3
220 210 200 190 180 170 160 150 140 130 120 110 100 90 80 70 60 50 40 30 20 10 0 -10 -20 -30
ppm

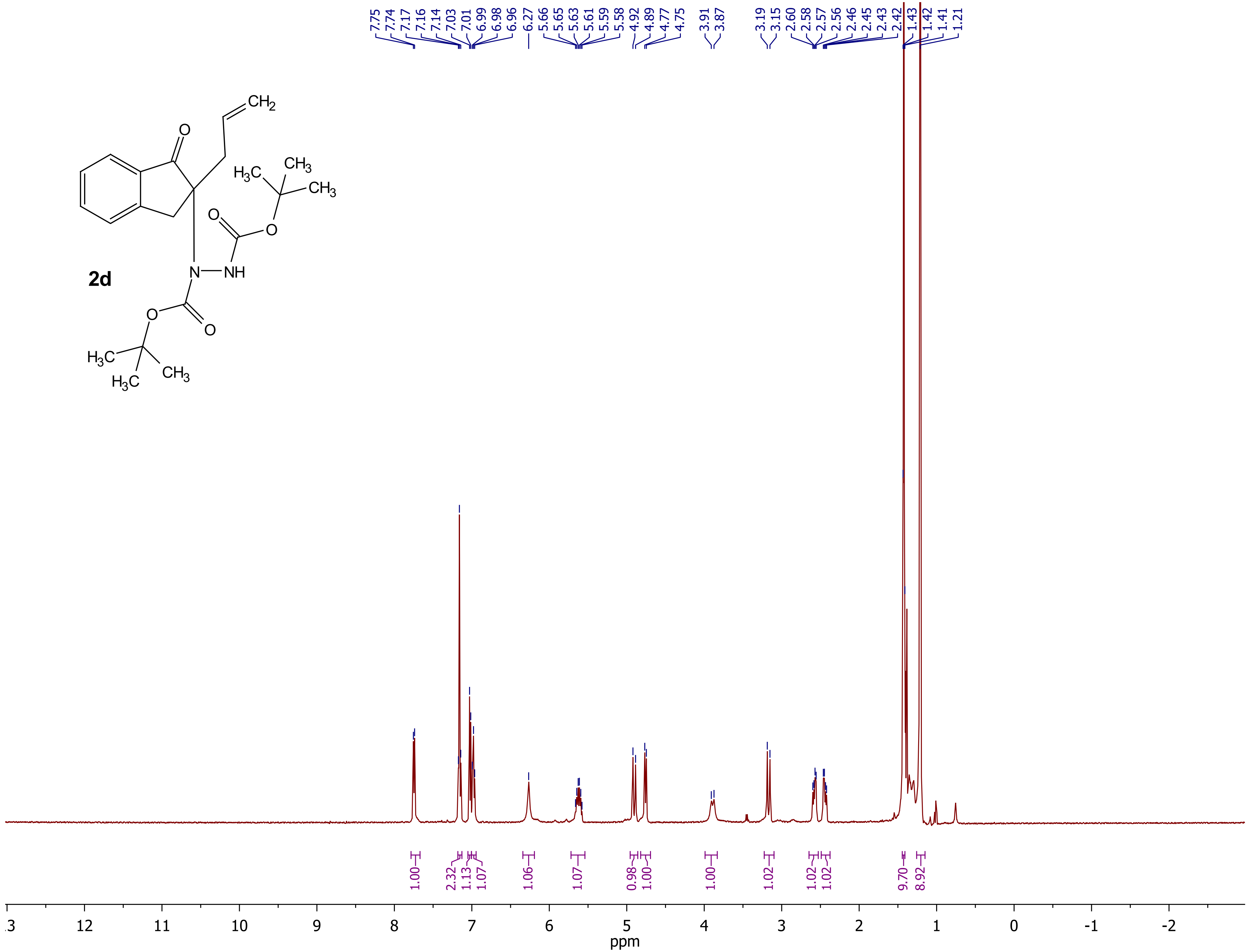
7.75
7.74
7.17
7.16
7.14
7.03
7.01
6.99
6.98
6.96
6.27
5.66
5.65
5.63
5.61
5.59
5.58
4.92
4.89
4.77
4.75
3.91
3.87
3.19
3.15
2.60
2.58
2.57
2.56
2.46
2.45
2.43
2.42
1.43
1.42
1.41
1.21
CH2
O
H3C
CH3
CH3
O
O
N
NH
O
O
H3C
H3C
CH3
2d
1.00
2.32
1.13
1.07
1.06
1.07
0.98
1.00
1.00
1.02
1.02
1.02
9.70
8.92
ppm

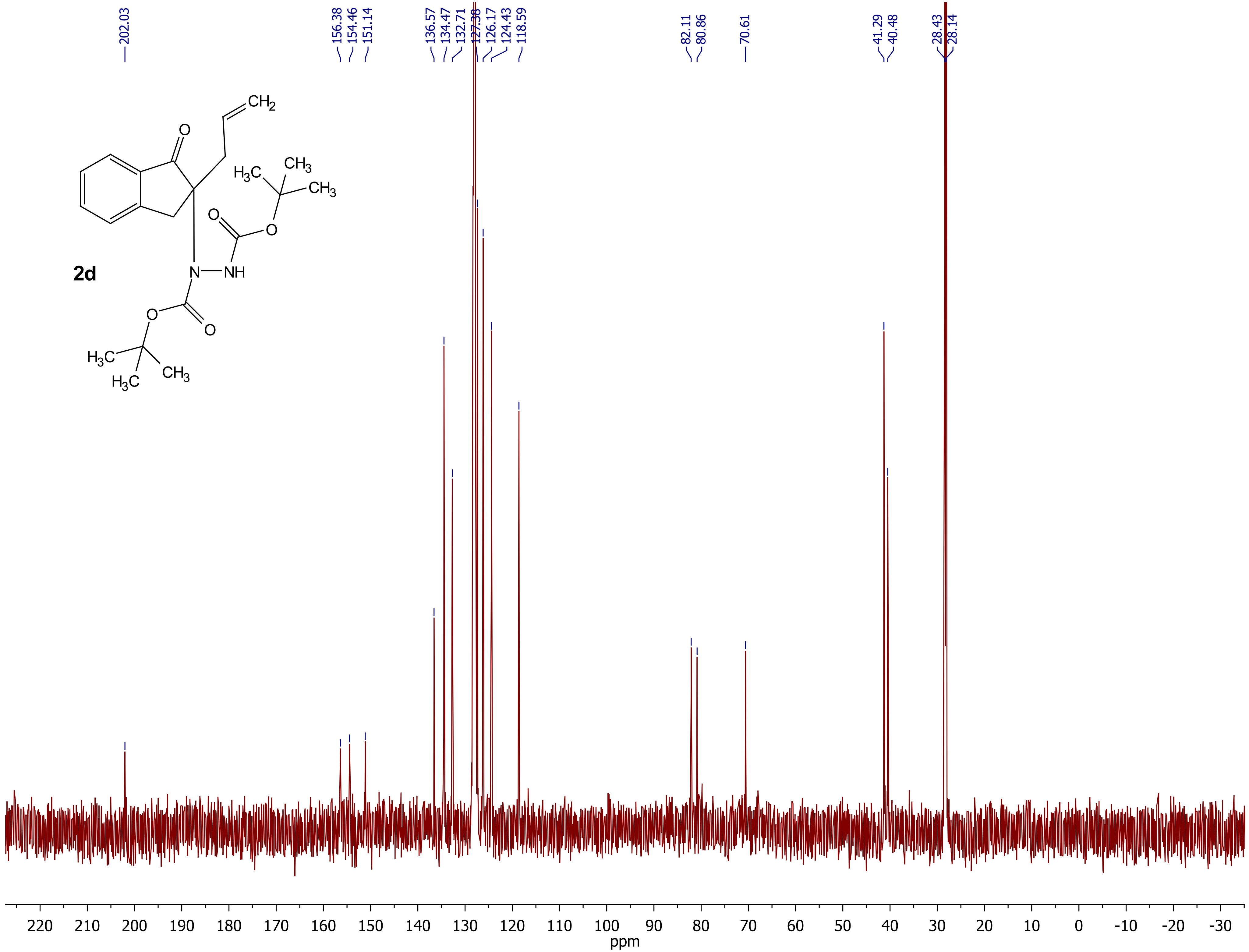
202.03
156.38
154.46
151.14
136.57
134.47
132.71
127.38
126.17
124.43
118.59
82.11
80.86
70.61
41.29
40.48
28.43
28.14
2d
O
CH2
H3C
CH3
CH3
O
O
N
NH
O
O
H3C
H3C
CH3
220 210 200 190 180 170 160 150 140 130 120 110 100 90 80 70 60 50 40 30 20 10 0 -10 -20 -30
ppm

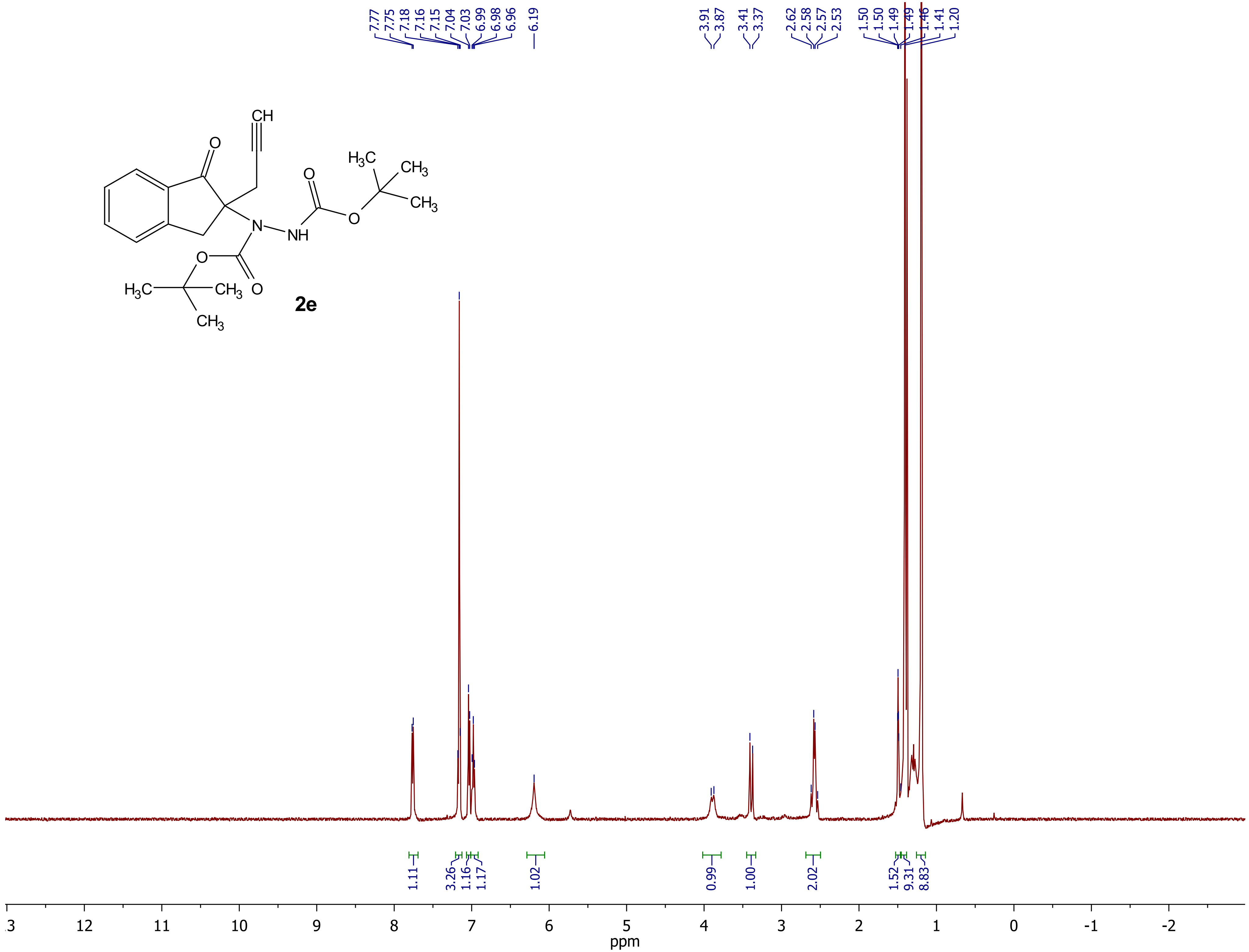
7.77 7.75 7.18 7.16 7.15 7.04 7.03 6.99 6.98 6.96
6.19
3.91 3.87 3.41 3.37
2.62 2.58 2.57 2.53
1.50 1.50 1.49 1.49 1.46 1.41 1.20
CH
O
O
H3C
CH3
CH3
N
NH
O
O
H3C
CH3
O
CH3
2e
1.11 3.26 1.16 1.17 1.02 0.99 1.00 2.02 1.52 9.31 8.83
13 12 11 10 9 8 7 6 5 4 3 2 1 0 -1 -2
ppm

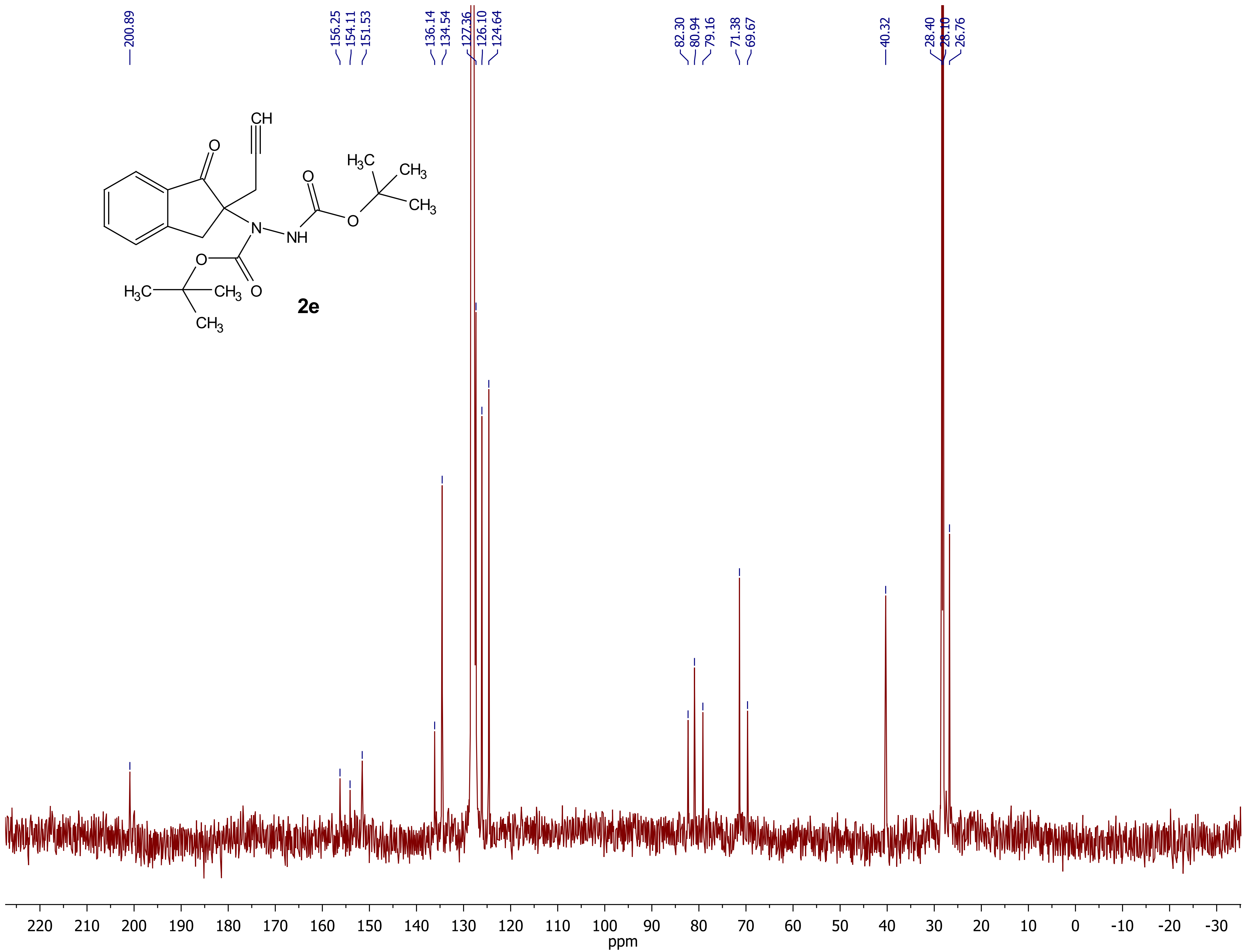
200.89
156.25
154.11
151.53
136.14
134.54
127.36
126.10
124.64
82.30
80.94
79.16
71.38
69.67
40.32
28.40
28.10
26.76
CH
O
H3C
CH3
O
CH3
O
N
NH
O
H3C
CH3
O
CH3
2e
220 210 200 190 180 170 160 150 140 130 120 110 100 90 80 70 60 50 40 30 20 10 0 -10 -20 -30
ppm

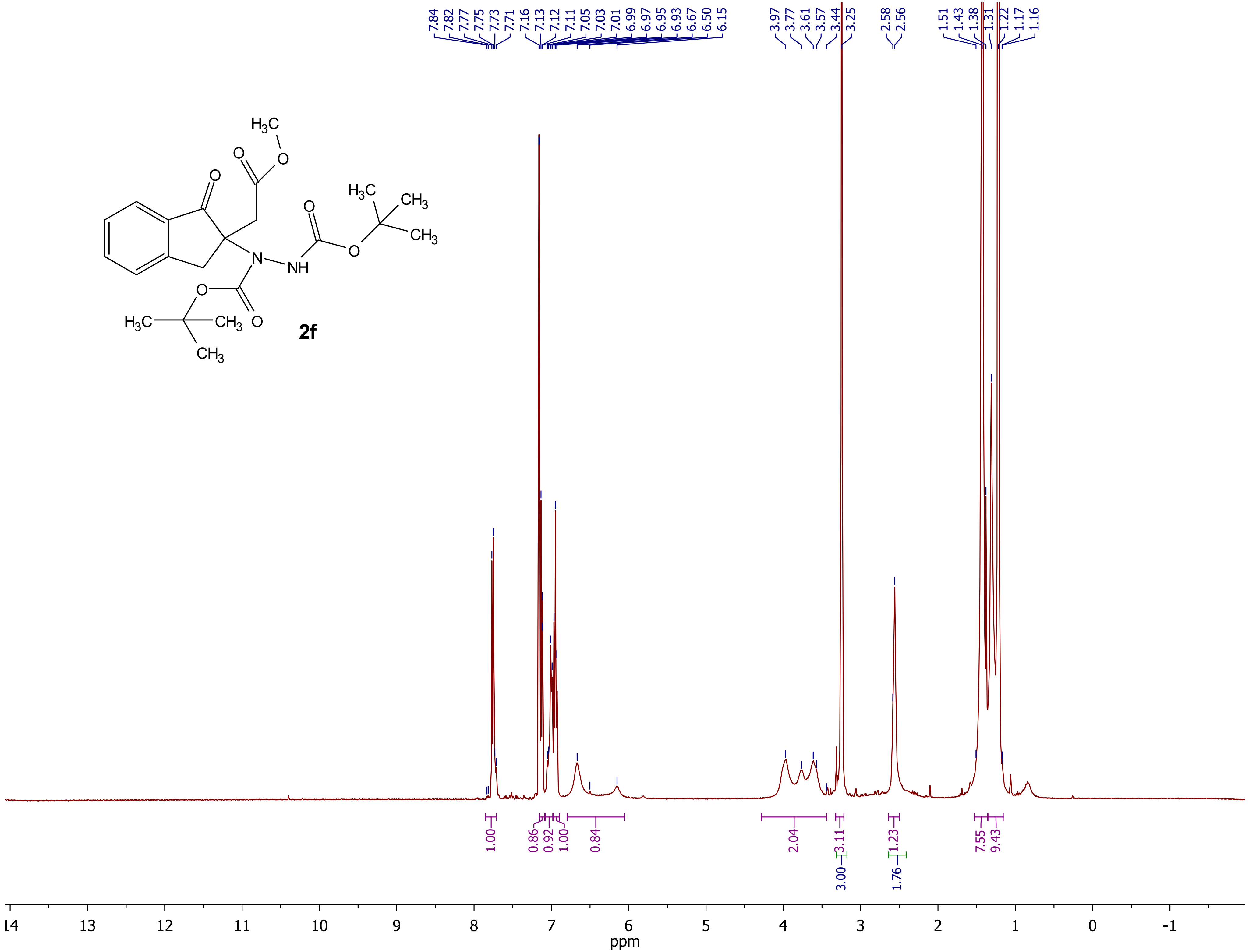

7.84 7.82 7.77 7.75 7.73 7.71 7.16 7.13 7.12 7.11 7.05 7.03 7.01 6.99 6.97 6.95 6.93 6.67 6.50 6.15
3.97 3.77 3.61 3.57 3.44 3.25
2.58 2.56
1.51 1.43 1.38 1.31 1.22 1.17 1.16
1.00 0.86 0.92 1.00 0.84 2.04 3.11 3.00 1.23 1.76 7.55 9.43
H3C
O
O
O
N
NH
H3C
CH3
CH3
O
O
O
H3C
CH3
CH3
O
2f
14 13 12 11 10 9 8 7 6 5 4 3 2 1 0 -1
ppm

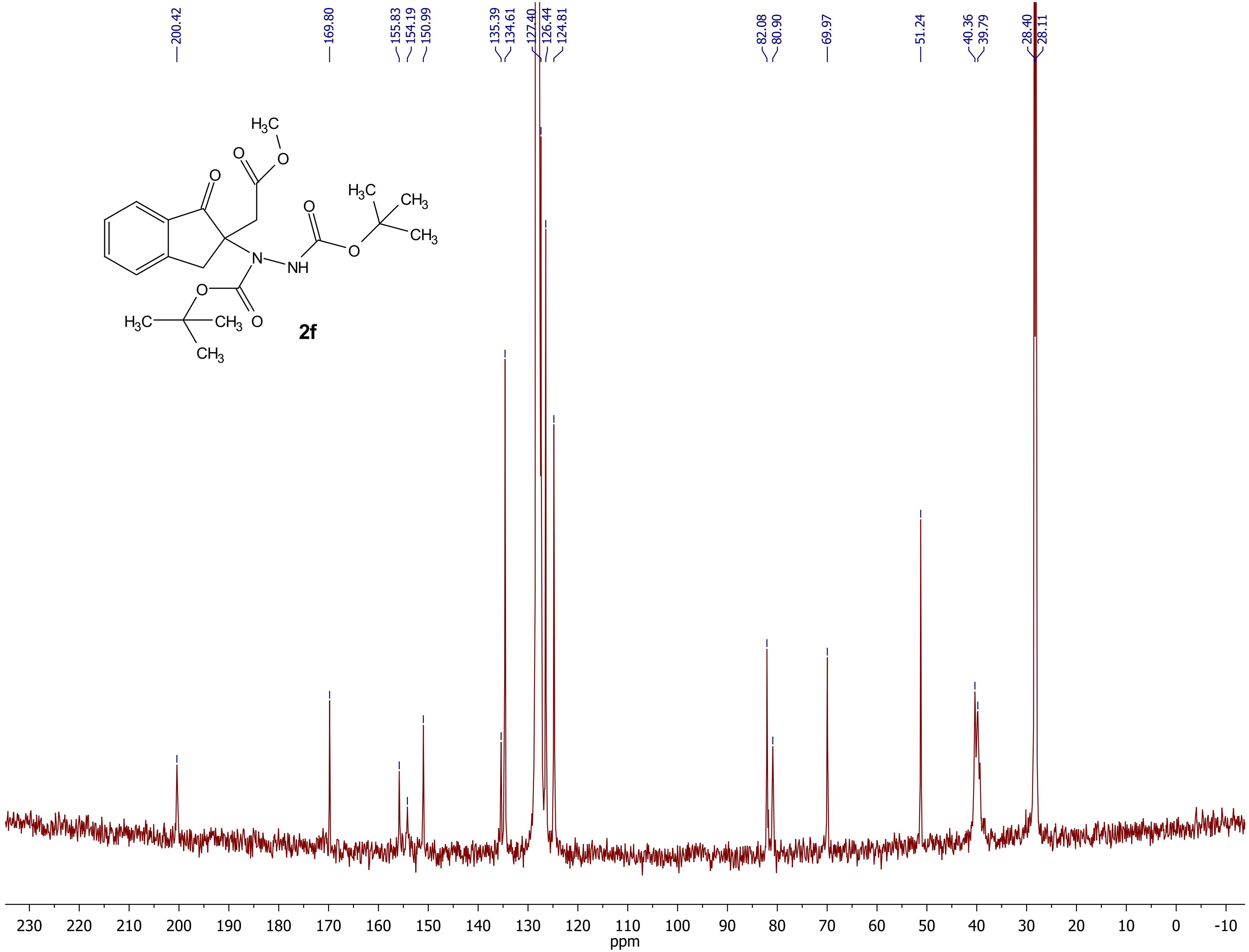
200.42
169.80
155.83
154.19
150.99
135.39
134.61
127.40
126.44
124.81
82.08
80.90
69.97
51.24
40.36
39.79
28.40
28.11
2f
ppm

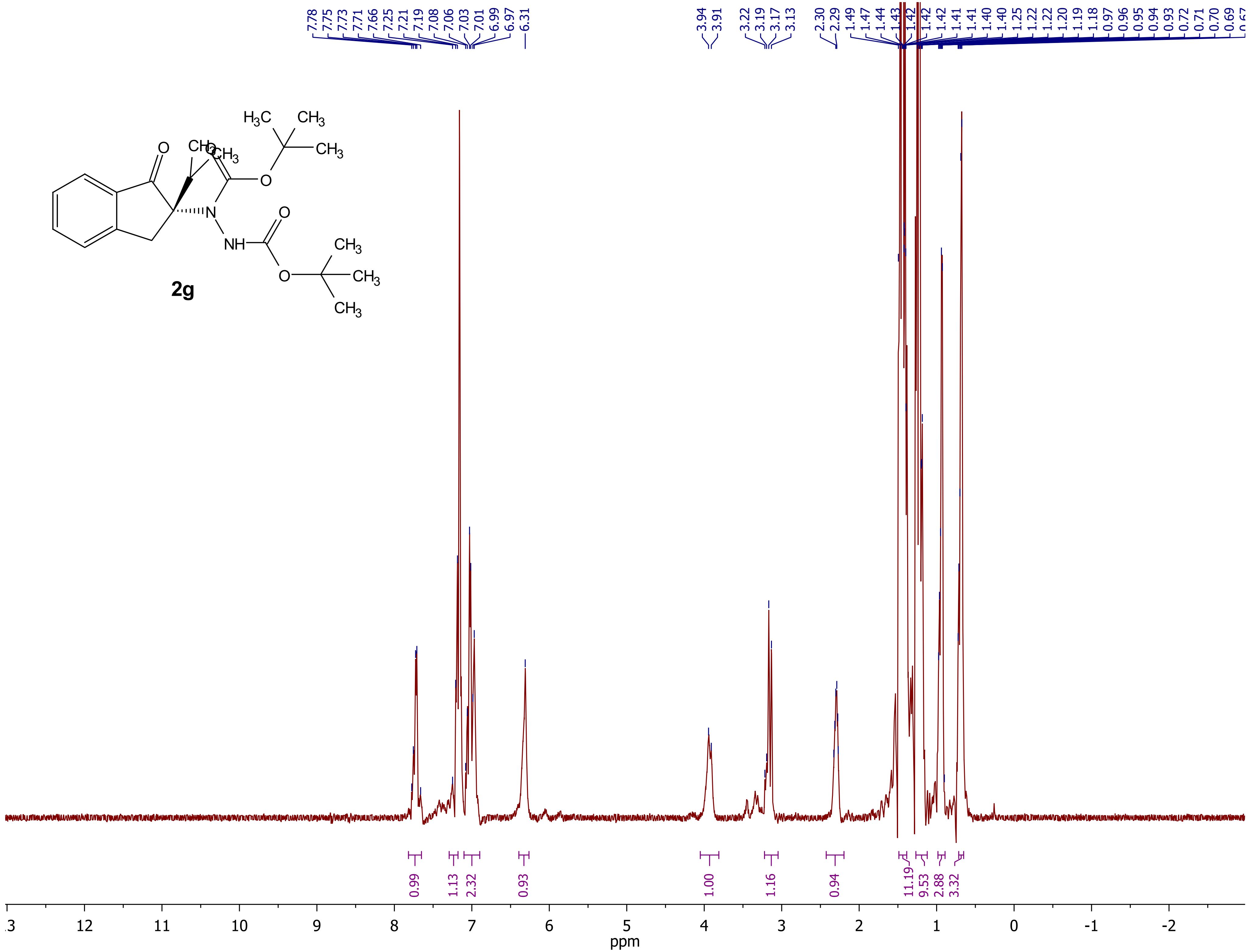

7.78 7.75 7.73 7.71 7.66 7.25 7.21 7.19 7.08 7.06 7.03 7.01 6.99 6.97 6.31
3.94 3.91
3.22 3.19 3.17 3.13
2.30 2.29
1.49 1.47 1.44 1.43 1.42 1.42 1.42 1.41 1.41 1.40 1.40 1.25 1.22 1.22 1.20 1.19 1.18 0.97 0.96 0.95 0.94 0.93 0.72 0.71 0.70 0.69
2g
0.99 1.13 2.32 0.93 1.00 1.16 0.94 11.19 9.53 2.88 3.32
13 12 11 10 9 8 7 6 5 4 3 2 1 0 -1 -2
ppm

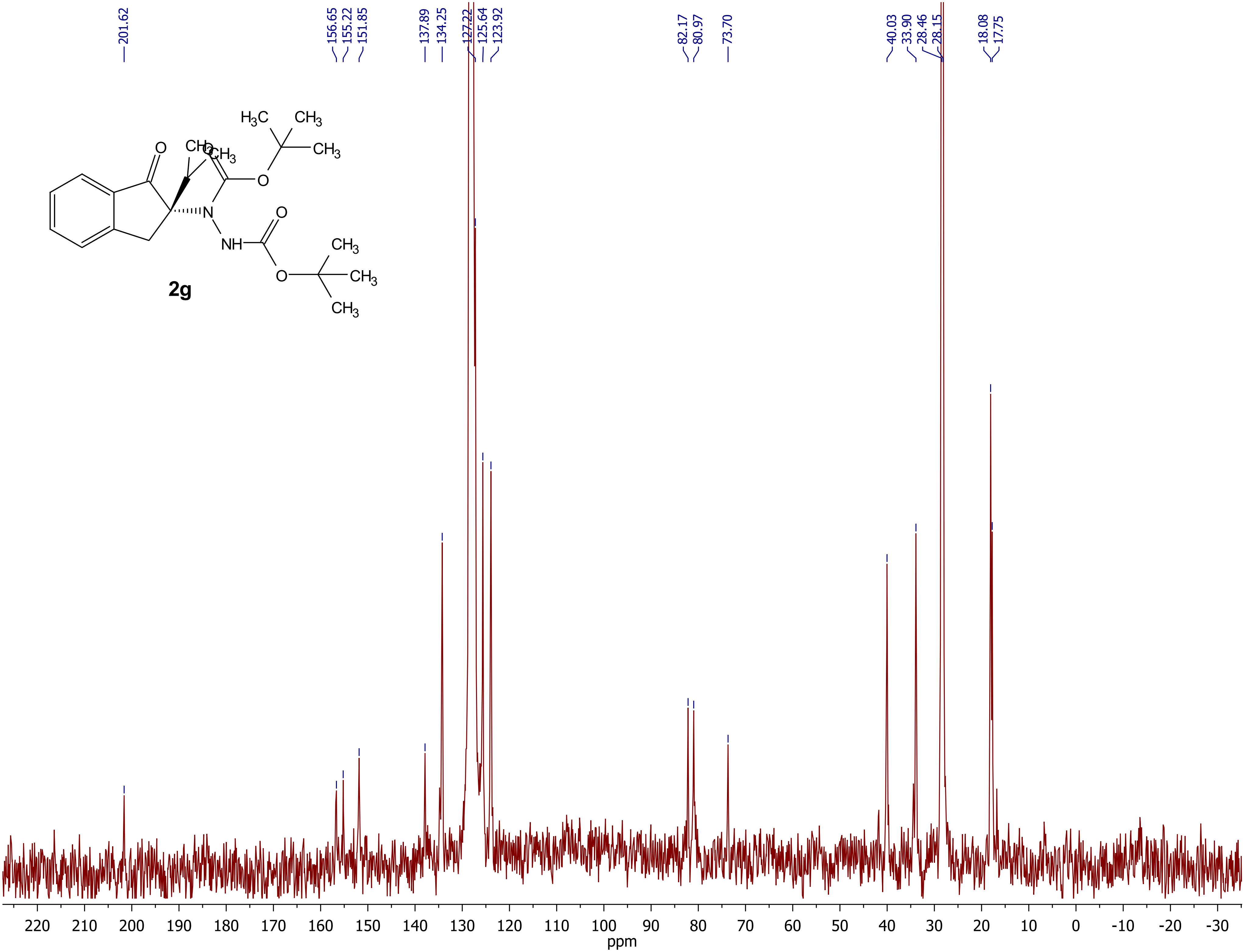
201.62
156.65
155.22
151.85
137.89
134.25
127.22
125.64
123.92
82.17
80.97
73.70
40.03
33.90
28.46
28.15
18.08
17.75
2g
220 210 200 190 180 170 160 150 140 130 120 110 100 90 80 70 60 50 40 30 20 10 0 -10 -20 -30
ppm

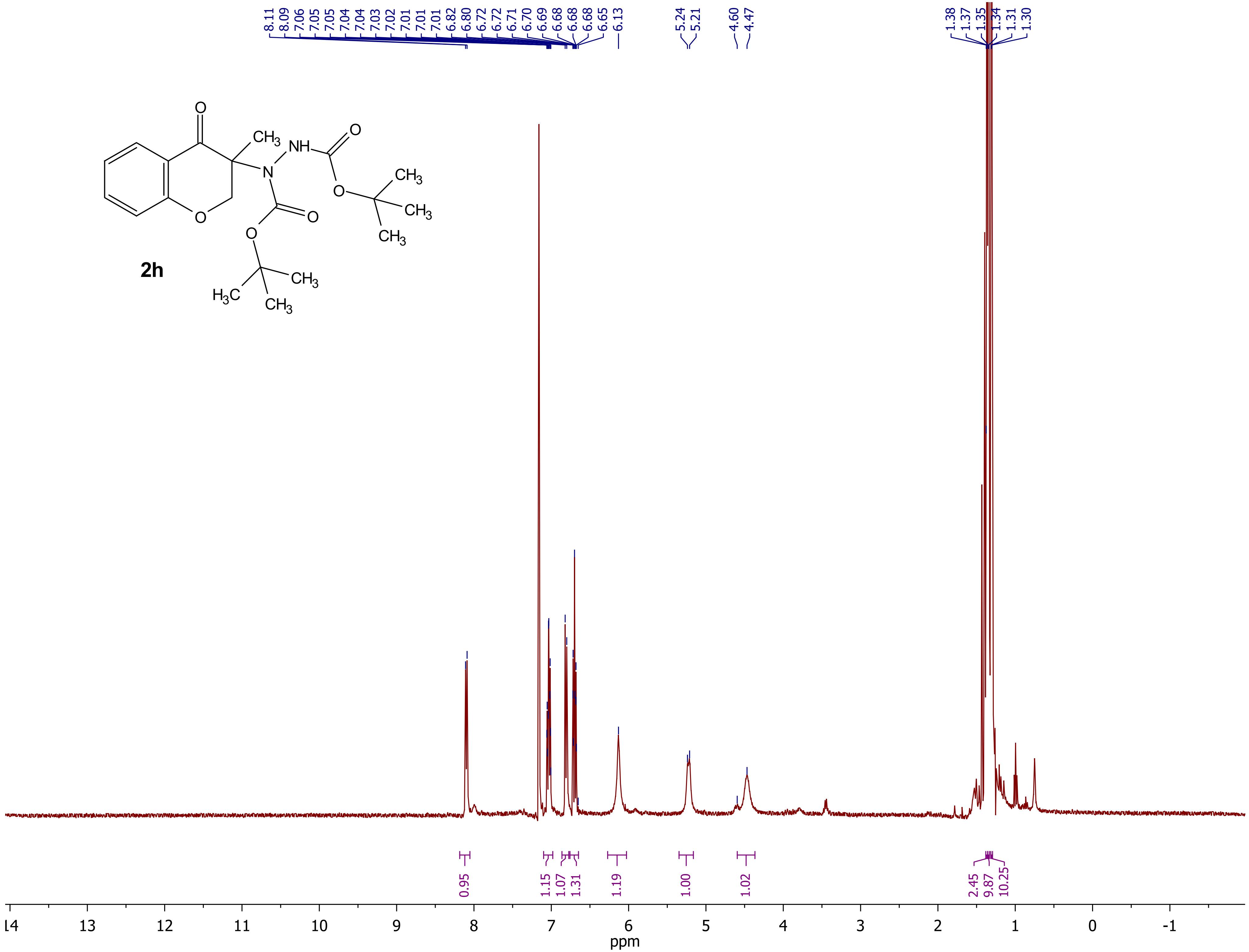
2h
ppm

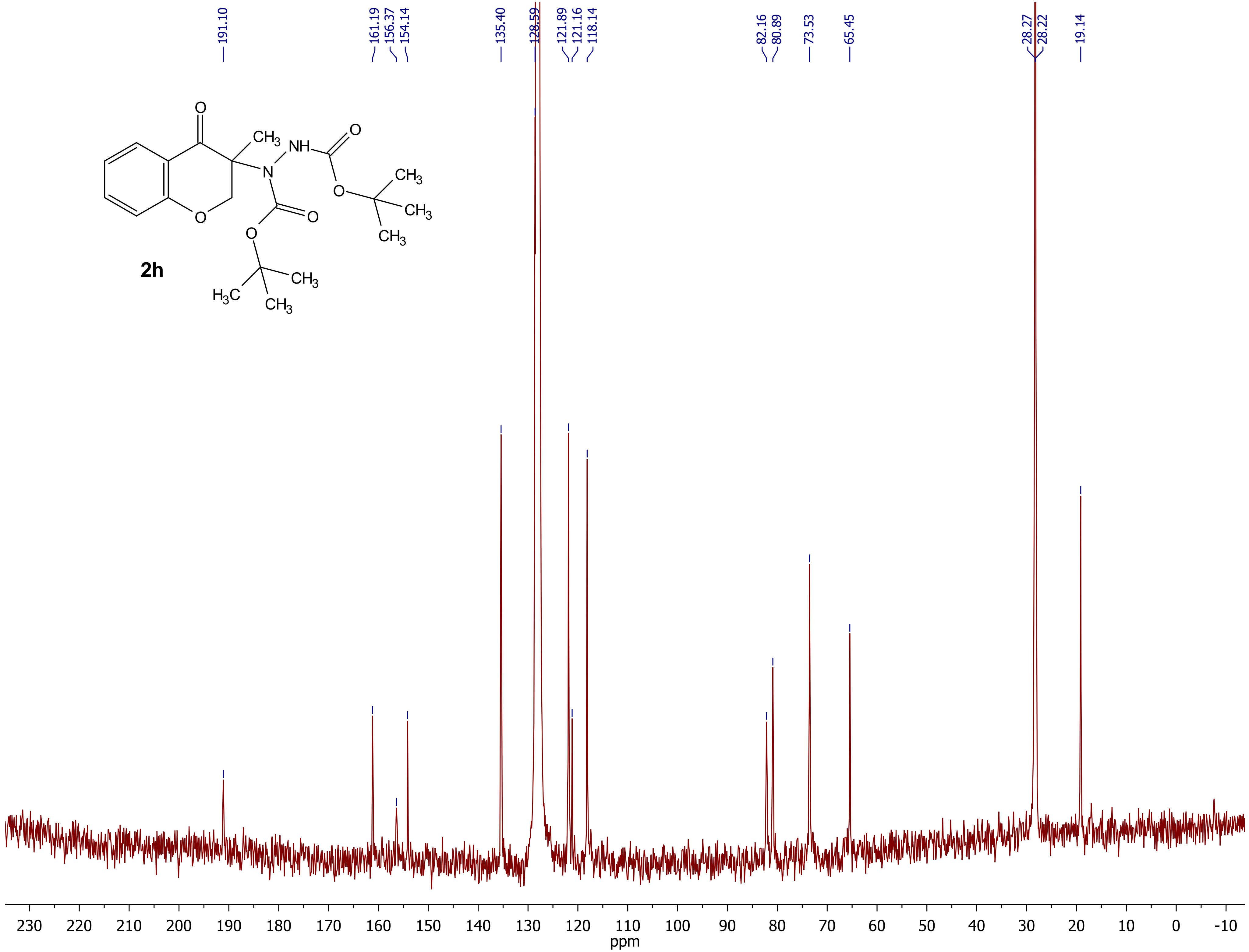
191.10
161.19
156.37
154.14
135.40
128.59
121.89
121.16
118.14
82.16
80.89
73.53
65.45
28.27
28.22
19.14
2h
230 220 210 200 190 180 170 160 150 140 130 120 110 100 90 80 70 60 50 40 30 20 10 0 -10
ppm

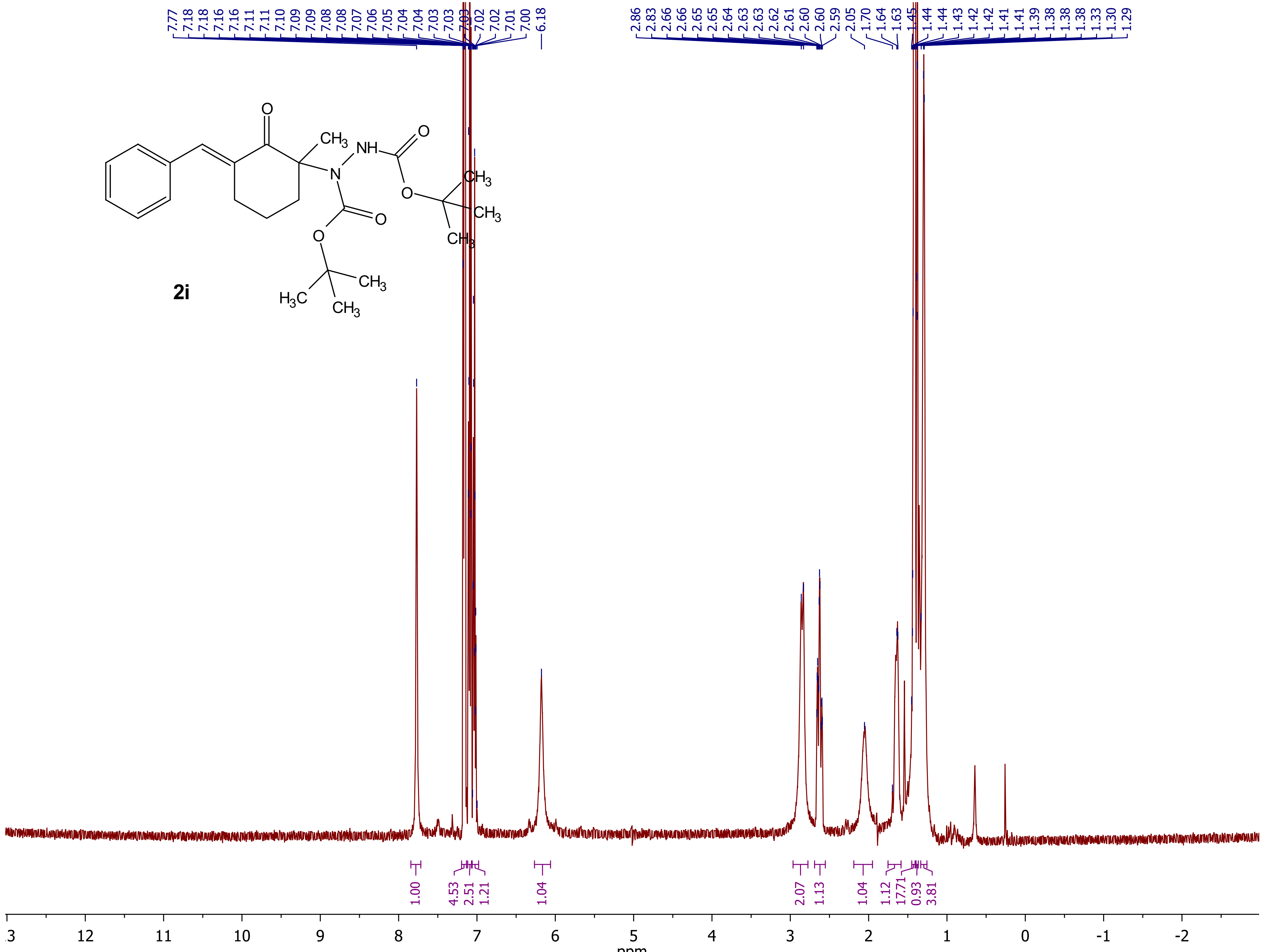
7.77
7.18
7.18
7.16
7.16
7.11
7.11
7.10
7.09
7.09
7.08
7.08
7.07
7.06
7.05
7.04
7.04
7.03
7.03
7.02
7.02
7.01
7.00
6.18
2.86
2.83
2.66
2.66
2.65
2.65
2.64
2.63
2.63
2.62
2.61
2.60
2.60
2.59
2.05
1.70
1.64
1.63
1.45
1.44
1.44
1.43
1.42
1.42
1.41
1.41
1.39
1.38
1.38
1.38
1.33
1.30
1.29
O
CH3
NH
O
N
O
CH3
CH3
CH3
O
O
2i
CH3
H3C
CH3
1.00
4.53
2.51
1.21
1.04
2.07
1.13
1.04
1.12
17.71
0.93
3.81
13
12
11
10
9
8
7
6
5
4
3
2
1
0
-1
-2
ppm

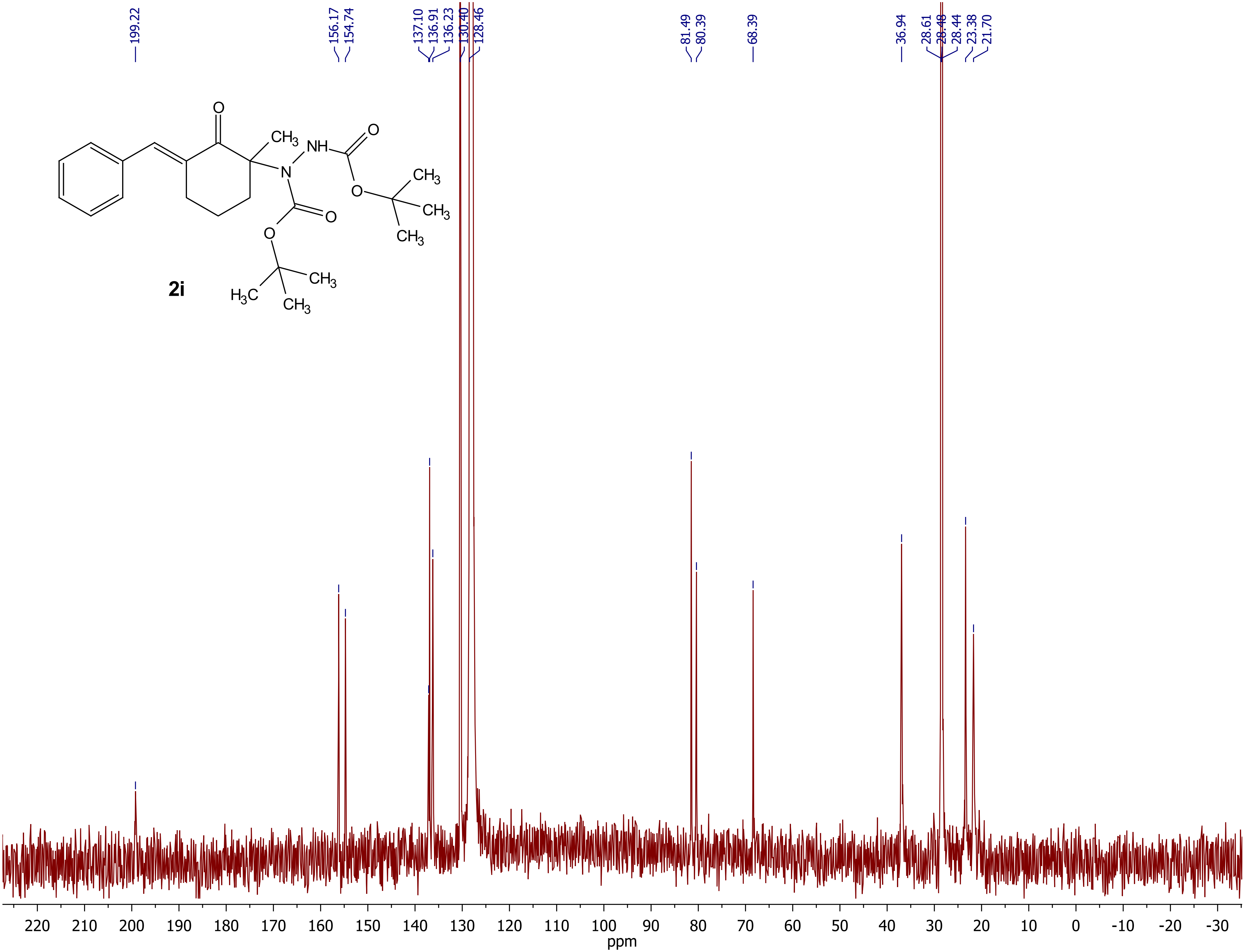
199.22
156.17
154.74
137.10
136.91
136.23
130.40
128.46
81.49
80.39
68.39
36.94
28.61
28.48
28.44
23.38
21.70
2i
ppm

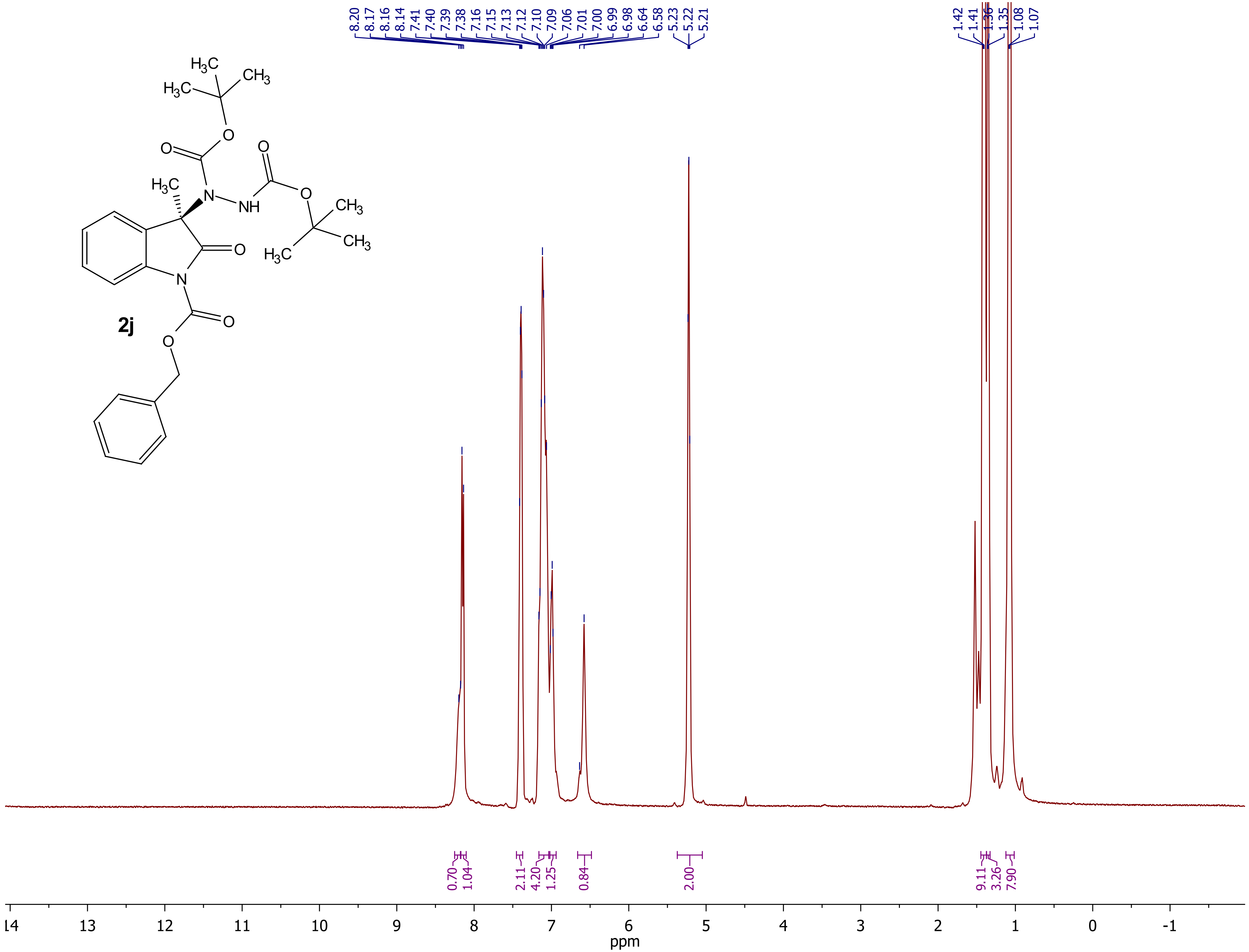
8.20
8.17
8.16
8.14
7.41
7.40
7.39
7.38
7.16
7.15
7.13
7.12
7.10
7.09
7.06
7.01
7.00
6.99
6.98
6.64
6.58
5.23
5.22
5.21
1.42
1.41
1.36
1.35
1.08
1.07
2j
0.70
1.04
2.11
4.20
1.25
0.84
2.00
9.11
3.26
7.90
ppm

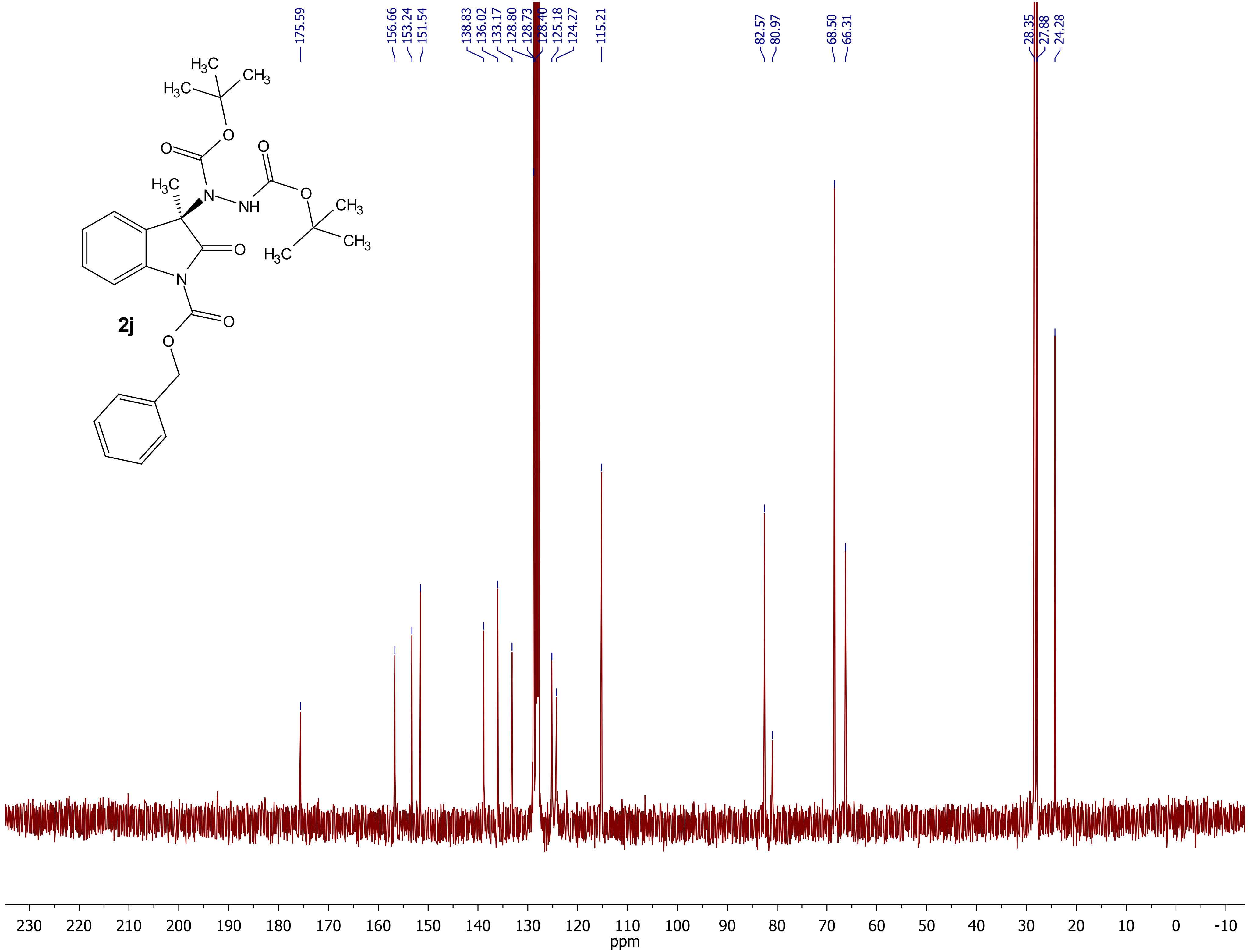

2j
175.59
156.66
153.24
151.54
138.83
136.02
133.17
128.80
128.73
128.40
125.18
124.27
115.21
82.57
80.97
68.50
66.31
28.35
27.88
24.28
230 220 210 200 190 180 170 160 150 140 130 120 110 100 90 80 70 60 50 40 30 20 10 0 -10
ppm

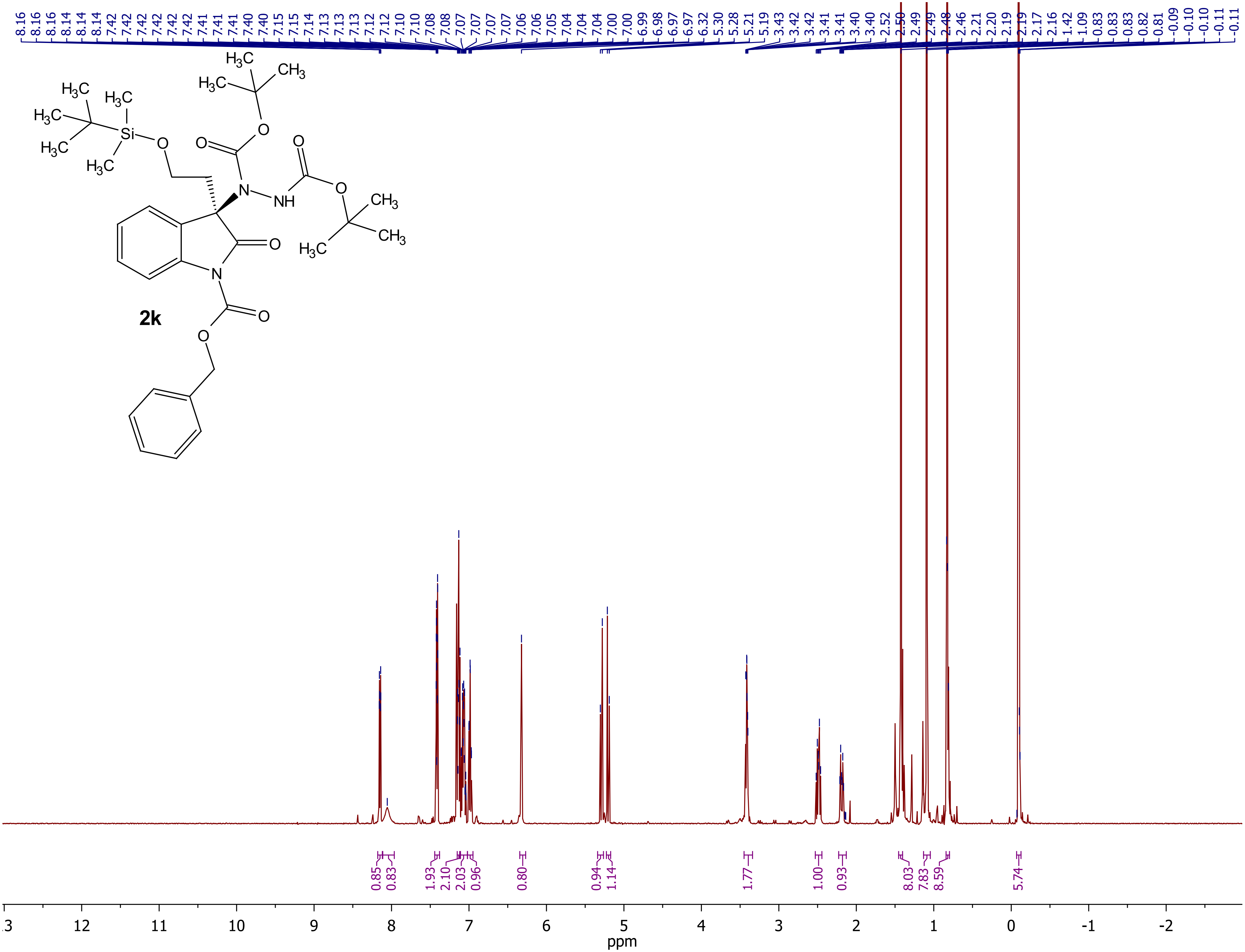

2k
8.16
8.16
8.16
8.14
8.14
8.14
7.42
7.42
7.42
7.42
7.42
7.41
7.41
7.41
7.40
7.40
7.15
7.15
7.14
7.13
7.13
7.13
7.12
7.12
7.10
7.10
7.08
7.08
7.07
7.07
7.07
7.06
7.06
7.05
7.04
7.04
7.04
7.00
7.00
6.99
6.98
6.97
6.97
6.32
5.30
5.28
5.21
5.19
3.43
3.42
3.42
3.41
3.41
3.40
3.40
2.52
2.50
2.49
2.49
2.48
2.46
2.21
2.20
2.19
2.19
2.17
2.16
1.42
1.09
0.83
0.83
0.82
0.81
-0.09
-0.10
-0.10
-0.11
-0.11
0.85
0.83
1.93
2.10
2.03
0.96
0.80
0.94
1.14
1.77
1.00
0.93
8.03
7.83
8.59
5.74
ppm

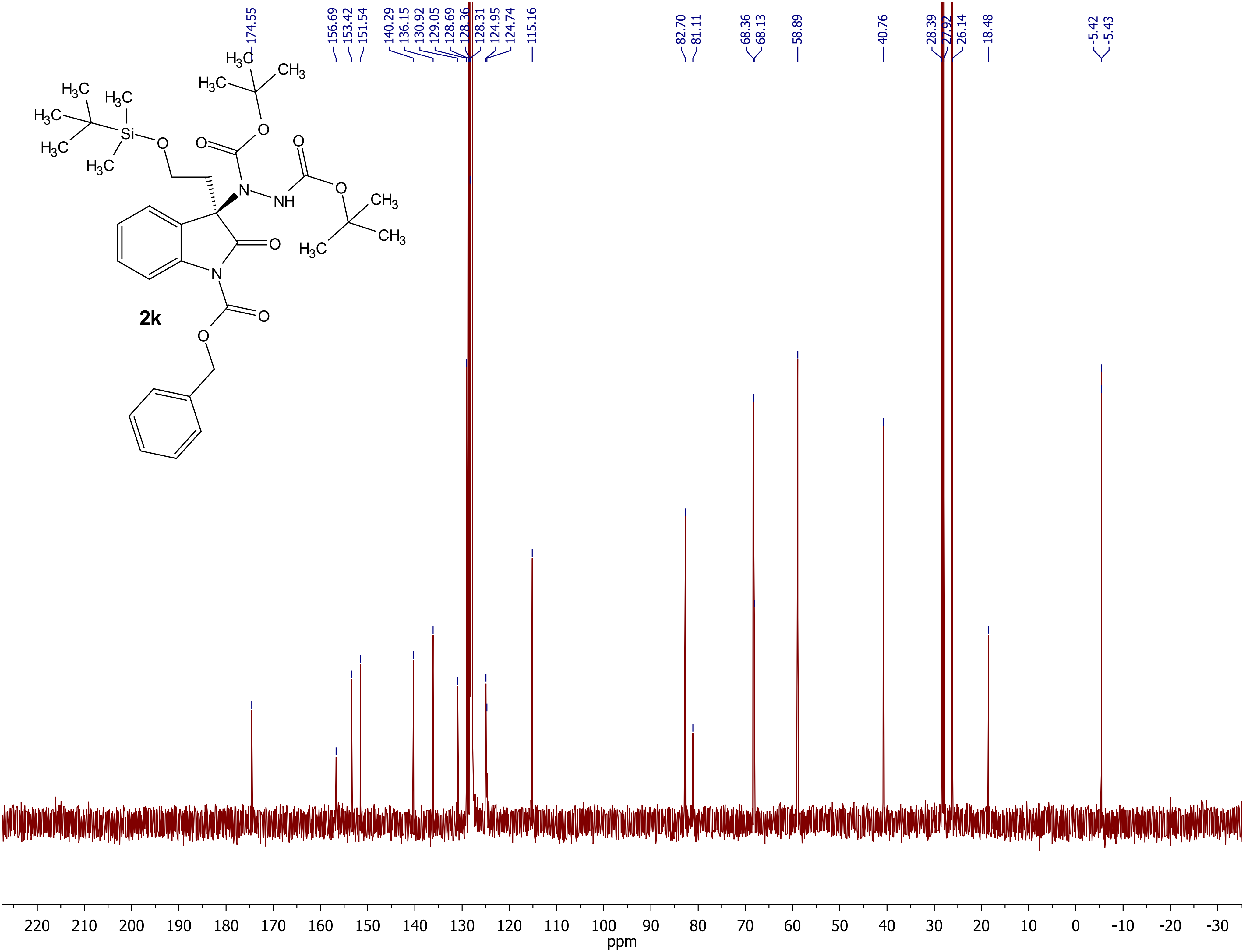
174.55
156.69
153.42
151.54
140.29
136.15
130.92
129.05
128.69
128.36
128.31
124.95
124.74
115.16
82.70
81.11
68.36
68.13
58.89
40.76
28.39
27.92
26.14
18.48
-5.42
-5.43
2k
220 210 200 190 180 170 160 150 140 130 120 110 100 90 80 70 60 50 40 30 20 10 0 -10 -20 -30
ppm

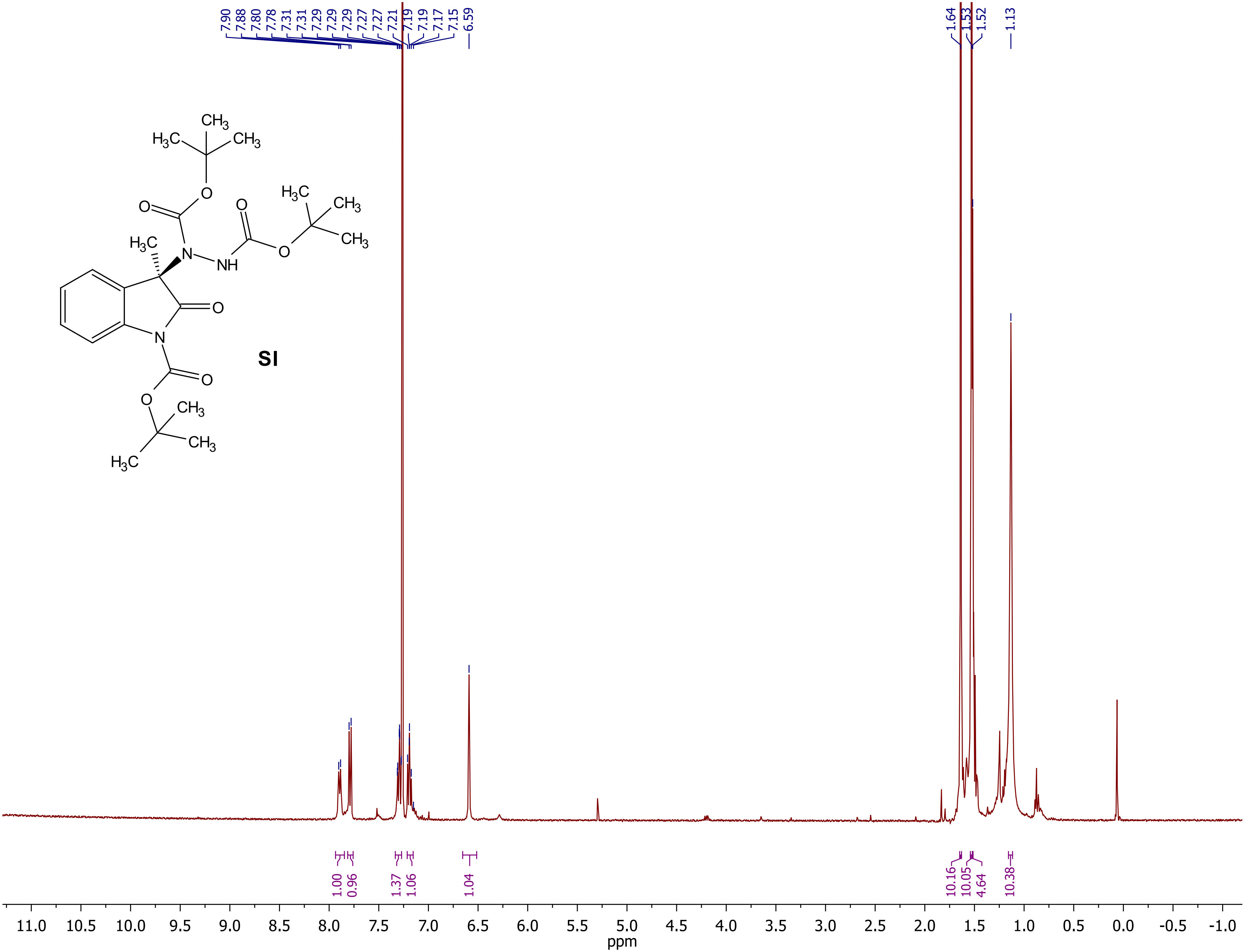
7.90
7.88
7.80
7.78
7.31
7.31
7.29
7.29
7.29
7.27
7.27
7.21
7.19
7.19
7.17
7.15
6.59
1.64
1.53
1.52
1.13
1.00
0.96
1.37
1.06
1.04
10.16
10.05
4.64
10.38
11.0
10.5
10.0
9.5
9.0
8.5
8.0
7.5
7.0
6.5
6.0
5.5
5.0
4.5
4.0
3.5
3.0
2.5
2.0
1.5
1.0
0.5
0.0
-0.5
-1.0
ppm

SI

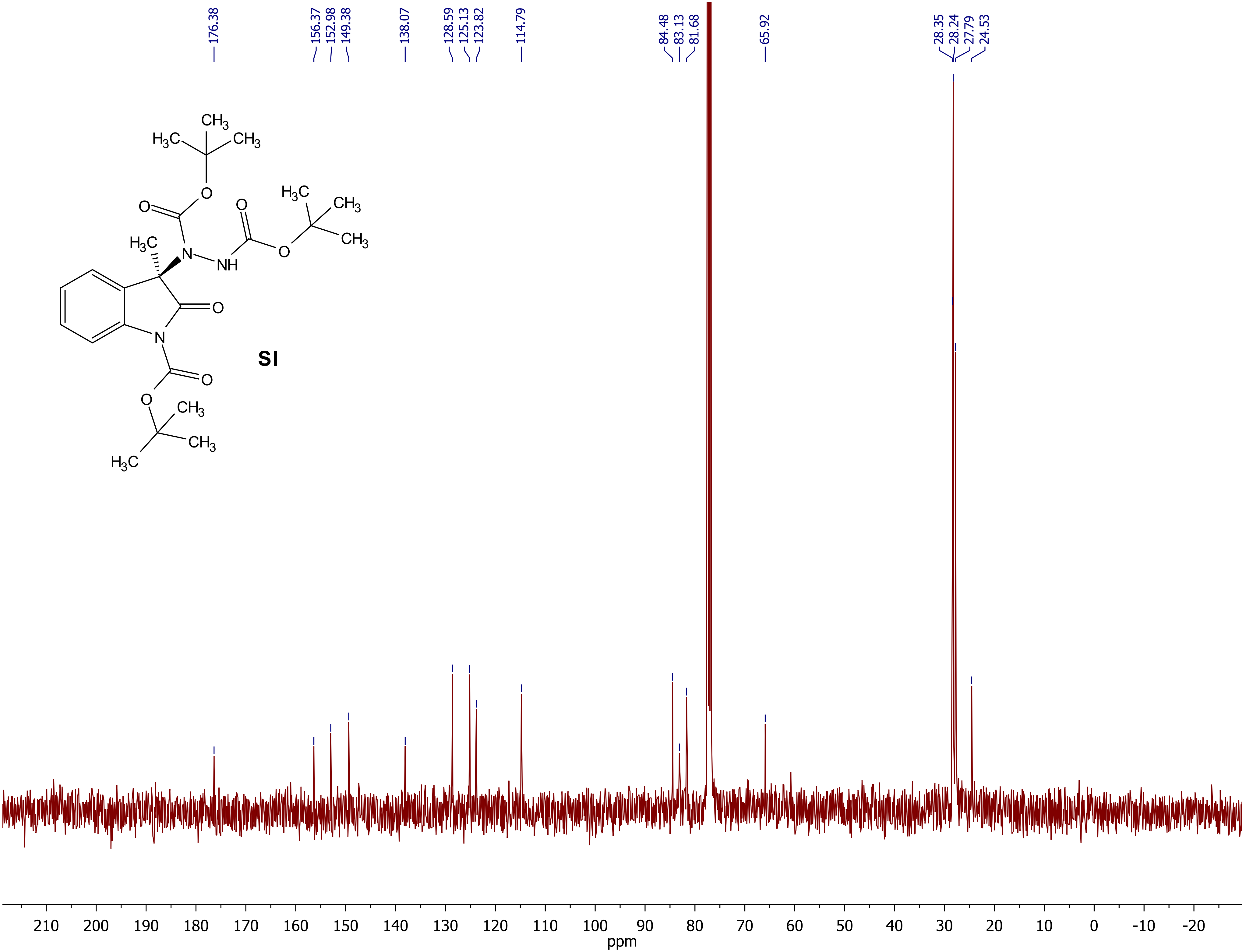
176.38
156.37
152.98
149.38
138.07
128.59
125.13
123.82
114.79
84.48
83.13
81.68
65.92
28.35
28.24
27.79
24.53
SI
210
200
190
180
170
160
150
140
130
120
110
100
90
80
70
60
50
40
30
20
10
0
-10
-20
ppm

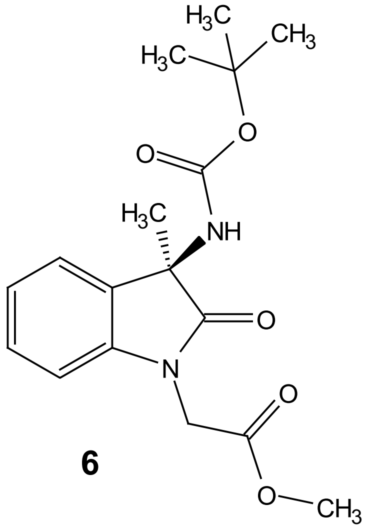
H3C
CH3
H3C
O
O
H3C
NH
O
N
O
O
CH3

6

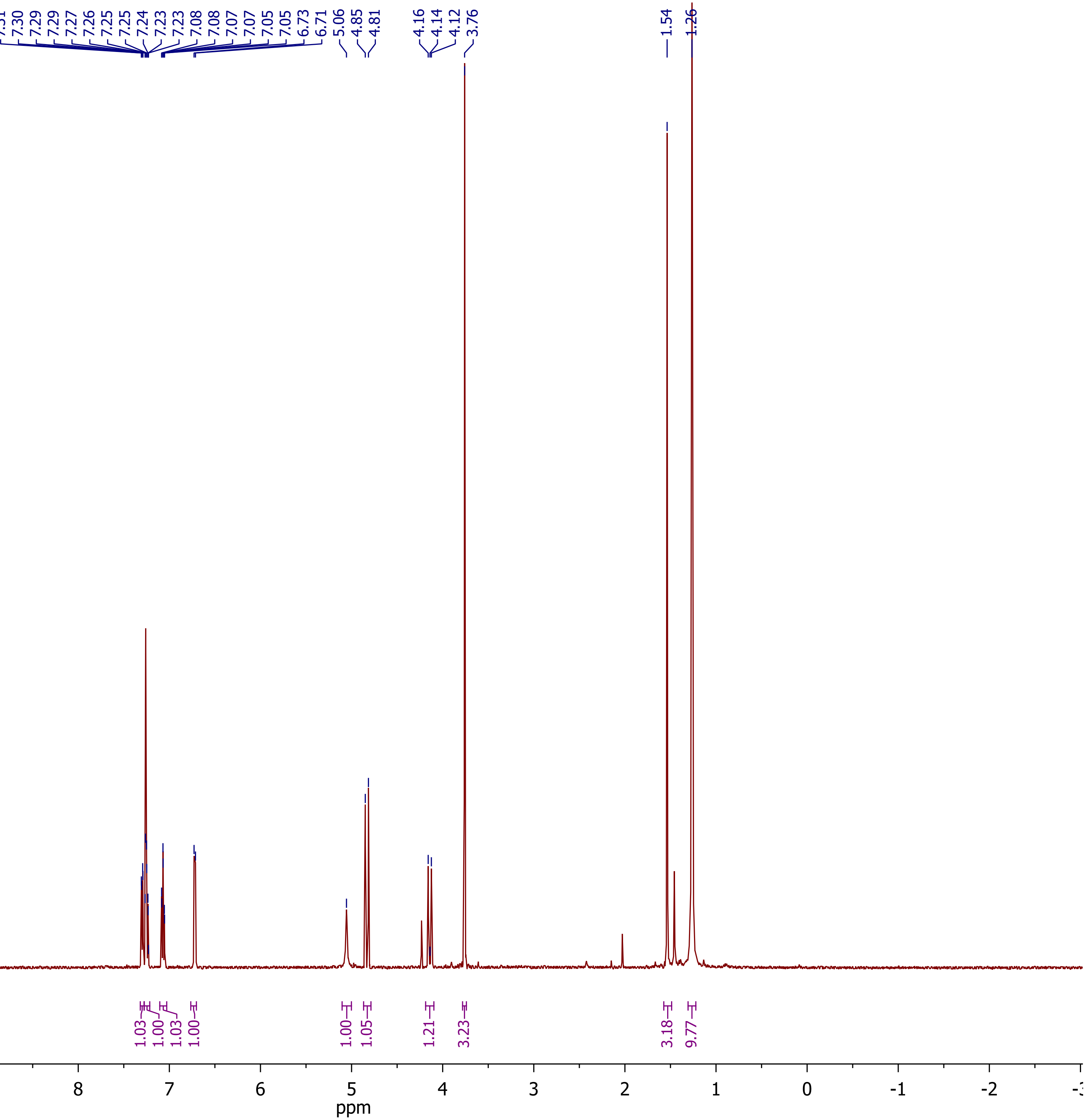
7.31
7.30
7.29
7.29
7.27
7.26
7.25
7.25
7.24
7.23
7.23
7.08
7.08
7.07
7.07
7.05
7.05
6.73
6.71
5.06
4.85
4.81
4.16
4.14
4.12
3.76
1.54
1.26
1.03
1.00
1.03
1.00
1.00
1.05
1.21
3.23
3.18
9.77
12
11
10
9
8
7
6
5
4
3
2
1
0
-1
-2
ppm

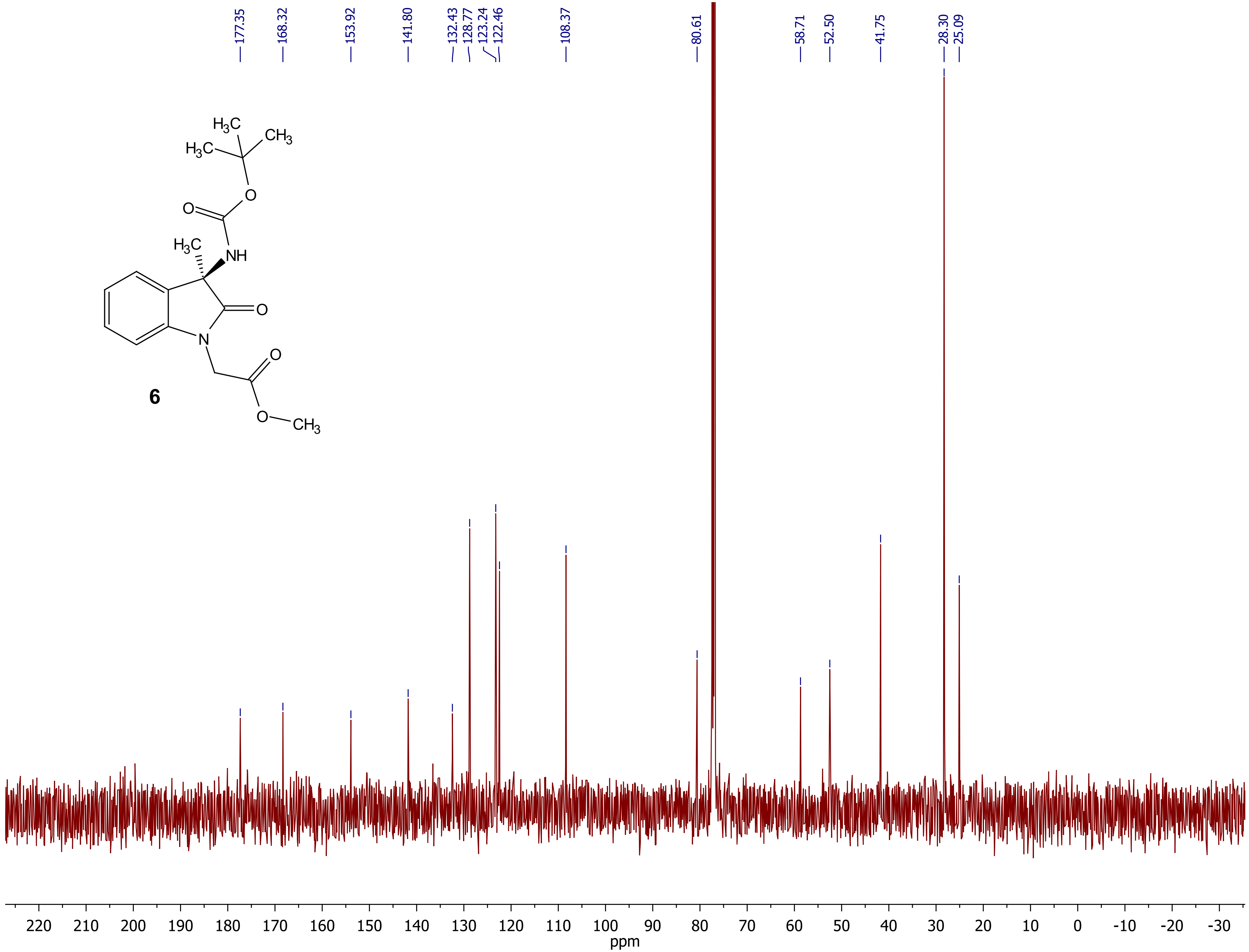

177.35
168.32
153.92
141.80
132.43
128.77
123.24
122.46
108.37
80.61
58.71
52.50
41.75
28.30
25.09
H3C
CH3
H3C
O
O
H3C
NH
O
N
O
O
CH3
6
220 210 200 190 180 170 160 150 140 130 120 110 100 90 80 70 60 50 40 30 20 10 0 -10 -20 -30
ppm

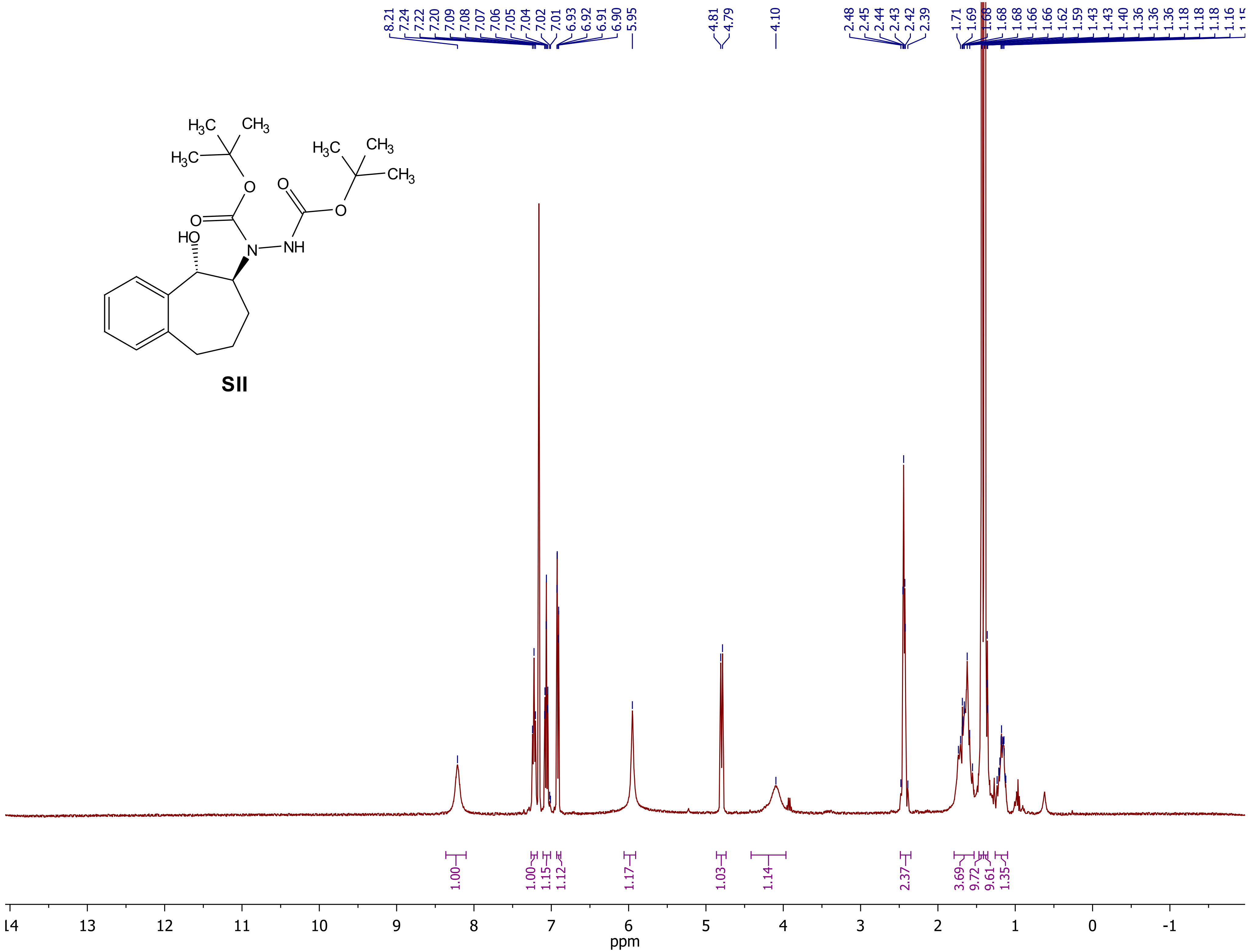
8.21
7.24
7.22
7.20
7.09
7.08
7.07
7.06
7.05
7.04
7.02
7.01
6.93
6.92
6.91
6.90
5.95
4.81
4.79
4.10
2.48
2.45
2.44
2.43
2.42
2.39
1.71
1.69
1.68
1.68
1.66
1.66
1.62
1.59
1.43
1.43
1.40
1.36
1.36
1.36
1.18
1.18
1.18
1.16
H3C
CH3
H3C
O
O
HO
N
NH
O
O
CH3
SII
1.00
1.00
1.15
1.12
1.17
1.03
1.14
2.37
3.69
9.72
9.61
1.35
14
13
12
11
10
9
8
7
6
5
4
3
2
1
0
-1
ppm

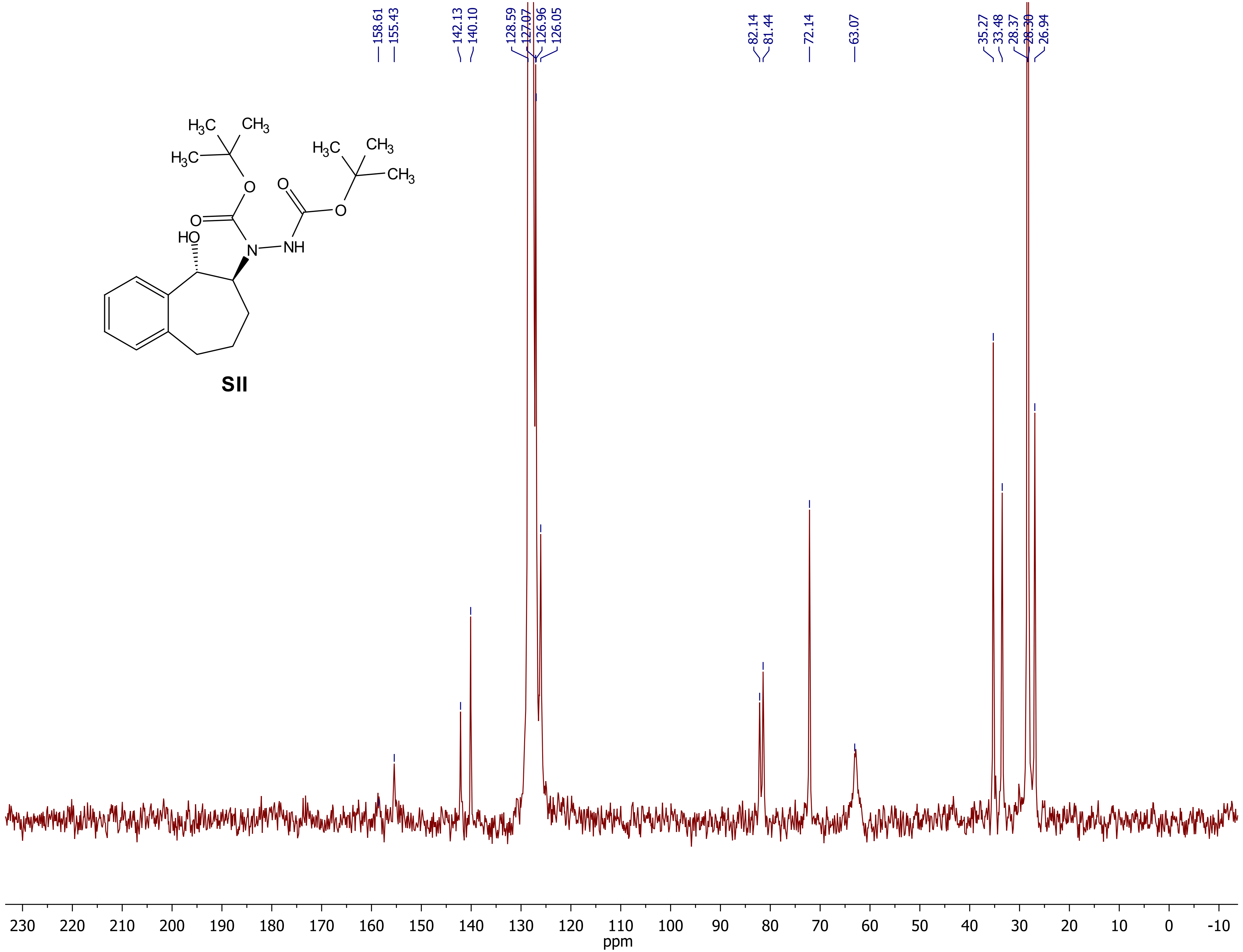

158.61
155.43
142.13
140.10
128.59
127.07
126.96
126.05
82.14
81.44
72.14
63.07
35.27
33.48
28.37
28.30
26.94
H3C
CH3
H3C
O
O
HO
N
NH
O
O
H3C
CH3
CH3
SII
230
220
210
200
190
180
170
160
150
140
130
120
110
100
90
80
70
60
50
40
30
20
10
0
-10
ppm

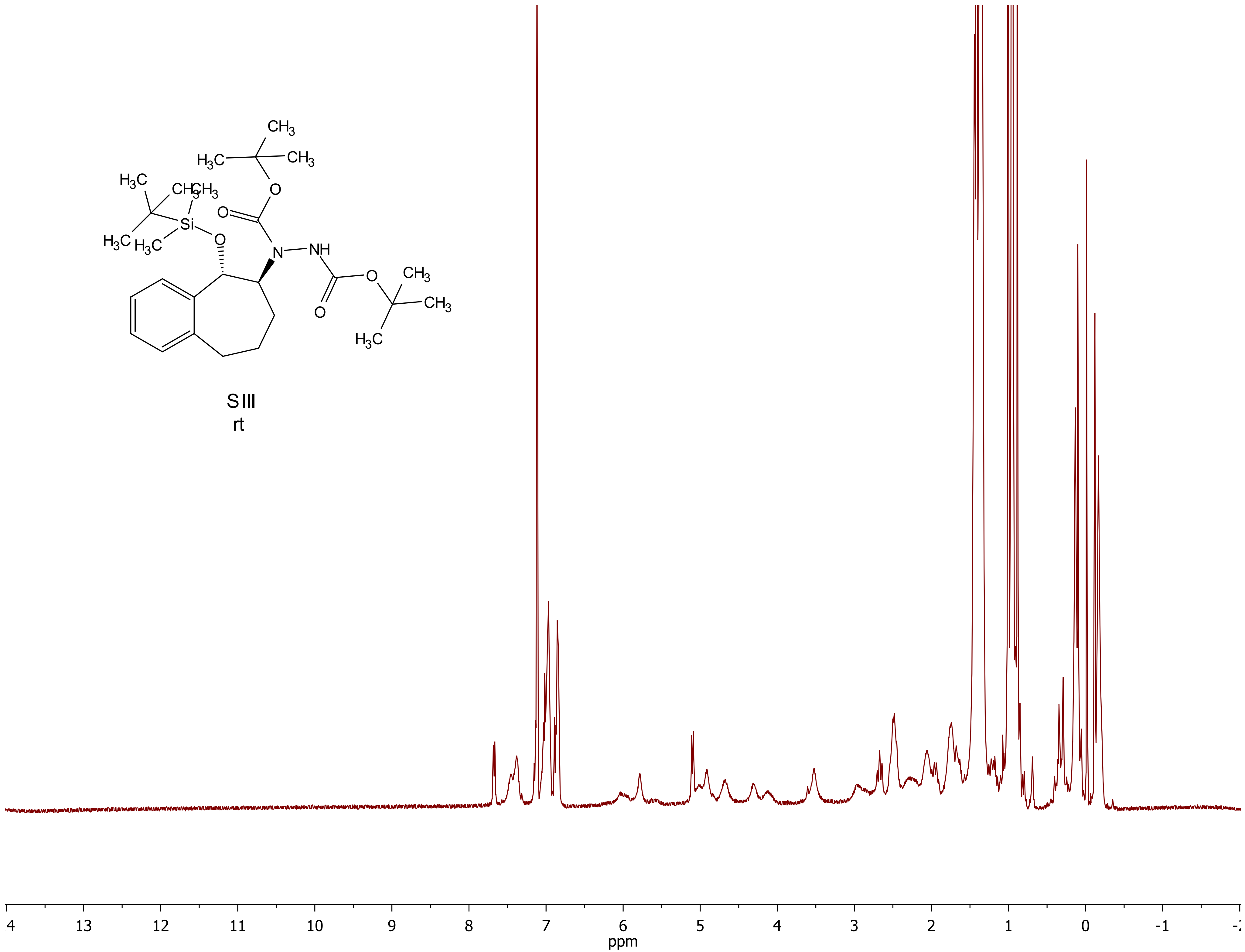

S III
rt
ppm

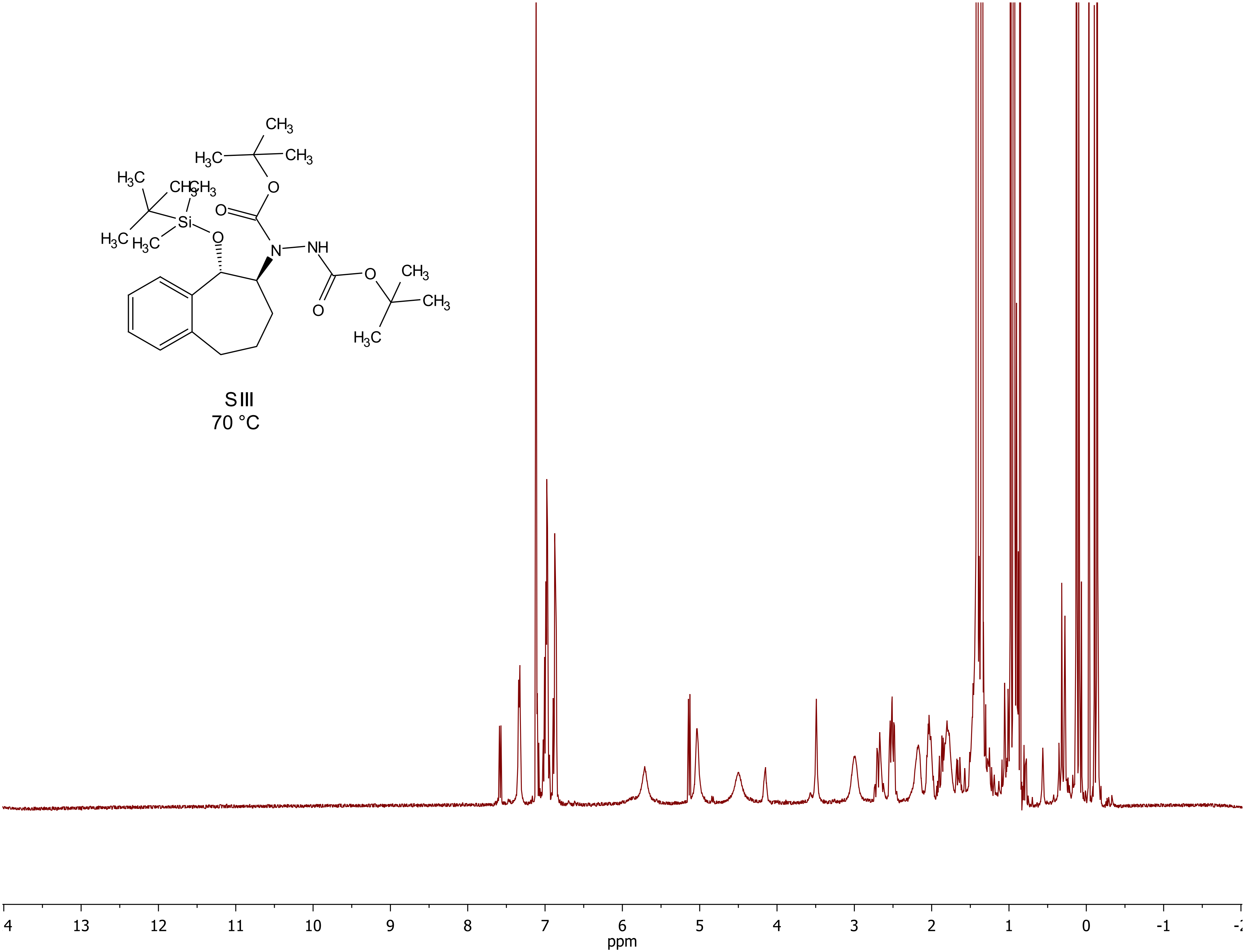
SIII
70 °C
4
13
12
11
10
9
8
7
6
5
4
3
2
1
0
-1
ppm

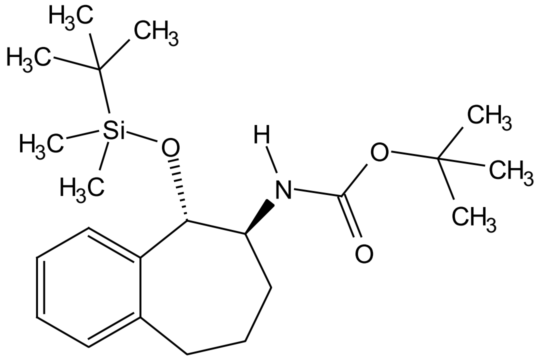
H3C
CH3
H3C
Si
O
H3C
H3C
H
N
O
CH3
CH3
CH3
O

7

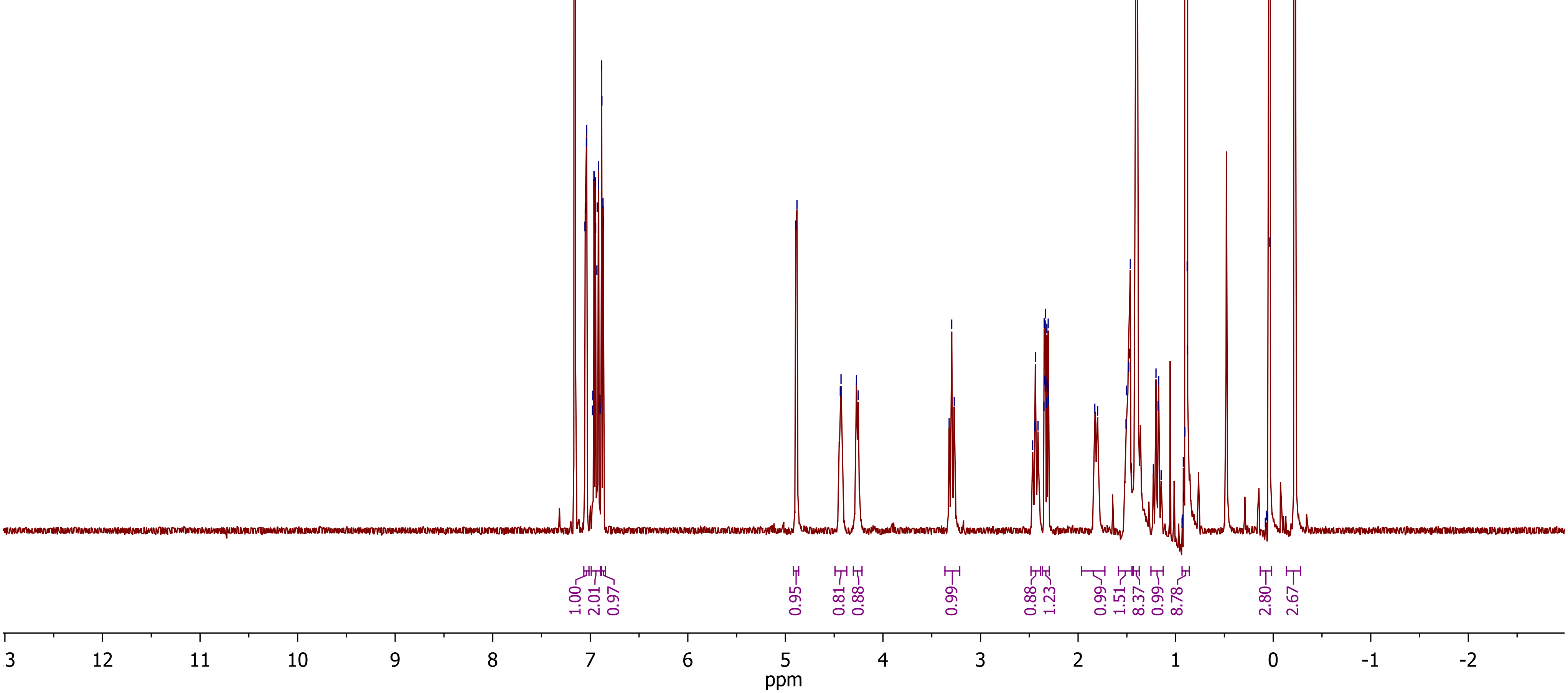
7.05
7.05
7.04
7.04
6.98
6.97
6.96
6.96
6.95
6.94
6.93
6.93
6.92
6.91
6.90
6.88
6.88
6.87
6.87
4.89
4.88
4.44
4.43
4.27
4.25
3.32
3.30
3.27
2.47
2.45
2.44
2.41
2.35
2.35
2.34
2.34
2.33
2.33
2.32
2.32
2.31
2.31
2.30
1.83
1.80
1.51
1.50
1.48
1.47
1.46
1.45
1.40
1.23
1.20
1.18
1.17
1.15
0.93
0.93
0.92
0.92
0.91
0.90
0.89
0.89
0.88
0.88
0.08
0.07
0.06
0.04
0.03
-0.22
1.00
2.01
0.97
0.95
0.81
0.88
0.99
0.88
1.23
0.99
1.51
8.37
0.99
8.78
2.80
2.67
.3
12
11
10
9
8
7
6
5
4
3
2
1
0
-1
-2
ppm

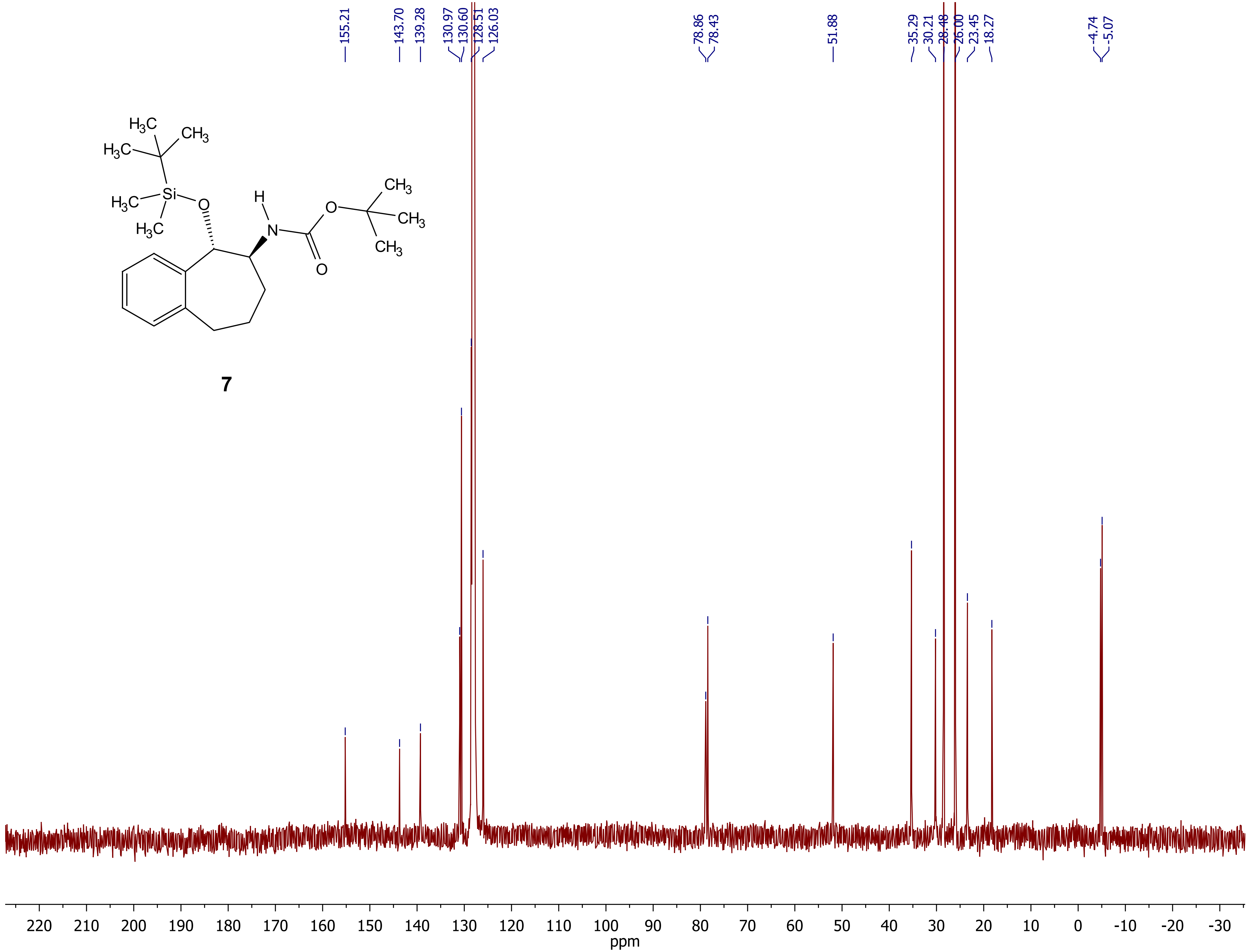
155.21
143.70
139.28
130.97
130.60
128.51
126.03
78.86
78.43
51.88
35.29
30.21
28.48
26.00
23.45
18.27
-4.74
-5.07
H_3C
CH_3
H_3C
Si
H_3C
H_3C
O
H
N
O
CH_3
CH_3
CH_3
O
7
220 210 200 190 180 170 160 150 140 130 120 110 100 90 80 70 60 50 40 30 20 10 0 -10 -20 -30
ppm

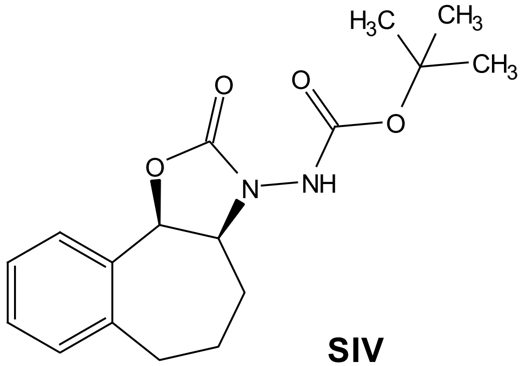

O
O
N
NH
O
O
H_3C
CH_3
CH_3

SIV

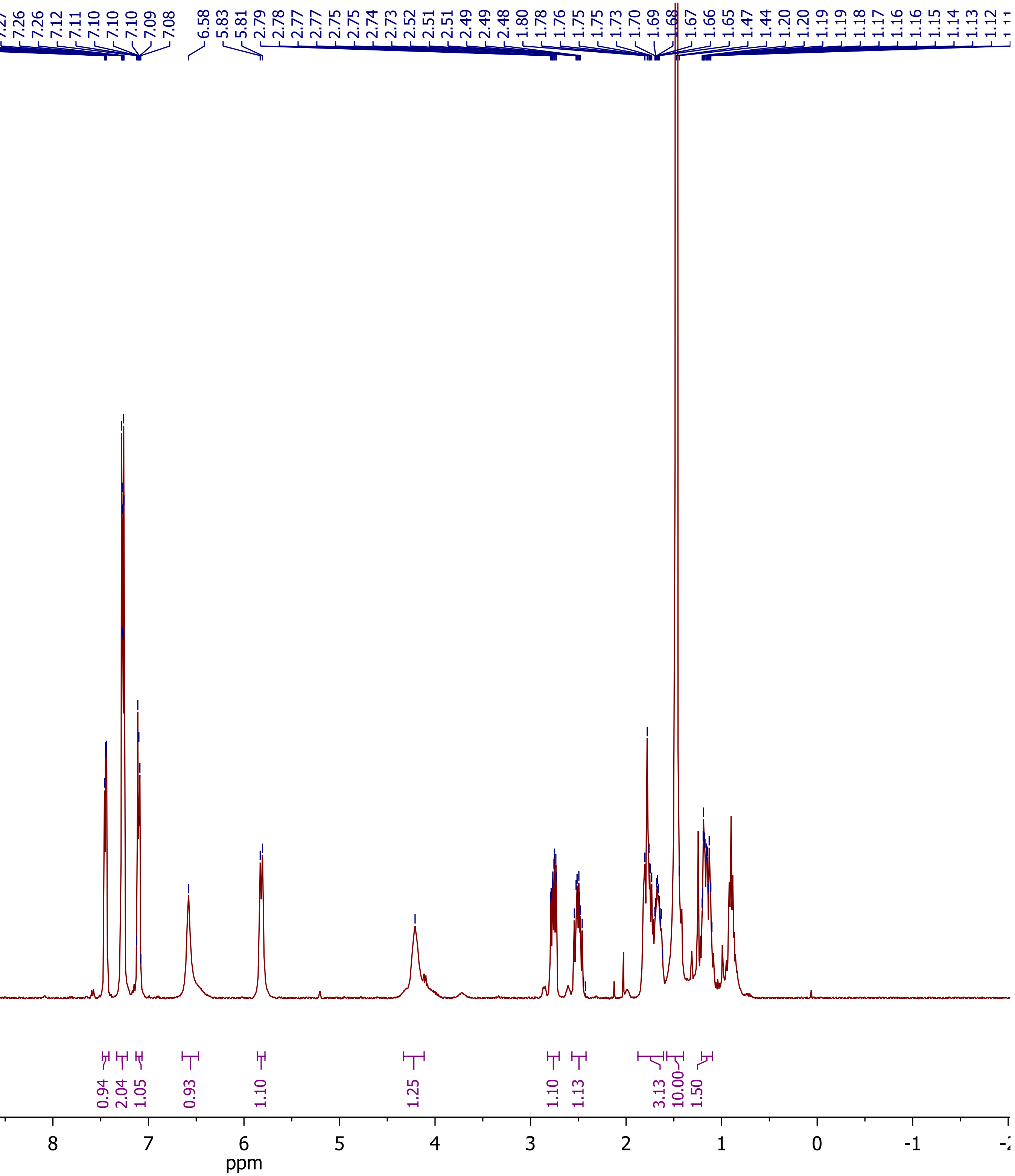

7.46
7.45
7.45
7.44
7.28
7.28
7.27
7.27
7.27
7.26
7.26
7.12
7.11
7.10
7.10
7.10
7.09
7.08
6.58
5.83
5.81
2.79
2.78
2.77
2.77
2.75
2.75
2.74
2.73
2.52
2.51
2.51
2.49
2.49
2.48
1.80
1.78
1.76
1.75
1.75
1.73
1.70
1.69
1.68
1.67
1.66
1.65
1.47
1.44
1.20
1.20
1.19
1.19
1.18
1.17
1.16
1.16
1.15
1.14
1.13
1.12
0.94
2.04
1.05
0.93
1.10
1.25
1.10
1.13
3.13
10.00
1.50
13
12
11
10
9
8
7
6
5
4
3
2
1
0
-1
ppm

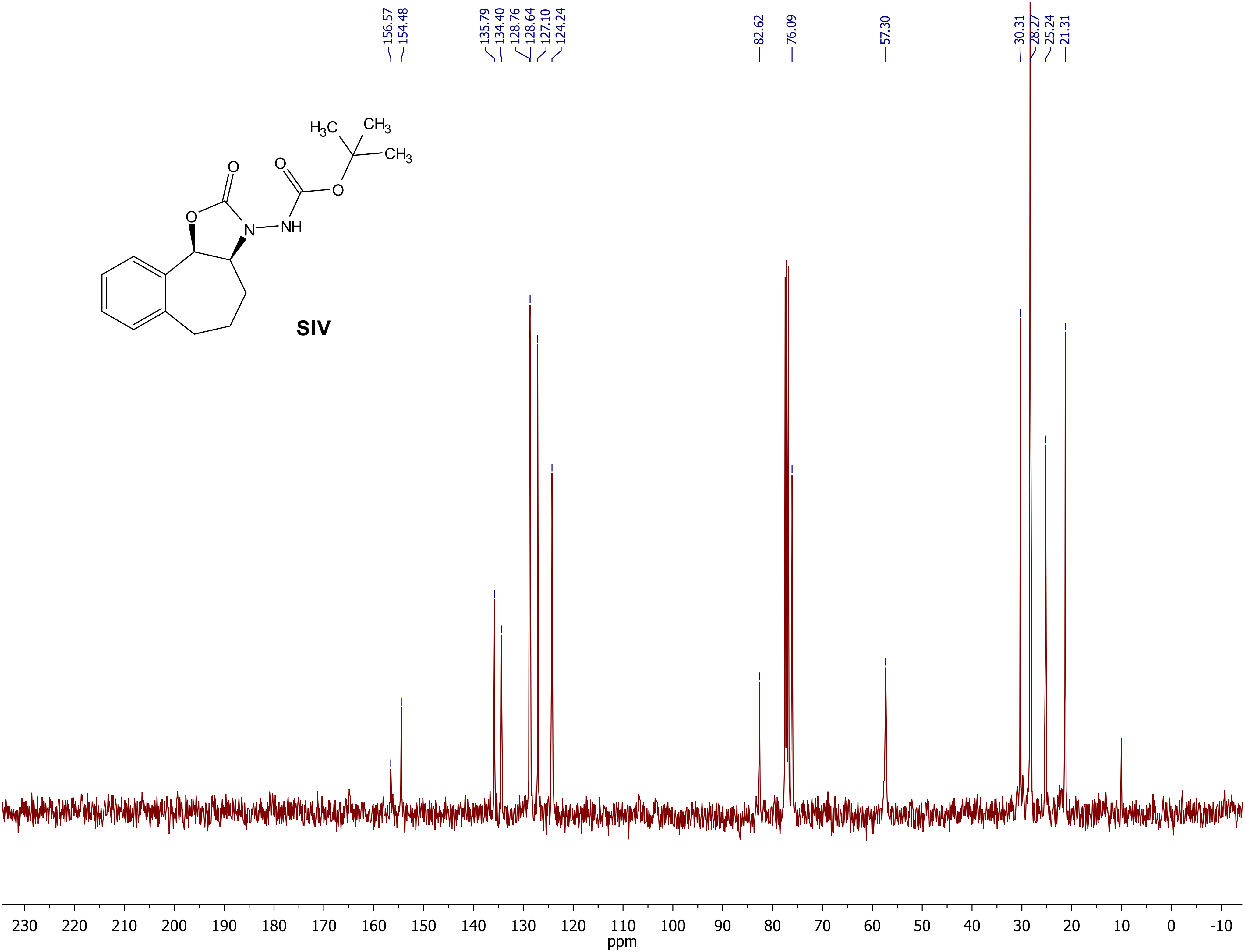
156.57
154.48
135.79
134.40
128.76
128.64
127.10
124.24
82.62
76.09
57.30
30.31
28.27
25.24
21.31
H3C
CH3
CH3
O
O
O
O
N
NH
SIV
230 220 210 200 190 180 170 160 150 140 130 120 110 100 90 80 70 60 50 40 30 20 10 0 -10
ppm

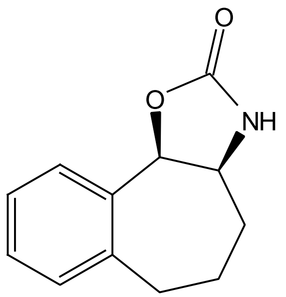

8

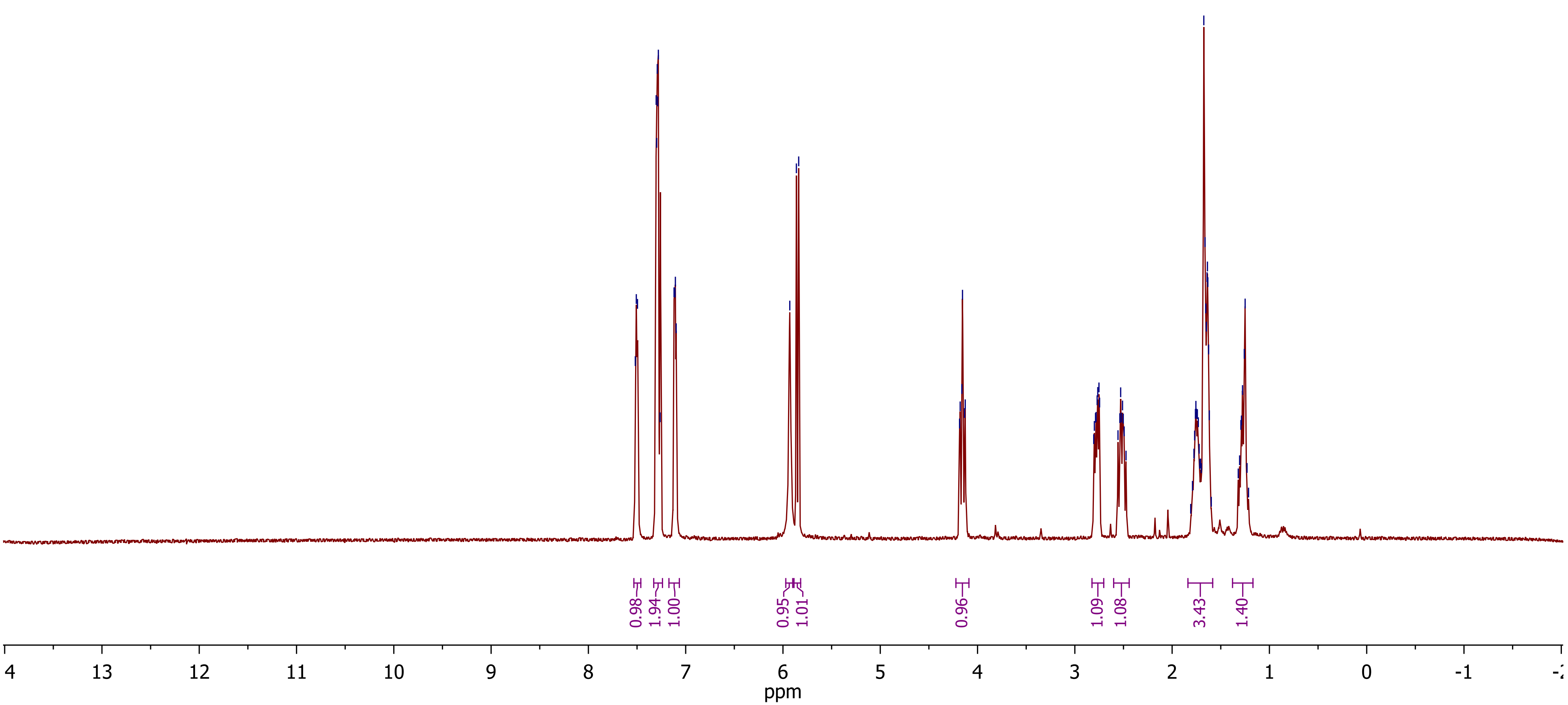
7.52 7.51 7.50 7.30 7.30 7.29 7.29 7.28 7.26 7.12 7.11 7.11 7.10
5.93 5.86 5.84 4.18 4.18 4.16 4.15 4.13 4.13 2.80 2.79 2.78 2.77 2.76 2.75 2.75 2.54 2.53 2.52 2.51 2.50 2.49 1.76 1.76 1.75 1.74 1.73 1.67 1.66 1.65 1.65 1.64 1.64 1.63 1.62 1.62 1.29 1.29 1.28 1.26
0.98 1.94 1.00 0.95 1.01 0.96 1.09 1.08 3.43 1.40
13 12 11 10 9 8 7 6 5 4 3 2 1 0 -1
ppm

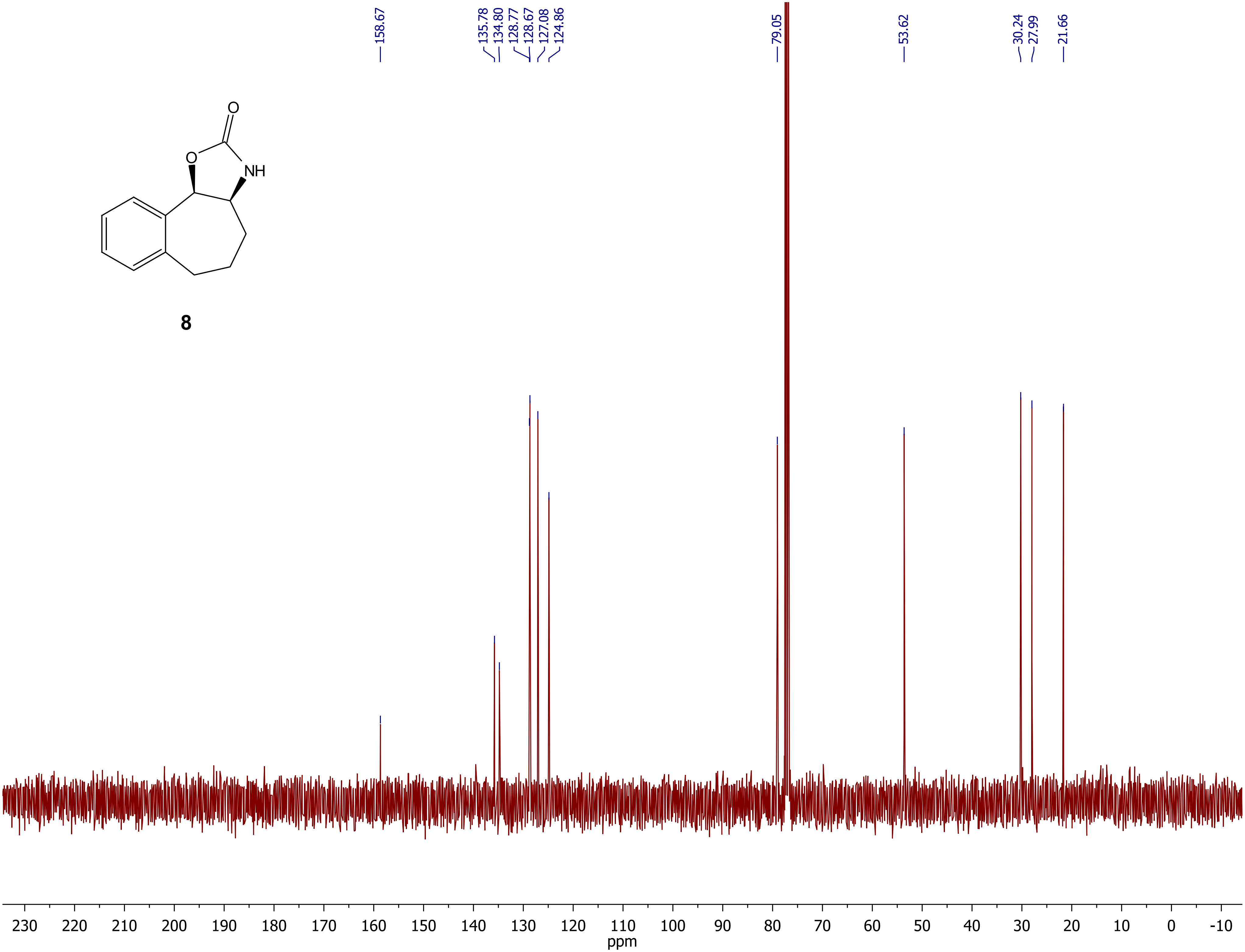
158.67
135.78
134.80
128.77
128.67
127.08
124.86
79.05
53.62
30.24
27.99
21.66
O
O
NH
8
230 220 210 200 190 180 170 160 150 140 130 120 110 100 90 80 70 60 50 40 30 20 10 0 -10
ppm

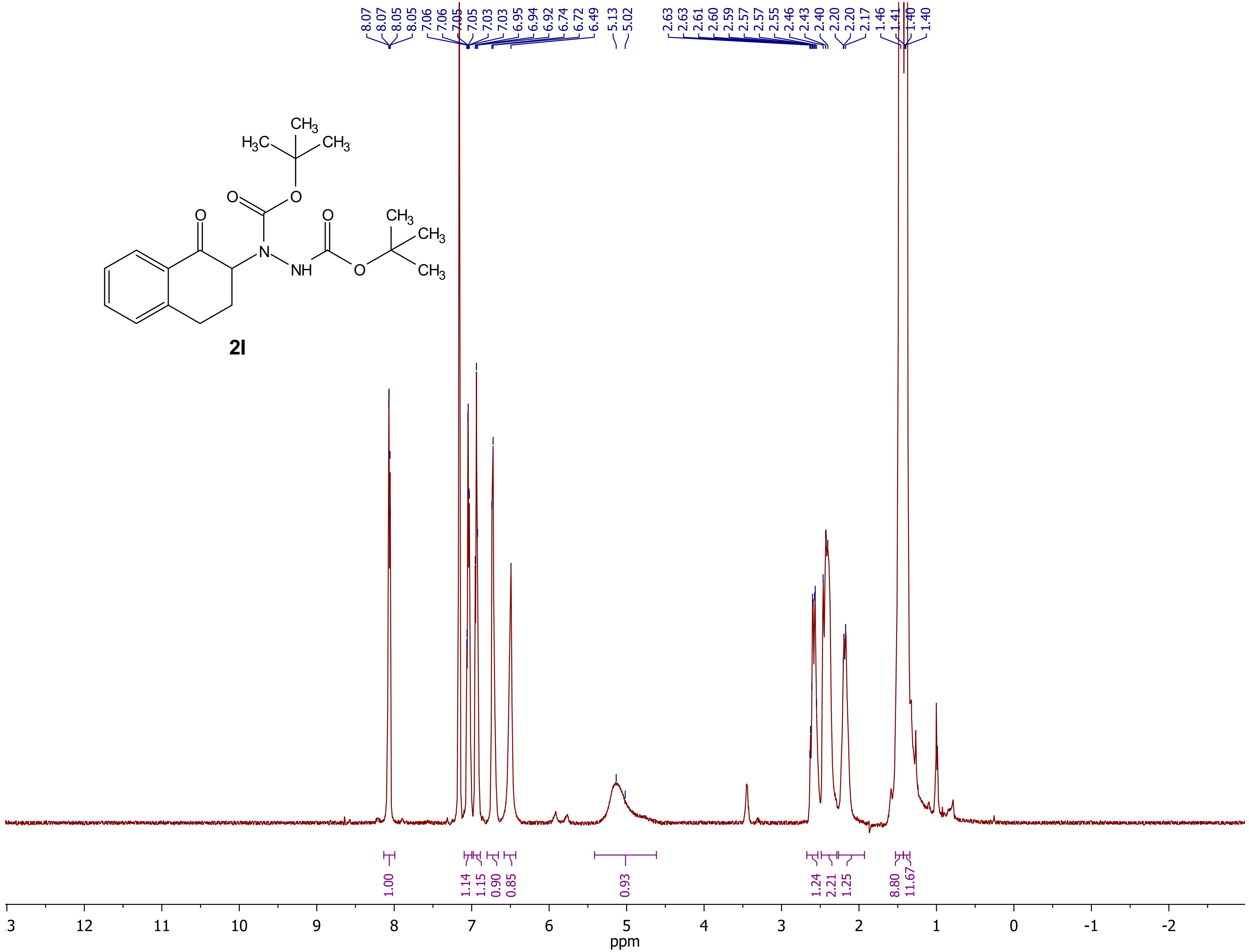
8.07
8.07
8.05
8.05
7.06
7.06
7.05
7.05
7.03
7.03
6.95
6.94
6.92
6.74
6.72
6.49
5.13
5.02
2.63
2.63
2.61
2.60
2.59
2.57
2.57
2.55
2.46
2.43
2.40
2.20
2.20
2.17
1.46
1.41
1.40
1.40
2l
1.00
1.14
1.15
0.90
0.85
0.93
1.24
2.21
1.25
8.80
11.67
ppm

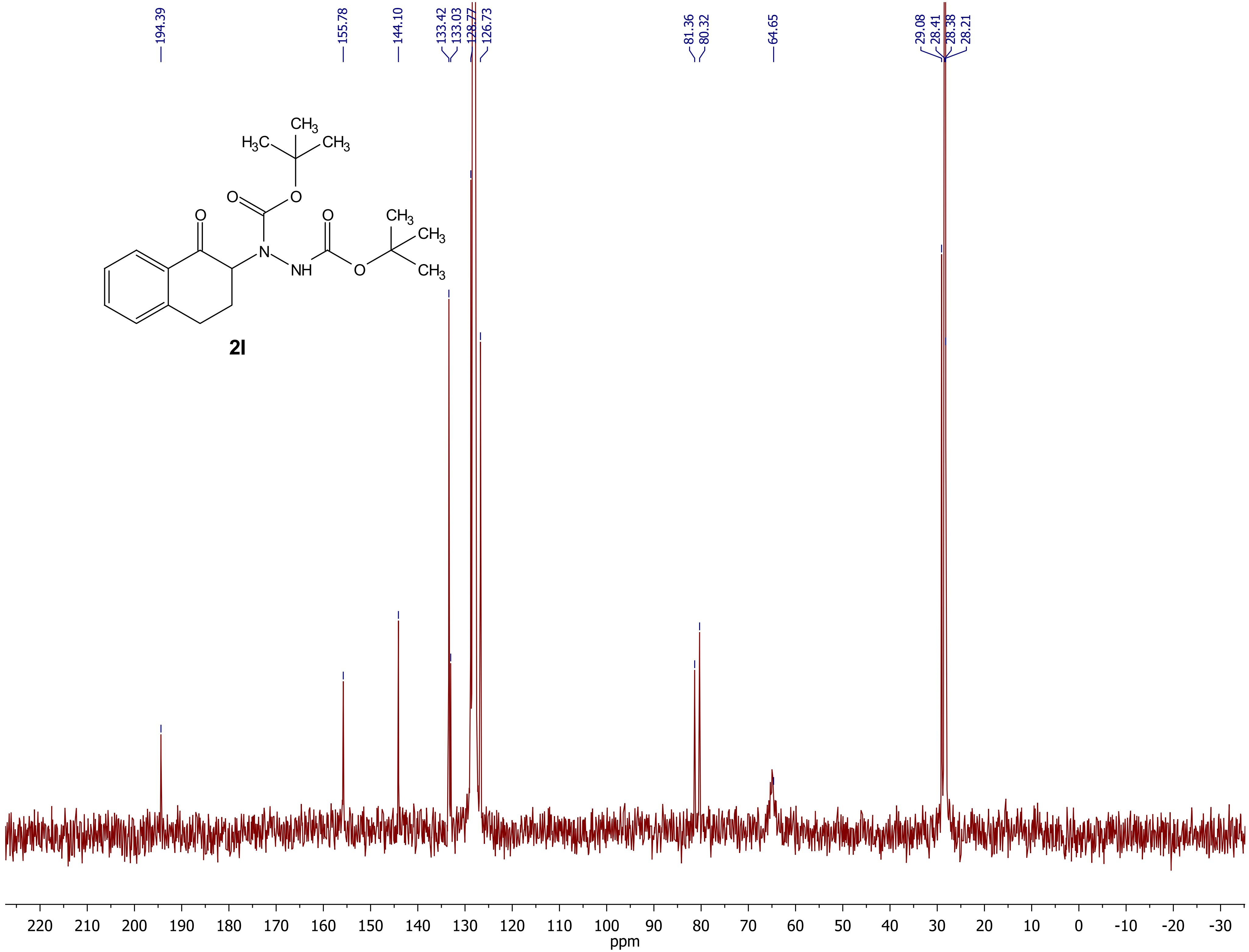
194.39
155.78
144.10
133.42
133.03
128.77
126.73
81.36
80.32
64.65
29.08
28.41
28.38
28.21
2l
220 210 200 190 180 170 160 150 140 130 120 110 100 90 80 70 60 50 40 30 20 10 0 -10 -20 -30
ppm

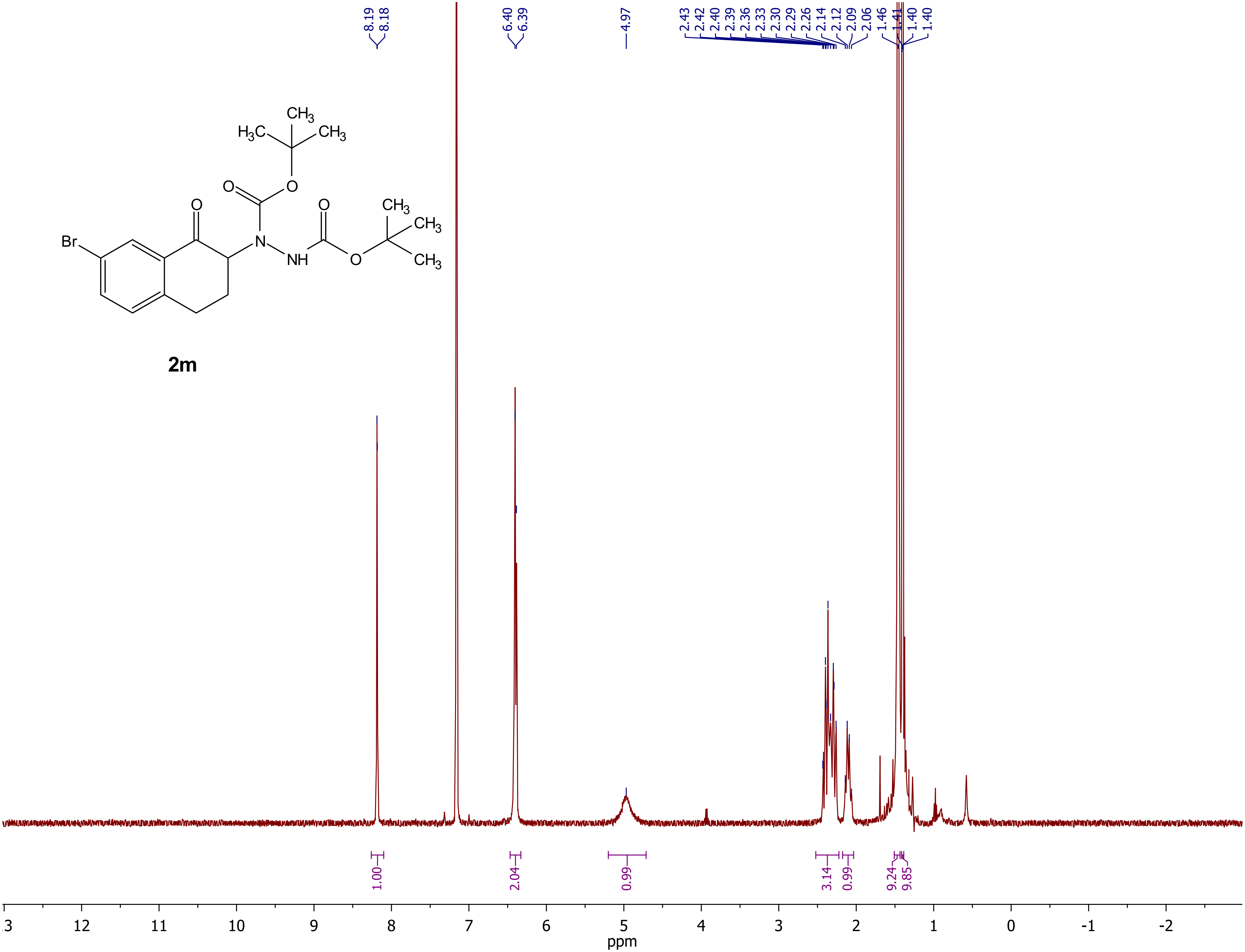
8.19
8.18
6.40
6.39
4.97
2.43
2.42
2.40
2.39
2.36
2.33
2.30
2.29
2.26
2.14
2.12
2.09
2.06
1.46
1.41
1.40
1.40
Br
O
N
NH
O
O
O
O
CH_3
H_3C
CH_3
CH_3
CH_3
CH_3
2m
1.00
2.04
0.99
3.14
0.99
9.24
9.85
13
12
11
10
9
8
7
6
5
4
3
2
1
0
-1
-2
ppm

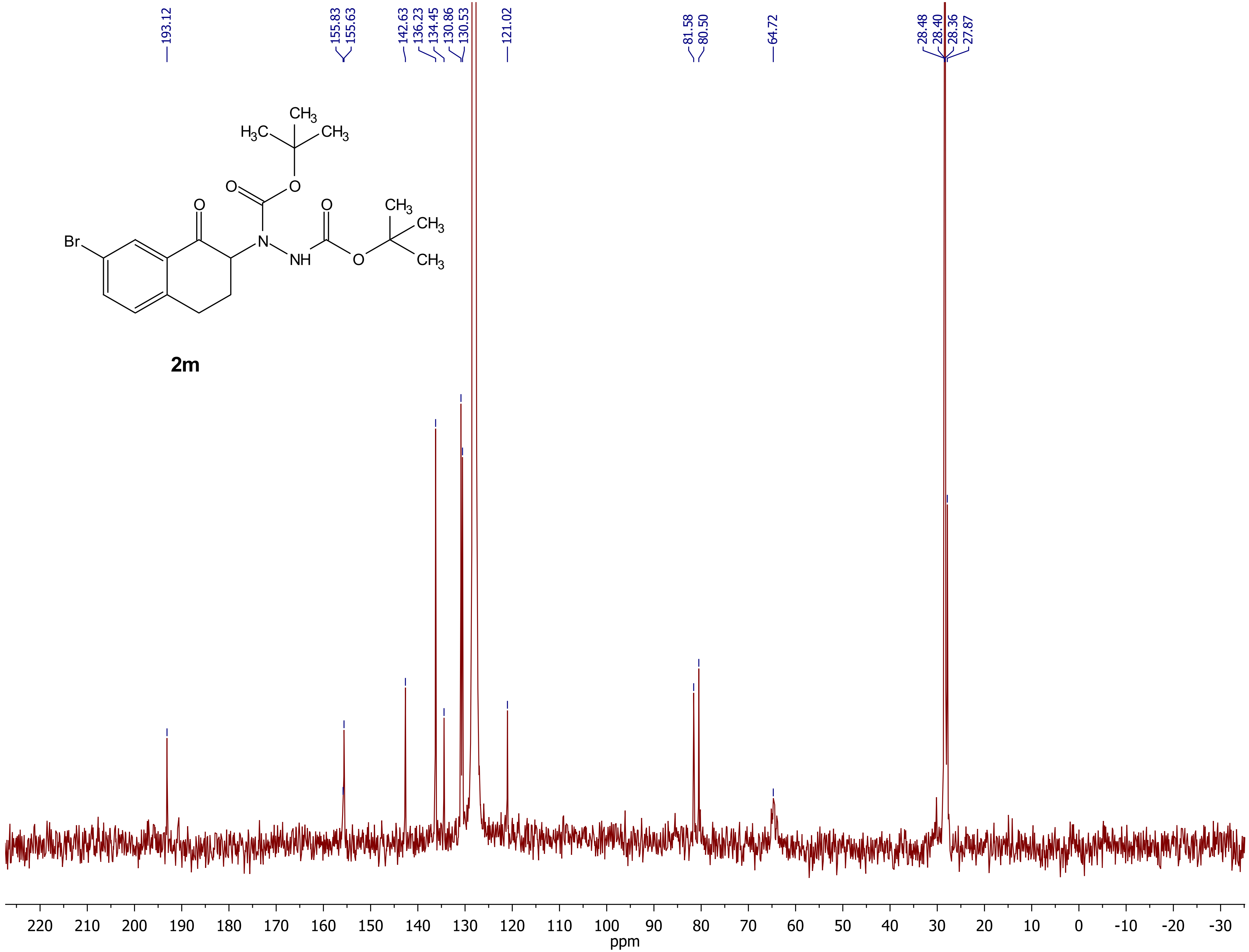

193.12
155.83
155.63
142.63
136.23
134.45
130.86
130.53
121.02
81.58
80.50
64.72
28.48
28.40
28.36
27.87
Br
O
N
NH
H_3C
CH_3
220 210 200 190 180 170 160 150 140 130 120 110 100 90 80 70 60 50 40 30 20 10 0 -10 -20 -30
ppm

2m

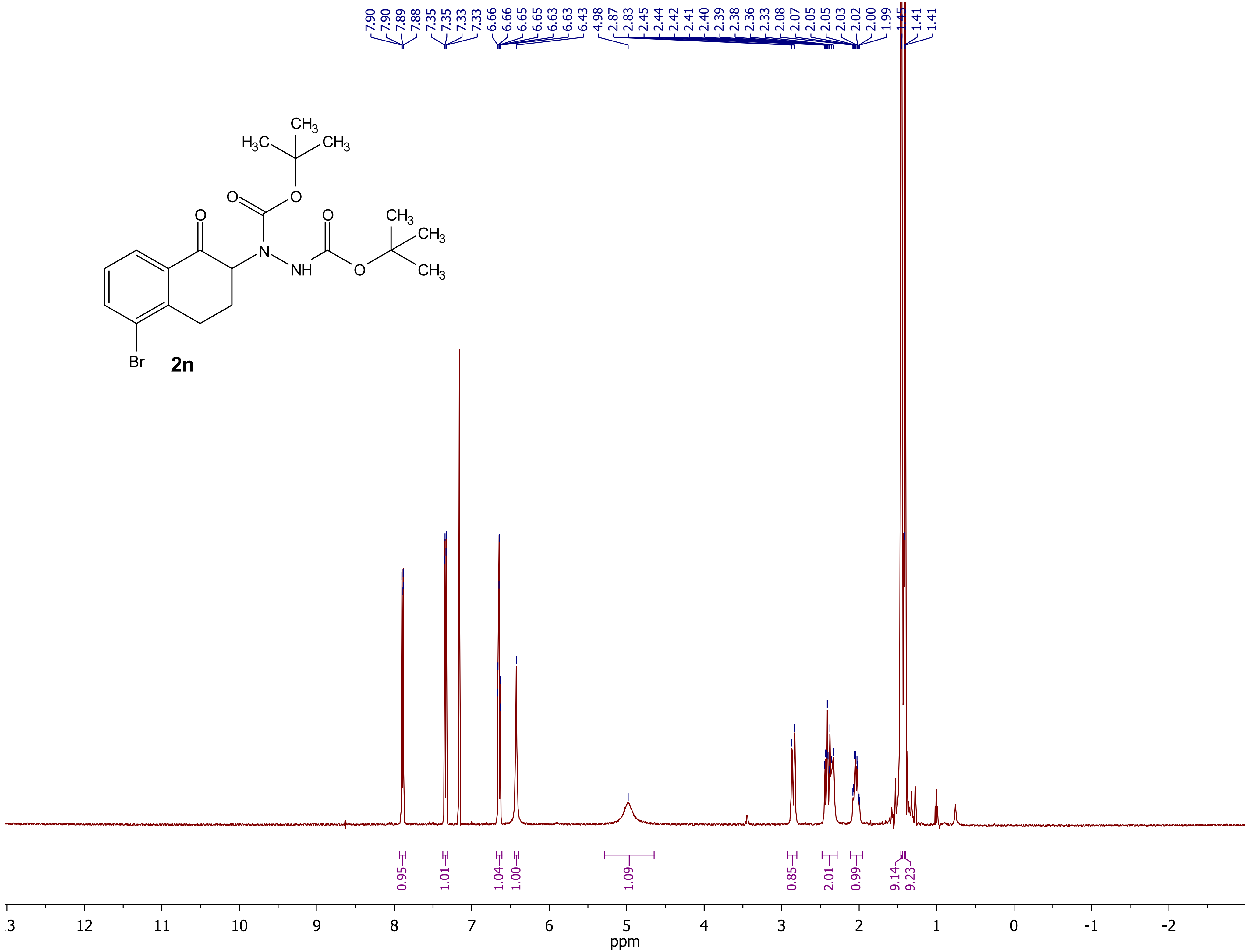

7.90
7.90
7.89
7.88
7.35
7.35
7.33
7.33
6.66
6.66
6.65
6.65
6.63
6.63
6.43
4.98
2.87
2.83
2.45
2.44
2.42
2.41
2.40
2.39
2.38
2.36
2.33
2.08
2.07
2.05
2.05
2.03
2.02
2.00
1.99
1.45
1.41
1.41
0.95
1.01
1.04
1.00
1.09
0.85
2.01
0.99
9.14
9.23
ppm

2n

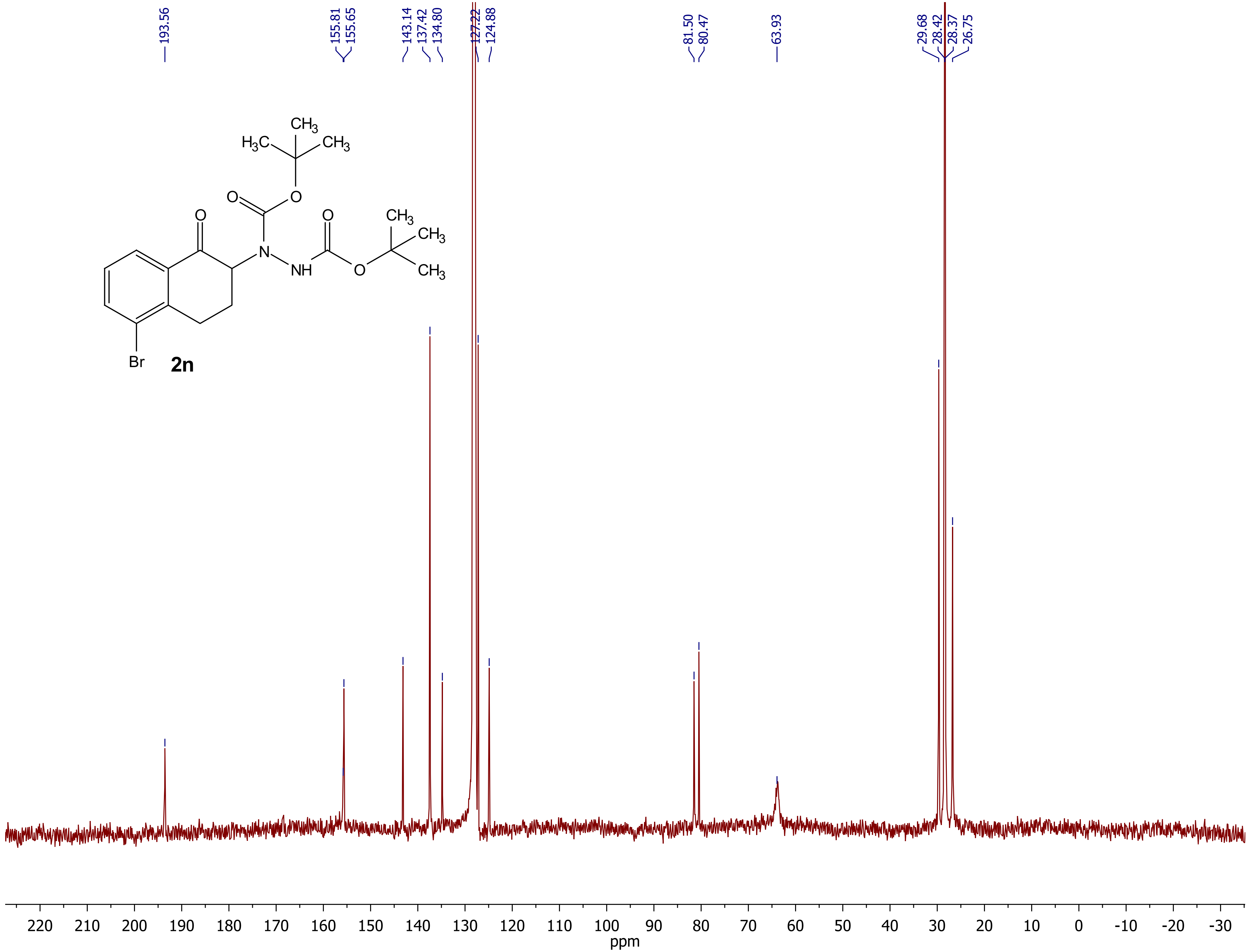

193.56
155.81
155.65
143.14
137.42
134.80
127.22
124.88
81.50
80.47
63.93
29.68
28.42
28.37
26.75
CH3
H3C
CH3
O
O
O
O
CH3
CH3
N
NH
O
CH3
Br
2n
220 210 200 190 180 170 160 150 140 130 120 110 100 90 80 70 60 50 40 30 20 10 0 -10 -20 -30
ppm

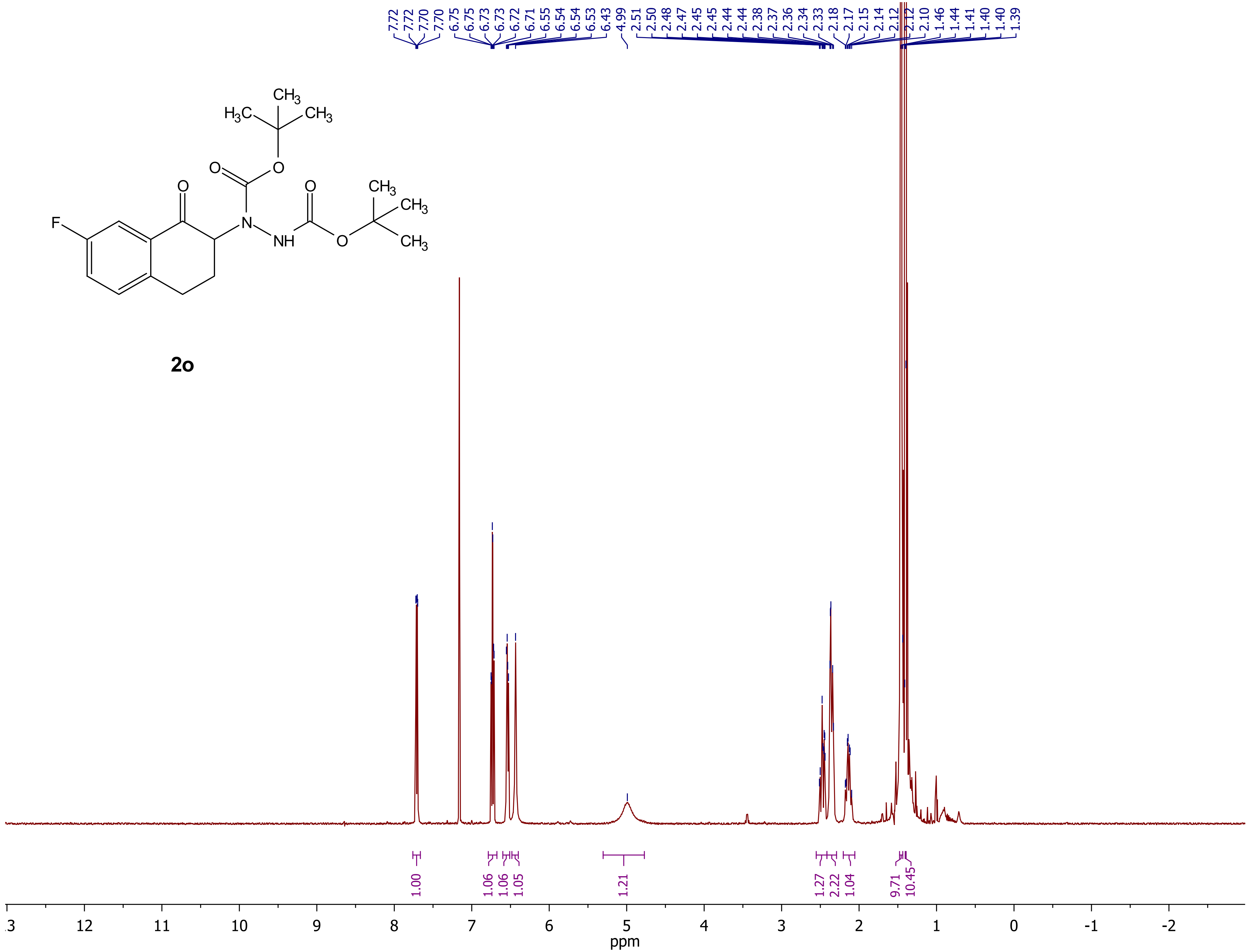

7.72
7.72
7.70
7.70
6.75
6.75
6.73
6.73
6.72
6.71
6.55
6.54
6.54
6.53
6.43
4.99
2.51
2.50
2.48
2.47
2.45
2.45
2.44
2.44
2.38
2.37
2.36
2.34
2.33
2.18
2.17
2.15
2.14
2.12
2.12
2.10
1.46
1.44
1.41
1.40
1.40
1.39
F
O
O
O
N
NH
O
O
H_3C
CH_3
CH_3
CH_3
CH_3
CH_3
1.00
1.06
1.06
1.05
1.21
1.27
2.22
1.04
9.71
10.45
ppm

2o

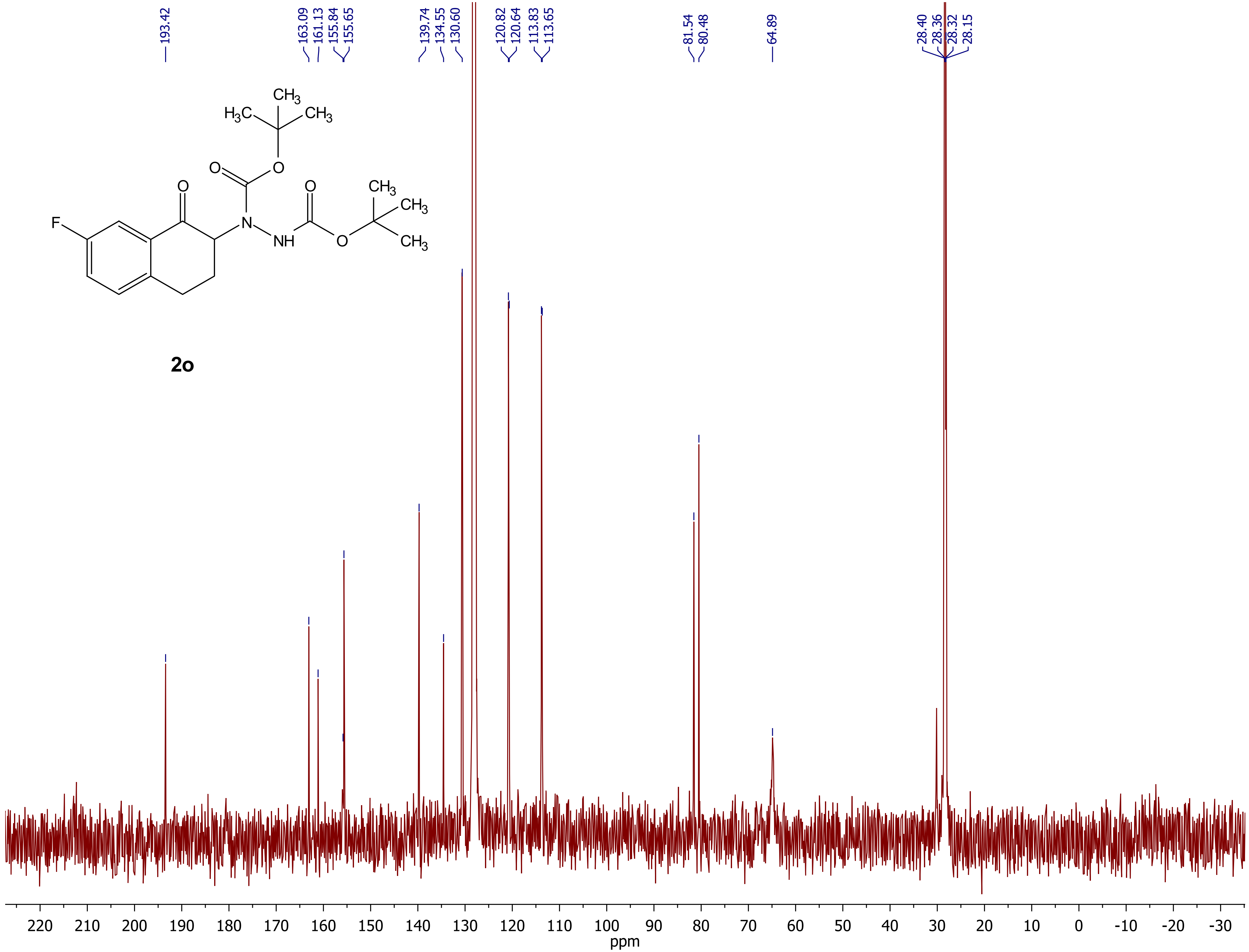
193.42
163.09
161.13
155.84
155.65
139.74
134.55
130.60
120.82
120.64
113.83
113.65
81.54
80.48
64.89
28.40
28.36
28.32
28.15
2o
220 210 200 190 180 170 160 150 140 130 120 110 100 90 80 70 60 50 40 30 20 10 0 -10 -20 -30
ppm

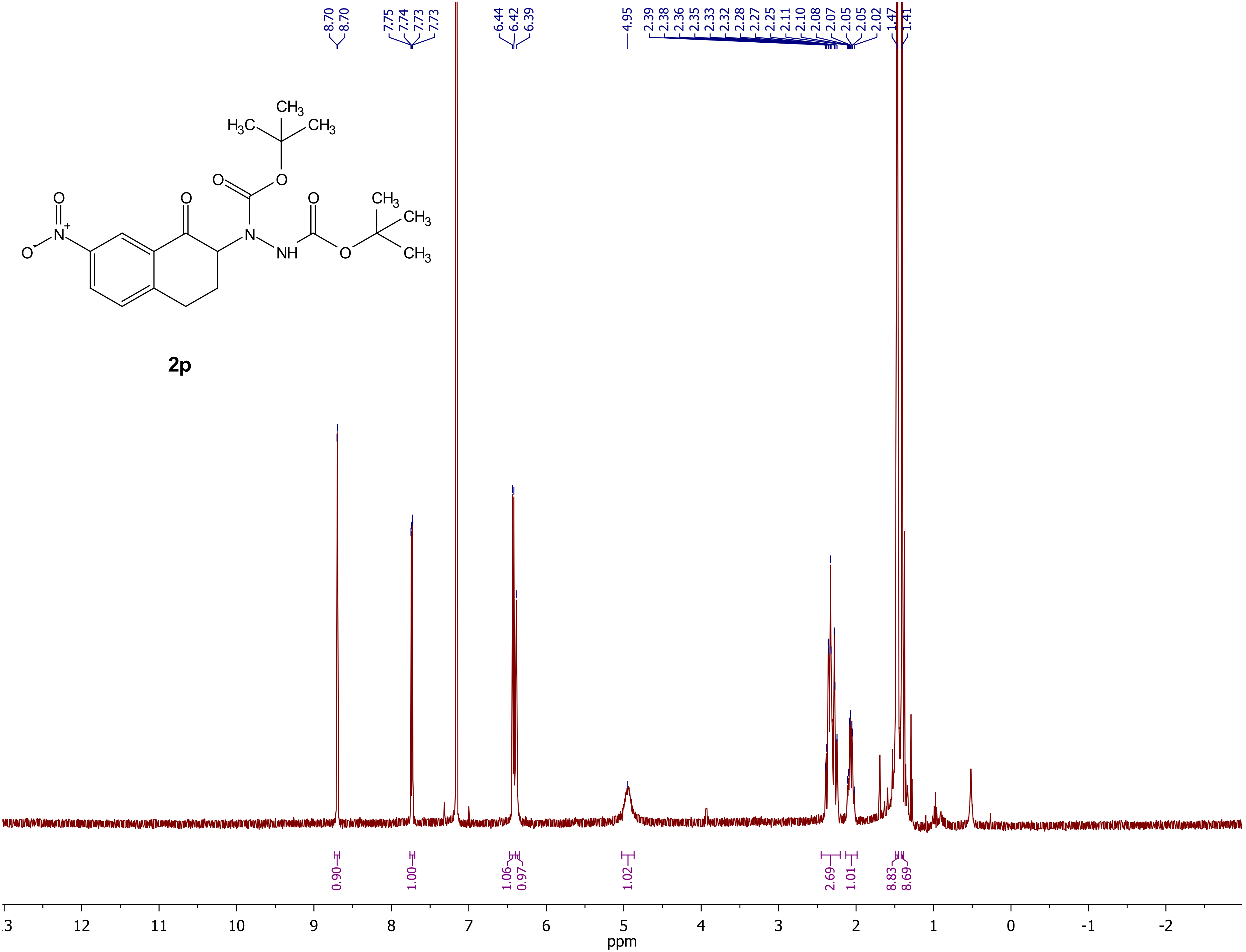

8.70
8.70
7.75
7.74
7.73
7.73
6.44
6.42
6.39
4.95
2.39
2.38
2.36
2.35
2.33
2.32
2.28
2.27
2.25
2.11
2.10
2.08
2.07
2.05
2.05
2.02
1.47
1.41
2p
0.90
1.00
1.06
0.97
1.02
2.69
1.01
8.83
8.69
ppm

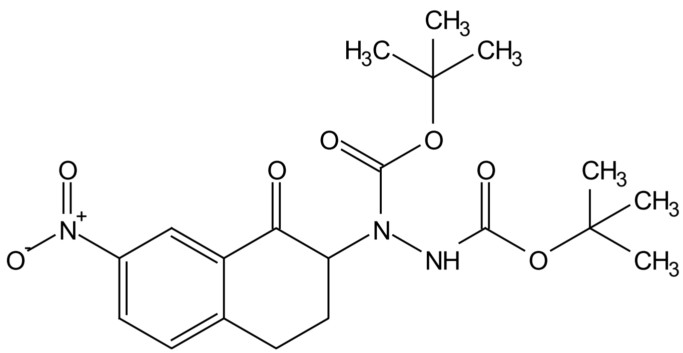

2p

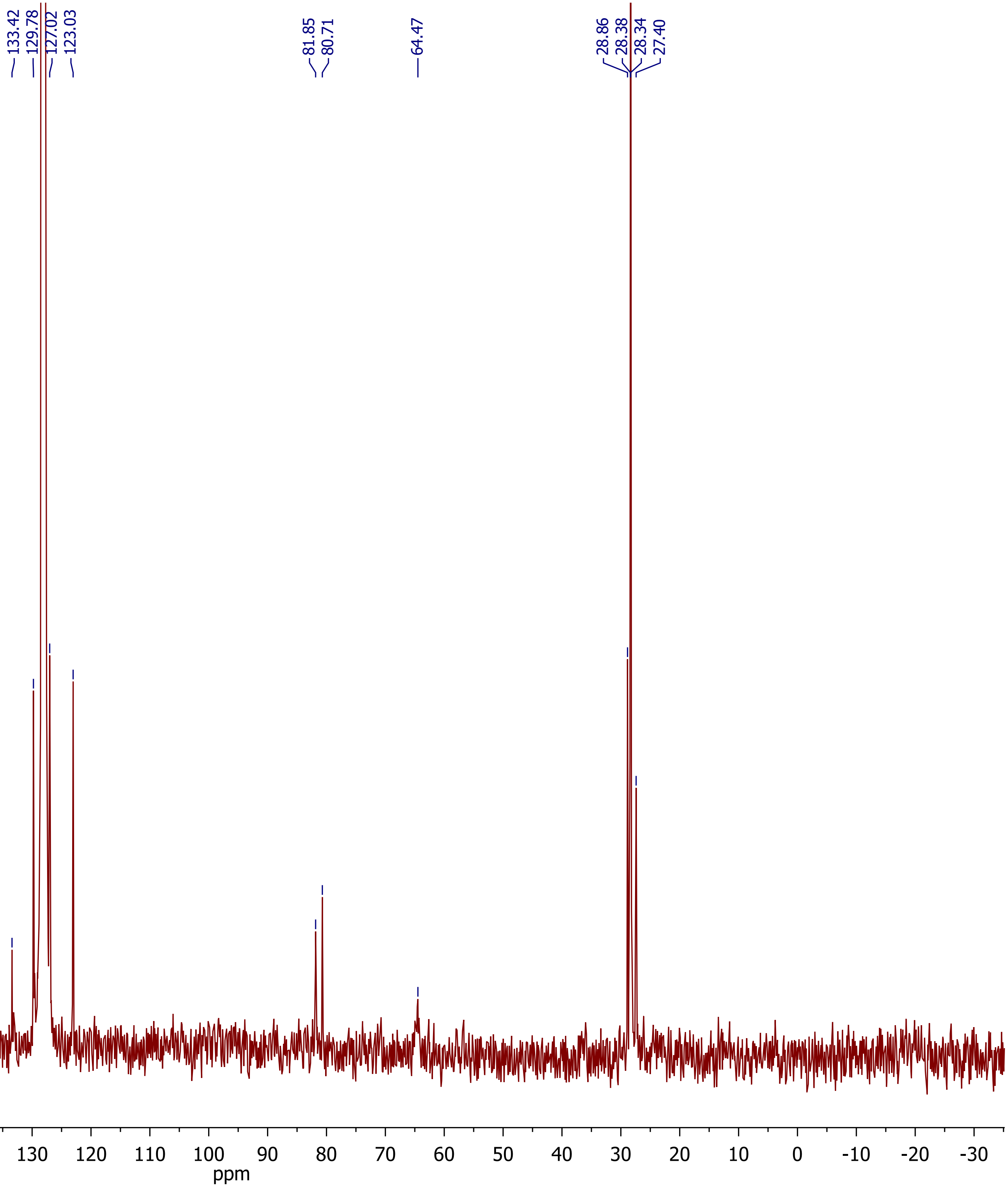

192.47
149.64
147.81
133.42
129.78
127.02
123.03
81.85
80.71
64.47
28.86
28.38
28.34
27.40
220
210
200
190
180
170
160
150
140
130
120
110
100
90
80
70
60
50
40
30
20
10
0
-10
-20
-30
ppm

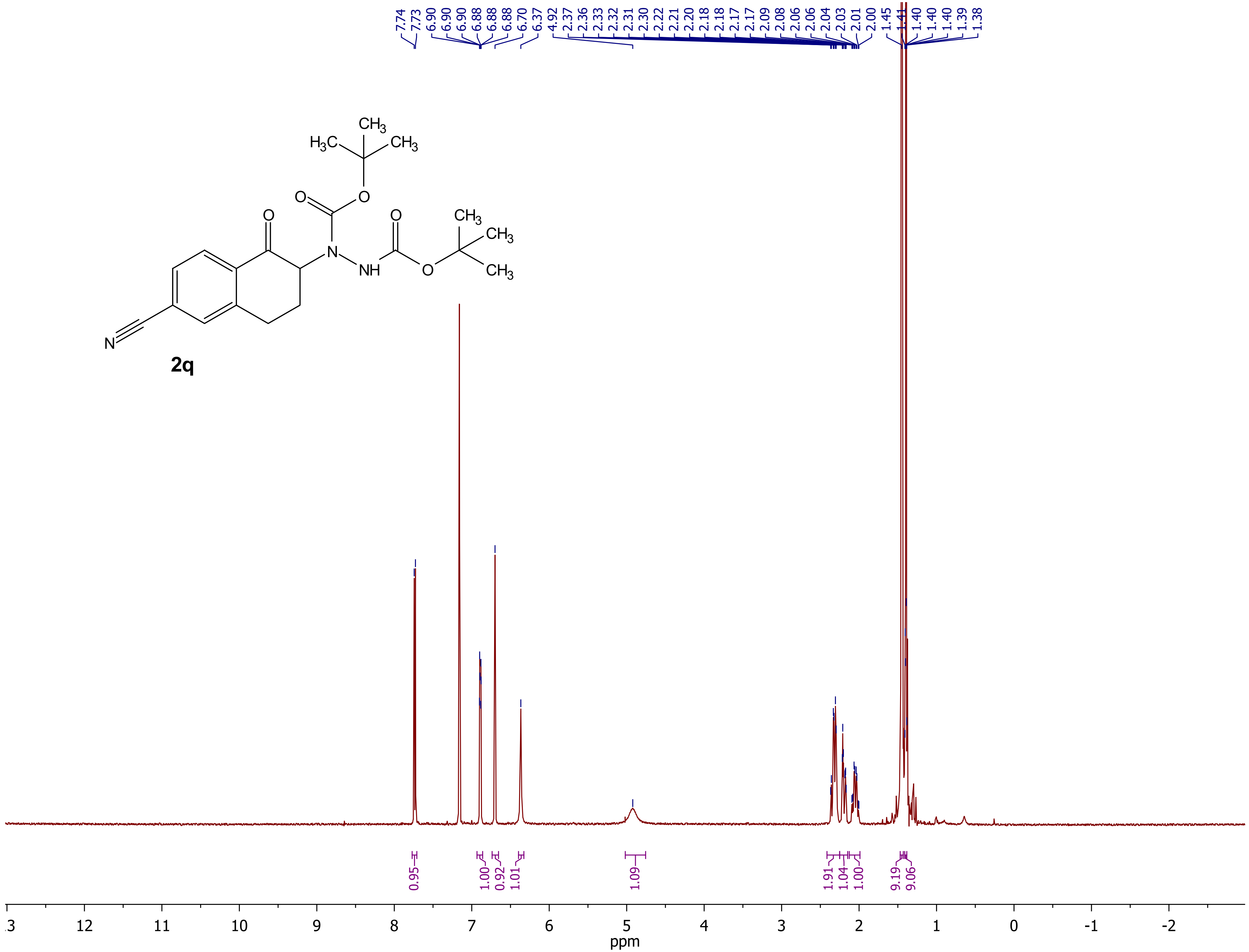
7.74
7.73
6.90
6.90
6.90
6.88
6.88
6.88
6.70
6.37
4.92
2.37
2.36
2.33
2.32
2.31
2.30
2.22
2.21
2.20
2.18
2.18
2.17
2.17
2.09
2.08
2.06
2.06
2.04
2.03
2.01
2.00
1.45
1.41
1.40
1.40
1.40
1.39
1.38
0.95
1.00
0.92
1.01
1.09
1.91
1.04
1.00
9.19
9.06
ppm

2q

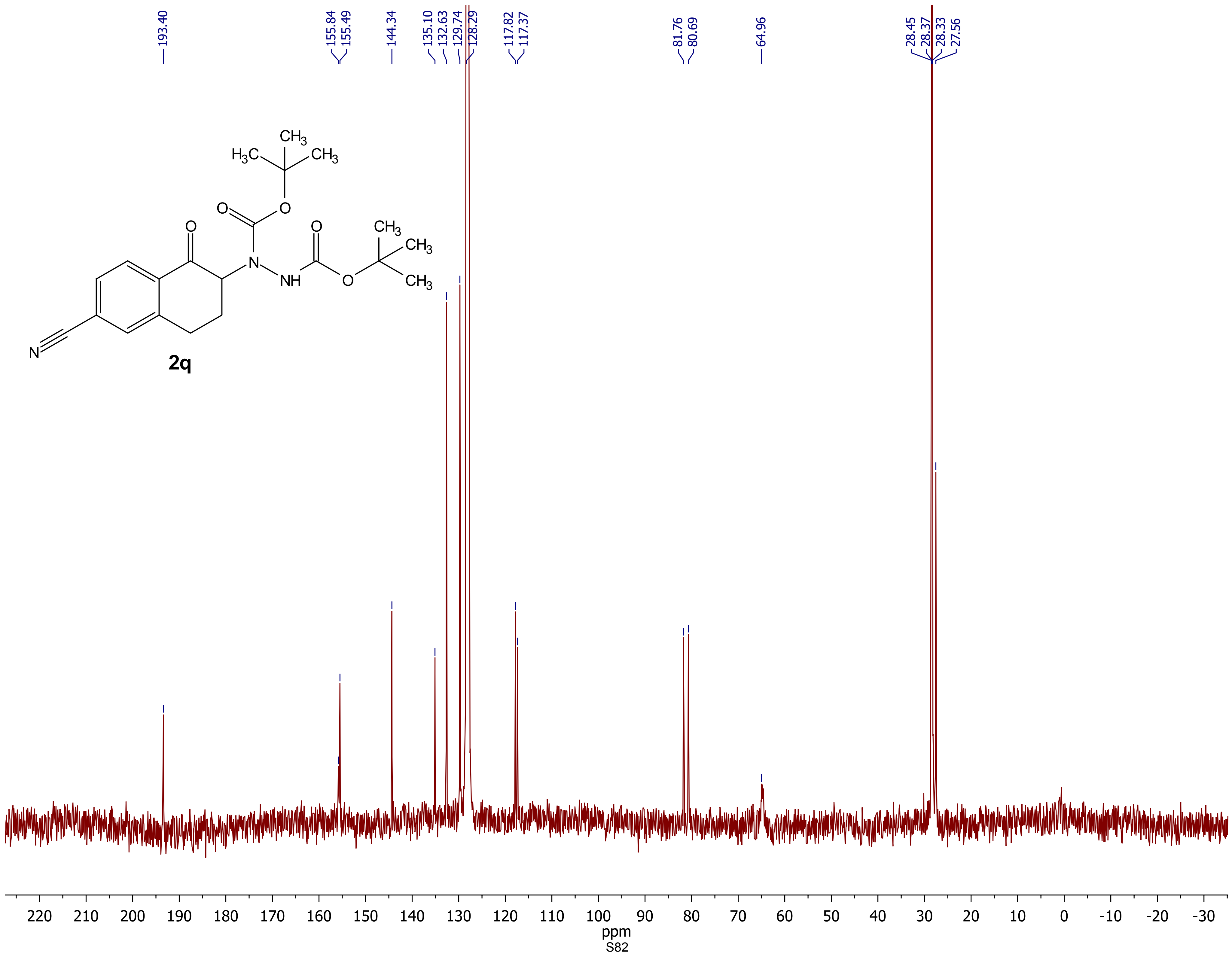
193.40
155.84
155.49
144.34
135.10
132.63
129.74
128.29
117.82
117.37
81.76
80.69
64.96
28.45
28.37
28.33
27.56
CH3
H3C
CH3
O
O
O
N
NH
O
CH3
CH3
O
CH3
N
2q
220 210 200 190 180 170 160 150 140 130 120 110 100 90 80 70 60 50 40 30 20 10 0 -10 -20 -30
ppm

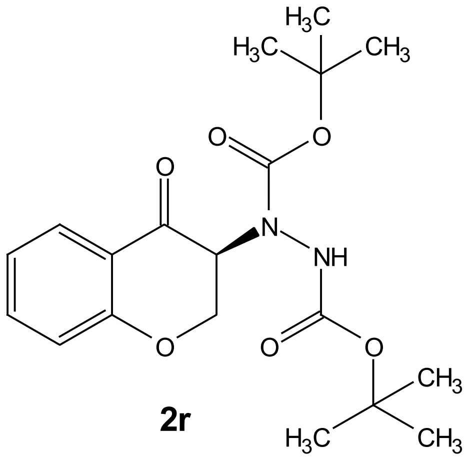

2r

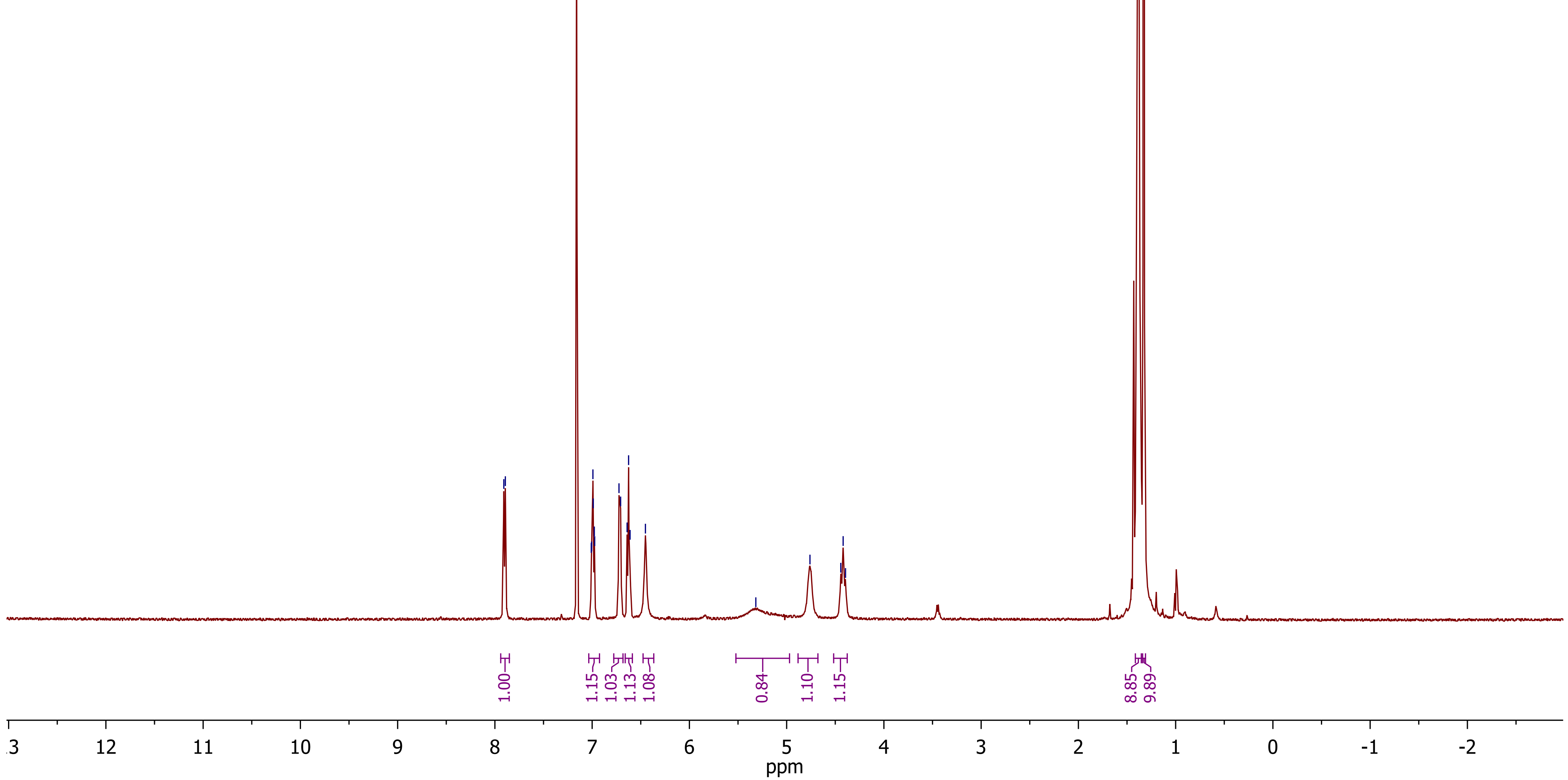
7.91
7.89
7.01
7.01
6.99
6.99
6.98
6.97
6.72
6.71
6.64
6.63
6.61
6.45
5.32
4.76
4.44
4.42
4.39
1.40
1.38
1.33
1.00
1.15
1.03
1.13
1.08
0.84
1.10
1.15
8.85
9.89
ppm

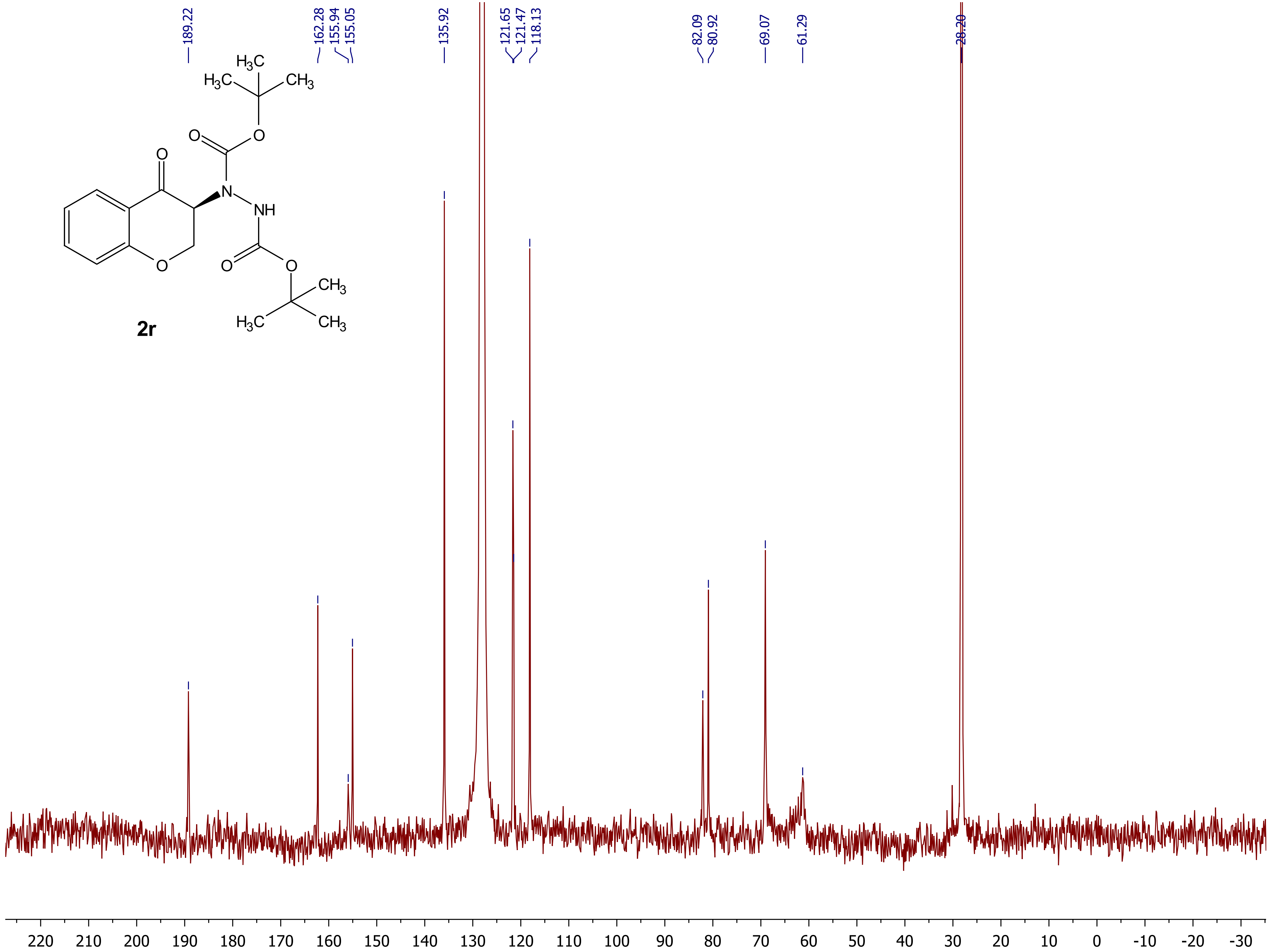

189.22
162.28
155.94
155.05
135.92
121.65
121.47
118.13
82.09
80.92
69.07
61.29
28.20
2r
220 210 200 190 180 170 160 150 140 130 120 110 100 90 80 70 60 50 40 30 20 10 0 -10 -20 -30
ppm

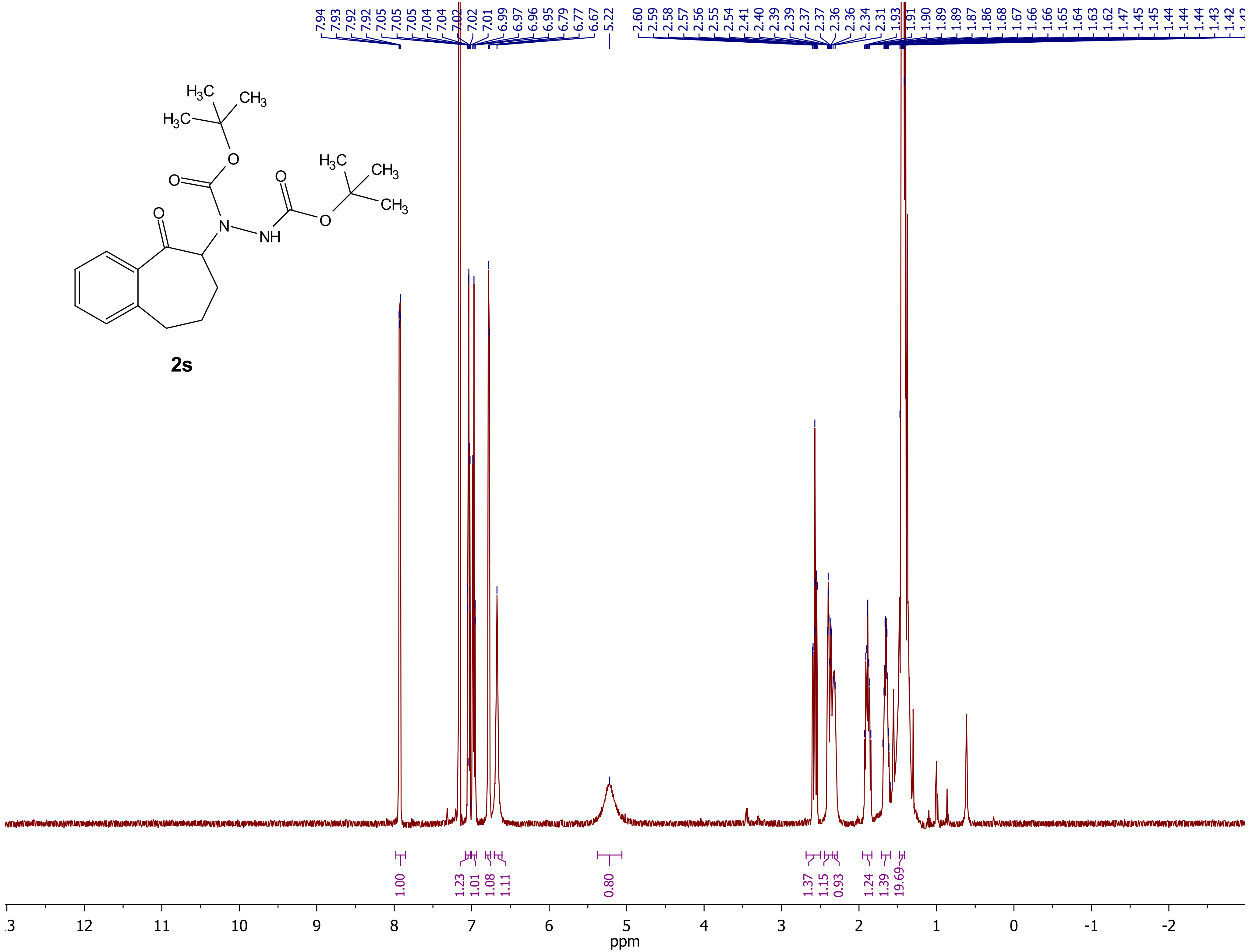
7.94 7.93 7.92 7.92 7.05 7.05 7.05 7.04 7.04 7.02 7.02 7.01 6.99 6.97 6.96 6.95 6.79 6.77 6.67 5.22
2.60 2.59 2.58 2.57 2.56 2.55 2.54 2.41 2.40 2.39 2.39 2.37 2.37 2.36 2.36 2.34 2.31 1.93 1.91 1.90 1.89 1.89 1.87 1.86 1.68 1.67 1.66 1.66 1.65 1.64 1.63 1.62 1.47 1.45 1.45 1.44 1.44 1.44 1.43 1.42
H3C
CH3
O
NH
N
CH3
2s
1.00 1.23 1.01 1.08 1.11 0.80 1.37 1.15 0.93 1.24 1.39 19.69
12 11 10 9 8 7 6 5 4 3 2 1 0 -1 -2
ppm

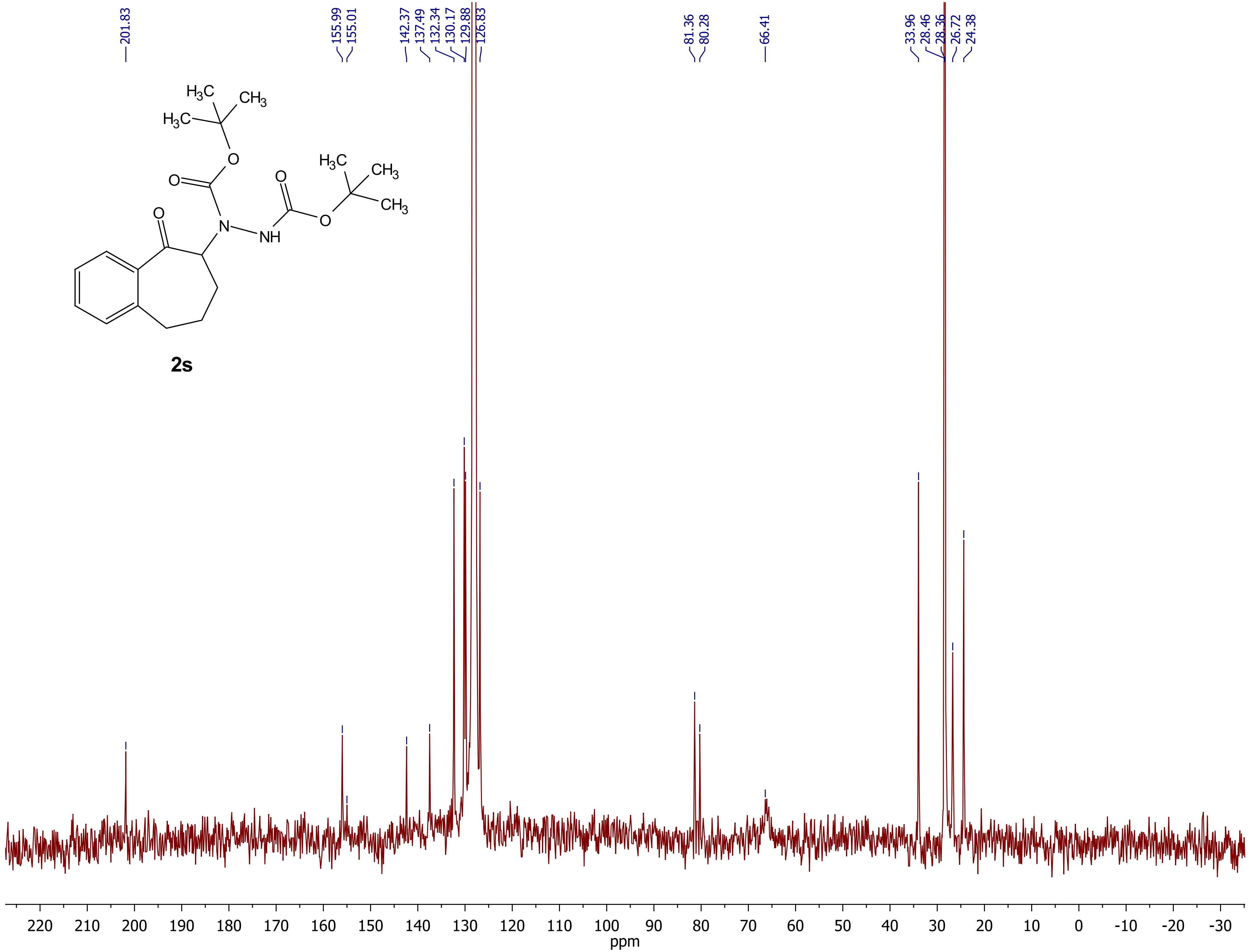
201.83
155.99
155.01
142.37
137.49
132.34
130.17
129.88
126.83
81.36
80.28
66.41
33.96
28.46
28.36
26.72
24.38
H3C
CH3
H3C
O
O
H3C
CH3
O
CH3
O
N
NH
O
O
2s
220 210 200 190 180 170 160 150 140 130 120 110 100 90 80 70 60 50 40 30 20 10 0 -10 -20 -30
ppm

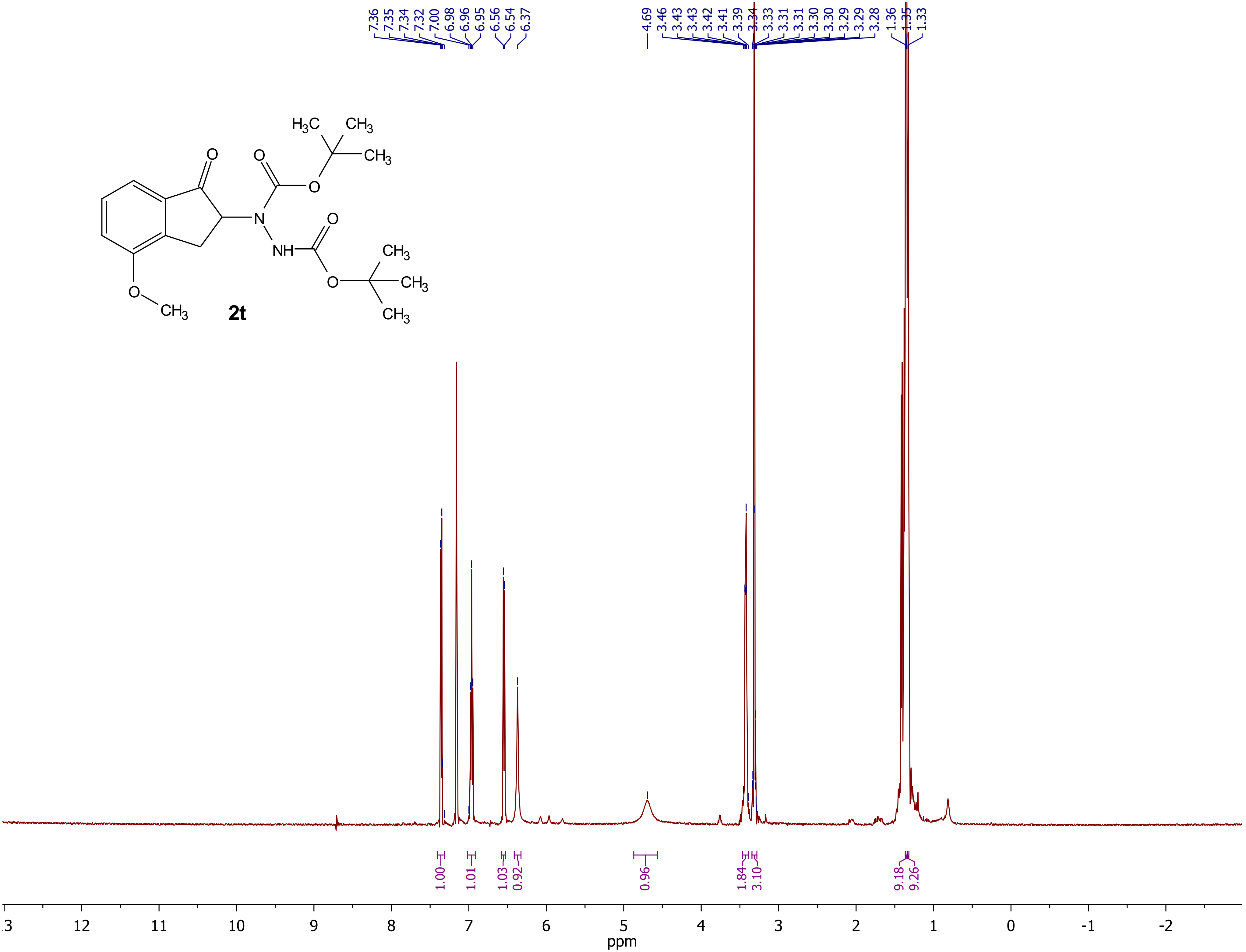
7.36
7.35
7.34
7.32
7.00
6.98
6.96
6.95
6.56
6.54
6.37
4.69
3.46
3.43
3.43
3.42
3.41
3.39
3.34
3.33
3.31
3.31
3.30
3.30
3.29
3.29
3.28
1.36
1.35
1.33
H_3C
CH_3
CH_3
O
O
O
N
O
NH
CH_3
O
CH_3
O
CH_3
CH_3
2t
1.00
1.01
1.03
0.92
0.96
1.84
3.10
9.18
9.26
13
12
11
10
9
8
7
6
5
4
3
2
1
0
-1
-2
ppm

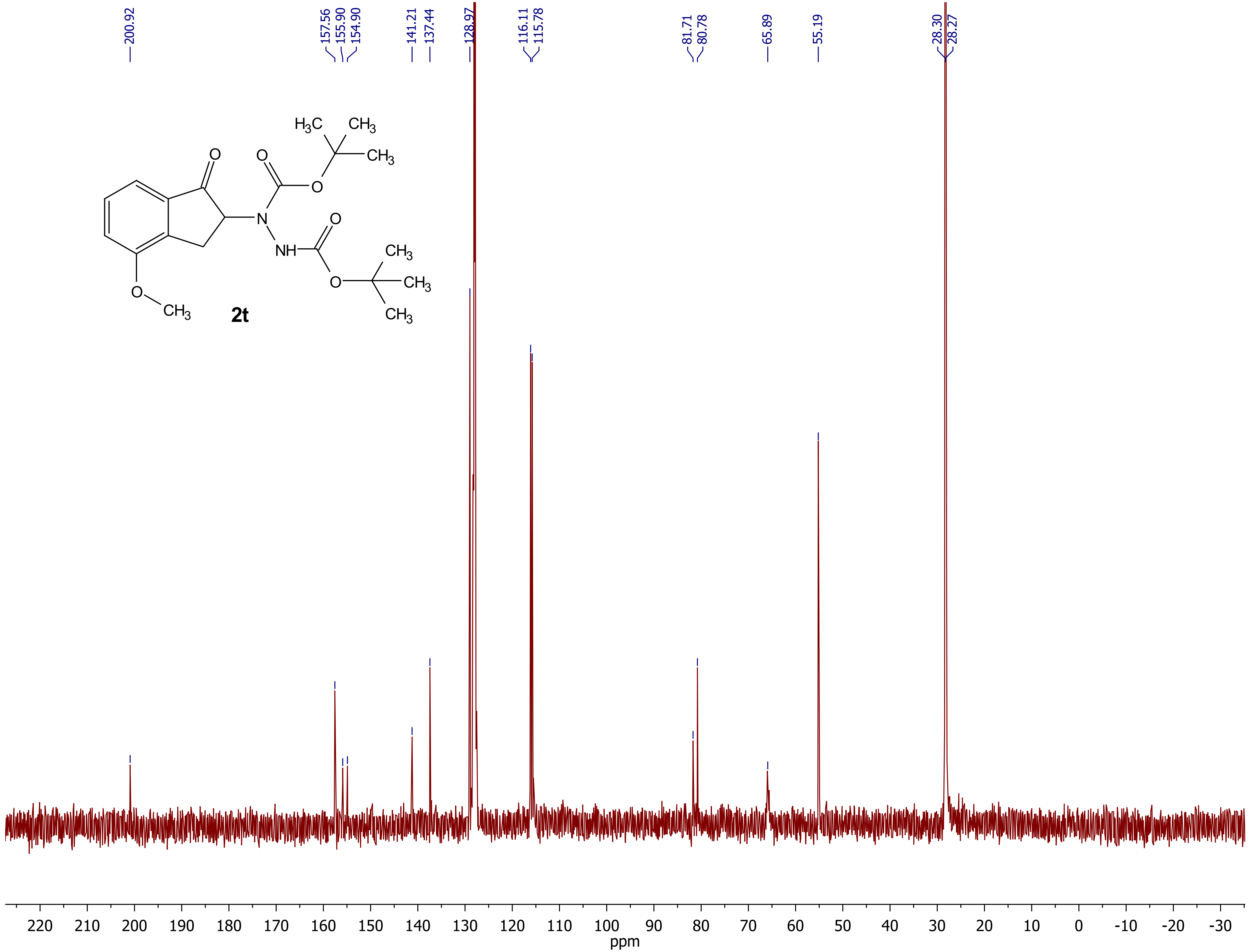
200.92
157.56
155.90
154.90
141.21
137.44
128.97
116.11
115.78
81.71
80.78
65.89
55.19
28.30
28.27
2t
220 210 200 190 180 170 160 150 140 130 120 110 100 90 80 70 60 50 40 30 20 10 0 -10 -20 -30
ppm

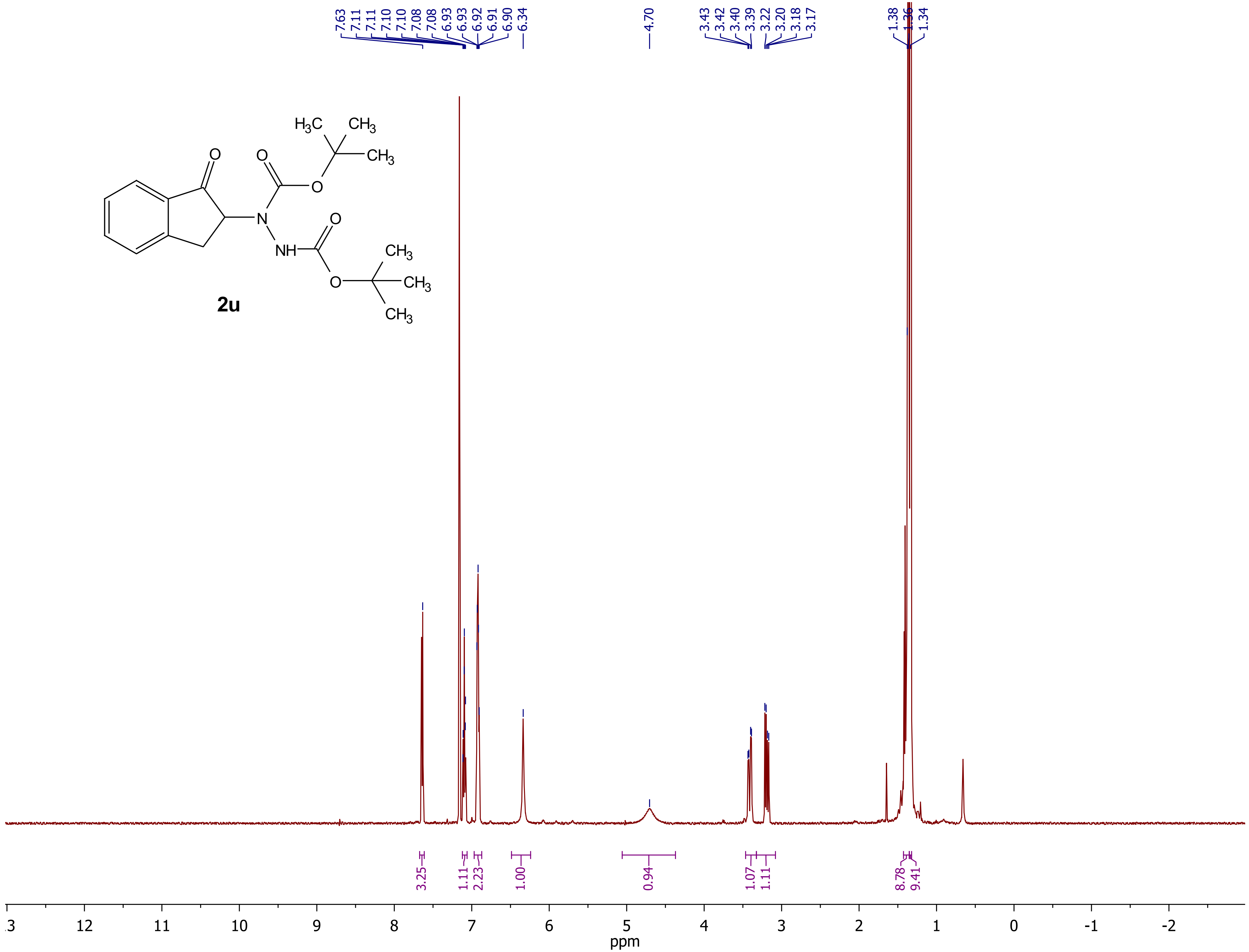

7.63
7.11
7.11
7.10
7.10
7.08
6.93
6.93
6.92
6.91
6.90
6.34
4.70
3.43
3.42
3.40
3.39
3.22
3.20
3.18
3.17
1.38
1.36
1.34
2u
3.25
1.11
2.23
1.00
0.94
1.07
1.11
8.78
9.41
ppm

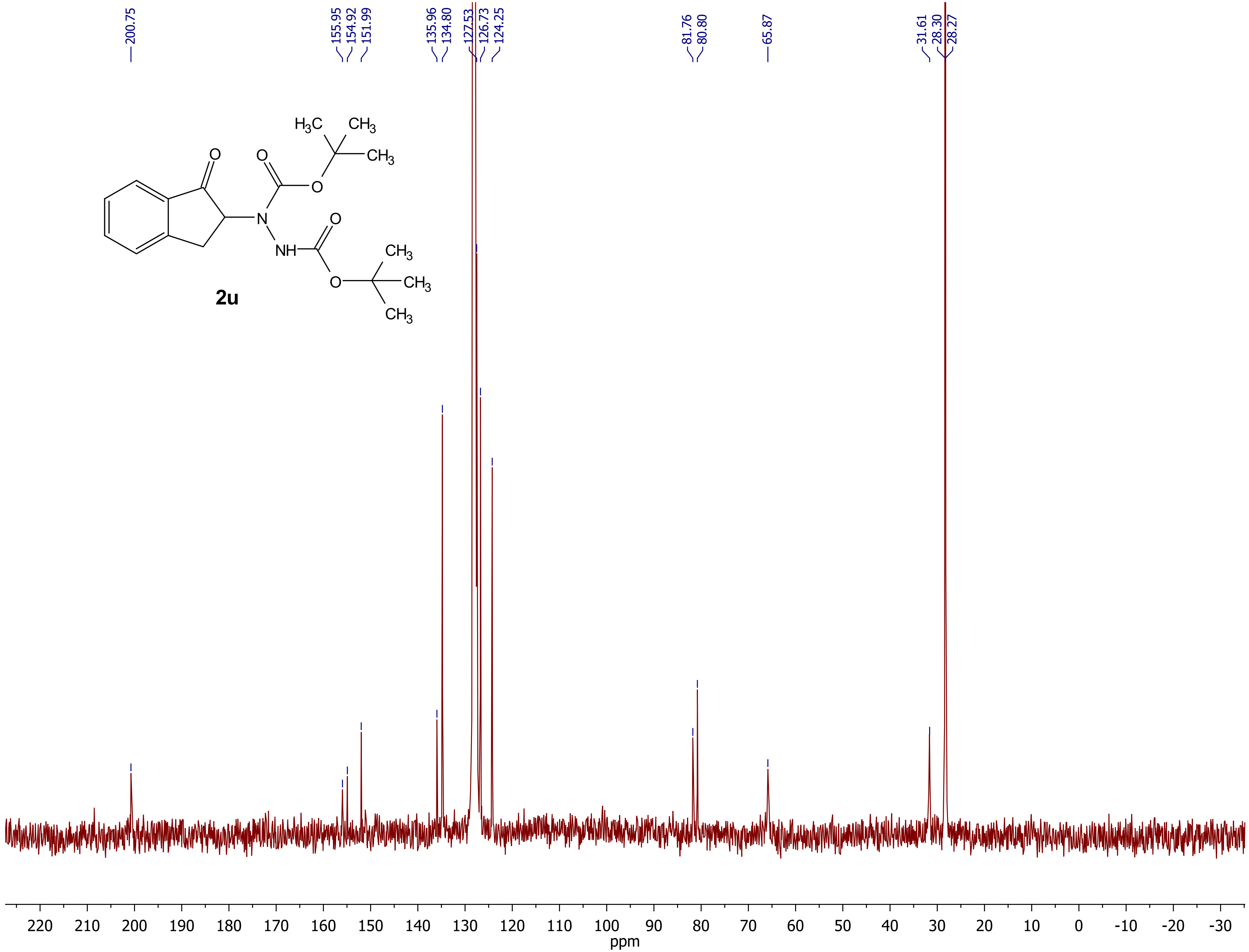
200.75
155.95
154.92
151.99
135.96
134.80
127.53
126.73
124.25
81.76
80.80
65.87
31.61
28.30
28.27
H3C
CH3
CH3
O
O
O
N
NH
O
CH3
O
CH3
CH3
2u
220 210 200 190 180 170 160 150 140 130 120 110 100 90 80 70 60 50 40 30 20 10 0 -10 -20 -30
ppm
